# α-Selective *syn*-Carbotrifluoromethylthiolation of Alkynes

**DOI:** 10.1021/acs.orglett.5c00570

**Published:** 2025-03-03

**Authors:** Prachi Shah, Wojciech Chaładaj

**Affiliations:** Institute of Organic Chemistry, Polish Academy of Sciences, Kasprzaka 44/52, 01-224 Warsaw, Poland

## Abstract

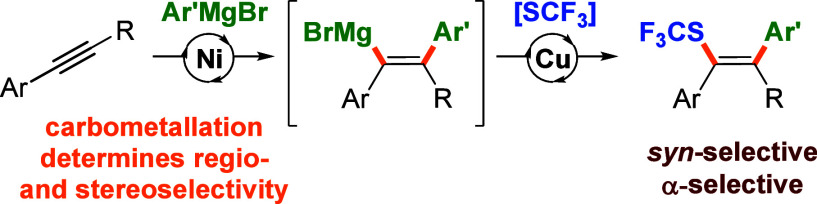

Trifluoromethylthiolative difunctionalization of alkynes typically proceeds in an *anti*-fashion delivering the SCF_3_ group in the β-position (*anti*-Markovnikov). Herein, we disclose a vicinal *syn*-arylation-trifluoromethylthiolation of alkynes enabling α-selective introduction of the SCF_3_ group (Markovnikov). The unique selectivity was achieved via a merge of Ni-catalyzed carbomagnesiation with a subsequent Cu-mediated trifluoromethylthiolation of the resulting vinyl-magnesium species. The former component of the sequential process determines both the regio- and stereoselectivity of the overall transformation.

Fluorinated organic compounds feature a range of unique properties originating in small size and high electronegativity of the fluorine atom capable of the formation of particularly strong C–F bonds.^1^ One of the important fluorine-containing groups is the strongly electron-deficient trifluoromethylthio group (CF_3_S), which exerts significant impact on physical, chemical, and biological properties of organic molecules. From the pharmaceutical and agrochemical perspective, better bioavailability and metabolic stability are particularly relevant.^2^ Therefore, development of synthetic methods aimed at efficient and selective installation of the SCF_3_ group on the organic scaffolds has attracted considerable attention. Seminal reports utilizing hazardous, toxic reagents like CF_3_SH and CF_3_SCl date back to the early 1960s.^3^ More recently, numerous stable and easy to handle trifluoromethylthiolating agents were introduced, enabling functionalization of sp^3^, sp^2^, and sp centers.^4^

Trifluoromethylthio-substituted olefins are typically accessed through direct thiolation of alkenes^5^ or take advantage of the reactivity of properly prefunctionalized alkenes: vinyl halides,^6^ sulfonates, carboxylic acids,^7^ thiocyanates^8^ or boronic acids,^9^ among others (Scheme 1a). These methods, while mild and efficient, allow the introduction of just one substituent and often rely on prior synthesis of specifically functionalized precursors.

**Scheme 1:**
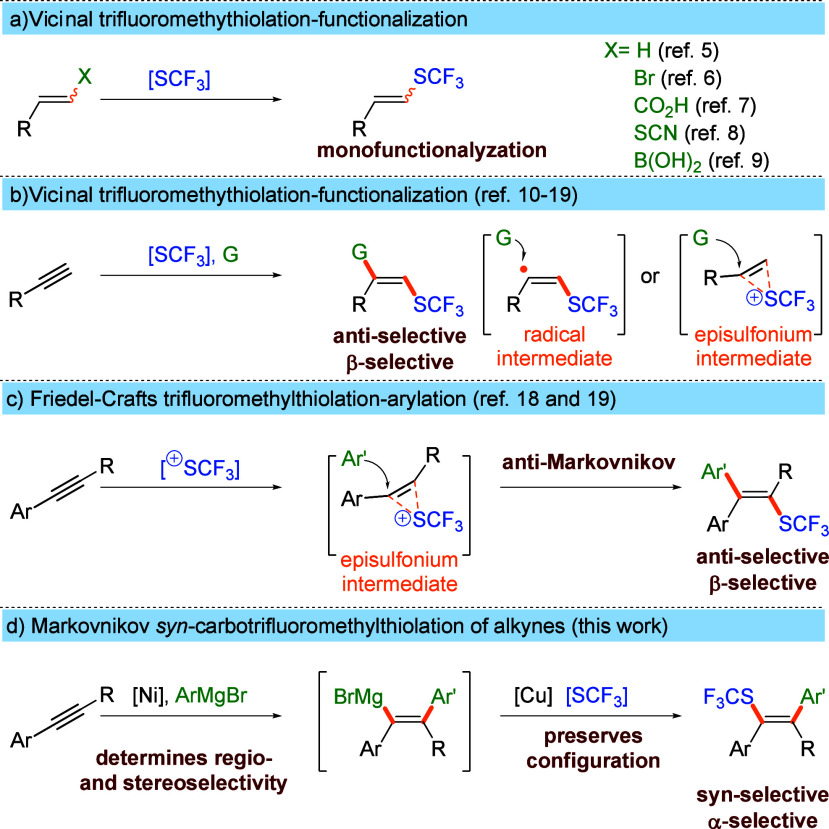
Access to SCF_3_-Substituted Olefins

Concomitant installations of multiple functional groups offer an attractive alternative, providing a significant increase in the molecular complexity in a single synthetic step. Recently, a handful of protocols were disclosed, allowing the addition of SCF_3_ to simple alkynes alongside intermolecular introduction of other moieties, including Cl,^10^ OH,^11^ OTs,^12^ CN,^13^ SO_2_Ph,^14^ Bpin,^15^ H,^16^ CF_2_COOEt,^17^ OTf,^18^ and arenes^18,19^ (Scheme 1b). These methods primarily rely either on the initial addition of the SCF_3_ radical to the alkyne or on an electrophilic manifold, involving an episulfonium intermediate. Both approaches offer excellent *anti*-selectivity and practically complete regioselectivity, originating from better stabilization of a radial or carbocationic center at the α position, thus placing SCF_3_ at the β position (anti-Markovnikov). These methods are often limited to terminal alkynes, while showing poor reactivity and selectivity when applied to internal alkynes.

To date, only two α-selective trifluoromethylthiolations were achieved through a cascade initiated by the addition of specifically tailored, relatively stable radicals (·CF_2_CO_2_Et^17^ or ·SO_2_Ph^14^), followed by *anti*-selective thiolation of the resulting vinyl radical, which severely confines the generality of the approach.

Trifluoromethylthiolation accompanied by arylation was achieved only *via* the Friedel–Crafts strategy (Scheme 1c).^18,19^ Due to involvement of the episulfonium intermediate, the process features excellent selectivity toward anti-Markovnikov products. However, the scope suffers from a narrow array of electron rich arenes installed.

We envisioned that carbometalation^20^ merged with trifluoromethylthiolation of the metal-vinyl intermediate would provide analogous products with opposite regio- and stereoselectivity (Scheme 1d). The selectivity of the entire process was controlled in the former step, while the latter is expected to proceed with retention of the configuration. Herein, we disclose a method for α-selective trifluoromethylthiolation of alkynes accompanied by the installation of electronically varied arenes.

Initial trials for trifluoromethylthiolation of model trisubstituted vinyl-magnesium compound **2** accessed *via* addition of phenylmagnesium bromide to 1-phenylhexyne (**1**) turned out to be unproductive with reagent **5**, as well as with more electrophilic trifluoromethylthiolatingating agents **6**–**7** (Table 1, entry 2). The results were surprising and disappointing, as **5** was reported to effectively transfer SCF_3_ to a range of aryl magnesium species, even at −78 °C. We suppose that the lack of reactivity could be attributed to steric reasons.

**Table 1:**
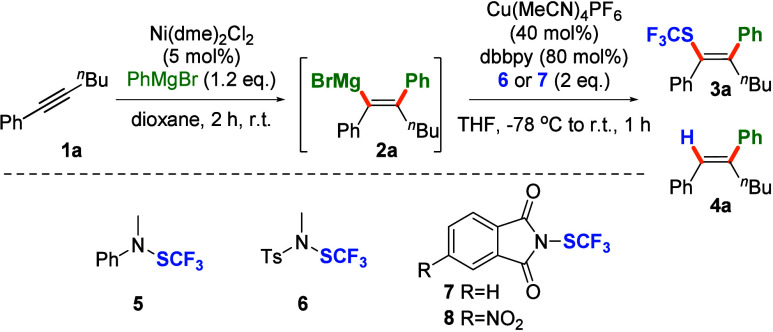
Optimization of Trifluoromethylthiolation of *In Situ* Generated Vinyl-Magnesium Species **2**

			Yield	
Entry	[SCF_3_]	Variation from Benchmark Conditions	**3**	**4**
1	**7**		60%	6%
2	**6** or **7**	No Cu and ligand	0%	82%
3	**6**	1 eq. of CuBr·Me_2_S, no ligand	18%	44%
4	**6**	20 mol % of Cu (Cu:L 1:2)	42%	10%
5	**6**	1 eq. of Cu (Cu:L 1:2)	56%	6%
6	**6**		55%	9%
7	**8**		60%	6%
8	**5**		0%	22%
9	**7**	1.1 equiv of **7**	33%	7%
10	**7**	1.5 equiv of **7**	45%	6%
11	**7**	Run at –78 °C	40%	8%
12	**7**	Run at r.t.	39%	6%
**13**^b^	**7**	**Cu, L, and 7 added to vinylmagnesium species**	**73%**	**8%**

a Reaction conditions: Ni(dme)Cl_2_ (5 mol %), phenyl hexyne (0.2 mmol), PhMgBr (1.2 equiv), dioxane (0.8 mL), 2 h, r.t., then Cu(MeCN)_4_ (40 mol %), dbppy (80 mol %), 6 or 7 (2 equiv), THF (0.6 mL), −78 °C to r.t., 1 h; yield determined by GC with mesitylene as an internal standard.

b Isolated yield.

Further attempts revealed that small amounts of expected SCF_3_-substituted olefin **3** were formed in the presence of Cu(I) salts and reagent **6** (Table 1, entry 3). Careful optimization of both steps of the model reaction, carbometalation of alkyne and trifluoromethylthiolation of the resulting vinylmagnesium species, brought us to satisfactory conditions (see the SI for details). Out of the tested ligands and copper sources, Cu(MeCN)_4_PF_6_ and 4,4′-bis(*tert*-butyl)-2,2′-bipyridine (dbbpy) in a 1:2 ratio performed best (Table 1, entry 1). Lowering the catalyst loading slightly decreased yield, while the efficiency of the process remained constant, even upon increasing the amount of the copper to 1 equiv (entries 4–6). The change of the trifluoromethylthiolating agent to phthalimide derivatives **7** and **8** resulted in comparable or slightly better results (entries 1 and 7). In contrast, less electrophilic reagent **5** delivered no expected product **3**, under otherwise similar conditions (entry 8).

Trifluoromethylthiolation was ineffective at −78 °C (entry 11). On the other hand, a reaction preformed at room temperature provided inferior yield, compared to standard protocol in which vinylmagnesium compound **2** was added to a solution of **7** and copper catalyst at −78 °C and carried out for 1 h at room temperature (entry 12). Further improvement in the yield was observed when copper, ligand, and **7** were sequentially added to a solution of vinyl-magnesium intermediate **2** at −78 °C and further carried out at room temperature, ultimately delivering **3** in 73% yield along with 8% of olefin **4**, presumably originating from hydrolysis of Grignard reagent during workup.

Having developed satisfactory conditions for the model reaction, we investigated the scope and limitations of the transformation (Scheme 2). In all cases, the expected highly substituted vinyltrifluoromethylsulfides **3** were isolated as single isomers resulting from *syn*-difunctionalization of alkynes along with the small amount of hardly separable byproduct **4** arising from the hydrolysis of intermediate **2**. The stereochemical outcome of the reaction was unambiguously confirmed through single-crystal X-ray diffraction of sulfone **10** derived from **3a** (Scheme 3) and 1D-NOESY of **3g** (see the SI for details). The main limitation of the method stems from the compatibility of Grignard reagent with functional groups present in the starting materials. First, we tested various aryl-alkyl-substituted alkynes cleanly giving rise to products bearing the SCF_3_ group α to aryl substituents, which is unprecedented and complementary to carbotrifluoromethylthiolation through the Friedel–Crafts strategy.^18,19^ Variation of the alkyl substituent on the phenylacetylene moiety is well tolerated, enabling the formation of products substituted with methyl (**3b**), linear (**3a**, **3e**), and branched (**3c**, **3d**) alkyl groups. Highly sterically crowded alkynes, such as *t*-butylphenylacetylene and trimethylsilylphenylacetylene, proved to be problematic reaction partners due to incomplete carbomagnesiation and poor regioselectivity. In contrast, benzyl-substituted phenylacetylene performed similarly to model phenylhexyne (cf. **3f** and **3a**). Importantly, alkyl chlorides are well tolerated under the reaction conditions (**3e**), providing a versatile handle for further functionalities of the product (*vide infra*). Further, the methodology proved to be fully compatible with a variety of arylhexynes (**3g**–**3o**). Typically, electron rich substrates provided products with higher yields (**3g**) compared to electron poor alkynes (**3h**–**3k**). Sterically demanding 1-naphthylhexyne is well tolerated, delivering product **3h** in a decent 58% yield. Phenylhexynes bearing halogens (**3i**), sulfonates (**3j**, **3k**), protected carbonyl (**3l**), or phenol functions (**3m**) at the aryl ring are all compatible with the reaction conditions, opening the possibilities for further synthetic transformations.

**Scheme 2:**
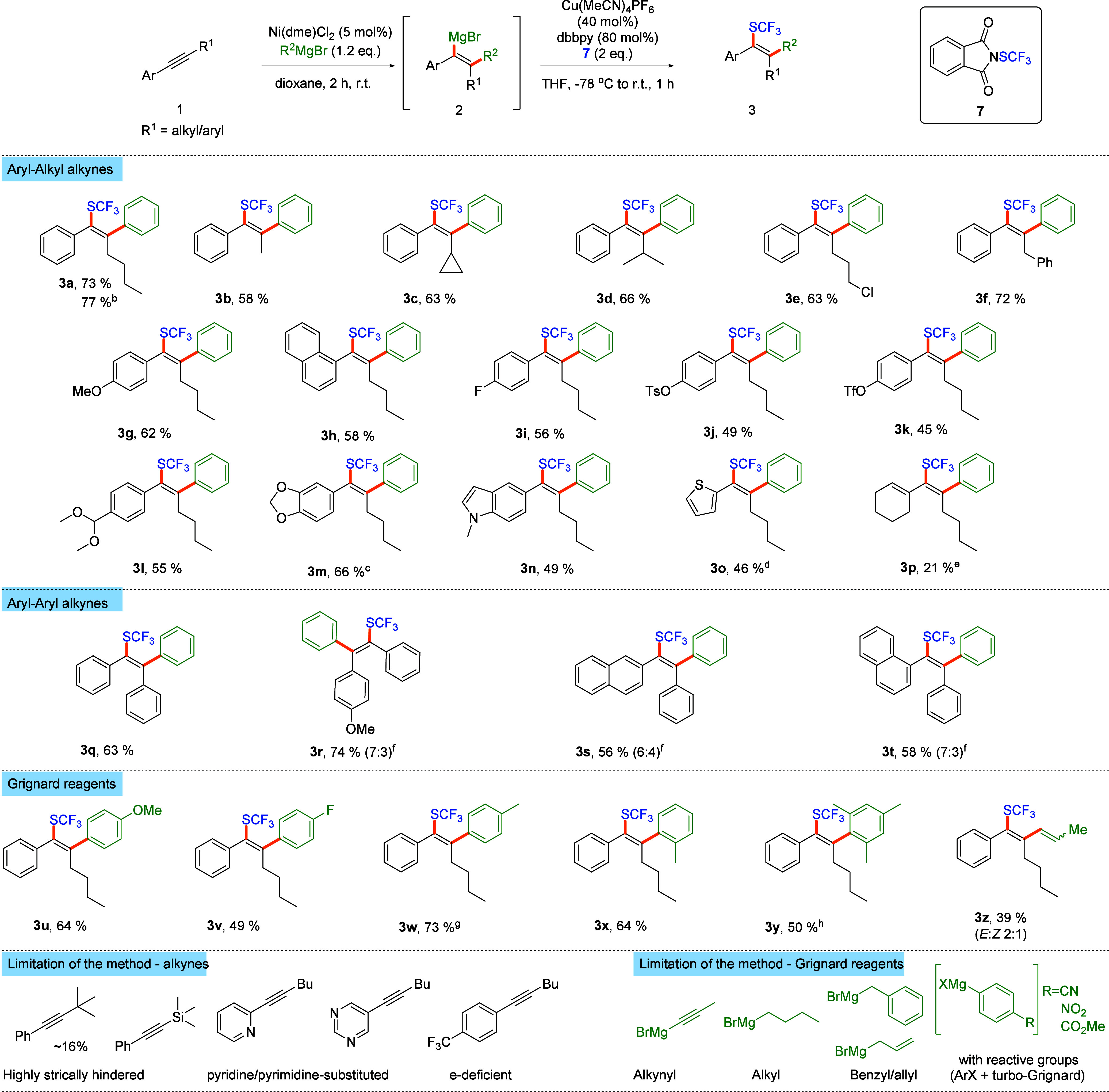
Scope of Carbo-Trifluoromethylthiolation of Alkynes Reaction conditions: Ni(dme)Cl_2_ (5 mol %), **1** (0.2 mmol), R^2^ MgBr (1.2 equiv), dioxane (0.8 mL), 2 h, r.t., then Cu(MeCN)_4_PF_6_ (0.4 equiv), *t*-Bu Bpy (0.8 equiv), *N*-(trifluoromethylthio)phthalimide (2 equiv), THF (0.6 mL), 1 h, −78 °C to r.t. Isolated yields reported; typically products **3** were isolated along with small amounts of olefins **4** (inseparable). Run at 1 mmol scale. Carbomagnesiation run for 3 h. Carbomagnesiation at 60 °C. Carbomagnesiation at 60 °C for 4 h. Mixture of regioisomers, major isomer drawn. Carbomagnesiation run for 4 h. Carbomagnesiation at 75 °C.

**Scheme 3:**
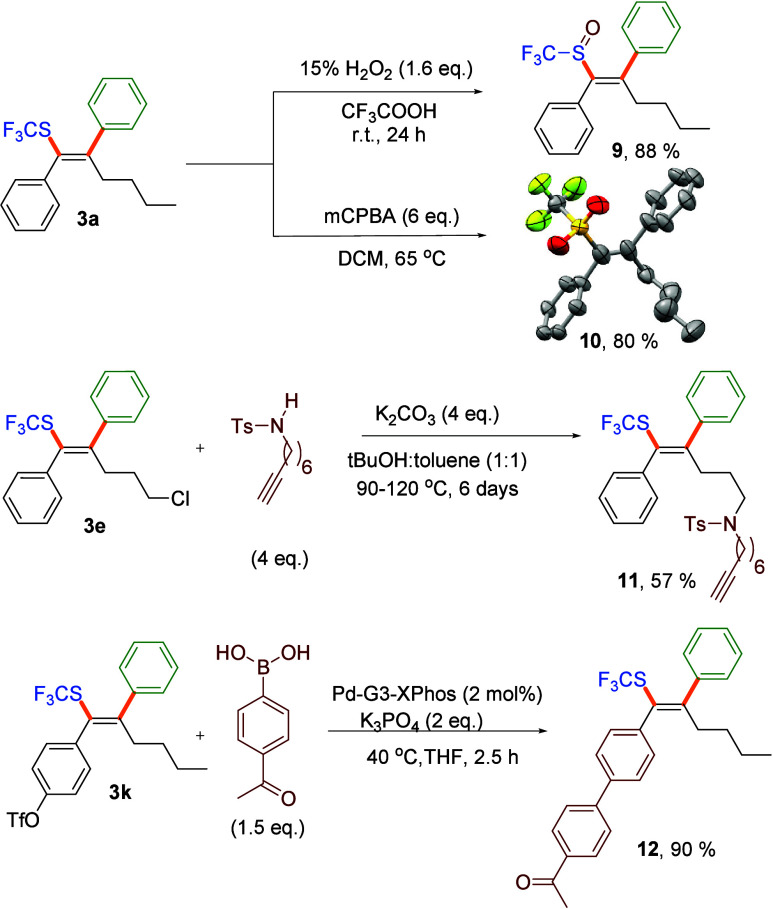
Synthetic Utility of Products **3** ORTEP drawing of **10** with thermal ellipsoids shown at 30% probability.

Alkynes, bearing heterocyclic moieties like benzodioxole (**3m**), indole (**3n**), or thiophene (**3o**), are competent reaction partners. Enynes are more difficult partners, providing the desired highly substituted dienes (**3p**) in excellent regio- and stereoselectivity, albeit in low yield. The method was well accommodated to diaryl-substituted acetylenes (**3q**–**3t**); however, unsymmetrically substituted substrates suffered from low regioselectivity (**3r**–**3t**). Dialkyl-substituted alkynes are incompatible reaction partners, due to sluggish carbomagnesation.^20^

Finally, the scope with respect to Grignard reagents in reaction with model phenylhexyne **1a** was investigated (**3u**–**3z**). Electron-donating (**3u**, **3w**, and **3x**) and moderately electron-withdrawing (**3v**) groups are well tolerated in the structure of phenylmagnesium bromide. Sterically crowded *o*-substituted substrates are also competent partners (**3x**, **3y**); however, the yields of the isolated products are slightly lower compared to those of *p*-substituted analogues (cf. **3x** and **3w**). Propenyl magnesium bromide also underwent the transformation (**3z**), albeit with moderate yield. Aliphatic, benzylic, allylic, and alkynylic Grignard reagents did not undergo Ni-catalyzed carbomagnesation.^20^ Similarly, reagents bearing reactive functional groups (CN, NO_2_, and CO_2_Me) in situ generated from corresponding halides with turbo-Grignard were also incompatible partners.

Further transformations of the products obtained demonstrate the wider utility of the disclosed carbotrifluoromethylthiolation methodology (Scheme 3). Thus, the trifluoromethylthio group in **3a** could be selectively oxidized with high yields to either the corresponding sulfoxide **9** or sulfone **10**. The structure of **10** was unambiguously confirmed by single crystal X-ray diffraction. The chloro-substituted product **3e** allows the generation of the desired product with higher structural complexity through S_N_2 reactions, which was demonstrated through the synthesis of acetylenic sulfonamide **11**. Triflate **3k** could be considered a versatile intermediate that allows functionalization through cross coupling chemistry. For instance, Suzuki coupling performed under mild conditions with 4-acetyl phenyl boronic acid delivered unprotected ketone **12** in a high yield.

A plausible mechanistic picture, supported by our observations and literature data,^21^ is depicted in Scheme 4. First, the reduction of Ni^II^ to Ni^0^ takes place with the aid of Grignard reagent. The resulting Ni^0^ species **I** is capable of coordination to the alkyne (a π-accepting ligand) with a strong component of backdonation from the electron-rich metal center^22^ and can react further with Grignard reagent,^23^ leading to nicklate complex **II**. Then, insertion to the C–Ni bond delivers **III**, which finally liberates vinyl-magnesium compound **2**, a product of arylmagnesiation of alkyne. In the second step, electrophilic trifluoromethylthiolating agent (e.g., **7**) reacts with Cu^I^ to form Cu^III-^SCF_3_ species **IV**,^24^ which upon transmetalation with vinyl-magnesium intermediate **2** delivers **V**. Ultimately, reductive elimination gives rise to the final product **3**, thus regenerating Cu^I^ and completing the catalytic cycle.

**Scheme 4:**
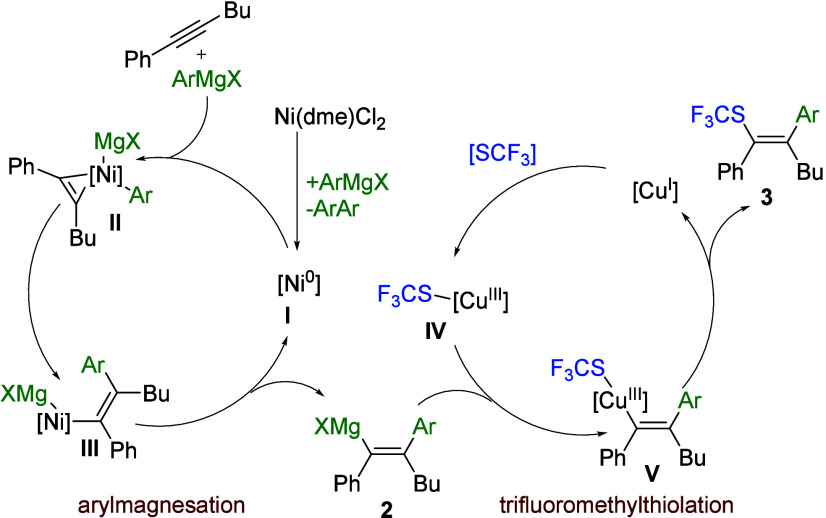
Plausible Mechanism

In conclustion, we developed a convenient protocol for *syn*-selective carbotrifluoromethylthiolation of internal alkynes. Along stereoselectivity, unprecedented for trifluoromethylthiolation-functionalization of alkynes, the method features a unique opportunity for α-selective installation of SCF_3_. Mechanistically, the transformations exploit a sequence of Ni-catalyzed carbomagnesiation, which controls regio- and stereoselectivity, followed by Cu-mediated introduction of SCF_3_ with the preservation of the configuration at the alkene backbone.

## Data Availability

The data underlying this study are available in the published article and its Supporting Information.

## Supporting Information Available

Evaluation of reaction conditions, experimental procedures, analytical data and copies of ^1^H, ^19^F, and ^13^C NMR spectra of new compounds, and crystallographic data (PDF)
