# Supplementary material for: α-Selective syn-Carbotrifluoromethylthiolation of Alkynes
Source: Org Lett. 2025 Mar 3;27(10):2498–503. doi: 10.1021/acs.orglett.5c00570 (PMC11915486; doi:10.1021/acs.orglett.5c00570)
Supplement: Supplementary file 1 — ol5c00570_si_001.pdf [file ol5c00570_si_001.pdf]

Supporting information

α-Selective *syn*-Carbotrifluoromethylthiolation of Alkynes

Prachi Shah, Wojciech Chaładaj*

Institute of Organic Chemistry, Polish Academy of Sciences, Kasprzaka 44/52, 01-224 Warsaw, Poland
**wojciech.chaladaj@icho.edu.pl*

Table of contents

Table of contents 1
General Information 2
Materials 2
Evaluation of reaction conditions for Ni-catalysed carbomagnesiation of internal alkynes 3
Evaluation of reaction conditions for one pot procedure of carbotrifluoromethylthiolation of alkynes 5
Configuration Assignment for compound 3g (1D-NOESY) 10
Synthesis of alkynes (starting materials) 11
General procedure for carbotrifluoromethylthiolation of alkynes. 13
Further transformations of the selected SCF_3-substituted olefins. 21
Copies of ^{1}H, ^{19}F and $^{13}C\{^{1}H\}$ NMR spectra of isolated compounds 23
X-Ray Crystallographic Data for compound 10 87
References 95

General Information

All the manipulations were performed in a nitrogen-filled glovebox or under an argon atmosphere using Schlenk techniques, unless mentioned otherwise. Flash chromatography was performed using Merck silica gel 60 (230-400 mesh). TLC analysis of reaction mixtures was performed on Merck silica gel 60 F254 TLC plates and visualized with cerium molybdate stain (Hanessian's stain) and potassium permanganate stain. ^{1}H, $^{13}C\{1H\}$, and ^{19}F NMR spectra were recorded with a Bruker AV 400 spectrometer. ^{1}H and ^{13}C chemical shifts are given in ppm relative to TMS. The solvent signals were used as references ($CDCl_3$ δ_H = 7.26 ppm, δ_C = 77.0 ppm) and the chemical shift converted to the TMS scale. Coupling constants (*J*) are reported in Hz, and the following abbreviations were used to denote multiplets: s = singlet, d = doublet, t = triplet, q = quartet, quint = quintet, m = multiplet (denotes complex pattern), dd = doublet of doublets, dt = doublet of triplets and br = broad signal. Infrared spectra were recorded with a Jasco FTIR-6200 spectometer. Electron ionization high-resolution mass spectra (EI-HR) were recorded with an Autospec Premier (Waters Inc) mass spectrometer spectrometer equipped with an electron impact (EI) ion source and the EBE double focusing geometry mass analyzer, using the narrow-range high-voltage scan technique with low-boiling perfluorokerosene (PFK) as internal standard. The instrument was controlled and recorded data were processed using MassLynx 4.1 software package (Waters Inc). Samples were introduced by using a heated direct insertion probe. Atmospheric pressure chemical ionization (APCI-TOF) was recorded on Synapt G2-S HDMS (Waters Inc) mass spectrometer equipped with an APCI ion source and q-TOF type mass analyzer. The instrument was controlled and recorded data were processed using MassLynx V4.1 software package (Waters Inc).

Materials

Unless otherwise noted, all commercially available compounds (ABCR, Acros, Angene, Ambeed, Fluorochem, TCI, Sigma-Aldrich, Strem) were used as received. Aryl iodides[1], Alkynes[2–15], PhMgBr[16], Buchwald-type 3rd-generation palladacyclic precatalyst (Ligand Pd G3)[17] and thiotrifluoromethylating agent[18] were prepared following literature procedure.

Evaluation of reaction conditions for Ni-catalysed carbomagnesiation of internal alkynes

General procedure for evaluation of reaction conditions: Under argon, in a dark vial, $NiCl_2$(dme) (10.99 mg, 1 mmol, 5 mol%) was added along with a magnetic stirring bar. To the catalyst, alkyne (1 mmol) and dioxane (4 mL) were added. The resultant solution was stirred for 5 minutes. Then phenyl magnesium bromide (0.24 mL, 0.24 mmol, 1.0 M in THF, 1.2 eq) was added dropwise. The reaction mixture was stirred at r.t. for 2 h. The mixture was diluted with MTBE (1mL) quenched with sat. aqueous NH_4Cl (1 mL) and mesitylene (30 µl) was added as an internal standard.

Table S1. Effect of catalyst[a]

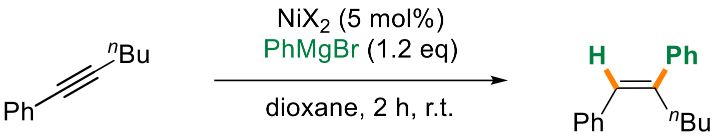

Entry	NiX_2	Conversion (%)	Yield (%)
1	**Ni(dme)Cl_2**	**95**	**93**
2	Ni(dme)Br_2	95	80
3	$NiCl_2$	95	80
4	$NiBr_2$	95	80
5	$Ni(acac)_2$	100	61
6	$Ni(OTf)_2$	25	21
7	$NiCl_2.6H_20$	70	58
8	$Fe(acac)_3$	0	0
9	Co(dme)Br_2	14	0
10	CuI	0	0

[a]Conditions: NiX_2 (5 mol%), phenylhexyne (1 mmol), PhMgBr (1.2 eq), dioxane (4 mL), 2 h, r.t.; yield and conversion determined by GC using mesitylene as an internal standard.

Table S2. Effect of mol% of the catalyst[a]

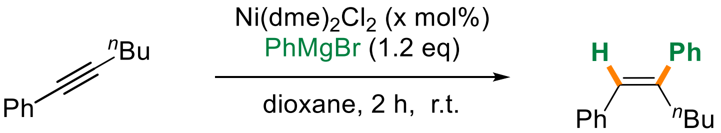

Entry	Mol% of catalyst	Conversion (%)	Yield (%)
1	2mol%	85	80
2	**5 mol%**	**95**	**93**
3	10 mol%	100	93
4	15 mol%	100	94
5	20 mol%	100	94

[a]Conditions: Ni(dme)Cl_2 (x mol%), phenylhexyne (1 mmol), PhMgBr (1.2 eq), dioxane (4 mL), 2 h, r.t.; yield and conversion determined by GC using mesitylene as an internal standard.

Table S3. Effect of ligands[a]

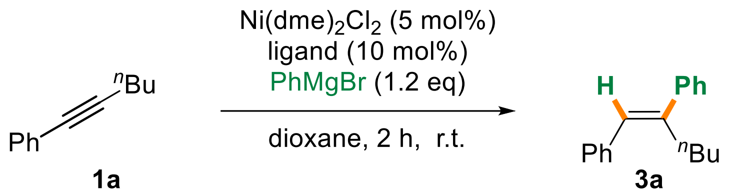

Entry	Ligand	Conversion (%)	Yield (%)
1	2,2' bpy	100	75
2	1,10 phen	100	65
3	TMEDA	100	80
4	PMDTA	100	73
**5**	**-**	**100**	**82**

[a]Conditions: Ni(dme)Cl_2 (5 mol%), ligand (10 mol%), phenylhexyne (1 mmol), PhMgBr (1.2 eq), solvent (4mL), 2 h, r.t.; Isolated yields reported.

Table S4. Equivalents of PhMgBr[a]

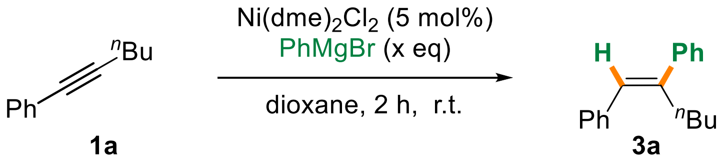

Entry	Eq of PhMgBr	Conversion (%)	Yield (%)
1	1.0	100	70
2	**1.2**	**100**	**93**
3	1.4	100	92
4	1.6	100	93

[a]Conditions: Ni(dme)Cl_2 (5 mol%), phenylhexyne (1mmol), PhMgBr (y eq), dioxane (4 mL), 2 h, r.t.; yield and conversion determined by GC using mesitylene as an internal standard.

Table S5. Effect of solvent[a]

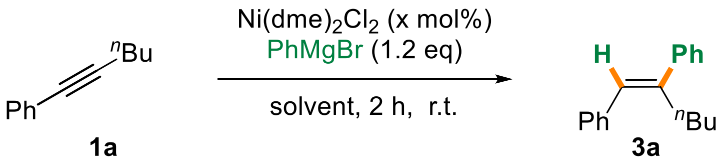

Entry	Solvent	Conversion (%)	Yield (%)
1	**Dioxane**	**100**	**93**
2	THF	80	41
3	Toluene	100	62
4	ACN	30	20
5	HMPA	18	0
6	DMPU	33	0
7	NMP	77	0

[a]Conditions: Ni(dme)Cl_2 (5 mol%), phenylhexyne (1 mmol), PhMgBr (1.2 eq), Solvent (4 mL), 2 h, r.t.; yield and conversion determined by GC using mesitylene as an internal standard.

Evaluation of reaction conditions for one pot procedure of carbotrifluoromethylthiolation of alkynes

General procedure for evaluation of reaction conditions. Vinylmagnesium intermediate **2a** was prepared through Ni-catalyzed carbomagnesation of phenylhexyne: In the glove box, $NiCl_2$(dme) (5 mol%, 2.2 mg, 0.01 mmol) was introduced into a 4mL vial equipped with a magnetic stir bar. To the catalyst were added phenylhexyne (31.7 mg, 0.2 mmol), mesitylene (30 µl), dioxane (0.8 mL) and then phenyl magnesium bromide solution (0.24 mL, 0.24 mmol, 1.0 M in THF) dropwise. The resulting mixture was stirred at room temperature for 2 h. To a 15-mL flame dried shlenk were added, $Cu(MeCN)_4PF_6$ (40 mol%, 29.9 mg, 0.08 mmol), 4,4' *tert*-butyl bipyridine (80 mol%, 43 mg, 0.16 mmol), N-Methyl-N-[(trifluoromethyl)thio]-p-toluenesulfonamide **6** (114.1 mg, 0.4 mmol, 2 eq) and 0.6 mL of THF. The solution was cooled to -78 °C and the freshly prepared solution of vinylmagnesium intermediate **2a** was added to it dropwise. The reaction mixture was removed from the cooling bath and allowed to stir at r.t. for 18 h. The mixture was quenched with sat. aq. NH_4Cl (1 mL) and diluted with MTBE (1 mL).

Table S6. Effect of ligand

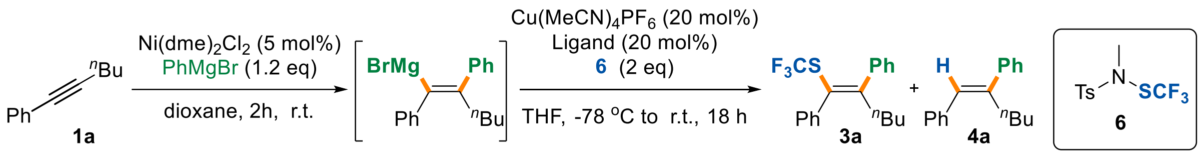

Entry	Ligand	Yield of 3a (%)	Yield of 4a (%)
1	2,2'-bipyridine	23	10
2	4,4'- dimethoxy 2,2'-bipyridine	27	11
3	4,4'-dimethyl 2,2'-bipyridine	27	10
4	5,5'-dimethyl 2,2'-bipyridine	29	9
5	6,6'-dimethyl 2,2'-bipyridine	10	11
6	**4,4'-di-tert-butyl 2,2'-bipyridine**	**42**	**10**
7	4,4′,4″-tri-tert-Butyl-2,2′:6′,2″-terpyridine	20	15
8	dipicolyl amine	15	30
9	1,10- phenanthroline	25	9
10	**3,4,7,8-tetra methyl 1,10-phenanthroline**	**45**	**13**
11	BINAP	23	17
12	Xant Phos	22	20
13	Dppb	4	42
14	dppy	14	17
15	X phos	13	12
16	PMDTA	6	13

[a]Conditions: $NiCl_2$(dme) (5 mol%), phenylhexyne (0.2 mmol, 1eq), PhMgBr (1.2 eq), dioxane (0.8 mL), 2 h, r.t. then $Cu(MeCN)_4PF_6$ (20 mol%), Ligand (20 mol%), N-Methyl-N-[(trifluoromethyl)thio]-p-toluenesulfonamide **6** (2 eq) in 0.6mL THF (0.67M), -78°C to r.t., 18 h; yield determined by GC using mesitylene as an internal standard.

Table S7. Ratio of $Cu(MeCN)_4PF_6$ to ligand[a]

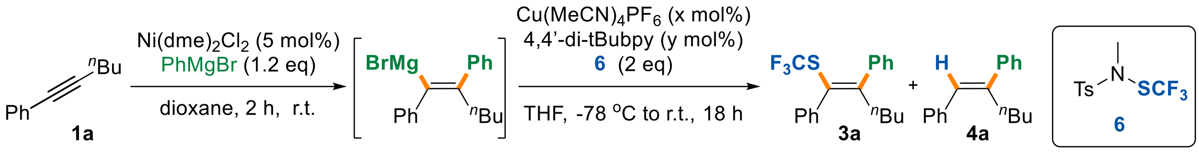

Entry	Cu (mol%) : L (mol%)	Yield of 3a (%)	Yield of 4a (%)
1	5:5 (1:1)	19	8
2	10:10	22	9
3	20:20	39	9
4	40:40	48	11
5	5:10 (1:2)	26	8
6	10:20	44	7
7	20:40	56	8
8	**40:80**	**59**	**8**
9	40:120 (1:3)	64	9
10[b]	20:40 (1:2)	44	15
11[b]	40:80	58	16
12[b]	40:120 (1:3)	65	13

[a]Conditions: $NiCl_2$(dme) (5 mol%), phenylhexyne (0.2 mmol, 1eq), PhMgBr (1.2 eq), dioxane (0.8 mL), 2 h, r.t. then $Cu(MeCN)_4PF_6$ (x mol%), 4,4'-di-tert-butyl 2,2'-bipyridine (y mol%), N-Methyl-N-[(trifluoromethyl)thio]-p-toluenesulfonamide **6** (2 eq) in 0.6 mL THF (0.67 M), -78 °C to r.t., 18 h; yield determined by GC using mesitylene as an internal standard. [b]1,10 phenanthroline was used as a ligand in trifluoromethylthiolation step.

Table S8. Ratio of $CuBr.Me_2S$ to ligand.[a]

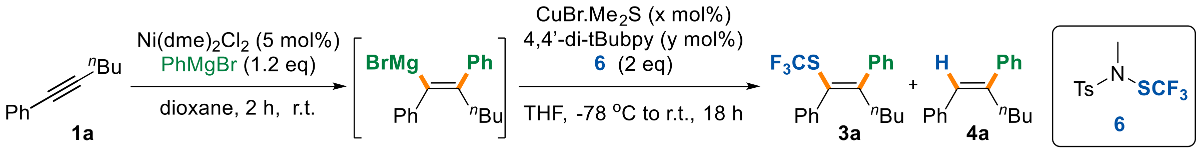

Entry	Cu (mol%) : L (mol%)	Yield of 3a (%)	Yield of 4a (%)
1	40:40 (1:1)	41	7
2	10:20 (1:2)	35	7
3	20:40	50	7
4	**40:80**	**56**	**7**
5	100:200	56	6
6[b]	100, no ligand	18[c]	44
7[b]	20:40 (1:2)	49[d]	30
8[b]	40:80	45[d]	35

[a]Conditions: $NiCl_2$(dme) (5 mol%), phenylhexyne (0.2 mmol, 1eq), PhMgBr (1.2 eq), dioxane (0.8 mL), 2 h, r.t. then $CuBr.Me_2S$ (x mol%), 4,4'-di-tert-butyl 2,2'-bipyridine (y mol%), N-Methyl-N-[(trifluoromethyl)thio]-p-toluenesulfonamide **6** (2 eq) in 0.6 mL THF (0.67 M), -78 °C to r.t., 18 h; yield determined by GC using mesitylene as an internal standard. [b]1,10 phenanthroline was used as a ligand in trifluoromethylthiolation step.

Table S9. Effect of the copper source.[a]

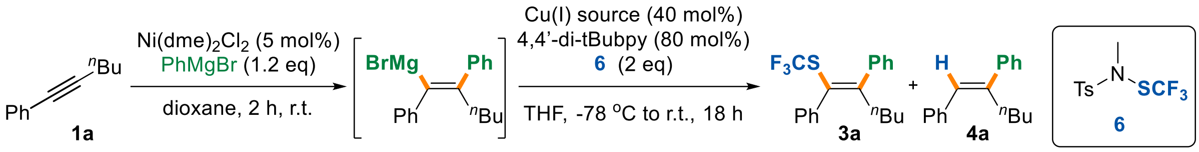

Entry	Cu(I) source	Yield of 3a (%)	Yield of 4a (%)
1	**$Cu(MeCN)_4PF_6$**	**59**	**10**
2	CuI	40	9
3	**CuSCN**	**61**	**8**
4	CuCN	54	8
5	CuCl	54	7
6	**$CuBr.Me_2S$**	**62**	**6**
7	$CuCF_3(PPh_3)_3$	33	15

[a]Conditions: $NiCl_2$(dme) (5 mol%), phenylhexyne (0.2 mmol, 1eq), PhMgBr (1.2 eq), dioxane (0.8 mL), 2 h, r.t. then Cu(I) source (40 mol%), 4,4'-di-tert-butyl 2,2'-bipyridine (80 mol%), N-Methyl-N-[(trifluoromethyl)thio]-p-toluenesulfonamide **6** (2 eq) in 0.6mL THF (0.67 M), -78 °C to r.t., 18 h; yield determined by GC using mesitylene as an internal standard.

Table S10. Effect of solvent.[a]

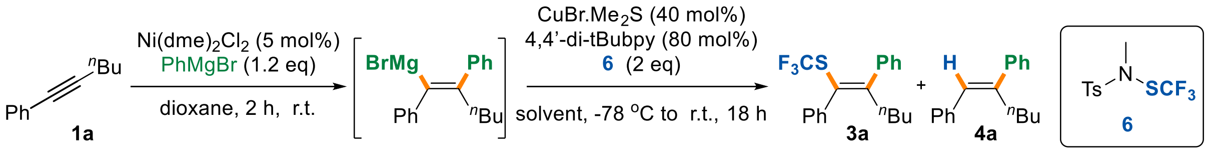

Entry	Solvent (mL)	Yield of 3a (%)	Yield of 4a (%)
1	**THF**	**59**	**5**
2	Dioxane	42	5
3	ACN	55	7
4	Toluene	52	5
5	NMP	19	19

[a]Conditions: $NiCl_2$(dme) (5 mol%), phenylhexyne (0.2 mmol, 1eq), PhMgBr (1.2 eq), dioxane (0.8 mL), 2 h, r.t. then $CuBr.Me_2S$ (40 mol%), 4,4'-di-tert-butyl 2,2'-bipyridine (80 mol%), N-Methyl-N-[(trifluoromethyl)thio]-p-toluenesulfonamide **6** (2 eq) in 0.6 mL solvent (0.67 M), -78 °C to r.t., 18 h; yield determined by GC using mesitylene as an internal standard.

Table S11. Volume of solvent[a]

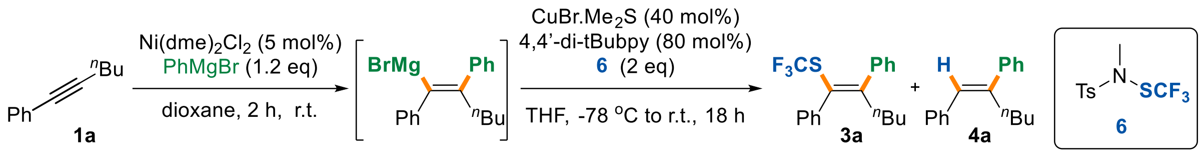

Entry	Volume of THF	Yield of 3a (%)	Yield of 4a (%)
1	**0.6**	**61**	**6**
2	1	61	7
3	2	61	10

[a]Conditions: $NiCl_2$(dme) (5 mol%), phenylhexyne (0.2 mmol, 1eq), PhMgBr (1.2 eq), dioxane (0.8 mL), 2 h, r.t. then CuBr.Me_2S (40 mol%), 4,4'-di-tert-butyl 2,2'-bipyridine (80 mol%), N-Methyl-N-[(trifluoro-methyl)thio]-p-toluenesulfonamide **6** (2 eq) in THF (volume), -78 °C to r.t., 18 h; yield determined by GC using mesitylene as an internal standard.

Table S12. Effect of temperature[a]

Entry	Temperature of thiolation	Yield of 3a (%)	Yield of 4a (%)
1	**-78 °C to r.t.**	**63**	**6**
2	-78 °C to R.t. to 60 °C	57	6
3	-40 °C to r.t.	58	6
4	r.t.	39	6
5	r.t. to 60 °C	42	6
6	-78 °C to -78 °C (1 h) to r.t.	40	8

[a]Conditions: $NiCl_2$(dme) (5 mol%), phenylhexyne (0.2 mmol, 1eq), PhMgBr (1.2 eq), dioxane (0.8 mL), 2 h, r.t. then CuBr.Me_2S (40 mol%), 4,4'-di-tert-butyl 2,2'-bipyridine (80 mol%), N-Methyl-N-[(trifluoro-methyl)thio]-p-toluenesulfonamide **6** (2 eq) in 0.6 mL THF, temperature, 18 h; yield determined by GC using mesitylene as an internal standard.

Table S13. Equivalents of SCF_3-agent **6**.[a]

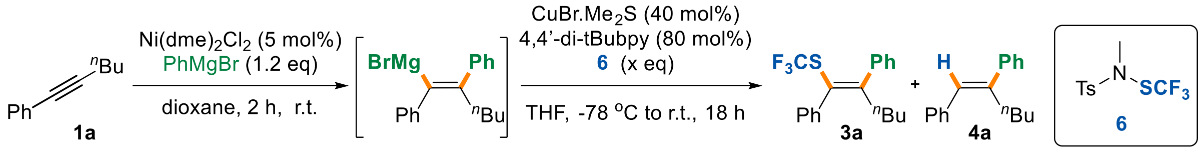

Entry	Amount of 6 (eq)	Yield of 3a (%)	Yield of 4a (%)
1	1.1	33	7
2	1.5	45	6
3	**2**	**52**	**6**
4	2.5	53	6

[a]Conditions: $NiCl_2$(dme) (5 mol%), phenylhexyne (0.2 mmol, 1eq), PhMgBr (1.2 eq), dioxane (0.8 mL), 2 h, r.t. then CuBr.Me_2S (40 mol%), 4,4'-di-tert-butyl 2,2'-bipyridine (80 mol%), N-Methyl-N-[(trifluoro-methyl)thio]-p-toluenesulfonamide **6** (x eq) in THF (0.6 mL), -78 °C to r.t., 18 h; yield determined by GC using mesitylene as an internal standard.

Table S14. Order of addition[a]

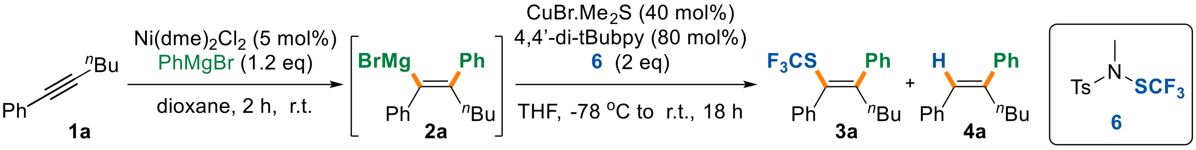

C: Vinylmagnesium species **2a** prepared by Ni-catalyzed carbomagnesiation of phenylhexyne

SCF₃: Solution of Cu(I), ligand and SCF₃-agent **6** in THF

Cu + L: Mixture of copper and ligand (as solid)

Entry	Order of addition[a]	Yield of 3a (%)	Yield of 4a (%)
1	**C to SCF$_3$**	**58**	**10**
2	SCF$_3$ to C	45	13
3	**C to Cu + L then 6**	58	10
4	Cu + L to C, then **6**	57	7
5	Cu + L to C, then **6**	33[b]	8[b]

[a]Conditions: $NiCl_2$(dme) (5 mol%), phenylhexyne (0.2 mmol, 1eq), PhMgBr (1.2 eq), dioxane (0.8 mL), 2 h, r.t. then CuBr.Me_2S (40 mol%), 4,4'-di-tert-butyl 2,2'-bipyridine (80 mol%), N-Methyl-N-[(trifluoro-methyl)thio]-p-toluenesulfonamide **6** (2 eq) in THF (0.6 mL), -78 °C to r.t., 18 h; yield determined by GC using mesitylene as an internal standard. [b]Cu(MeCN)$_4$PF$_6$ used as a Cu source.

Table S15. Order of addition (isolated yield)[a]

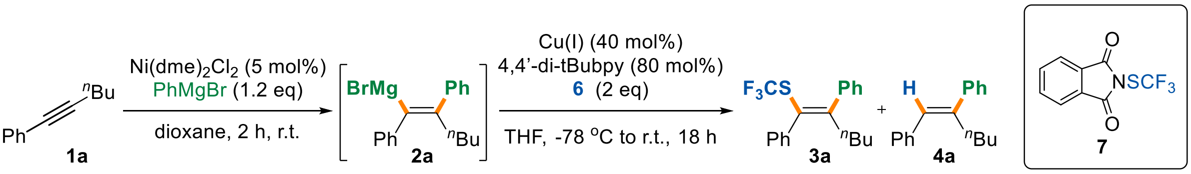

C.: Vinylmagnesium species **2a** prepared by Ni-catalyzed carbomagnesation of phenylhexyne

SCF₃: Solution of Cu(I), ligand and SCF₃-agent **6** in THF

Cu + L: Mixture of copper and ligand (as solid)

Entry	Order of addition	Cu(I)	Yield of 3a (%)	Yield of 4a (%)
1	C to SCF$_3$	Cu(MeCN)$_4$PF$_6$	60	6
2	C to SCF$_3$	CuBr.Me_2S	59[c]	6
3	Cu + L to C then **6**	Cu(MeCN)$_4$PF$_6$	73	8
4	Cu + L to C then **6**	CuBr.Me_2S	55[c]	8

[a]Conditions: $NiCl_2$(dme) (5 mol%), phenylhexyne (0.2 mmol, 1eq), PhMgBr (1.2 eq), dioxane (0.8 mL), 2 h, r.t. then CuBr.Me_2S (40 mol%), 4,4'-di-tert-butyl 2,2'-bipyridine (80 mol%), N-Methyl-N-[(trifluoro-methyl)thio]-p-toluenesulfonamide **6** (2 eq) in THF (0.6 mL), -78 °C to r.t., 18 h; isolated yields reported.

Configuration Assignment for compound 3g (1D-NOESY)

In 1D-NOESY spectrum of **3g** a through space interactions between allylic CH_2 (2.35-39 ppm) with both aryl rings were observed, confirming a proposed geometry at the vinyl system.

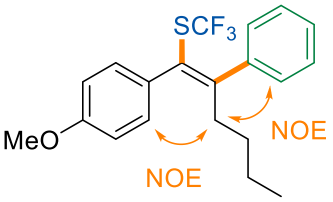

3g

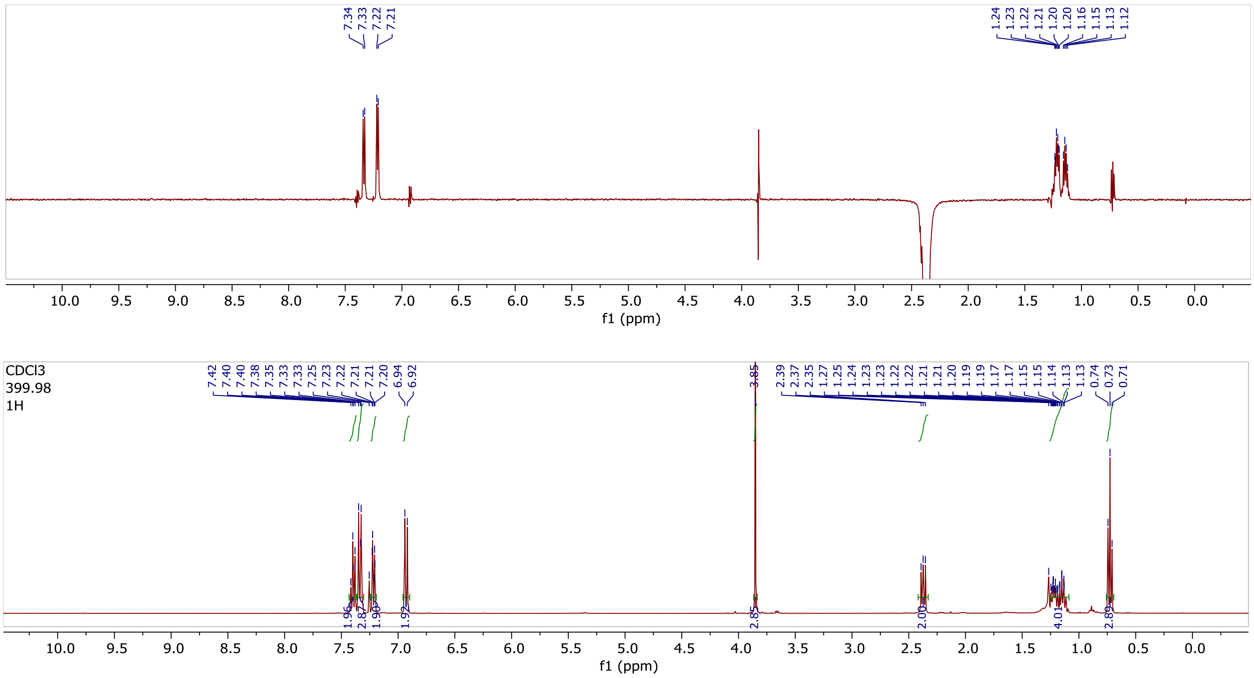

1D-NOESY (upper) and ^{1}H NMR (lower) of **3g**.

Synthesis of alkynes (starting materials)

1-(dimethoxymethyl)-4-(hex-1-yn-1-yl)benzene. A flame dried round bottom flask was evacuated and backfilled with argon (the cycle was performed twice) and then charged under a positive pressure of argon with XPhos Pd G3 (8.5 mg, 0.010 mmol, 1 mol%), XPhos (14.3 mg, 0.030 mmol, 3 mol%), Cs_2CO_3 (847.2 mg, 2.678 mmol, 2.6 eq), MeCN (2 mL), 1-bromo-4-(dimethoxymethyl) benzene (238.0 mg, 172.0 µl, 1.030 mmol). The reaction mixture was stirred for 25min at r.t. Then 1-hexyne (106.8 mg, 150.5 µl, 1.339 mmol, 1.3 eq) was added and the resultant was heated to 75 °C for 5 h. The resulting suspension was cooled to room temperature, diluted with water, and extracted with MTBE. The combined organic layers were dried over Na_2SO_4, concentrated providing red oil as a crude product (239.3 mg, 1.030 mmol, 100%), which was used without further purification. ^{1}H NMR (400 MHz, $CDCl_3$): 7.38 (m, 4H), 5.37 (s, 1H), 3.30 (s, 6H) 2.40 (t, J = 7.0 Hz, 2H), 1.5 (m, 4H), 0.95 (t, J = 7.3 Hz, 3H); ^{13}C NMR (400 MHz, $CDCl_3$): 137.2, 131.3, 128.5, 126.5, 124.2, 102.6, 102.3, 90.7, 80.3, 52.4, 30.8, 21.9, 19.0, 13.5; HRMS (EI-EBE) m/z: $[M]^+$ Calc'd for $C_{15}H_{20}O_2$: 232.1463, Found: 232.1467.

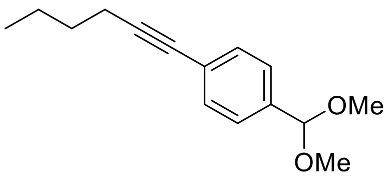

5-(hex-1-yn-1-yl)-1-methyl-1H-indole. A flame dried round bottom flask was evacuated and backfilled with argon (the cycle was performed twice) and then charged under a positive pressure of argon with XPhos Pd G3 (72.2 mg, 0.085 mmol, 1 mol%), XPhos (121.8 mg, 0.255 mmol, 3 mol%), Cs_2CO_3 (7.2 g, 22.157 mmol, 2.6 eq), MeCN (17 mL), 5-bromo-1-methyl-1H-indole (1.8 g, 8.522 mmol). The reaction mixture was stirred for 25 min at r.t. Then 1-hexyne (910.1 mg, 1.3 mL, 11.078 mmol, 1.3 eq) was added and the resultant was heated to 75 °C for 48 h. The resulting suspension was cooled to room temperature, diluted with water, and extracted with MTBE. The combined organic layers were dried over Na_2SO_4, concentrated, and the residue was purified by flash chromatography on silica gel (60/90 petroleum ether/ethyl acetate 99.5:0.5 to 95:10) to provide the desired product as an orange oil (1.4 g, 6.625 mmol, 78%). Analytical data was in agreement with literature data.[19]

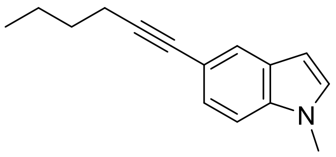

4-(hex-1-yn-1-yl)phenyl tosylate. A flame dried round bottom flask was evacuated and backfilled with argon (the cycle was performed twice) and then charged under a positive pressure of argon with 4-iodophenyl 4-methylbenzenesulfonate (2.0 g, 5.344 mmol), $Pd(PPh_3)_2Cl_2$ (18.8 mg, 0.267 mmol, 5 mol%), CuI (101.8 mg, 0.534 mmol, 10 mol%), Et_3N (1.1 g, 1.5 mL, 10.689 mmol, 2eq), THF (22 mL), 1-hexyne (658.6 mg, 0.93 mL, 8.017 mmol, 1.5 eq). The resultant reaction mixture was stirred for 24 h at r.t. The reaction mixture was diluted with water and extracted with MTBE. The combined organic layers were dried over Na_2SO_4, concentrated, and the residue was purified by flash chromatography on silica gel (60/90 petroleum ether/ethyl acetate 95:5 to 90:10) to provide the desired product as thick reddish-orange oil (1.7 g, 5.176 mmol, 97%). Analytical data was in agreement with literature data.[20]

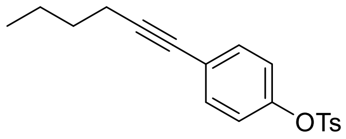

4-(hex-1-yn-1-yl)phenol. A flame dried round bottom flask was evacuated and backfilled with argon (the cycle was performed twice) and then charged under a positive pressure of argon with 4-iodophenol (1.9 g, 9.090 mmol), $Pd(PPh_3)_2Cl_2$ (127.7 mg, 0.181 mmol, 2 mol%), CuI (34.7 mg, 0.181 mmol, 2 mol%), dioxane (9 mL), Et_3N (6.3 mL, 4.6 g, 45.452 mmol, 5 eq), 1-hexyne (1.27 mL, 10.908 mmol, 1.2 eq). The resultant was subjected on a pre-heated bath (55 °C) for 30min. The resultant was cooled to room temperature diluted with water and extracted with MTBE. The combined organic layers were dried over Na_2SO_4, concentrated, and the residue was purified by flash chromatography on silica gel (60/90 petroleum ether/ethyl acetate 90:10 to 75:25) to provide the desired product as red oil (1.5 g, 8.608 mmol, 95%). Analytical data was in agreement with literature data.[21]

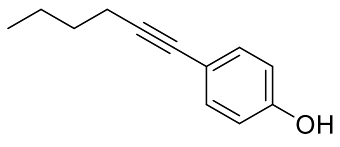

4-(hex-1-yn-1-yl)phenyl triflate. A flame dried round bottom flask was evacuated and backfilled with argon (the cycle was performed twice) and then charged under a positive pressure of argon with 4-(hex-1-yn-1-yl)phenol (1.4 g, 8.215 mmol), CH_2Cl_2 (8 mL), and pyridine (1.32 mL, 1.3 g, 16.431 mmol, 2 eq). The resultant was cooled to 0 °C. Triflic anhydride (2.8 g, 1.62 mL, 9.858 mmol, 1.2 eq) was added dropwise at 0 °C and the reaction mixture was stirred at r.t. for 1.5 h and quenched with the addition of Et_2O (15 mL) and aqueous HCl (10%, 5 mL). The reaction mixture was washed with aqueous saturated $NaHCO_3$ (10 mL) and brine (10 mL). The combined organic layers were dried over Na_2SO_4, concentrated, and the residue was purified by flash chromatography on silica gel (60/90 Petroleum ether/diethylether 100% to 98:2) to provide the desired product as light-yellow oil (2.2 g, 7.182 mmol, 88%). ^{1}H NMR (400 MHz, $CDCl_3$): 7.45 (dd, 2H), 7.18 (dd, 2H), 2.40 (t, J = 7.0 Hz, 2H), 1.53 (m, 4H), 0.95 (t, J = 7.3 Hz, 3H); ^{19}F NMR (400 MHz, $CDCl_3$): -72.89 (s , 3H); ^{13}C NMR (400 MHz, $CDCl_3$): 148.4, 133.2, 124.8, 121.2, 118.7 (q, $^{1}J_{CF}$ = 320.8 Hz), 92.6, 78.8, 30.6, 21.9, 19.0, 13.5; HRMS (EI-EBE) m/z: $[M]^+$ Calc'd for $C_{13}H_{13}F_3O_3S$: 306.0537, Found: 306.0536.

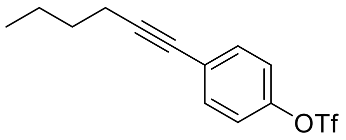

General procedure for carbotrifluoromethylthiolation of alkynes.

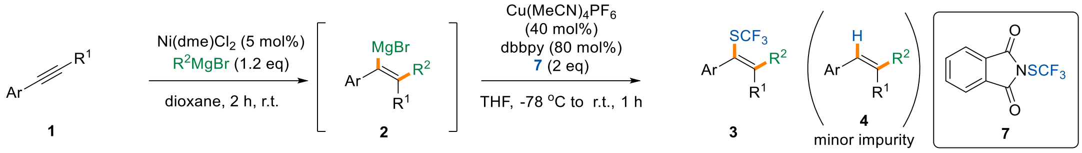

In the glove box after standard cycles of evacuation and back-fill with pure N_2, $NiCl_2$(dme) (2.2 mg, 0.01 mmol, 5 mol%) was introduced into a 15-mL Schlenk tube equipped with a magnetic stir bar. To the catalyst were added alkyne (0.2 mmol), dioxane (0.8 mL) and then aryl magnesium bromide dropwise (0.24 mL, 0.24 mmol, 1.0 M in THF). The resulting mixture was stirred at r.t. for 2 h. After that time the reaction mixture was cooled to -78 ºC and $Cu(MeCN)_4PF_6$ (29.9 mg, 0.08 mmol, 40 mol%), 4,4' *tert*-butyl bipyridine (43 mg, 0.16 mmol, 80 mol%) were added as a solid and N-(trifluoromethylthio)phthalimide **7** (99 mg, 0.4 mmol, 2 eq) as a solution in 0.6 mL THF. The reaction mixture was removed from the cooling bath and allowed to stir for 1 h at r.t.. The mixture was quenched with sat. aqueous NH_4Cl and diluted with MTBE. The combined organic layers were dried over Na_2SO_4, concentrated, and the residue was purified by flash chromatography on silica gel to provide the desired product **3**, typically containing small amount of inseparable trisubstituted olefin **4**.

Procedure for reaction run at 1 mmol scale for compound 3a.

A 30-mL Shlenk tube equipped with a magnetic stirring bar was charged with $NiCl_2$(dme) (10.9 mg, 0.05 mmol, 5 mol%), then evacuated and back-filled with argon (three times). To the catalyst were added 1-phenylhexyne (158.3 mg, 1.0 mmol), dioxane (4 mL) and then phenyl magnesium bromide dropwise (1.2 mL, 1.2 mmol, 1.0 M in THF). The resulting was stirred at r.t. for 2 h. After 2 h, the reaction mixture was cooled to -78 ºC and $Cu(MeCN)_4PF_6$ (149.1 mg, 0.4 mmol, 40 mol%), 4,4' *tert*-butyl bipyridine (214.7 mg, 0.8 mmol, 80 mol%) were added as a solid and N-(trifluoromethylthio)phthalimide **7** (494.3 mg, 2 mmol, 2 eq) as a solution in 3 mL THF. The reaction mixture was removed from the cooling bath and allowed to stir for 1h at r.t.. The mixture was quenched with sat. aqueous NH_4Cl and diluted with MTBE. The combined organic layers were dried over Na_2SO_4, concentrated, and the residue was purified by column chromatography on silica gel. The title compound was isolated as a yellow oil (278.1) after chromatography on silica gel (40 g column, 40/60 petroleum ether) containing **3a** (257.4 mg, 0.765 mmol, 77%) along with small amount of **4a** (20.7 mg, 0.087 mmol, 9%). The compound can be further purified by reverse-phase chromatography (25 g C18-silica column, 7:3 to 9:1 MeOH:H_2O). First run delivered 206.0 mg (0.612 mmol, 61%) of pure **3a** and a fraction containing mixture of **3a** and **4a**. After a second separation of the mixture a total of 234.7 mg of pure **3a** (0.697 mmol, 70%) was isolated.

Analytical data of isolated products of carbotrifluoromethylthiolation of alkynes

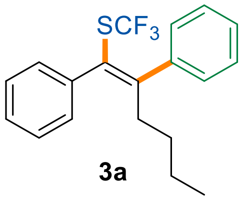

3a

(*Z*)-(1,2-diphenylhex-1-en-1-yl)(trifluoromethyl)sulfane (3a) Prepared by reaction of 1-phenylhexyne (32.0 mg, 0.202 mmol) with phenylmagnesium bromide following general procedure. The title compound was isolated as a yellow oil (53.0 mg) after chromatography on silica gel (15 g column, 40/60 petroleum ether) containing **3a** (49.6 mg, 0.147 mmol, 73%) along with small amount of **4a** (3.4 mg, 0.014 mmol, 7%). Analytical data of **3a**: ^{1}H NMR (400 MHz, $CDCl_3$): 7.42 (m, 6H), 7.36 (m, 2H), 7.25 (m, 2H), 2.39 (t, J = 7.9 Hz, 2H), 1.21 (m, 4H), 0.73 (t, J = 7.2 Hz, 3H); ^{19}F NMR (400 MHz, $CDCl_3$): -40.0 (s, 3F); ^{13}C NMR (400 MHz, $CDCl_3$): 154.4, 141.1, 139.3, 129.3 (q, $^{1}J_{CF}$ = 309.9 Hz), 129.3, 128.2, 128.2, 128.1, 127.8, 127.4, 123.8, 36.4, 30.1, 22.3, 13.6; HRMS (EI-EBE) m/z: $[M]^+$ Calc'd for $C_{19}H_{19}F_3S$: 336.1160, Found: 336.1149; Indicative NMR signals of **4a**: ^{1}H NMR (400 MHz, $CDCl_3$): 7.49 (m, 2H), 6.72 (s, 1H), 2.73 (t, J = 8.1 Hz, 3H), 1.39 (m, 4H), 0.88 (t, J = 7.2 Hz, 3H); ^{13}C NMR (400 MHz, $CDCl_3$): 143.4, 143.2, 138.3, 128.7, 128.3, 128.0, 127.1, 126.6, 126.4, 30.9, 29.9, 22.7, 13.8.

(*Z*)-(1,2-diphenylprop-1-en-1-yl)trifluoromethyl)sulfane (3b) Prepared by reaction of 1-phenyl-propyne

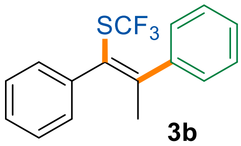

3b

(23.2 mg, 0.1997 mmol) with phenylmagnesium bromide following general procedure. The title compound was isolated as a white solid (36.1 mg) after chromatography on silica gel (15 g column, 40/60 petroleum ether), containing **3b** (34.2 mg, 0.116 mmol, 58 %) along with small amount of **4b** (1.9 mg, 0.009 mmol, 5%). Analytical data of **3b**: ^{1}H NMR (400 MHz, $CDCl_3$): 7.40 (m, 10H), 2.07 (s, 3H); ^{19}F NMR (400 MHz, $CDCl_3$): -40.2 (s, 3F); ^{13}C NMR (400 MHz, $CDCl_3$): 149.9, 142.7, 139.4, 129.4, 129.2 (q, $^{1}J_{CF}$ = 309.9 Hz), 128.3, 128.2, 127.8, 127.6, 127.5, 123.6, 24.4; HRMS (EI-EBE) m/z: $[M]^+$ Calc'd for $C_{16}H_{13}F_3S$: 294.0690, Found: 294.0692; Indicative NMR signals of **4b**: ^{1}H NMR (400 MHz, $CDCl_3$): 7.56 (m, 2H), 6.85 (s, 1H), 2.30 (d,3H); ^{13}C NMR (400 MHz, $CDCl_3$): 144.0, 138.4, 137.4, 129.1, 128.1, 127.7, 127.2, 126.4, 126.0, 17.4.

(*Z*)-(2-cyclopropyl-1,2-diphenylvinyl)trifluoromethyl)sulfane (3c) Prepared by reaction of 2-cyclo-

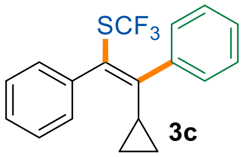

3c

propyl-1-phenyl ethyne (28.6 mg, 0.201 mmol) with phenylmagnesium bromide following general procedure. The title compound **3c** was isolated as a white solid (40.5 mg, 0.126 mmol, 63 %) after chromatography on silica gel (15 g column, 40/60 petroleum ether). Analytical data of **3c**: ^{1}H NMR (400 MHz, $CDCl_3$): 7.57 (d, J = 7.4 Hz, 2H), 7.37 (m, 6H), 7.08 (d, J = 6.8 Hz, 2H), 1.85 (tt, 1H), 0.61 (m, 2H), 0.39 (m, 2H); ^{19}F NMR (400 MHz, $CDCl_3$): -40.5 (s, 3F); ^{13}C NMR (400 MHz, $CDCl_3$): 155.7, 139.4, 137.1, 130.0, 129.1 (q, $^{1}J_{CF}$ = 310.1 Hz), 129.0, 128.2, 127.8, 127.7, 127.4, 122.5, 29.7, 16.7, 6.5; HRMS (EI-EBE) m/z: $[M]^+$ Calc'd for $C_{18}H_{15}F_3S$: 320.0842, Found: 320.0844.

(*Z*)-(3-methyl-1,2-diphenylbut-1-en-1-yl)trifluoromethyl)sulfane (3d) Prepared by reaction of 3-me-

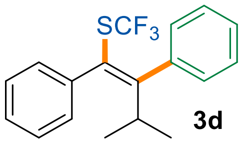

3d

thyl-1-phenyl butyne (29.1 mg, 0.201 mmol) with phenylmagnesium bromide following general procedure. The title compound was isolated as a white solid (44.5 mg) after chromatography on silica gel (15 g column, 40/60 petroleum ether), containing **3d** (42.5 mg, 0.131 mmol, 66%) along with small amount of **4d** (2.0 mg, 0.008 mmol, 5%). Analytical data of **3d**: ^{1}H NMR (400 MHz, $CDCl_3$): 7.37 (m, 8H), 7.11 (m, 2H), 2.92 (hept, 1H), 0.89 (d, J = 6.9 Hz, 6H); ^{19}F NMR (400 MHz, $CDCl_3$): -39.8 (s, 3F); ^{13}C NMR (400 MHz, $CDCl_3$): 158.7, 138.9, 138.0, 129.2, 129.1 (q, $^{1}J_{CF}$ = 309.7 Hz), 128.9, 128.2, 127.8, 127.8, 127.2, 124.3, 32.6, 21.0; HRMS (EI-EBE) m/z: $[M]^+$ Calc'd for $C_{18}H_{17}F_3S$: 322.0998, Found: 322.1005; Indicative NMR signals of **4d**: ^{1}H NMR (400 MHz, $CDCl_3$): 6.37 (s, 1H), 3.31 (m, 1H), 1.08 (d, J = 7.0 Hz, 6H); ^{13}C NMR (400 MHz, $CDCl_3$): 128.8, 128.1, 127.5, 126.5, 126.4, 29.7, 21.9.

(*Z*)-(5-chloro-1,2-diphenylpent-1-en-1-yl))trifluoromethyl)sulfane (3e) Prepared by reaction of 5-chloro-1-phenyl pent-1-yne (36.1 mg, 0.202 mmol) with phenylmagnesium bromide following general procedure. The title compound was isolated as a colorless oil (48.6 mg) after chromatography on silica gel (15 g column, 40/60 petroleum ether), containing **3e** (45.1 mg, 0.126 mmol, 63%) along with small amount of **4e** (3.5 mg, 0.013 mmol, 7%). Analytical data of **3e**: ^{1}H NMR (400 MHz, $CDCl_3$): 7.40 (m, 8H), 7.25 (m, 2H), 3.30 (t, J = 6.6 Hz, 2H), 2.52 (m, 2H), 1.71 (m, 2H); ^{19}F NMR (400 MHz, $CDCl_3$): -39.8 (s, 3F); ^{13}C NMR (400 MHz, $CDCl_3$): 151.9, 140.3, 138.8, 129.2 (q, $^{1}J_{CF}$ = 309.9 Hz), 129.2, 128.4, 128.4, 128.1, 128.1, 127.8, 125.4, 44.1, 34.1, 31.0; HRMS (EI-EBE) m/z: $[M]^+$ Calc'd for $C_{18}H_{16}ClF_3S$: 356.0613, Found: 356.0620. Indicative NMR signals of **4e**: ^{1}H NMR (400 MHz, $CDCl_3$): 7.48 (m, 2H), 7.45 (m, 4H), 7.31 (dd, 4H), 6.78 (s,1H), 3.5 (t, J = 6.6 Hz, 2H), 2.90 (m, 2H), 1.91 (m, 2H); ^{13}C NMR (400 MHz, $CDCl_3$): 142.4, 141.4, 137.9, 129.2, 128.7, 128.5, 128.3, 127.4, 126.7, 126.5, 44.8, 31.6, 27.6.

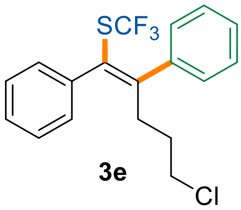

3e

(Z)-(trifluoromethyl)(1,2,3-triphenylprop-1-en-1-yl)sulfane (3f) Prepared by reaction of 1,3-diphenyl propyne (38.7 mg, 0.201 mmol) with phenylmagnesium bromide following general procedure. The title compound **3f** was isolated as a white solid (53.2 mg, 0.143 mmol, 72%) after chromatography on silica gel (15 g column, 40/60 petroleum ether). Analytical data of **3f**: ^{1}H NMR (400 MHz, $CDCl_3$): 7.55 (m, 2H), 7.46 (m, 2H), 7.39 (m, 1H), 7.31 (m, 3H), 7.16 (m, 3H), 7.09 (m, 2H), 6.88 (m, 2H), 3.77 (s, 2H); ^{19}F NMR (400 MHz, $CDCl_3$): -39.6 (s, 3F); ^{13}C NMR (400 MHz, $CDCl_3$): 151.5, 140.4, 138.9, 137.7, 129.5, 129.2 (q, $^{1}J_{CF}$ = 309.9 Hz), 128.9, 128.5, 128.4, 128.2, 128.1, 128.0, 127.5, 126.3, 125.8, 42.5; HRMS (EI-EBE) m/z: $[M]^+$ Calc'd for $C_{22}H_{17}F_3S$: 370.1003, Found: 370.0998.

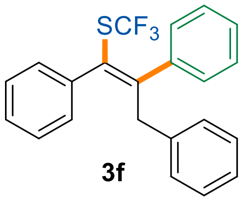

3f

(*Z*)-(1-(4-methoxyphenyl)-2-phenylhex-1-en-1-yl))trifluoromethyl)sulfane (3g) Prepared by reaction of 1-(4-methoxyphenyl)hex-1-yne (37.8 mg, 0.200 mmol) with phenylmagnesium bromide following general procedure. The title compound **3g** was isolated as a colorless oil (45.5 mg, 0.200 mmol, 62%) after chromatography on silica gel (15 g column, 40/60 petroleum ether/diethyl ether 100:0 to 97:3). Analytical data of **3g**: ^{1}H NMR (400 MHz, $CDCl_3$): 7.40 (m, 2H), 7.33 (m, 3H), 7.22 (m, 2H), 6.93 (m, 2H) 3.85 (s, 3H), 2.37 (t, J = 8.0 Hz, 2H), 1.18 (m, 4H), 0.72 (t, J = 7.2 Hz, 3H); ^{19}F NMR (400 MHz, $CDCl_3$): -40.0 (s, 3F); ^{13}C NMR (400 MHz, $CDCl_3$): 159.1, 153.7, 141.3, 131.5, 130.5, 129.3 (q, $^{1}J_{CF}$ = 310.1 Hz), 128.1, 128.1, 127.3, 123.7, 113.5, 55.2, 36.4, 30.1, 22.3, 13.7; HRMS (EI-EBE) m/z: $[M]^+$ Calc'd for $C_{20}H_{21}F_3OS$: 366.1265 Found: 366.1277.

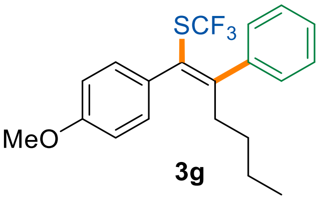

3g

(*Z*)-(1-(naphthalen-1-yl)-2-phenylhex-1-en-1-yl))trifluoromethyl)sulfane (3h) Prepared by reaction of 1-(hex-1-yn-1-yl)naphthalene (42.07 mg, 0.201 mmol) with phenylmagnesium bromide following general procedure. The title compound was isolated as a colorless oil (45.0 mg, 0.116 mmol, 58%) after chromatography on silica gel (15 g column, 40/60 petroleum ether). Analytical data of **3h**: ^{1}H NMR (400 MHz, $CDCl_3$, 8.00 (d, J = 7.4 Hz, 1H), 7.89 (ddd, 2H), 7.54 (m, 6H), 7.41 (d, 1H), 7.37(d, 2H), 2.22 (m, 2H), 1.16 (m, 2H), 0.99 (m, 2H), 0.57 (t, J = 7.3 Hz, 3H); ^{19}F NMR (400 MHz, $CDCl_3$): -39.6 (s, 3F); ^{13}C NMR (400 MHz, $CDCl_3$): 156.2, 140.6, 135.9, 133.8, 130.9, 128.7, 128.6, 128.3, 128.2, 127.6, 127.0 (q, $^{1}J_{CF}$ = 306.8 Hz), 126.3, 126.0, 125.2, 125.0, 121.5, 36.7, 29.7, 22.2, 13.5; HRMS (EI-EBE) m/z: $[M]^+$ Calc'd for $C_{23}H_{21}F_3S$: 386.1311 Found: 386.1326.

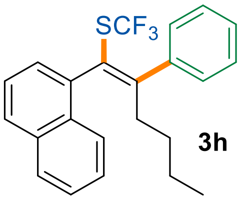

3h

(*Z*)-(1-(4-fluorophenyl)-2-phenylhex-1-en-1-yl))trifluoromethyl)sulfane (3i) Prepared by reaction of 1-(4-fluorophenyl)hex-1-yne (35.7 mg, 0.202 mmol) with phenylmagnesium bromide following general procedure. The title compound was isolated as a colorless oil (42.6 mg) after chromatography on silica gel (15 g column, 40/60 petroleum ether), containing **3i** (39.7 mg, 0.112 mmol, 56%) along with small amount of **4i** (2.9 mg, 0.011 mmol, 6%). Analytical data of **3i**: ^{1}H NMR (400 MHz, $CDCl_3$): 7.41 (m, 5H), 7.25 (d, *J* = 6.8 Hz 2H), 7.13 (t, *J* = 8.7 Hz, 2H), 2.38 (t, *J* = 7.9 Hz, 2H), 1.20 (m 4H), 0.75 (t, *J* = 7.2 Hz, 3H); ^{19}F NMR (400 MHz, $CDCl_3$): -39.9 (s, 3F), -113.6 (s, 1F); ^{13}C NMR (400 MHz, $CDCl_3$): 163.5, 161.0, 154.7, 140.9, 135.2 (d, *J* = 3.41 Hz), 131.1(d, *J* = 8.11.5hz), 129.2 (q, $^1J_{CF}$ = 309.8 Hz), 128.2, 128.0, 127.5, 122.9, 115.4 (d), 36.4, 30.0, 22.3, 13.6; HRMS (EI-EBE) m/z: $[M]^+$ Calc'd for $C_{19}H_{18}F_4S$: 354.1060 Found: 354.1071. Indicative NMR signals of **4i**: ^{1}HNMR (400 MHz, $CDCl_3$): 7.48 (d, 3H), 7.31 (dd, 4H), 7.07 (d, *J* = 8.7 Hz, 2H), 6.67 (s, 1H), 2.70 (t, *J* = 8.2 Hz, 2H), 1.40 (m, 4H) 0.88 (t, *J* = 7.2 Hz, 3H); ^{19}F NMR (400 MHz, $CDCl_3$): -115.7 (s, 1F); ^{13}C NMR (400 MHz, $CDCl_3$: 162.7, 160.2, 143.4, 142.9, 134.3 (d, *J* = 3.35 Hz), 130.3 (d, *J* = 7.9 Hz), 128.3, 127.1, 126.8, 126.5, 115(d), 30.8, 29.8, 22.7, 13.8.

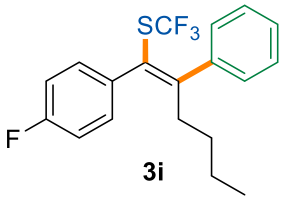

3i

(*Z*)-4-(2-phenyl-1-((trifluoromethyl)thio)hex-1-en-1-yl)phenyl 4-methylbenzenesulfonate (3j) Prepared by reaction of 1-(4-tosylatephenyl)hex-1-yne (65.9 mg, 0.200 mmol) with phenylmagnesium bromide following general procedure. The title compound was isolated as a yellow oil (54.9 mg) after chromatography on silica gel (15 g column, 40/60 petroleum ether/diethyl ether 10:0 to 9:1), containing **3j** (49.1 mg, 0.096 mmol, 49%) along with small amount of **4j** (5.8 mg, 0.014 mmol, 7%). Analytical data of **3j**: ^{1}H NMR (400 MHz, $CDCl_3$): 7.67 (dd, 2H), 7.39 (dd, 2H), 7.31 (dd, 3H), 7.28 (d, 2H), 7.18 (m, 2H), 7.01 (dd, 2H), 2.43 (s, 3H), 2.30 (t, *J* = 7.3 Hz, 2H), 1.15 (m, 4H), 0.70 (t, *J* = 7.2 Hz, 3H); ^{19}F NMR (400 MHz, $CDCl_3$): -39.9 (s, 3F); ^{13}C NMR (400 MHz, $CDCl_3$): δ 154.9, 149.0, 145.4, 140.6, 138.2, 130.6, 129.6, 129.1 (q), 128.6, 128.2, 127.9, 127.6, 126.5, 122.5, 122.3 36.4, 29.9, 22.3, 21.6, 13.6. HRMS (EI-EBE) m/z: $[M]^+$ Calc'd for $C_{26}H_{25}F_3O_3S_2$: 506.1197 Found: 506.1213. Indicative NMR signals of **4j**: ^{1}H NMR (400 MHz, $CDCl_3$): 7.72 (dd, 2H), 7.42 (m, 2H), 7.35 (m, 3H), 7.33 (d, 2H), 7.21 (dd, 2H), 6.97 (dd, 2H), 6.59 (s, 1H), 2.62 (t, *J* = 7.4 Hz, 2H), 2.45 (s, 3H), 1.33 (m, 4H), 0.82 (t, *J* = 7.2 Hz, 3H); ^{13}C NMR (101 MHz, $CDCl_3$) δ 147.9, 145.2, 144.3, 142.7, 137.3, 132.4, 131.9, 129.8, 129.7, 128.5, 128.3, 127.3, 126.6, 122.1, 30.7, 29.8, 22.6, 21.7, 13.7.

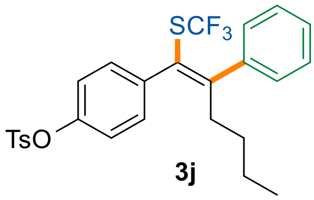

3j

(*Z*)-4-(2-phenyl-1-((trifluoromethyl)thio)hex-1-en-1-yl)phenyl trifluoromethanesulfonate (3k) Prepared by reaction of 1-(4-triflatephenyl)hex-1-yne (61.5 mg, 0.200 mmol) with phenylmagnesium bromide following general procedure. The title compound was isolated as a yellow oil (47.5 mg) after chromatography on silica gel (15 g column, 40/60 petroleum ether), containing **3k** (43.5 mg, 0.089 mmol, 45%) along with small amount of **4k** (4.0 mg, 0.010 mmol, 5%). Analytical data of **3k**: ^{1}H NMR (400 MHz, $CDCl_3$): 7.50 (m, 2H), 7.40 (m, 3H), 7.34 (m, 2H), 7.21 (m, 2H), 2.35 (t, *J* = 7.3 Hz, 2H), 1.19 (m, 4H), 0.71 (t, *J* = 7.2 Hz, 3H); ^{19}F NMR (400 MHz, $CDCl_3$): -39.9 (s, 3F), -72.7 (s, 3F); ^{13}C NMR (400 MHz, $CDCl_3$): 156.0, 148.8, 140.4, 139.8, 131.2, 129.0 (q, , $^1J_{CF}$ = 309.8 Hz), 128.3 127.9, 127.7, 121.9, 121.2, 118.7 (q, $^1J_{CF}$ = 320.8 Hz), 36.4, 29.9, 22.2, 13.5; HRMS (EI-EBE) m/z: $[M]^+$ Calc'd for $C_{20}H_{18}F_6O_3S_2$: 484.0602 Found: 484.0591. Indicative NMR signals of **4k**: ^{1}H NMR (400 MHz, $CDCl_3$): 7.46 (d, 1H), 7.27 (m, 2H), 6.64 (s, 1H), 2.67 (t, *J* = 7.6 Hz, 2H), 1.37 (m, 4H), 0.84 (t, *J* = 7.2 Hz, 3H); ^{19}F NMR (400 MHz, $CDCl_3$): -72.8 (s, 3F); ^{13}C NMR (400 MHz, $CDCl_3$): 147.9, 145.2, 142.5, 138.7, 130.4, 128.3, 127.5, 126.5, 126.0, 121.1, 30.7, 22.6, 13.7.

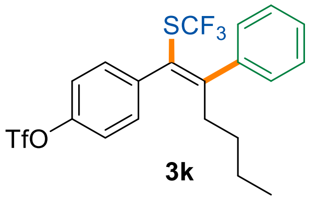

3k

(*Z*)-(1-(4-(dimethoxymethyl)phenyl)-2-phenylhex-1-en-1-yl)(trifluoromethyl)sulfane (3l) Prepared by reaction of 1-(4-dimethoxymethylphenyl)hex-1-yne (46.4 mg, 0.199 mmol) with phenylmagnesium bromide following general procedure. The title compound was isolated as a yellow oil (49.2 mg) after chromatography on silica gel (15 g column, 40/60 petroleum ether/methyl tert-butyl ether 98.5:1.5 to 96:4), containing **3l** (45.0 mg, 0.109 mmol, 55%) along with small amount of **4l** (4.2 mg, 0.013 mmol, 7%). Analytical data of **3l**: ^{1}H NMR (400 MHz, $CDCl_3$): 7.50 (d, 2H), 7.41 (m, 4H), 7.35 (m, 1H), 7.23 (d, 2H), 5.46 (s, 1H), 3.36 (s, 6H), 2.37 (t, *J* = 7.4 Hz, 2H), 1.19 (m, 4H), 0.70 (t, *J* = 7.2 Hz, 3H); ^{19}F

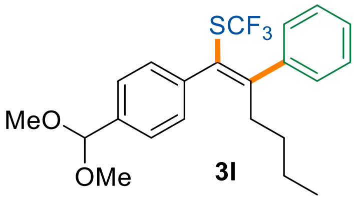

3l

NMR (400 MHz, $CDCl_3$): -40.0 (s, 3F); ^{13}C NMR (400 MHz, $CDCl_3$): 154.5, 141.0, 139.5, 137.6, 129.2, 129.2 (q, $^{1}J_{CF}$ = 309.8 Hz), 128.1, 128.0, 127.4, 126.6, 126.5, 123.5 102.8, 52.6, 36.4, 30.0, 22.3, 13.6; HRMS (EI-EBE) m/z: $[M]^+$ Calc'd for $C_{22}H_{25}F_3O_2S$: 410.1527, Found: 410.1538. Indicative NMR signals of **4l**: ^{1}H NMR (400 MHz, $CDCl_3$: 6.69 (s, 1H), 5.42 (s, 1H), 2.71 (t, *J* = 7.5 Hz, 2H), 1.37 (m, 4H), 0.85 (t, *J* = 7.3 Hz, 3H); ^{13}C NMR (400 MHz, $CDCl_3$): 143.7, 143.1, 138.5, 136.2, 129.4, 128.6, 128.2, 127.7, 127.3, 127.1, 126.4, 103.1, 52.7, 30.8, 29.9, 22.7, 13.8.

(*Z*)-5-(2-phenyl-1-((trifluoromethyl)thio)hex-1-en-1-yl)benzo[d][1,3]dioxole (3m) Prepared by reaction of 5-(hex-1-yn-1-yl)benzo[*d*][1,3]dioxole (40.7 mg, 0.201 mmol) with phenylmagnesium bromide following modified general procedure (carbomagnesiation run for 3 h). The title compound **3m** was isolated as a yellow oil (50.5 mg, 0.132 mmol, 66%) after chromatography on silica gel (15 g column, 40/60 petroleum ether/diethyl ether 100:0 to 97:3). Analytical data of **3m**: ^{1}H NMR (400 MHz, $CDCl_3$): 7.39 (m, 3H), 7.24 (dd, 2H), 6.89 (dd+s, 3H), 6.04 (s, 2H), 2.42 (t, *J* = 7.1 Hz, 2H), 1.23 (m, 4H), 0.77 (t, *J* = 7.1 Hz, 3H); ^{19}F NMR (400 MHz, $CDCl_3$): -40.0 (s, 3F); ^{13}C NMR (400 MHz, $CDCl_3$): 154.2, 147.5, 147.2, 141.1, 133.0, 129.3 (q, $^{1}J_{CF}$ = 310.1 Hz), 128.1, 128.0, 127.4, 123.6, 123.0, 109.7, 108.0, 101.2, 36.5, 30.1, 22.4, 13.6; HRMS (EI-EBE) m/z: $[M]^+$ Calc'd for $C_{20}H_{19}F_3O_2S$: 380.1058 Found: 380.1059.

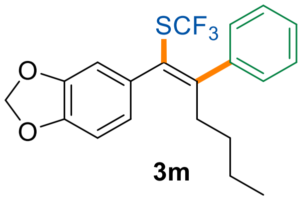

3m

(*Z*)-1-methyl-5-(2-phenyl-1-((trifluoromethyl)thio)hex-1-en-1-yl)-1H-indole (3n) Prepared by reaction of 5-(hex-1-yn-1-yl)-1-methyl-1*H*-indole (42.5 mg, 0.201 mmol) with phenylmagnesium bromide following general procedure. The title compound was isolated as a yellow oil (38.3 mg, 0.098 mmol, 49%) after chromatography on silica gel (15 g column, 40/60 petroleum ether/ethyl acetate 99:1 to 9:1) Analytical data of **3n**: ^{1}H NMR (400 MHz, $CDCl_3$): 7.60 (d, 1H), 7.43 (m, 2H), 7.35 (m, 2H), 7.28 (m, 3H), 7.09 (d, *J* = 3.1 Hz, 1H), 6.53 (d, *J* = 3.0, 0.8 Hz, 1H), 3.82 (s, 3H), 2.41 (t, *J* = 7.6 Hz, 2H), 1.25 (m, 2H), 1.13 (m, 2H), 0.72 (t, *J* = 7.3 Hz, 3H); ^{19}F NMR (400 MHz, $CDCl_3$): -39.9 (s, 3F); ^{13}C NMR (400 MHz, $CDCl_3$): 153.2, 141.6, 136.2, 130.3, 129.5 (q, $^{1}J_{CF}$ = 309.7 Hz), 129.3, 128.2, 128.1, 128.1, 127.2, 125.2, 123.2, 121.8, 36.5, 32.9, 30.2, 22.4, 13.7; HRMS (EI-EBE) m/z: $[M]^+$ Calc'd for $C_{22}H_{22}F_3NS$: 389.1425 Found: 389.1420.

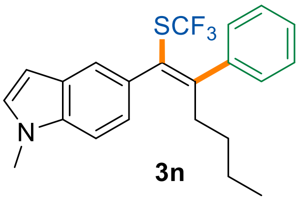

3n

(*Z*)-2-(2-phenyl-1-((trifluoromethyl)thio)hex-1-en-1-yl)thiophene (3o) Prepared by the reaction of 2-(hex-1-yn-1-yl)thiophene (33.0 mg, 0.200 mmol) with phenylmagnesium bromide following modified general procedure (carbomagnesiation at 60 ºC). The title compound was isolated as a yellow oil (35.3 mg) after chromatography on silica gel (15 g column, 40/60 petroleum ether), containing **3o** (31.5 mg, 0.091 mmol, 46%) along with small amount of **4o** (3.8 mg, 0.015 mmol, 8%). Analytical data of **3o**: ^{1}H NMR (400 MHz, $CDCl_3$): 7.38 (m, 4H), 7.21 (m, 2H), 7.14 (dd, 1H), 7.03 (dd, 1H), 2.55 (t, *J* = 7.5 Hz, 2H), 1.31 (m, 2H), 1.21 (m, 2H), 0.78 (t, *J* = 7.2 Hz, 3H); ^{19}F NMR (400 MHz, $CDCl_3$): -40.8 (s, 3F); ^{13}C NMR (400 MHz, $CDCl_3$): 157.8, 141.5, 141.2, 129.2 (q, $^{1}J_{CF}$ = 310.8 Hz), 128.1, 127.9, 127.5, 126.6, 126.5, 126.3, 116.5, 37.2, 30.3, 22.4, 13.7; HRMS (EI-EBE) m/z: $[M]^+$ Calc'd for $C_{17}H_{17}F_3S_2$: 342.0724 Found: 342.0712. Indicative NMR signals of **4o**: ^{1}H NMR (400 MHz, $CDCl_3$): 7.46 (m, 2H), 7.32 (m, 2H), 7.27 (m, 2H), 7.06 (dd, 2H), 6.84 (s, 1H), 2.89 (t, *J* = 7.4 Hz, 2H), 1.48 (m, 4H), 0.92 (t, *J* = 7.2 Hz, 3H); ^{13}C NMR (400 MHz, $CDCl_3$: 143.2, 141.5, 140.9, 128.3, 128.1, 127.7, 127.1, 126.8, 124.8, 120.8, 31.3, 30.5, 22.9, 13.9.

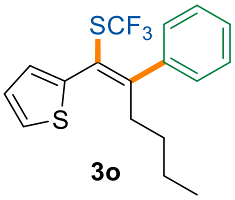

3o

((*Z*)-(1-(cyclohex-1-en-1-yl)-2-phenylhex-1-en-1-yl)(trifluoromethyl)sulfane (3p) Prepared by the reaction of 1-(hex-1-yn-1-yl)cyclohex-1-ene (32.4 mg, 0.199 mmol) with phenylmagnesium bromide following modified general procedure (carbomagnesation at 60 ºC, 4 h). The title compound was isolated as a colorless oil (14.5 mg, 0.042 mmol, 21%) after chromatography on silica gel (15 g column, 40/60 petroleum ether). Analytical data of **3p**: ^{1}H NMR (400 MHz, $CDCl_3$): 7.31 (m, 3H), 7.11 (m, 2H), 5.77 (t, 1H), 2.49 (t, 2H), 2.24 (m, 2H), 2.15 (m, 2H), 1.72 (m, 2H), 1.65 (m, 2H), 1.21 (m, 4H), 0.8 (t, *J* = 6.9 Hz, 3H); ^{19}F NMR (400 MHz, $CDCl_3$): -40.3 (s, 3F); ^{13}C NMR (400 MHz, $CDCl_3$): 151.4, 141.1, 135.2, 129.2 (q, $^{1}J_{CF}$ = 309.3 Hz), 128.7, 128.5, 128.2, 128.0, 127.1, 36.2, 30.4,

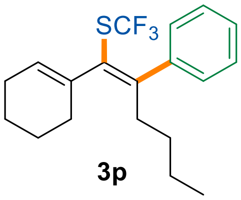

3p

27.6, 25.4, 22.7, 22.4, 22.0, 13.7; HRMS (EI-EBE) m/z: $[M]^+$ Calc'd for $C_{19}H_{23}F_3S$: 340.1473 Found: 340.1466.

(trifluoromethyl)(1,2,2-triphenylvinyl)sulfane (3q) Prepared by the reaction of diphenylacetylene (35.8 mg, 0.200 mmol) with phenylmagnesium bromide following general procedure. The title compound was isolated as a white solid (48.0 mg) after chromatography on silica gel (15 g column, 40/60 petroleum ether), containing **3q** (44.5 mg, 0.125 mmol, 63%) along with small amount of **4q** (3.4 mg, 0.013 mmol, 7%). Analytical data of **3q**: ^{1}H NMR (400 MHz, $CDCl_3$): 7.37 (m, 7H), 7.20 (m, 3H), 7.08 (m, 3H), 6.96 (dd, 2H); ^{19}F NMR (400 MHz, $CDCl_3$): -39.5 (s, 3F); ^{13}C NMR (400 MHz, $CDCl_3$): 152.4, 142.6, 141.4, 139.3, 130.5, 130.4, 129.6, 129.3 (q, $^{1}J_{CF}$ = 310.1 Hz), 128.2, 127.9, 127.8, 127.8, 127.7, 127.4, 126.1; HRMS (EI-EBE) m/z: $[M]^+$ Calc'd for $C_{21}H_{15}F_3S$: 356.0847 Found: 356.0839; Indicative NMR signals of **4q**: ^{1}H NMR (400 MHz, $CDCl_3$): 7.22 (m, 2H), 7.14 (m, 3H), 7.00 (s, 1H); ^{13}C NMR (400 MHz, $CDCl_3$): 143.4, 140.3, 137.4, 130.4, 129.5, 128.6, 128.2, 128.1, 127.6, 126.7.

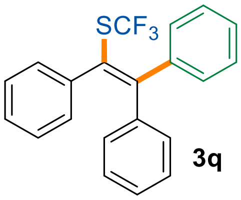

3q

(E)-(2-(4-methoxyphenyl)-1,2-diphenylvinyl)(trifluoromethyl)sulfane (3r) Prepared by the reaction of 4-methoxy diphenylacetylene (42.0 mg, 0.201 mmol) with phenyl magnesium bromide following general procedure. The title compound was isolated as a yellow oil (59.0 mg) after chromatography on silica gel (15g column, 60/90 petroleum ether/diethyl ether 99:1 to 96:4) containing **3r** and **3r'** (54.3 mg, 0.148 mmol, 74%, 7:3) along with small amount of **4r** and **4r'** (4.7 mg, 0.017 mmol, 9%). Analytical data of ***E*-3r**: ^{1}H NMR (400 MHz, $CDCl_3$): 7.37 (m, 10H), 6.87 (dd, 2H), 6.60 (dd, 2H), 3.71 (s, 3H); ^{19}F NMR (400 MHz, $CDCl_3$): -39.6 (s, 3H); ^{13}C NMR (400 MHz, $CDCl_3$): 158.8, 152.1, 142.9, 139.8, 133.7, 131.9, 130.4, 129.6, 129.3 (q, $^{1}J_{CF}$ = 310.8 Hz), 128.1 (d), 127.8, 127.1, 124.6, 113.2, 55.0; Analytical data of **3r'**: ^{1}H NMR (400 MHz, $CDCl_3$): 7.29 (dd, 2H), 7.22 (m, 5H), 7.09 (dd, 3H), 6.95 (dd, 2H), 6.72 (dd, 2H), 3.77 (s, 3H); ^{19}F NMR (400 MHz, $CDCl_3$): -39.5 (s, 3H); ^{13}C NMR (400 MHz, $CDCl_3$): 159.1, 151.2, 142.8, 141.7, 131.8, 131.6, 130.3, 129.6, 129.5, 129.4 (q), 128.2, 127.6 (d), 127.6, 113.4, 55.1; HRMS (EI-EBE) m/z: $[M]^+$ Calc'd for $C_{22}H_{17}F_3OS$: 386.0952 Found: 386.0950. Indicative NMR signals of **4r** and **4r'**:^{1}H NMR (400 MHz, $CDCl_3$): 7.15 (m, 8H), 6.92 (s, 1H), 6.66 (dd, 2H), 3.84 (s, 3H), *Z*- isomer 3.75 (s, 3H); ^{13}C NMR (400 MHz, $CDCl_3$): 132.5, 131.5, 130.8, 130.4, 128.7, 128.1, 127.9, 127.4, 126.6, 114.0, 113.4, 55.1.

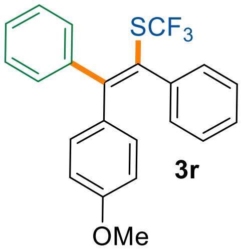

3r

(1-(naphthalen-2-yl)-2,2-diphenylvinyl)(trifluoromethyl)sulfane (3s) Prepared by the reaction of 2-(phenylethynyl)naphthalene (45.8 mg, 0.200 mmol) with phenyl magnesium bromide following general procedure. The title compound was isolated as a white solid (50.5 mg) after chromatography on silica gel (15g column, 60/90 petroleum ether) containing **3s** and **3s'** (45.6 mg, 0.112 mmol, 56%, 6:4) along with small amount of **4s** and **4s'** (4.9 mg, 0.016 mmol, 8%). Analytical data of **3s**: ^{1}H NMR (400 MHz, $CDCl_3$): 7.92 (s, 1H), 7.74 (dd, 2H), 7.65 (d, J = 8.6 Hz, 1H), 7.41 (m, 8H), 7.36 (m, 2H), 7.16 (m, 1H), 7.04 (m, 2H); ^{19}F NMR (400 MHz, $CDCl_3$): -39.4; ^{13}C NMR (400 MHz, $CDCl_3$): 152.9, 142.7, 141.4, 139.0, 130.5, 130.4, 129.8, 129.6, 129.3 (q, $^{1}J_{CF}$ = 310.8 Hz), 128.3(d), 128.1, 127.9, 127.9, 127.9, 127.8, 127.6, 127.5, 126.4, 126.0; Analytical data of ***E*-3s'**: ^{1}H NMR (400 MHz, $CDCl_3$): 7.68 (s, 1H), 7.54 (d, J = 8.7 Hz, 2H), 7.08 (m, 1H), 6.99 (m, 2H); ^{19}F NMR (400 MHz, $CDCl_3$): -39.4; ^{13}C NMR (400 MHz, $CDCl_3$): 152.2, 139.3, 136.9, 133.0, 132.8, 132.6, 132.3, 130.2, 130.1, 128.2, 128.1(d), 128.0 (q), 127.2; HRMS (EI-EBE) m/z: $[M]^+$ Calc'd for $C_{25}H_{17}F_3S$: 406.1003 Found: 406.0995. Indicative NMR signals of **4s** and **4s'**: ^{13}C NMR (400 MHz, $CDCl_3$): 143.4, 143.0, 142.4, 140.4, 135.1, 129.6, 129.1, 128.7, 128.2, 128.0, 127.7, 127.1, 125.8(d).

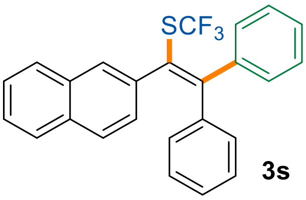

3s

(1-(naphthalen-1-yl)-2,2-diphenylvinyl)(trifluoromethyl)sulfane (3t) Prepared by the reaction of 1-(phenylethynyl)naphthalene (46.2 mg, 0.202 mmol) with phenyl magnesium bromide following general procedure. The title compound was isolated as a white solid (51 mg) after chromatography on silica gel (15g column, 60/90 petroleum ether) containing **3t** and **3t'** (47.3 mg, 0.116 mmol, 58%, 7:3) along with a small amount of **4t** and **4t'** (3.6 mg, 0.0118 mmol, 6%). Analytical data of **3t**: ^{1}H NMR (400 MHz, $CDCl_3$): 8.19 (d, J = 8.3 Hz, 1H), 7.77 (d, J = 8.2 Hz, 1H), 7.75 (d, J = 1.9 Hz, 1H), 7.43 (m, 10H), 7.02 (d, J = 2.6 Hz, 1H), 6.93

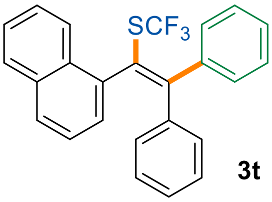

3t

(d, J = 2.1 Hz, 3H); ^{19}F NMR (400 MHz, $CDCl_3$): -39.2 (s, 3F); ^{13}C NMR (400 MHz, $CDCl_3$): δ 153.7, 141.9, 141.1, 136.1, 133.5, 131.0, 129.7, 129.3 (q, $^1J_{CF}$ = 310.8 Hz), 129.2, 129.0, 128.3, 128.1, 127.9, 127.6, 126.2, 125.7, 125.3,125.1, 124.3; Analytical data of ***E*-3t'**: ^{1}H NMR (400 MHz, CDCl3) 8.00 (d, J = 9.0 Hz, 1H), 7.83 (d, J = 7.8 Hz, 1H), 7.67 (d, J = 8.1 Hz, 1H), 7.04 (d, J = 1.9 Hz, 1H), 7.01 (m, 1H), 6.95 (m, 1H), 6.91 (m, 1H); ^{19}F NMR (400 MHz, $CDCl_3$): -39.3 (s, 3F) ^{13}C NMR (400 MHz, $CDCl_3$): 151.4, 141.4, 139.6, 139.2, 133.5, 129.1, 129.1 (q, $^1J_{CF}$ = 311.8 Hz), 128.8, 128.5, 128.3, 128.1, 127.9, 127.7, 127.4, 126.6, 126.3, 125.7, 125.6, 125.0; HRMS (EI-EBE) m/z: $[M]^+$ Calc'd for $C_{25}H_{17}F_3S$: 406.1003 Found: 406.0997. Indicative NMR signals of **4t** and **4t'**: ^{1}H NMR (400 MHz, $CDCl_3$): 7.93 (dd, 2H), 7.04 (dd, 3H); ^{13}C NMR (400 MHz, $CDCl_3$): 142. 9, 140.4, 137.8, 137.0, 132.0, 129.8, 129.1, 127.8, 126.9, 126.3, 126.0, 125.9, 125.9.

Scope of grignard reagent :

(*Z*)-(2-(4-methoxyphenyl)-1-phenylhex-1-en-1-yl) (trifluoromethyl)sulfane (3u) Prepared by the reaction of 1-phenyl-hexyne (31.8 mg, 0.200 mmol) with 4-methoxy phenyl magnesium bromide following general procedure. The title compound was isolated as a yellow oil (48.8 mg) after chromatography on silica gel (15g column, 60/90 petroleum ether/diethyl ether- 100 to 97:3) containing **3u** (47 mg, 0.128 mmol, 64%) along with a small amount of **4u** (1.8 mg, 0.006 mmol, 3%). Analytical data of **3u**: ^{1}H NMR (400 MHz, $CDCl_3$): 7.40 (m, 4H), 7.34 (m, 1H), 7.18 (dd, 2H), 6.96 (dd, 2H), 3.86 (s, 3H), 2.35 (t, J = 7.4 Hz, 2H), 1.18 (m, 4H), 0.72 (t, J = 7.2 Hz, 3H); ^{19}F NMR (400 MHz, $CDCl_3$): -39.9 (s, 3F); ^{13}C NMR (400 MHz, $CDCl_3$): 158.9, 153.9, 139.5, 133.2, 129.4, 129.3, 129.3 (q, $^1J_{CF}$ = 309.8 Hz), 128.1, 127.7, 123.5, 113.5, 55.1, 36.4, 30.2, 22.3, 13.7; HRMS (EI-EBE) m/z: $[M]^+$ Calc'd for $C_{20}H_{21}F_3OS$: 366.1265 Found 366.1261. Indicative NMR signals of **4u**: ^{1}H NMR (400 MHz, $CDCl_3$: 7.37 (m, 7H), 6.66 (s, 1H), 3.84 (s, 3H), 2.70 (t, J = 7.6 Hz, 2H), 1.40 (m, 4H), 0.87 (t, J = 7.2 Hz, 3H); ^{13}C NMR (400 MHz, $CDCl_3$): 142.7, 138.5, 135.4, 128.7, 127.6, 126.7, 126.3, 113.7, 55.2, 31.0, 29.9, 22.8, 13.8.

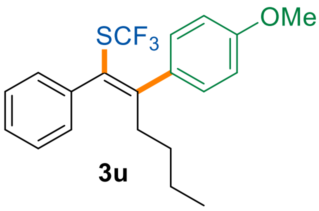

3u

(*Z*)-(2-(4-fluorophenyl)-1-phenylhex-1-en-1-yl)(trifluoromethyl)sulfane (3v) Prepared by the reaction of 1-phenyl-hexyne (32.2 mg, 0.203 mmol) with 4-fluoro phenyl magnesium bromide following general procedure. The title compound was isolated as a colorless oil (38.9 mg) after chromatography on silica gel (15g column, 40/60 petroleum ether) containing **3v** (35.0 mg, 0.203 mmol, 49%) along with a small amount of **4v** (3.9 mg, 0.015 mmol, 8%). Analytical data of **3v**: ^{1}H NMR (400 MHz, $CDCl_3$): 7.40 (m, 4H), 7.35 (m, 1H), 7.20 (m, 2H), 7.10 (m, 2H), 2.35 (t, J = 7.3 Hz, 2H), 1.18 (m, 4H), 0.72 (t, J = 7.2 Hz, 3H); ^{19}F NMR (400 MHz, $CDCl_3$): -40.0 (s, 3F), -114.4 (s, 1F); ^{13}C NMR (400 MHz, $CDCl_3$): 162.1 (d), 153.4, 139.1, 136.9 (d, J = 3.5 Hz), 129.3 (q, $^1J_{CF}$ = 309.9 Hz), 129.8 (d, J = 8.02 Hz), 129.2, 128.7, 128.2, 127.9, 126.5, 124.4, 115.2 (d), 36.4, 30.1, 22.3, 13.6 (d); HRMS (EI-EBE) m/z: $[M]^+$ Calc'd for $C_{19}H_{18}F_4S$: 354.1065 Found: 354.1053. Indicative NMR signals of **4v**: ^{1}H NMR (400 MHz, $CDCl_3$: 7.43 (m, 4H), 7.32 (d, 2H), 7.29 (d, 1H), 7.05 (m, 2H), 6.65 (s, 1H), 2.68 (t, J = 7.3 Hz, 2H), 1.35 (m, 4H), 0.85 (t, J = 7.2 Hz, 3H); ^{19}F NMR (400 MHz, $CDCl_3$): -115.7 (s, 1F); ^{13}C NMR (400 MHz, $CDCl_3$): 162.1 (d), 142.3, 139.1, 138.1, 128.1, 128.0, 115.1 (d), 30.8, 30.0, 22.7, 13.8.

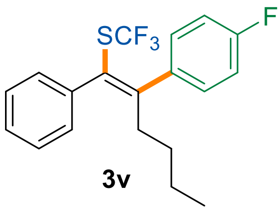

3v

(*Z*)-(1-phenyl-2-(p-tolyl)hex-1-en-1-yl)(trifluoromethyl)sulfane (3w) Prepared by the reaction of 1-phenyl-hexyne (31.9 mg, 0.201 mmol) with *p*-tolyl magnesium bromide following modified general procedure (carbomagnesiation done at r.t., 4h). The title compound was isolated as a colorless oil (51 mg, 0.201 mmol, 73%) after chromatography on silica gel (15g column, 40/60 petroleum ether). Analytical data of **3w**: ^{1}H NMR (400 MHz, $CDCl_3$): 7.43 (m, 4H), 7.37 (m, 1H), 7.27 (d, J = 7.9 Hz, 2H), 7.16 (d, J = 8.0 Hz, 2H), 2.44 (s, 3H), 2.39 (t, J = 7.4 Hz, 2H), 1.22 (m, 4H), 0.75 (t, J = 7.2 Hz, 3H); ^{19}F NMR (400 MHz, $CDCl_3$): -39.9 (s, 3F) (s); ^{13}C NMR (400 MHz, $CDCl_3$): 154.3, 139.5, 138.1, 137.1, 129.3 (q, $^1J_{CF}$ = 309.9 Hz), 129.3, 128.9, 128.1, 128.0, 127.7, 123.5, 36.4, 30.1, 22.3, 21.2, 13.6; HRMS (EI-EBE) m/z: $[M]^+$ Calc'd for $C_{20}H_{21}F_3S$: 350.1316 Found: 350.1322.

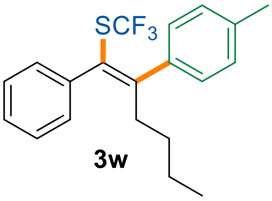

3w

(*Z*)-(1-phenyl-2-(o-tolyl)hex-1-en-1-yl)(trifluoromethyl)sulfane (3x) Prepared by the reaction of 1-phenyl-hexyne (31.9 mg, 0.201 mmol) with *o*-tolyl magnesium bromide following general procedure. The title compound was isolated as a light-yellow oil (50.0 mg) after chromatography on silica gel (15g column, 40/60 petroleum ether) containing **3x** (45.0 mg, 0.201 mmol, 64%) along with a small amount of **4x** (5.0 mg, 0.201 mmol, 10%). Analytical data of **3x**: ^{1}H NMR (400 MHz, $CDCl_3$): 7.43 (m, 4H), 7.37 (m, 1H), 7.28 (d, 2H), 7.24 (m, 1H), 7.06 (d, J = 6.9 Hz, 1H), 2.42 (ddd, 1H), 2.35 (s, 3H), 2.21 (ddd 1H), 1.24 (m, 4H), 0.73 (t, J = 7.3 Hz, 3H); ^{19}F NMR (400 MHz, $CDCl_3$): -39.2 (s, 3F) (s); ^{13}C NMR (400 MHz, $CDCl_3$): 152.3, 140.3, 138.7, 134.5, 130.2, 129.4 (q, $^1J_{CF}$ = 309.8), 129.4, 128.4, 128.2, 127.9, 127.5, 125.5, 125.1, 35.8, 29.8, 22.5, 19.1, 13.6; HRMS (EI-EBE) m/z: $[M]^+$ Calc'd for $C_{20}H_{21}F_3S$: 350.1316 Found: 350.1306. Indicative NMR signals of **4x**: ^{1}H NMR (400 MHz, $CDCl_3$: 7.35 (dd, 3H), 7.20 (dd, 4H), 6.36 (s, 1H), 2.60 (t, J = 7.1 Hz, 2H), 2.38 (s, 3H), 0.85 (t, J = 7.2 Hz, 3H); ^{13}C NMR (400 MHz, $CDCl_3$): 144.1, 143.9, 138.0, 135.2, 130.1, 129.1, 128.7, 128.6, 126.6, 126.4, 125.3, 125.1, 32.4, 30.3, 22.9, 19.9, 13.8.

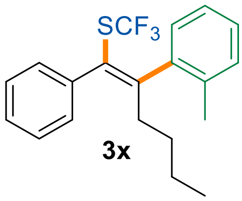

3x

(*Z*)-(2-mesityl-1-phenylhex-1-en-1-yl)(trifluoromethyl)sulfane (3y) Prepared by the reaction of 1-phenyl-hexyne (31.9 mg, 0.201 mmol) with mesityl magnesium bromide following general procedure (carbomagnesiation done at 75 °C). The title compound was isolated as a colorless oil (38 mg, 0.200 mmol, 50%) after chromatography on silica gel (15g column, 40/60 petroleum ether). Analytical data of **3y**: ^{1}H NMR (400 MHz, $CDCl_3$): 7.42 (m, 4H), 7.35 (m, 1H), 6.93 (s, 2H), 2.33 (s, 3H) 2,26 ((s + t), 8H), 1.24 (m, 2H), 1.09 (m, 2H), 0.70 (t, J = 7.3 Hz, 3H); ^{19}F NMR (400 MHz, $CDCl_3$): -38.5 (s, 3F); ^{13}C NMR (400 MHz, $CDCl_3$): 149.8, 138.4, 136.8, 136.7, 134.7, 129.3 (q, $^1J_{CF}$ = 309.9 Hz), 129.3, 128.4, 128.2, 127.9, 125.6, 35.9, 29.9, 22.9, 21.0, 19.7, 13.6; HRMS (EI-EBE) m/z: $[M]^+$ Calc'd for $C_{22}H_{25}F_3S$: 378.1629 Found: 378.1625.

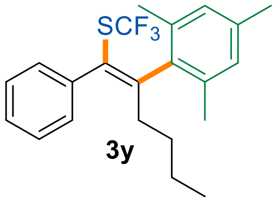

3y

((Z)-1-phenyl-2-((E)-prop-1-en-1-yl)hex-1-en-1-yl)(trifluoromethyl)sulfane (3z)) Prepared by the reaction of 1-phenyl-hexyne (31.75 mg, 0.200 mmol) with prop-1-en-1-yl magnesium bromide following general procedure. The title compound was isolated as a colorless oil (23.5 mg, 0.200 mmol, 39%, *E:Z* 2:1) after chromatography on silica gel (15g column, 40/60 petroleum ether). Analytical data of ***Z*-3z**: ^{1}H NMR (400 MHz, $CDCl_3$: 7.32 (m, 5H), 7.04 (d, J = 13.9 Hz, 1H), 6.08 (dq, 1H), 2.26 (t, J = 8.0 Hz, 2H), 1.95 (dd, J = 5.0 Hz, 3H), 1.39 (m, 4H), 0.79 (t, 3H); ^{19}F NMR (400 MHz, $CDCl_3$): -40.4 (s, 3F); ^{13}C NMR (400 MHz, $CDCl_3$): 150.7, 140.8, 130.9, 129.7 (q, $^1J_{CF}$ = 302.8 Hz), 129.4, 129.1, 128.9, 128.0, 127.6, 127.4, 31.8, 30.6, 22.7, 18.8, 13.6; Analytical data of ***E*-3z':** ^{1}H NMR (400 MHz, $CDCl_3$: 5.76 (dq, 1H), 2.16 (t, J = 7.7 Hz, 2H), 1.73 (dd, J = 5.3 Hz, 3H), 1.19 (m, 4H), 0.79 (t, 3H); ^{19}F NMR (400 MHz, $CDCl_3$): -39.4 (s, 3F); ^{13}C NMR (400 MHz, $CDCl_3$): 149.3, 139.1, 131.1, 129.3 (q, $^1J_{CF}$ = 310.5 Hz), 128.7, 128.4, 127.9, 127.8, 125.9, 121.2, 34.4, 30.2, 22.4, 14.7, 13.7; HRMS (APCI-TOF) m/z: $[M - H]^+$ Calc'd for $C_{16}H_{18}F_3S$: 299.1086 Found: 299.1080.

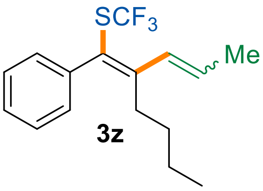

3z

Further transformations of the selected SCF_3-substituted olefins.

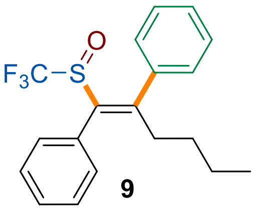

(Z)-(1-((trifluoromethyl)sulfinyl)hex-1-ene-1,2-diyl)dibenzene (9) In a 4mL dark vial add compound **3a** (23.6 mg, 0.070 mmol), 15% H_2O_2 (11.5 µl, 0.112 mmol, 1.6 eq), and CF_3COOH (0.7 mL) under argon. Stir the reaction for 24 h at r.t.. The resultant was poured into water, neutralized with aq. $NaHCO_3$ and extracted with MTBE. The combined organic layers were dried over Na_2SO_4, concentrated, and the residue was purified by flash chromatography on silica gel (60/90 petroleum ether/ethyl acetate 97:3 to 94:6) to provide the desired product as a white solid (21.8 mg, 0.061 mmol, 88%). ^{1}H NMR (400 MHz, $CDCl_3$): δ 7.44 (m, 6H), 7.36 (dd, 2H), 7.23 (m, 2H), 2.39 (m, 2H), 1.18 (m, 4H), 0.71 (t, J = 7.2 Hz, 3H); ^{19}F NMR (376 MHz, $CDCl_3$) δ -67.89 (s, 3F); ^{13}C NMR (400 MHz, $CDCl_3$): δ 160.7, 137.2, 136.7, 130.7, 130.3, 128.9, 128.8, 128.5, 128.2, 128.1, 125.2 (q, $^1J_{CF}$ = 337.9 Hz), 36.9, 29.5, 22.3, 13.5; HRMS (APCI-TOF) m/z: $[M + H]^+$ Calc'd for $C_{19}H_{20}F_3OS$: 353.1182 Found: 353.1189.

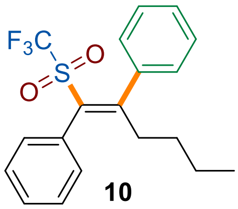

(Z)-(1-((trifluoromethyl)sulfonyl)hex-1-ene-1,2-diyl)dibenzene (10) In a 4mL dark vial add compound **3a** (18.5 mg, 0.055 mmol), mCPBA (57 mg, 0.330 mmol, 6 eq) and dry DCM (0.7 mL) under argon. Close the vial with a cup. Heat the reaction at 65 °C for 17 h. The resultant was quenched with $NaHSO_3$ at 0 °C. Workup with aq. $NaHCO_3$ and MTBE. The combined organic layers were dried over Na_2SO_4, concentrated, and the residue was purified by flash chromatography on silica gel (60/90 petroleum ether/diethyl ether 99.5: 0.5 to 94:6) to provide the desired product as a yellow solid (16.2 mg, 0.043 mmol, 80%). ^{1}H NMR (400 MHz, $CDCl_3$): 7.44 (m, 8H), 7.24 (dd, 2H), 2.29 (t, J = 7.5 Hz, 2H), 1.14 (m, 4H), 0.66 (t, J = 7.3 Hz, 3H); ^{19}F NMR (400 MHz, $CDCl_3$): -74.60 (s, 3F); ^{13}C NMR (400 MHz, $CDCl_3$): δ 166.4, 137.4, 132.1, 131.4, 131.3, 129.5, 128.6, 128.3, 127.7, 126.9, 119.9 (q, $^1J_{CF}$ = 329.2 Hz), 39.8, 29.1, 22.3, 13.4; HRMS (APCI-TOF) m/z: $[M - H]^+$ Calc'd for $C_{19}H_{18}F_3O_2S$: 367.0975 Found: 367.0982.

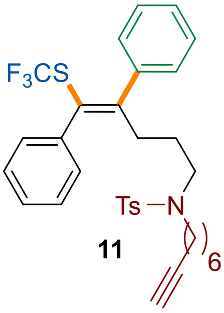

(Z)-N-(4,5-diphenyl-5-((trifluoromethyl)thio)pent-4-en-1-yl)-4-methyl-N-(oct-7-yn-1-yl)benzenesulfonamide (11) In a vial add compound **3e** (27.0 mg, 0.075 mmol) , 4-methyl-N-(oct-7-yn-1-yl)benzenesulfonamide (83.8 mg, 0.300 mmol, 4 eq), dry K_2CO_3 (41.5 mg, 0.300 mmol, 4 eq), NaI (2.5 mg, 0.015 mmol, 0.2 eq), tBuOH: toluene (1:1) 0.16 mL each. Close the vial with a cup. Heat it at 90 °C for 72 h. The temperature was further increased to 120 °C and the resultant was heated for another 72h. After 6 days the reaction was cooled to r.t.. The reaction mixture was diluted with MTBE and extracted with HCl (1.0 M). The aqueous phase was neutralized with NaOH 1.0 M, extracted with MTBE, washed with brine and dried over Na_2SO_4. The residue was purified by flash chromatography on silica gel (60/90 petroleum ether/Ethyl acetate 90:10 to 85:15) to provide the desired product as a colorless oil (25.7 mg, 0.042 mmol, 57%). ^{1}H NMR (400 MHz, $CDCl_3$): 7.54 (d, 2H), 7.39 (m, 8H), 7.22 (m, 4H), 2.85 (m, 4H), 2.40 (s, 3H), 2.35 (t, J = 7.8 Hz, 2H), 2.15 (td, 2H), 1.95 (t, J = 2.7 Hz, 1H), 1.45 (m, 4H), 1.25 (m, 4H), 1.11 (m, 2H); ^{19}F NMR (400 MHz, $CDCl_3$): -39.8 (s, 3F); ^{13}C NMR (400 MHz, $CDCl_3$): δ 152.5, 143.0, 140.4, 138.9, 136.7, 129.5, 129.2, 129.1 (q, $^1J_{CF}$ = 310.1 Hz), 128.3 (d), 128.1 (d), 127.7, 127.0, 124.8, 84.4, 68.3, 47.9, 47.7, 33.8, 28.2, 28.2, 28.1, 26.8, 26.0, 21.4, 18.2; HRMS (APCI-TOF) m/z: $[M + H]^+$ Calc'd for $C_{33}H_{37}F_3NO_2S_2$: 600.2213 Found: 600.2221.

(Z)-1-(4'-(2-phenyl-1-((trifluoromethyl)thio)hex-1-en-1-yl)-[1,1'-biphenyl]-4-yl)ethan-1-one (12) In a vial add Pd-G3-Xphos (2.0 mg 0.001 mmol, 3.5 mol%). Then add compound **3k** (33.9 mg, 0.0700 mmol), 4-acetyl phenyl boronic acid (17.5 mg, 0.106 mmol, 1.5 eq), THF (0.14 mL), followed by 0.5M K_3PO_4 solution (30.7 mg, 0.140 mmol, 2 eq) in degassed water (0.28 mL). Heat it at 40 ºC for 2.5h. Cool it to r.t.. Pour the reaction mixture in DI water and extract the organic layer with MTBE and dry over Na_2SO_4. Purify the residue with flash chromatography on silica gel (60/90 petroleum ether/ethyl acetate 97:3 to 92:8) to provide the desired product as a light-yellow oil (29.0 mg, 0.063 mmol, 90%). ^{1}H NMR (400 MHz, $CDCl_3$): 8.05 (d, J = 8.3 Hz, 2H), 7.75 (d, J = 8.1 Hz, 2H), 7.68 (d, J = 8.1 Hz, 2H), 7.52 (d, J = 8.0 Hz, 2H), 7.42 (m, 2H), 7.36 (m, 1H), 7.25 (d, J = 6.9 Hz, 2H), 2.65 (s, 3H), 2.42 (t, J = 7.3 Hz, 2H), 1.26 (m, 2H), 1.16 (m, 2H), 0.73 (t, J = 7.2 Hz, 3H); ^{19}F NMR (400 MHz, $CDCl_3$): -39.9; ^{13}C NMR (400 MHz, $CDCl_3$): δ 197.6, 155.1, 145.0, 140.9, 139.4, 139.1, 136.0, 129.9, 129.2 (q, $^1J_{CF}$ = 310.1 Hz), 128.9, 128.2, 128.0, 127.5, 127.1, 127.0, 123.2, 36.6, 30.1, 26.6, 22.3, 13.6; HRMS (EI-EBE) m/z: $[M]^+$ Calc'd for $C_{27}H_{25}F_3OS$: 454.1578 Found: 454.1566.

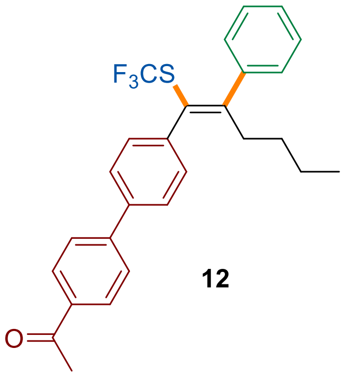

12

Copies of ^{1}H, ^{19}F and $^{13}C\{^{1}H\}$ NMR spectra of isolated compounds

1-(dimethoxymethyl)-4-(hex-1-yn-1-yl)benzene (400 MHz [^{1}H];101 MHz [^{13}C], $CDCl_3$)

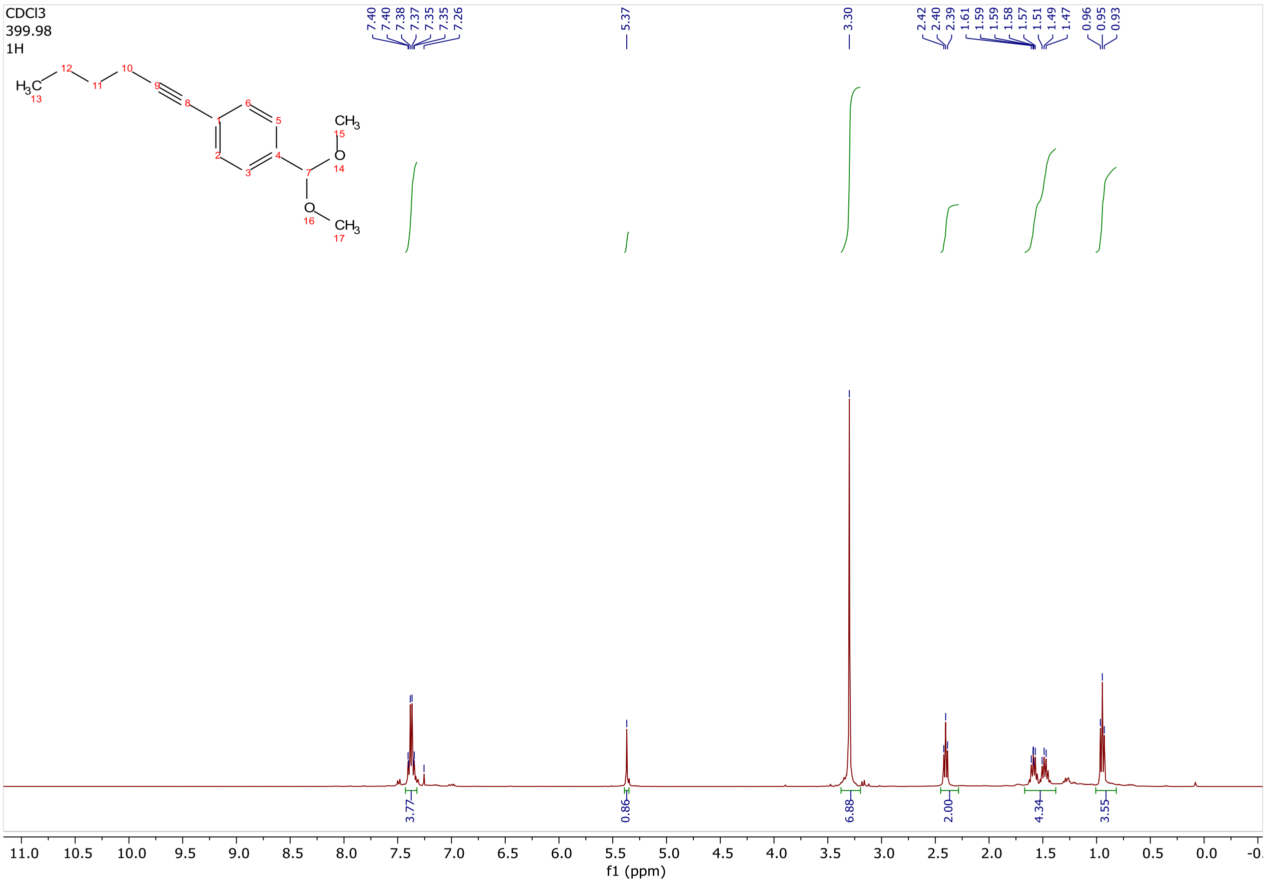

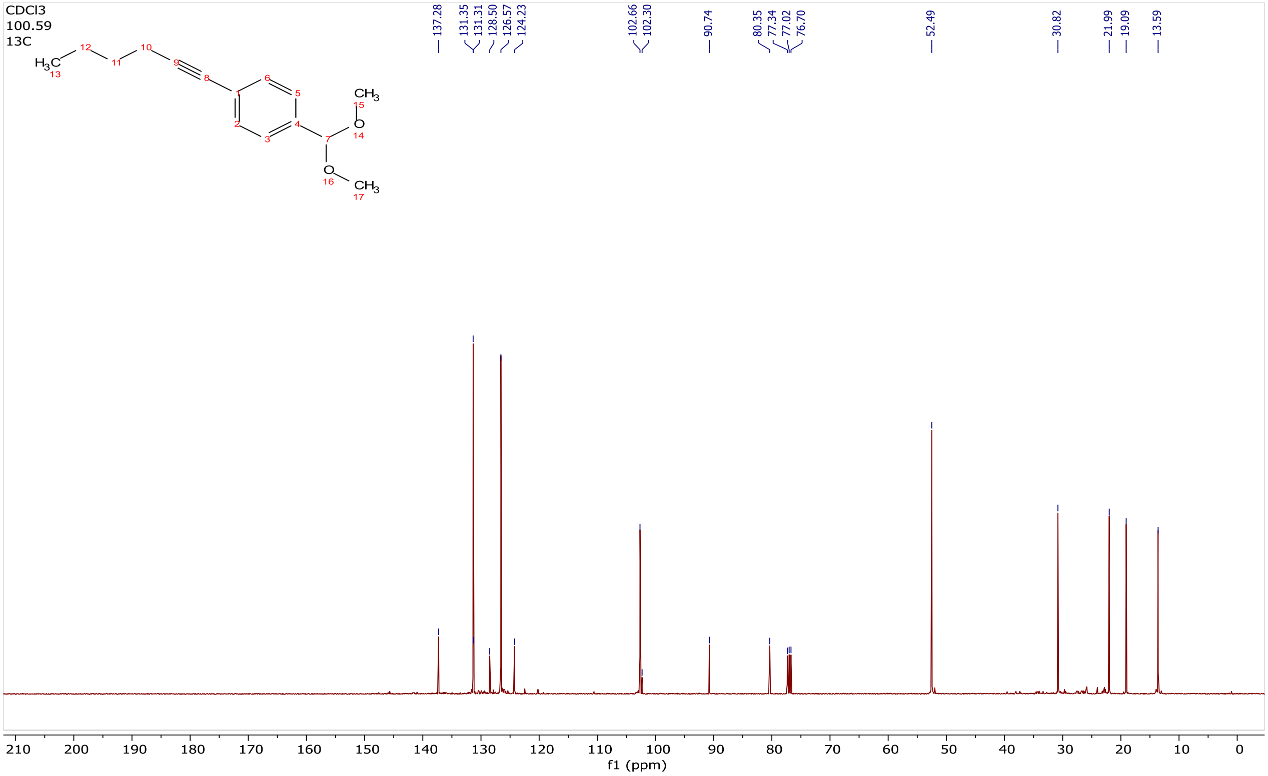

4-(hex-1-yn-1-yl)phenyl triflate (400 MHz [^{1}H];101 MHz [^{13}C], 376 MHz [^{19}F] $CDCl_3$)

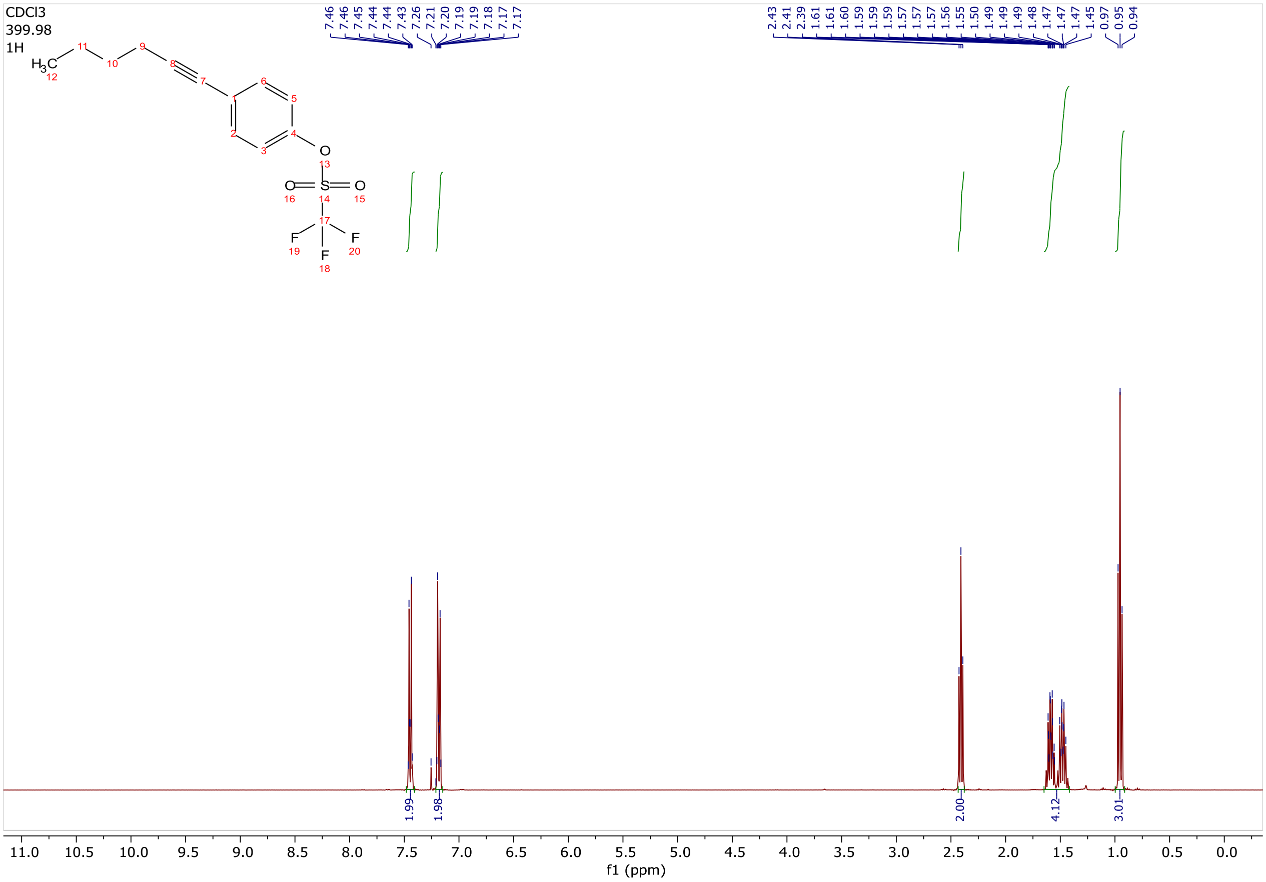

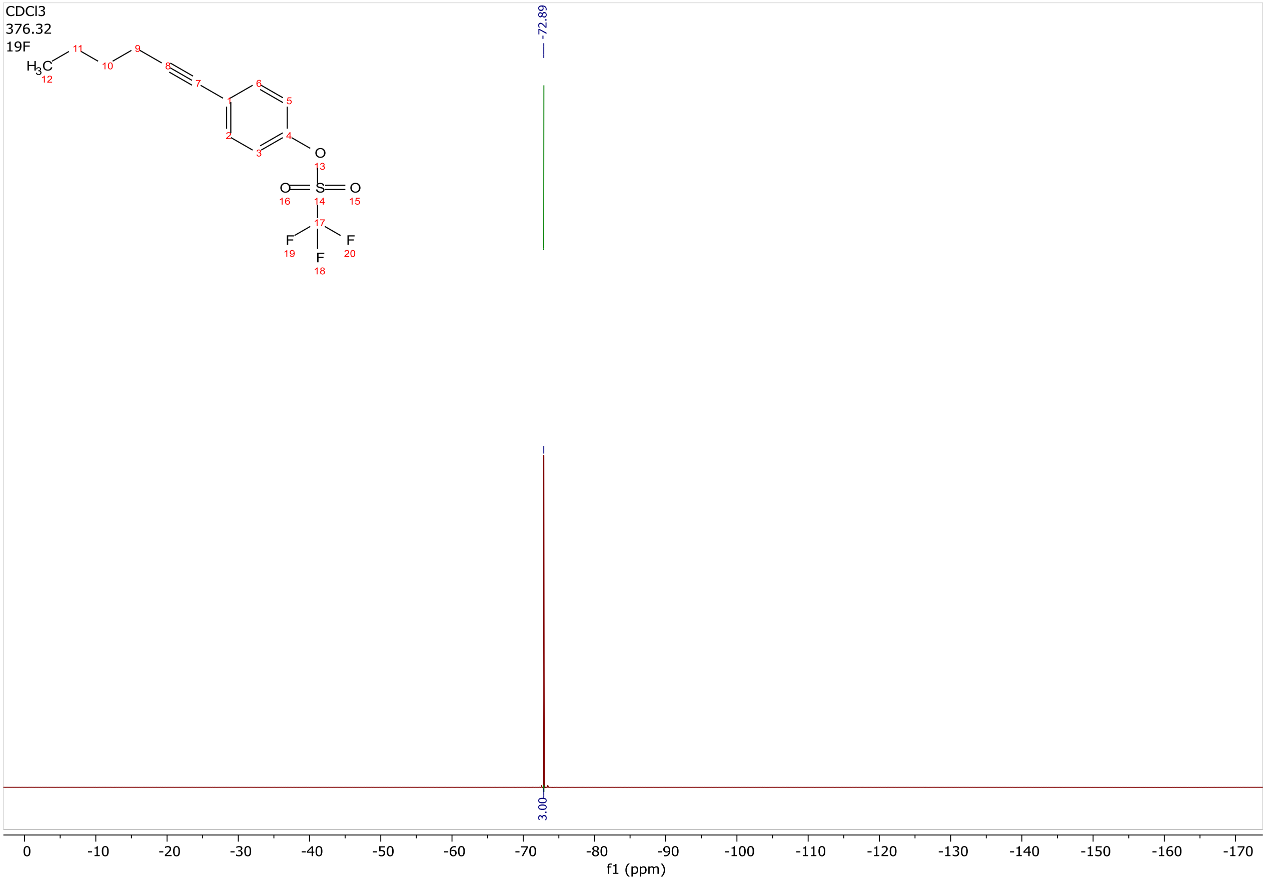

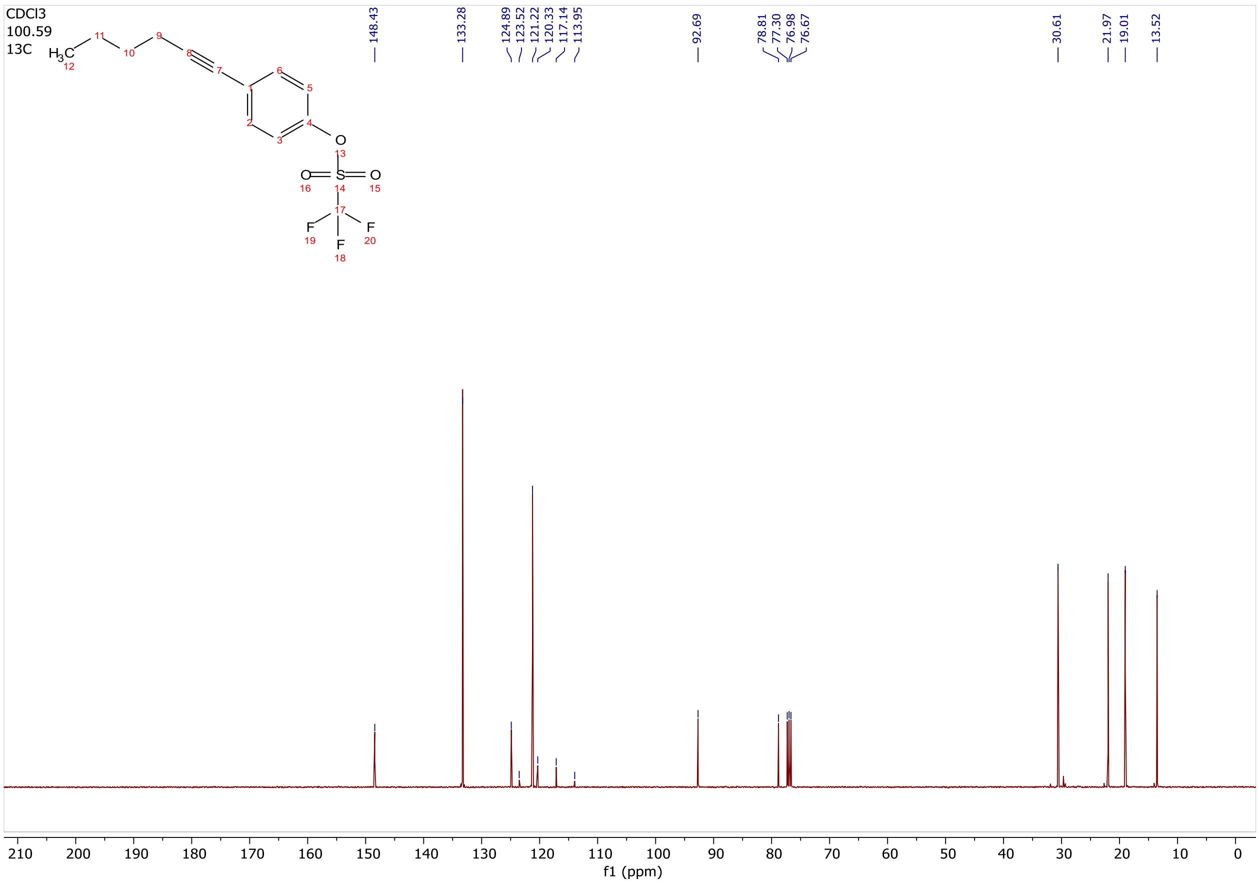
CDCl3
100.59
13C
148.43
133.28
124.89
123.52
121.22
120.33
117.14
113.95
92.69
78.81
77.30
76.98
76.67
30.61
21.97
19.01
13.52
f1 (ppm)

(Z)-(1,2-diphenylhex-1-en-1-yl)(trifluoromethyl)sulfane (3a) (400 MHz [^{1}H];101 MHz [^{13}C], 376 MHz [^{19}F] $CDCl_3$)

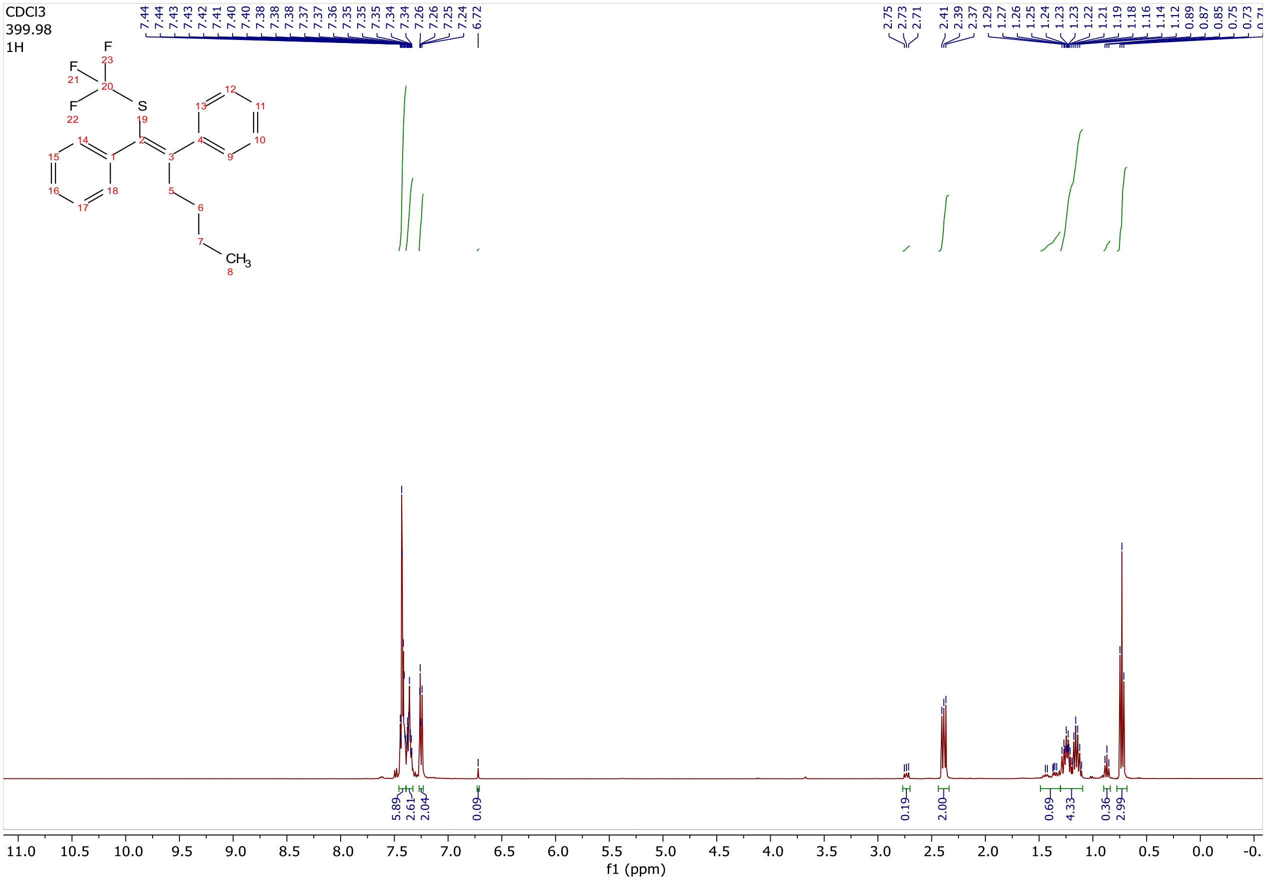

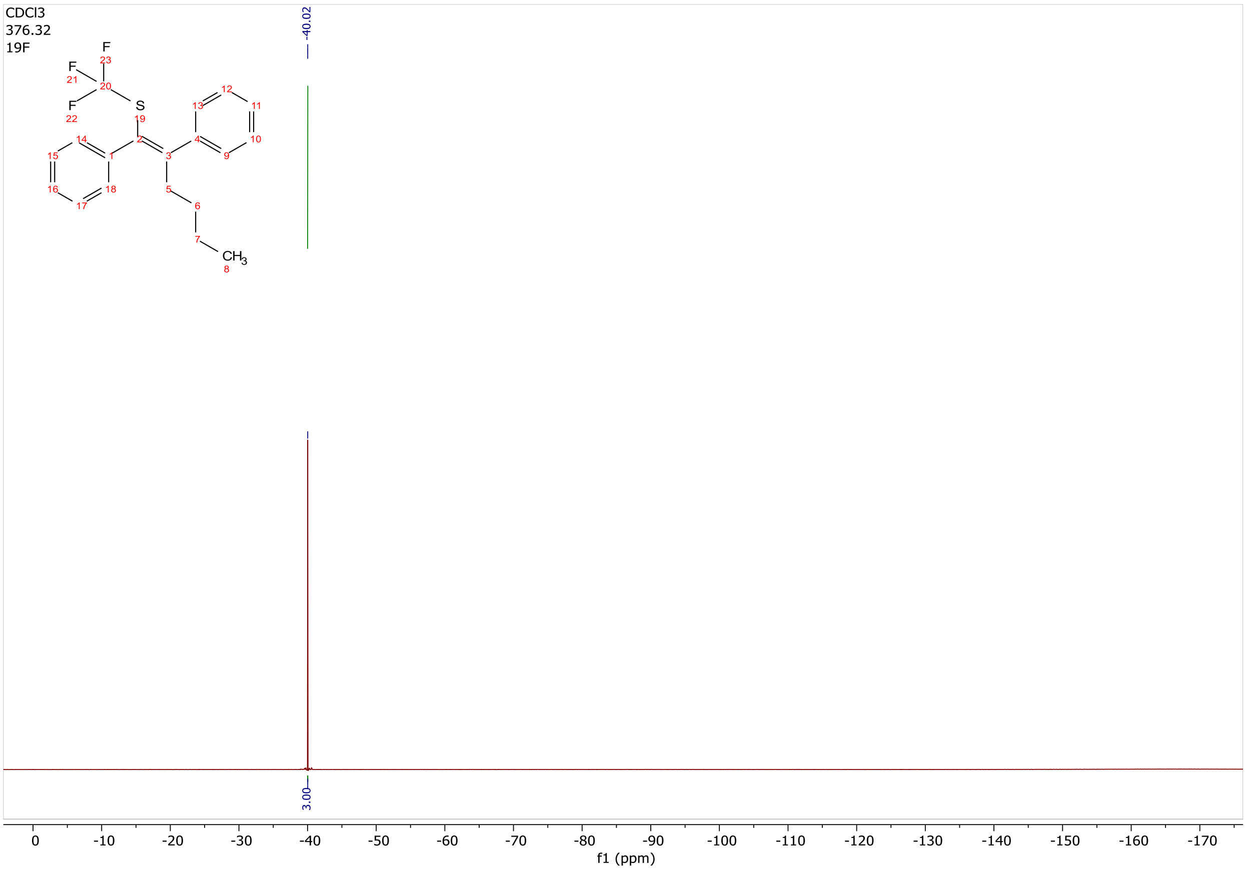

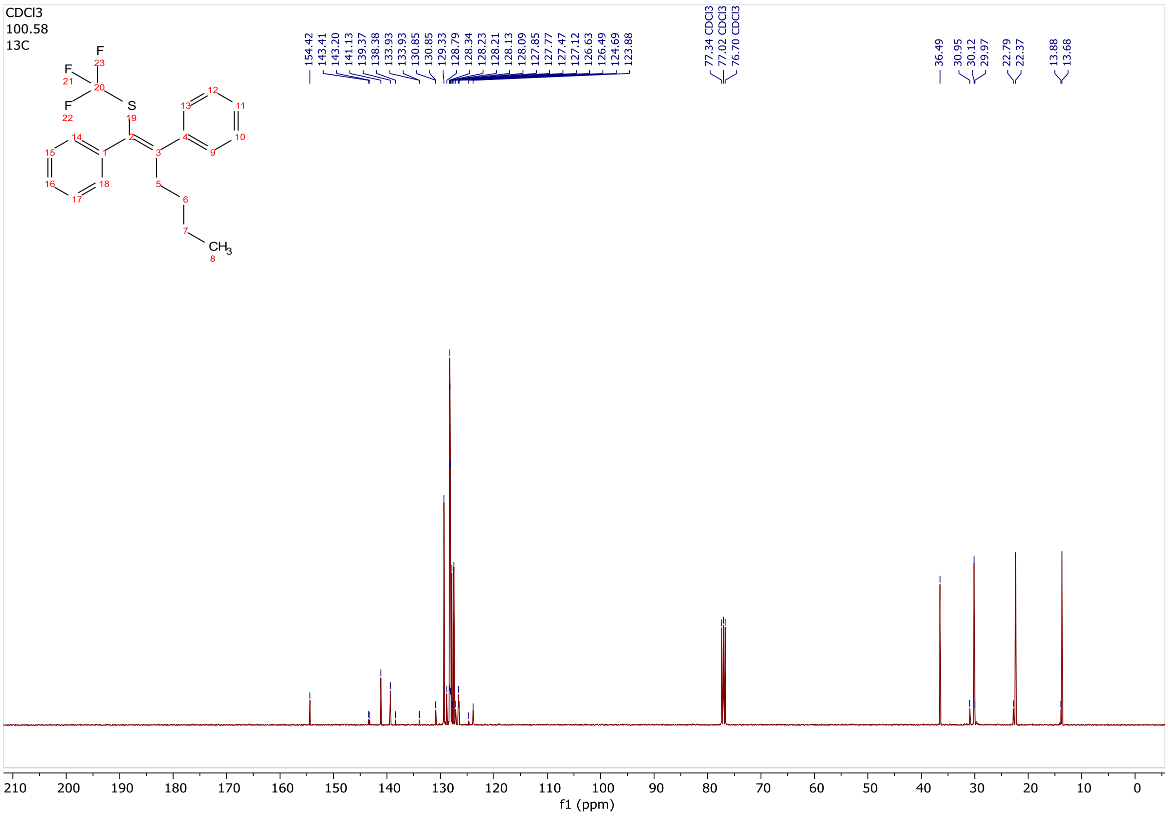
CDCl3
100.58
13C
154.42
143.41
143.20
141.13
139.37
138.38
133.93
133.93
130.85
130.85
129.33
128.79
128.34
128.23
128.21
128.13
128.09
127.85
127.77
127.47
127.12
126.63
126.49
124.69
123.88
77.34 CDCl3
77.02 CDCl3
76.70 CDCl3
36.49
30.95
30.12
29.97
22.79
22.37
13.88
13.68
f1 (ppm)

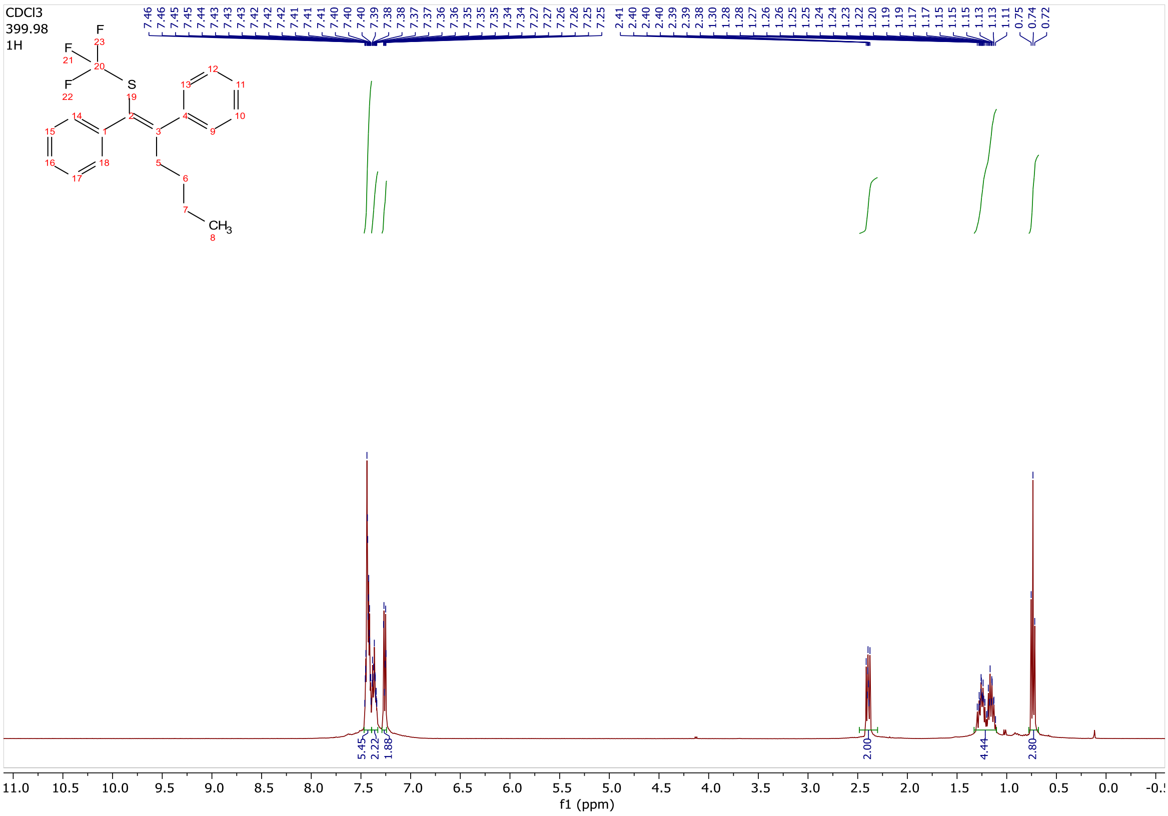
CDCl3
399.98
1H
f1 (ppm)

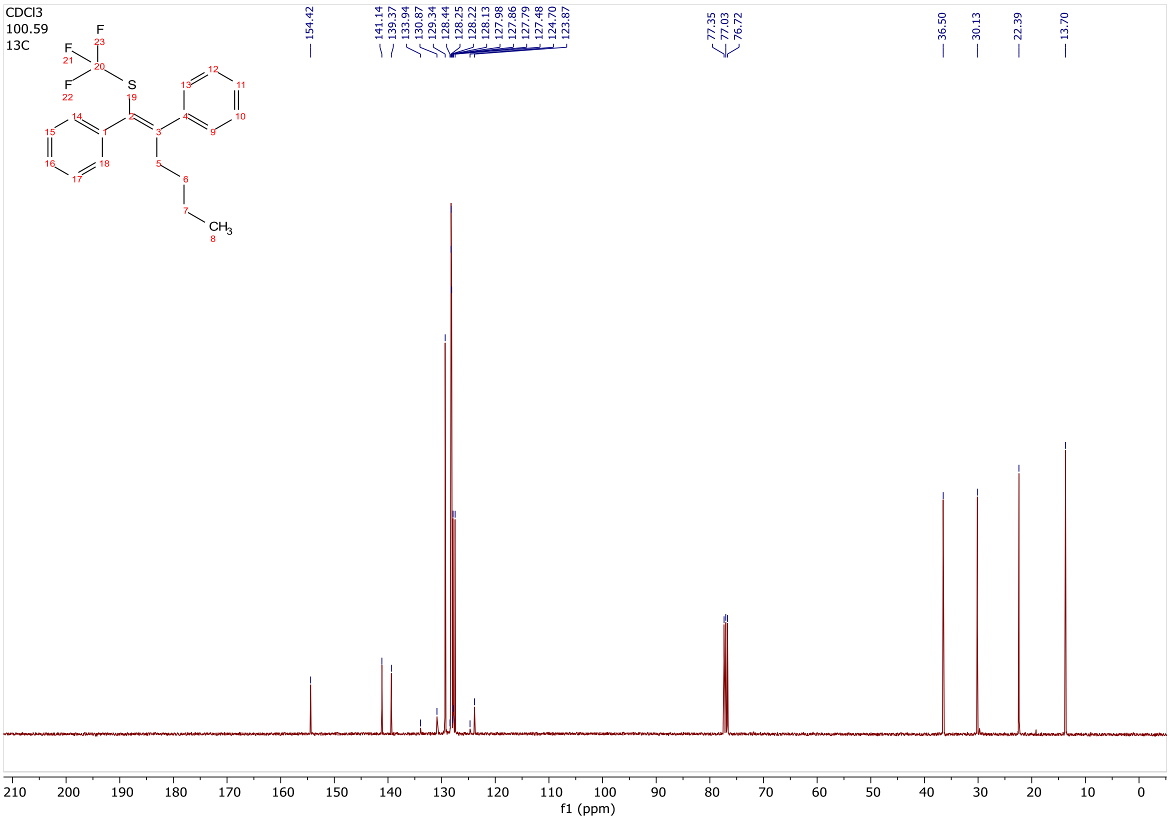
CDCl3
100.59
13C
f1 (ppm)

(*Z*)-(1,2-diphenylprop-1-en-1-yl)trifluoromethyl)sulfane (3b) (400 MHz [^{1}H];101 MHz [^{13}C], 376 MHz [^{19}F] $CDCl_3$)

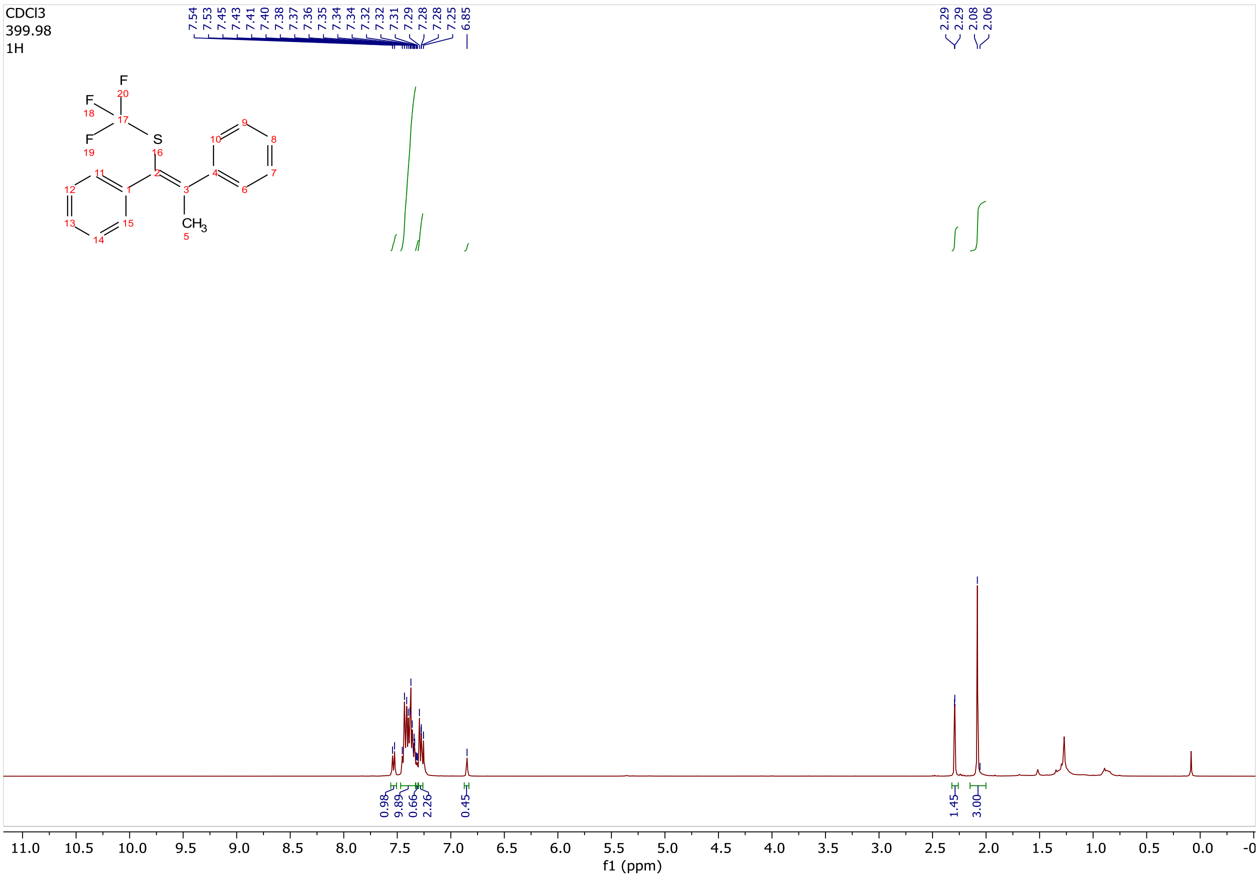

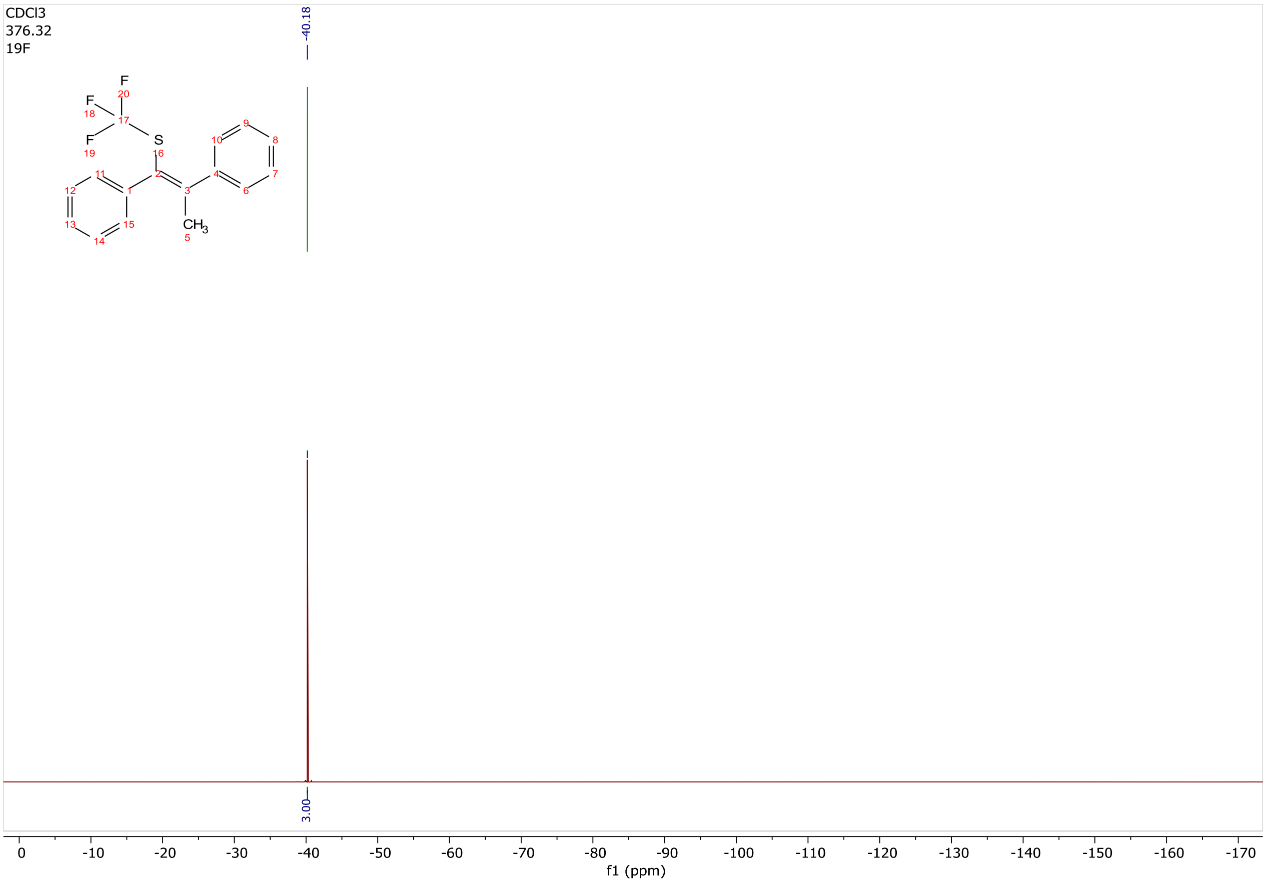

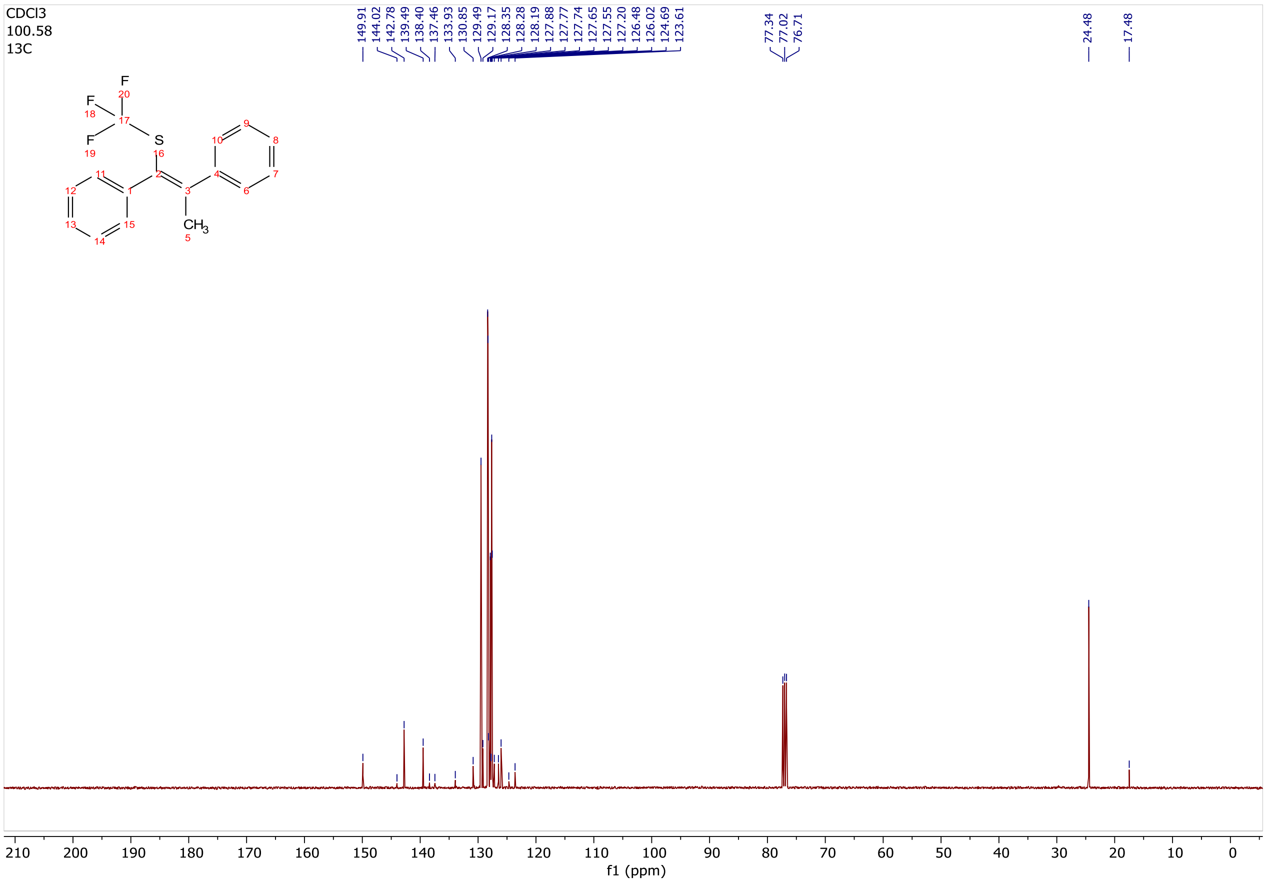
CDCl3
100.58
13C
149.91
144.02
142.78
139.49
138.40
137.46
133.93
130.85
129.49
129.17
128.35
128.28
128.19
127.88
127.77
127.74
127.65
127.55
127.20
126.48
126.02
124.69
123.61
77.34
77.02
76.71
24.48
17.48
f1 (ppm)

(*Z*)-(2-cyclopropyl-1,2-diphenylvinyl)trifluoromethyl)sulfane (3c) (400 MHz [^{1}H];101 MHz [^{13}C], 376 MHz [^{19}F] $CDCl_3$)

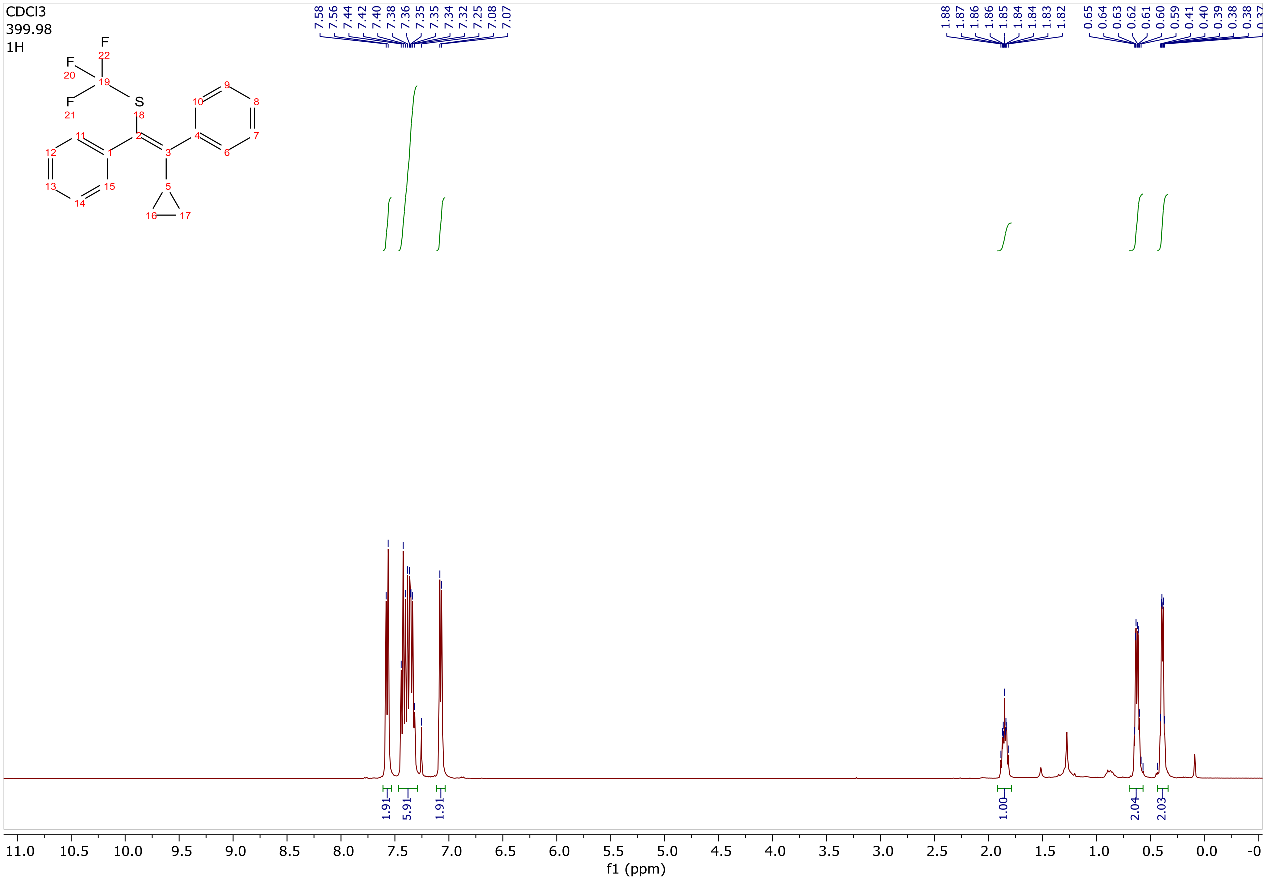

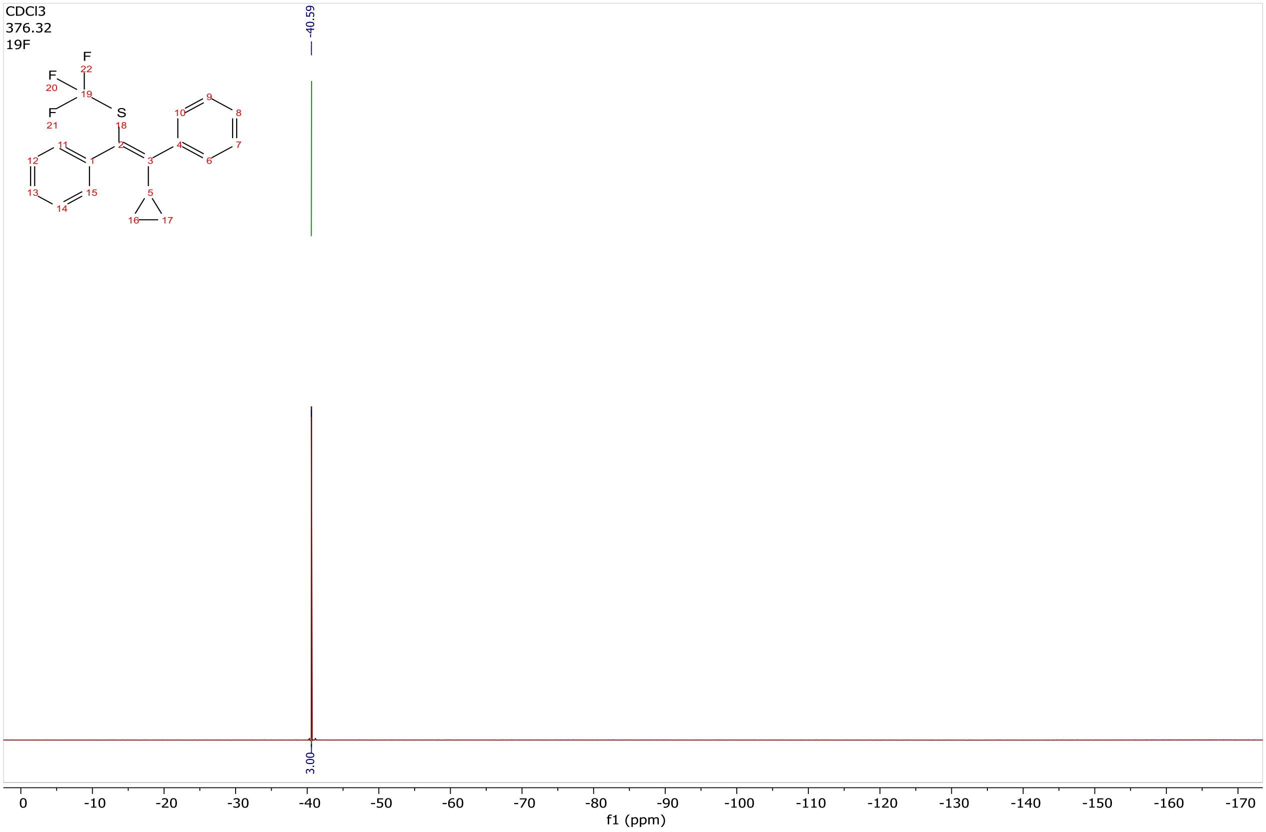

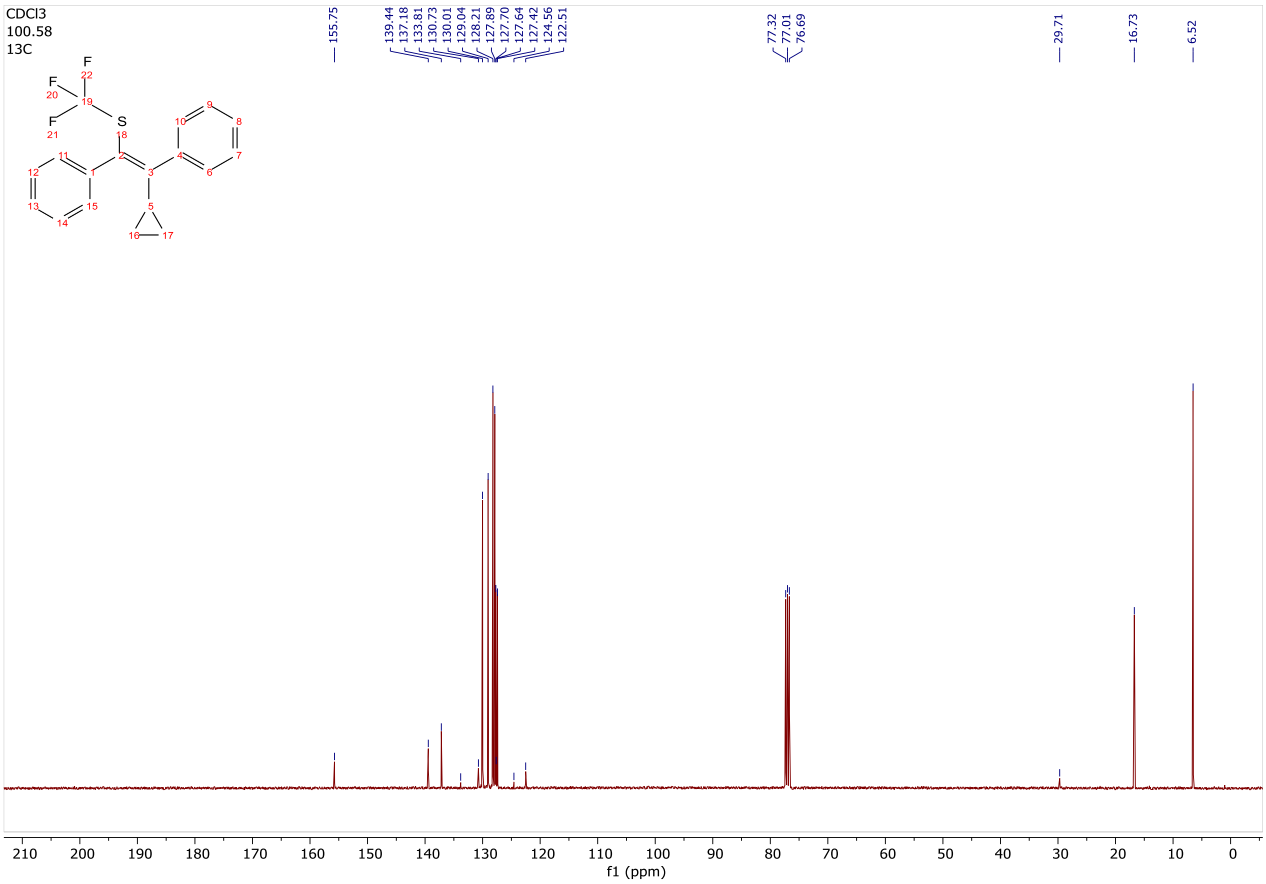
CDCl3
100.58
13C
155.75
139.44
137.18
133.81
130.73
130.01
129.04
128.21
127.89
127.70
127.64
127.42
124.56
122.51
77.32
77.01
76.69
29.71
16.73
6.52
f1 (ppm)

(*Z*)-(3-methyl-1,2-diphenylbut-1-en-1-yl)trifluoromethyl)sulfane (3d) (400 MHz [^{1}H];101 MHz [^{13}C], 376 MHz [^{19}F] $CDCl_3$)

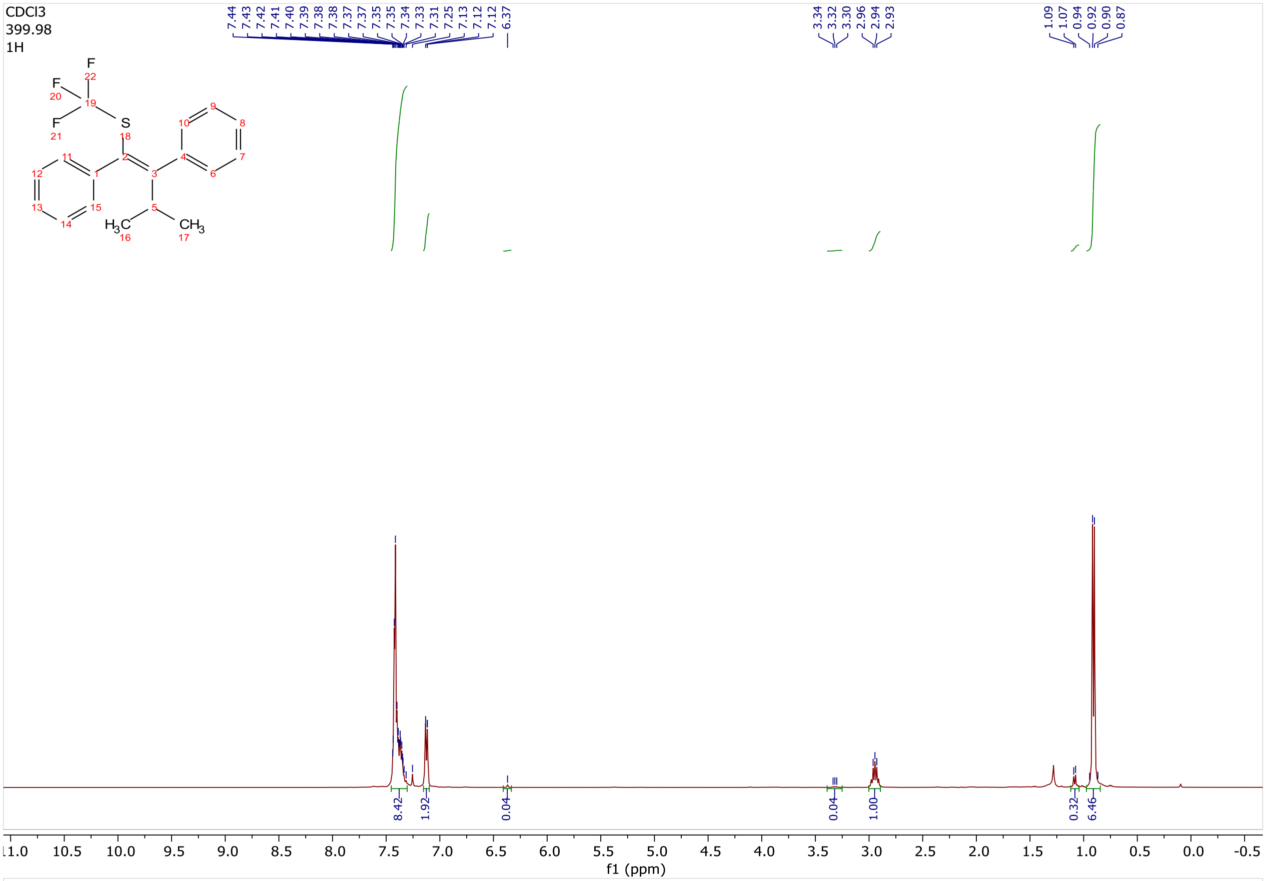

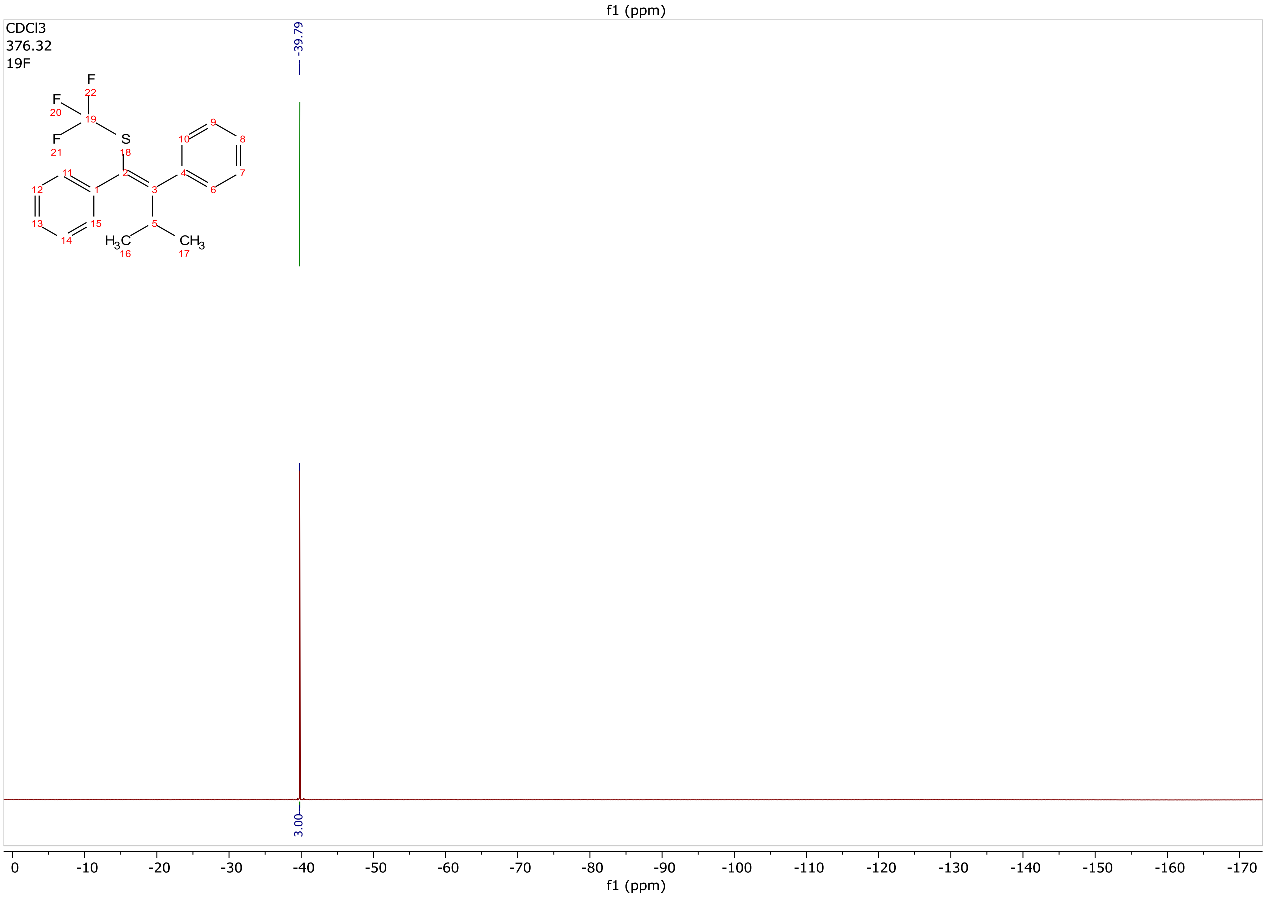

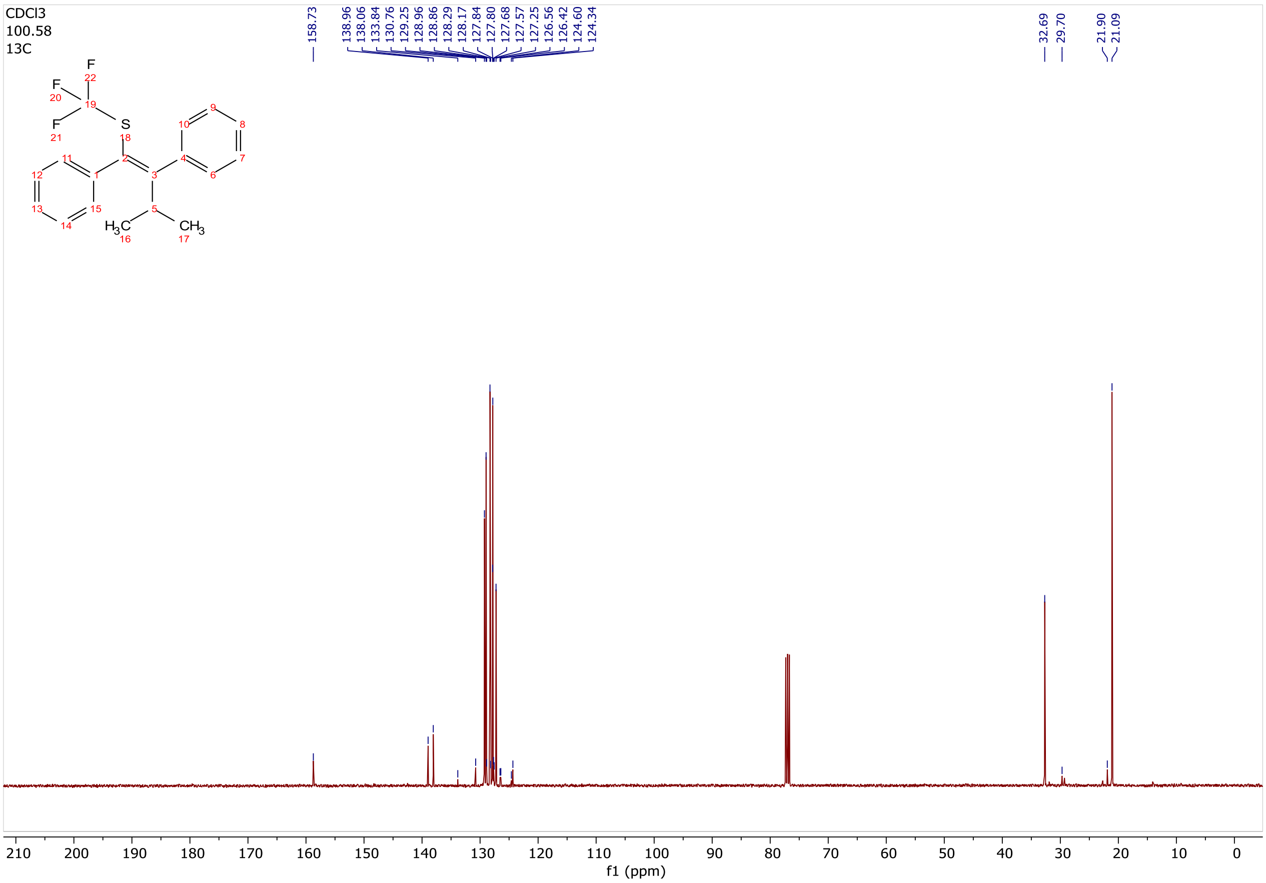
CDCl3
100.58
13C
158.73
138.96
138.06
133.84
130.76
129.25
128.96
128.86
128.29
128.17
127.84
127.80
127.68
127.57
127.25
126.56
126.42
124.60
124.34
32.69
29.70
21.90
21.09
210 200 190 180 170 160 150 140 130 120 110 100 90 80 70 60 50 40 30 20 10 0
f1 (ppm)

(*Z*)-(5-chloro-1,2-diphenylpent-1-en-1-yl))trifluoromethyl)sulfane (3e) (400 MHz [^{1}H];101 MHz [^{13}C], 376 MHz [^{19}F] $CDCl_3$)

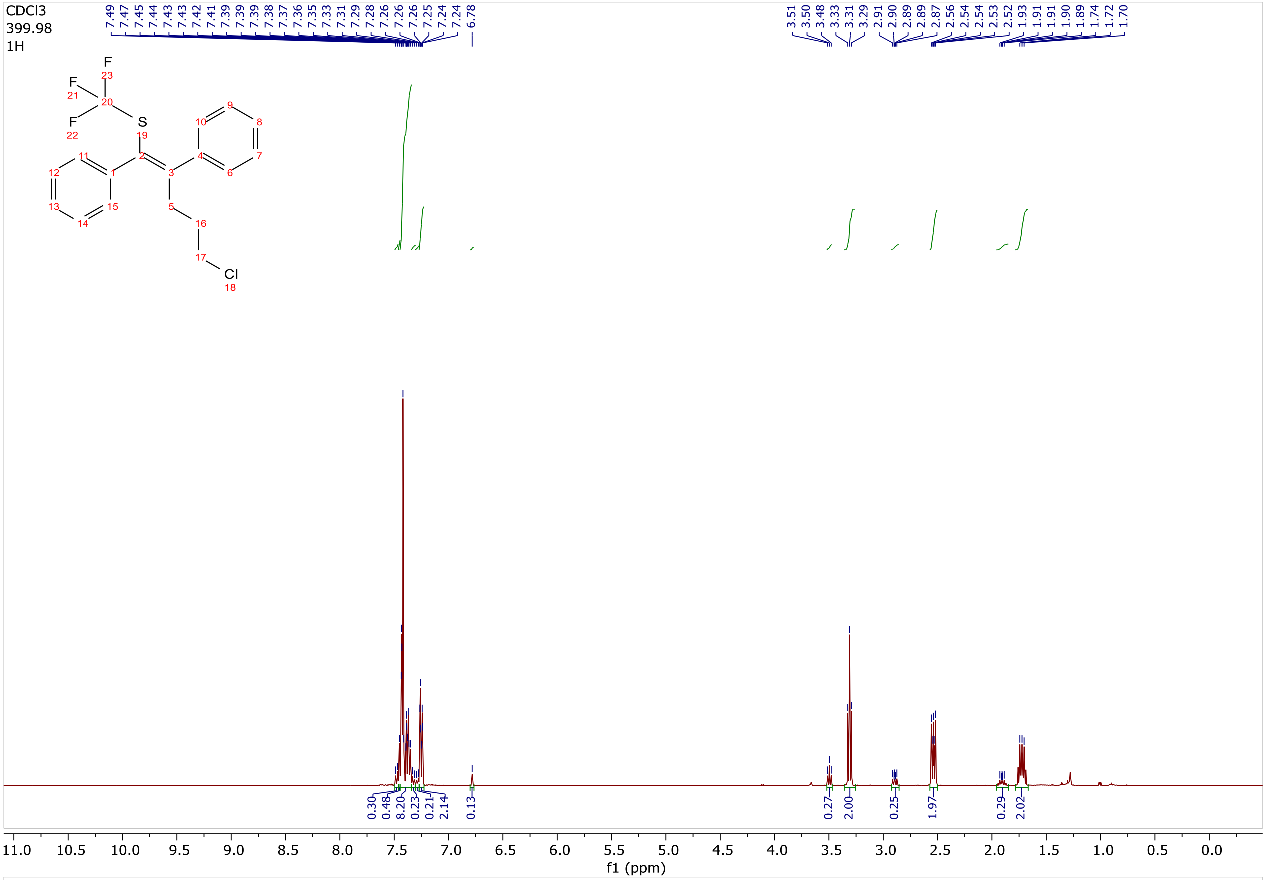

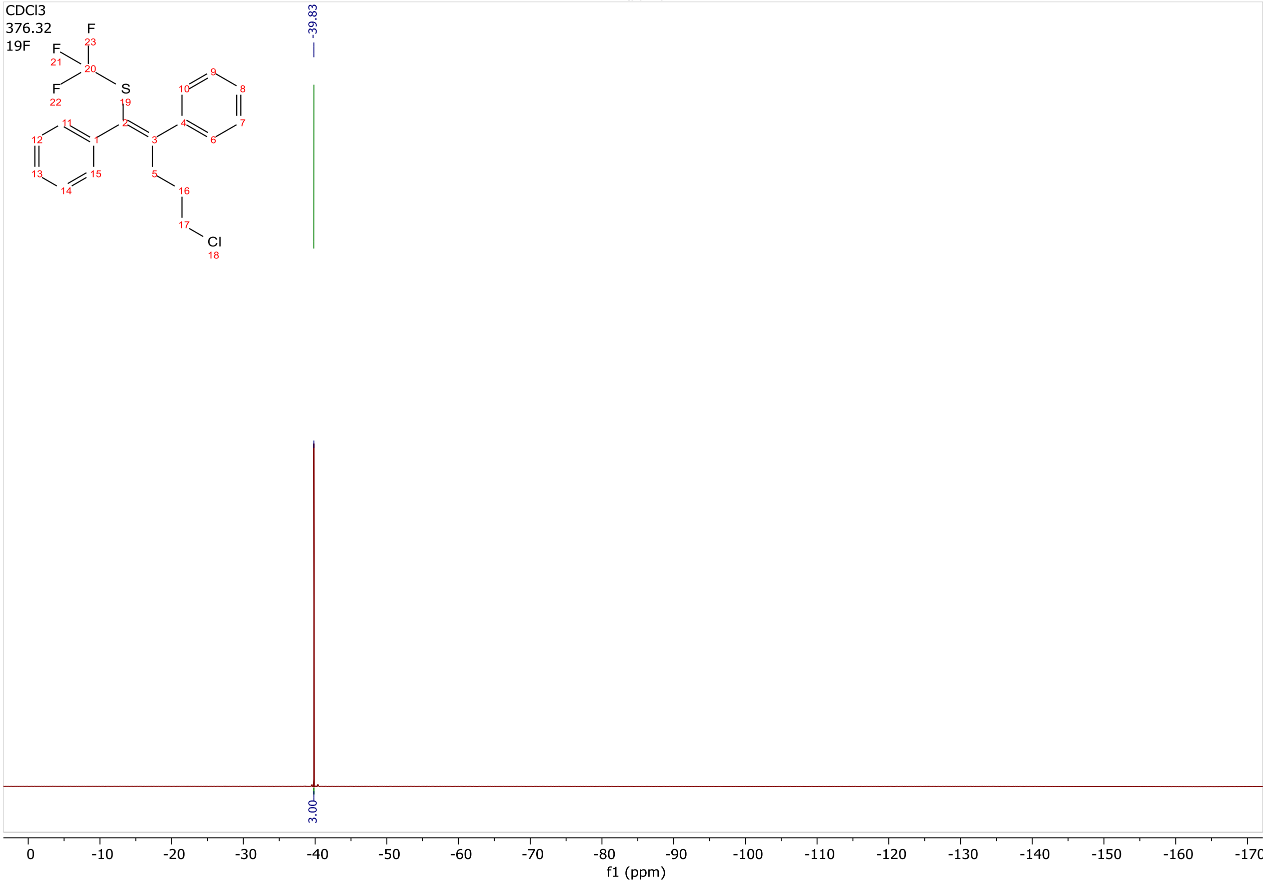

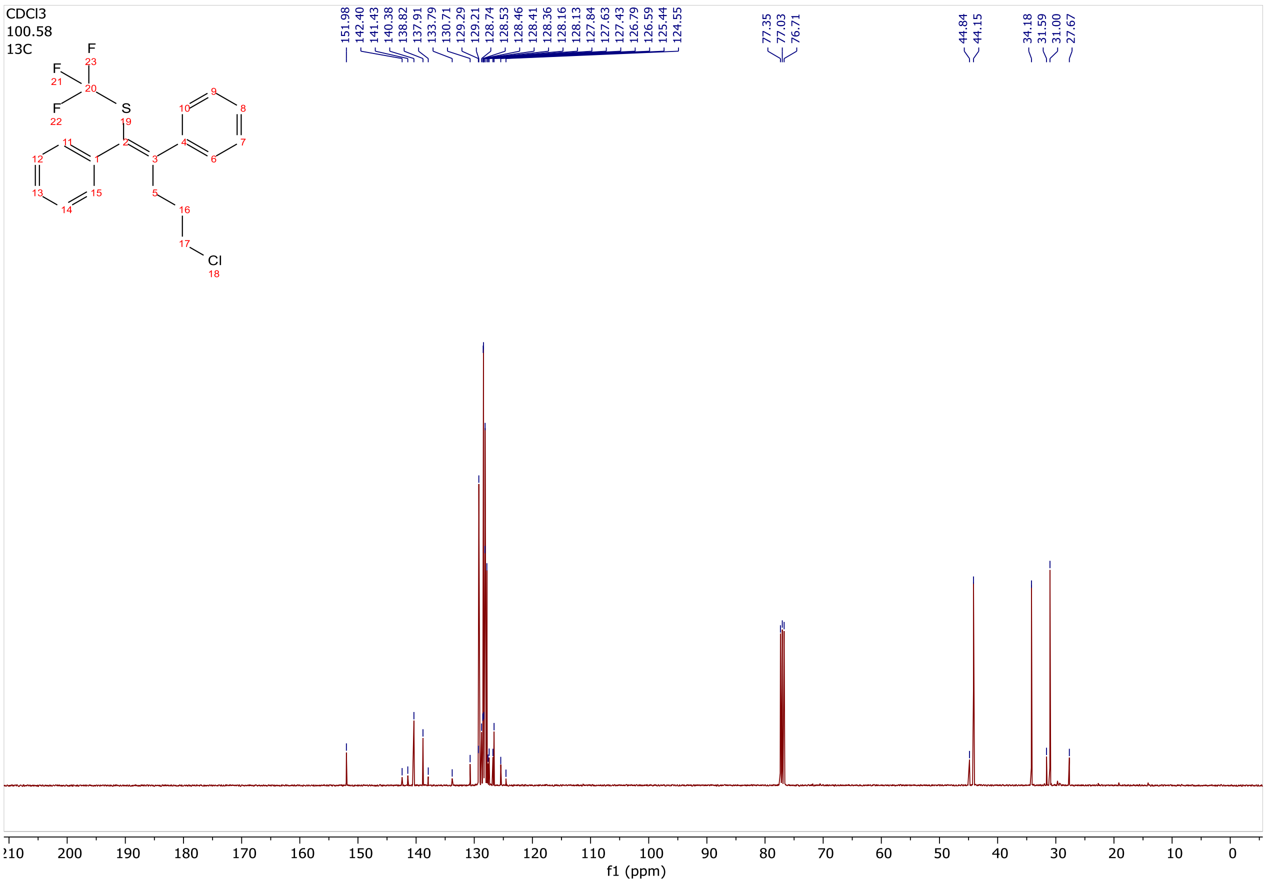
CDCl3
100.58
13C
151.98 142.40 141.43 140.38 138.82 137.91 133.79 130.71 129.29 129.21 128.74 128.53 128.46 128.41 128.36 128.16 128.13 127.84 127.63 127.43 126.79 126.59 125.44 124.55
77.35 77.03 76.71
44.84 44.15
34.18 31.59 31.00 27.67
f1 (ppm)

(Z)-(trifluoromethyl)(1,2,3-triphenylprop-1-en-1-yl)sulfane (3f) (400 MHz [^{1}H];101 MHz [^{13}C], 376 MHz [^{19}F] $CDCl_3$)

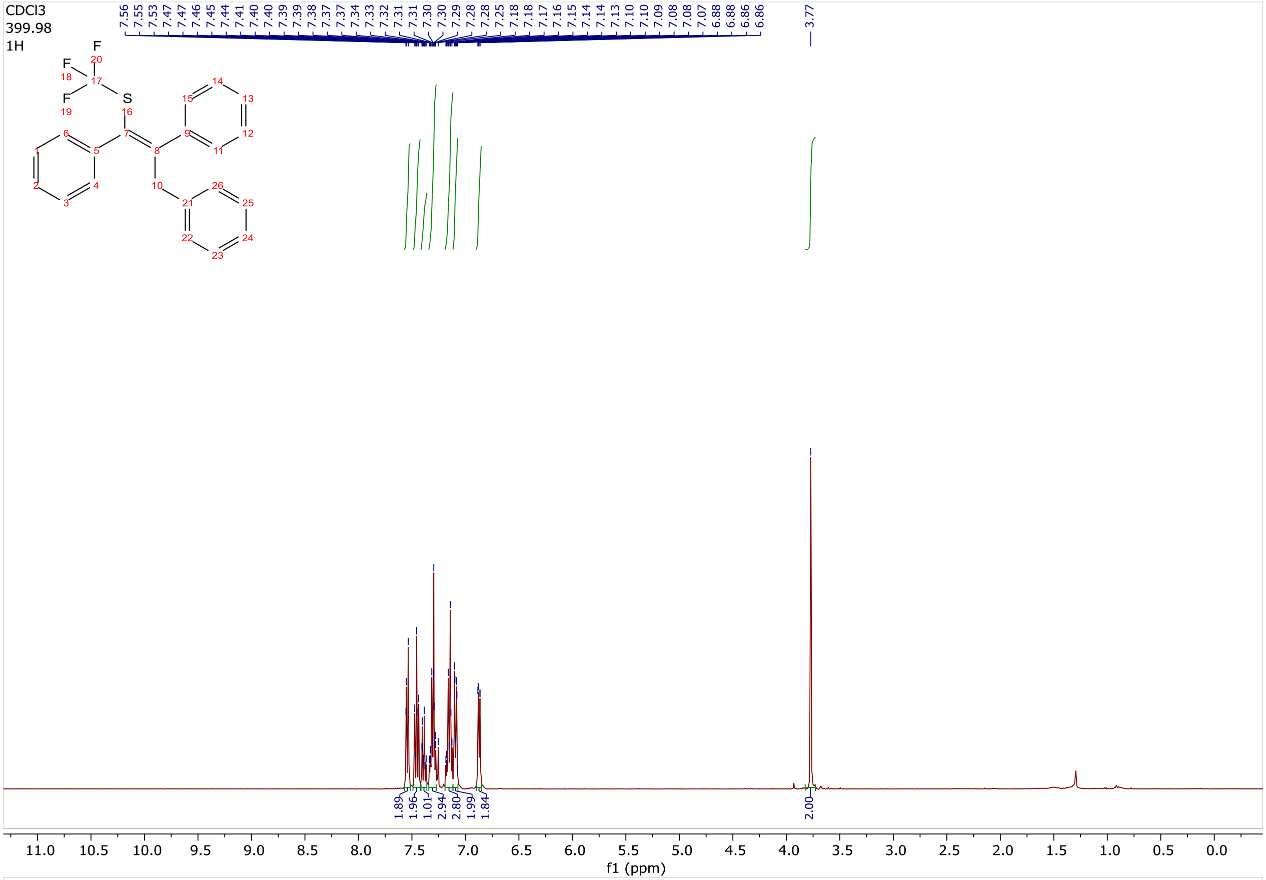

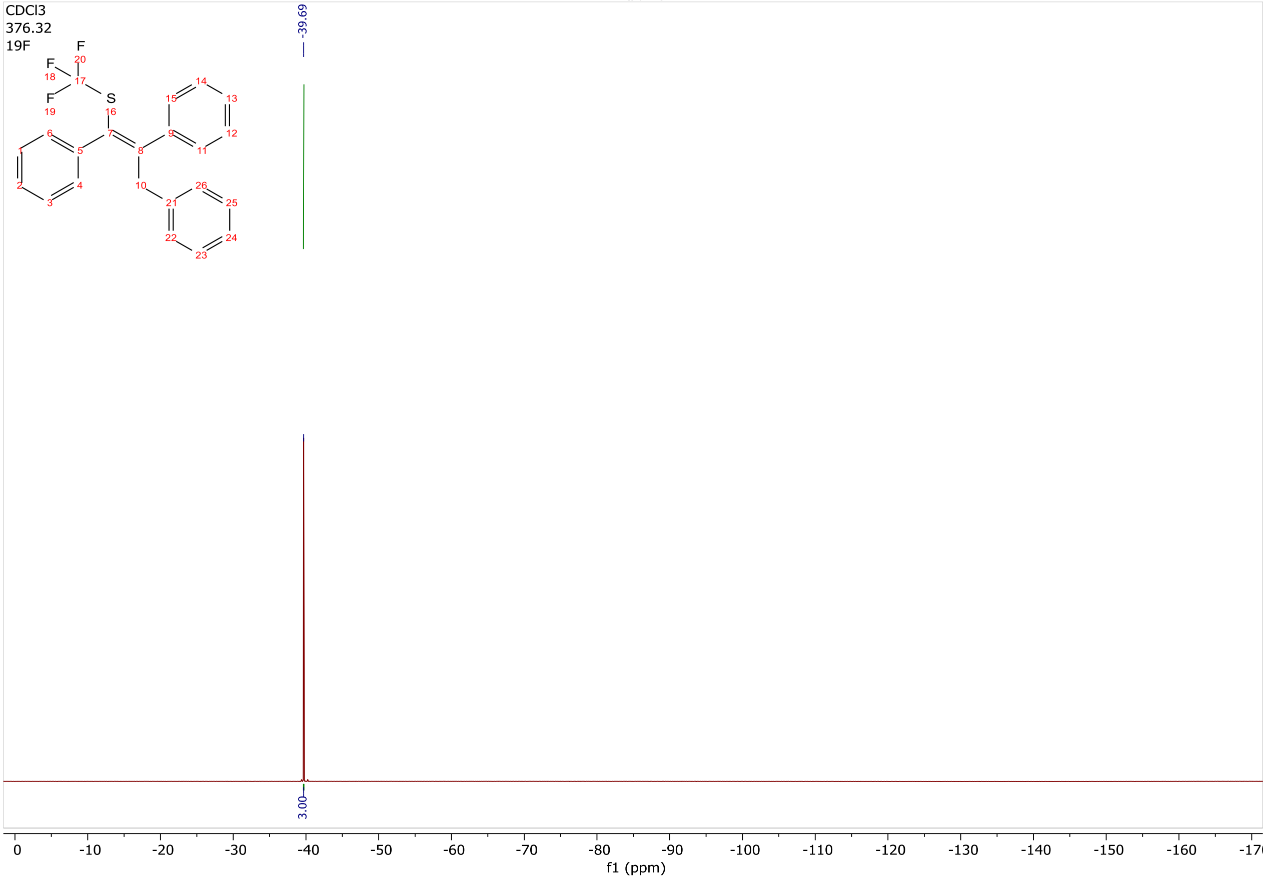

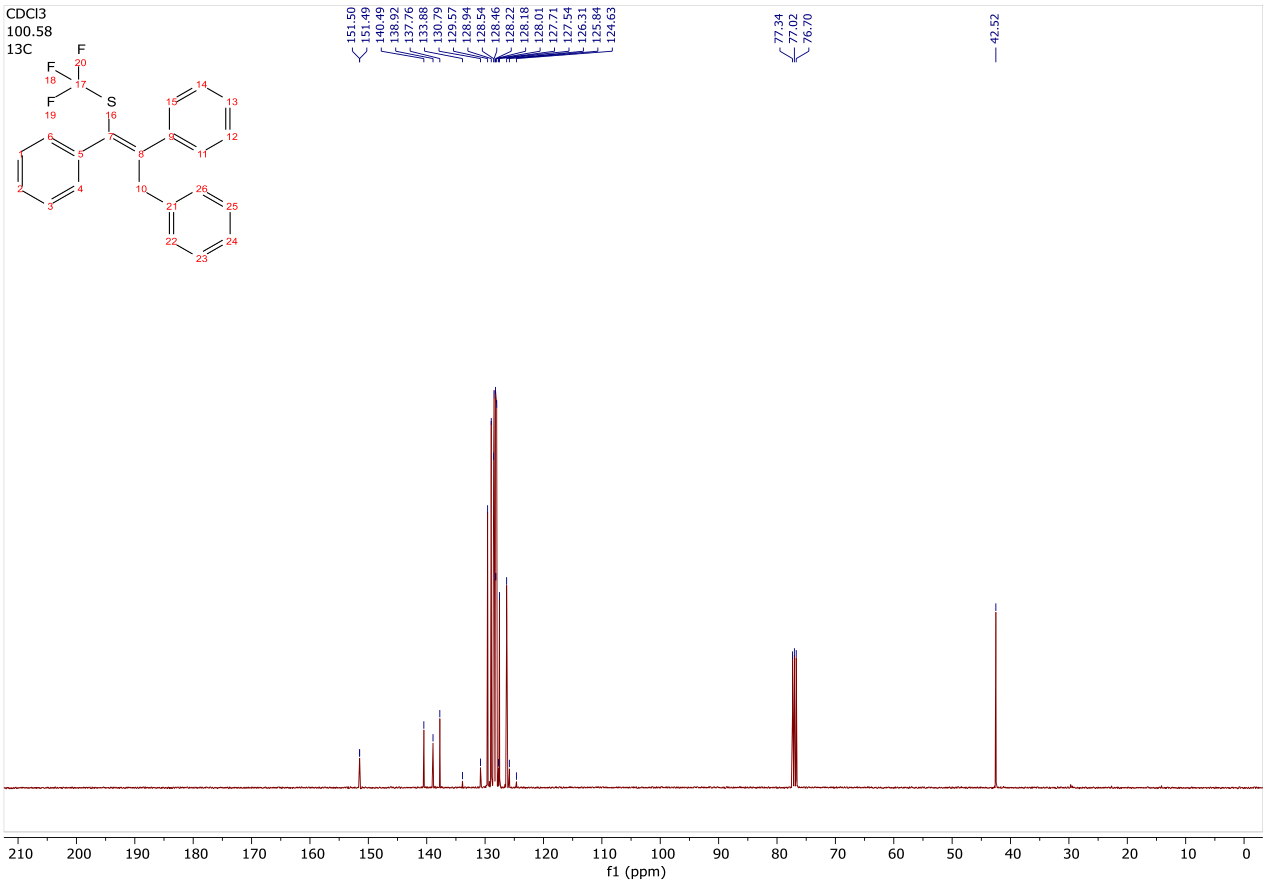
CDCl3
100.58
13C
151.50
151.49
140.49
138.92
137.76
133.88
130.79
129.57
128.94
128.54
128.46
128.22
128.18
128.01
127.71
127.54
126.31
125.84
124.63
77.34
77.02
76.70
42.52
f1 (ppm)

(*Z*)-(1-(4-methoxyphenyl)-2-phenylhex-1-en-1-yl))trifluoromethyl)sulfane (3g) (400 MHz [^{1}H];101 MHz [^{13}C], 376 MHz [^{19}F] $CDCl_3$)

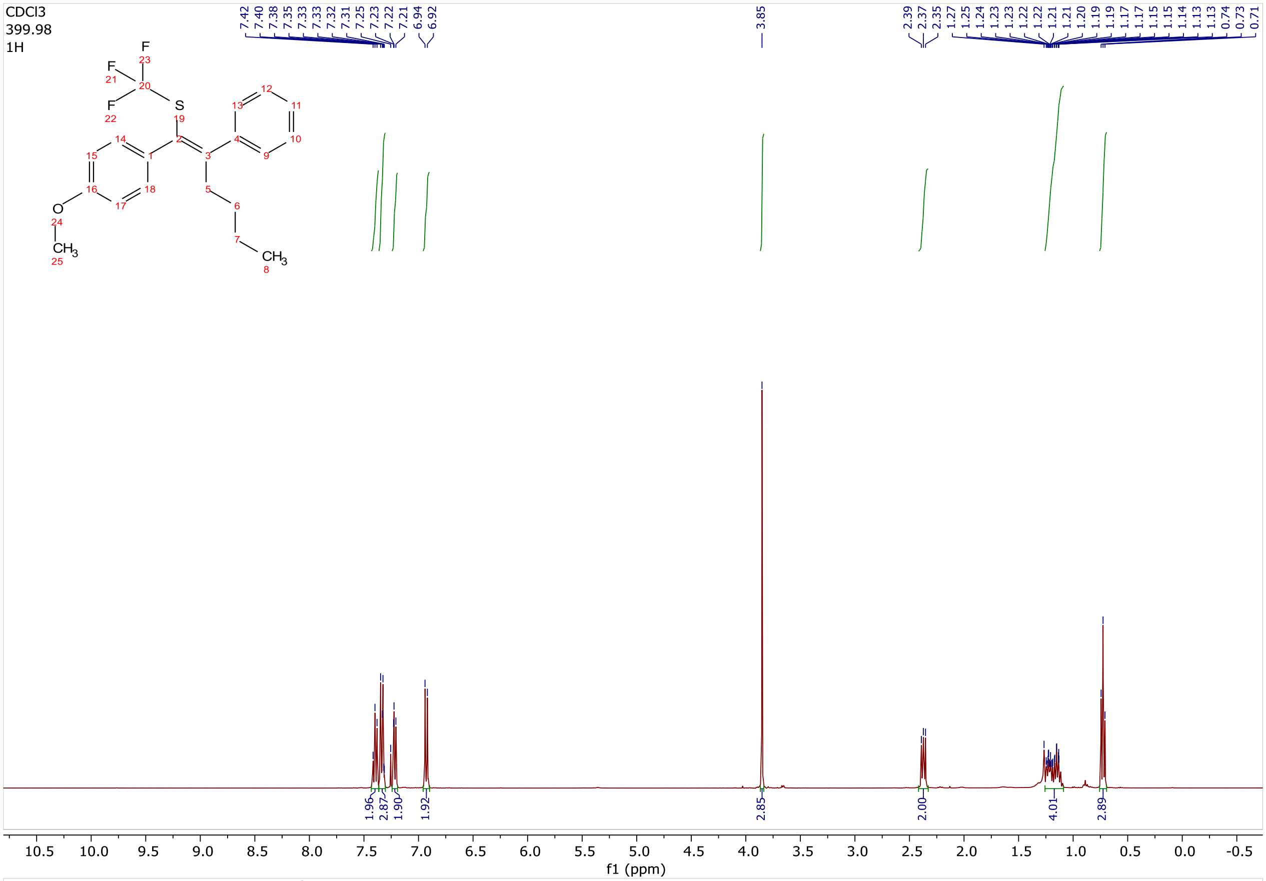

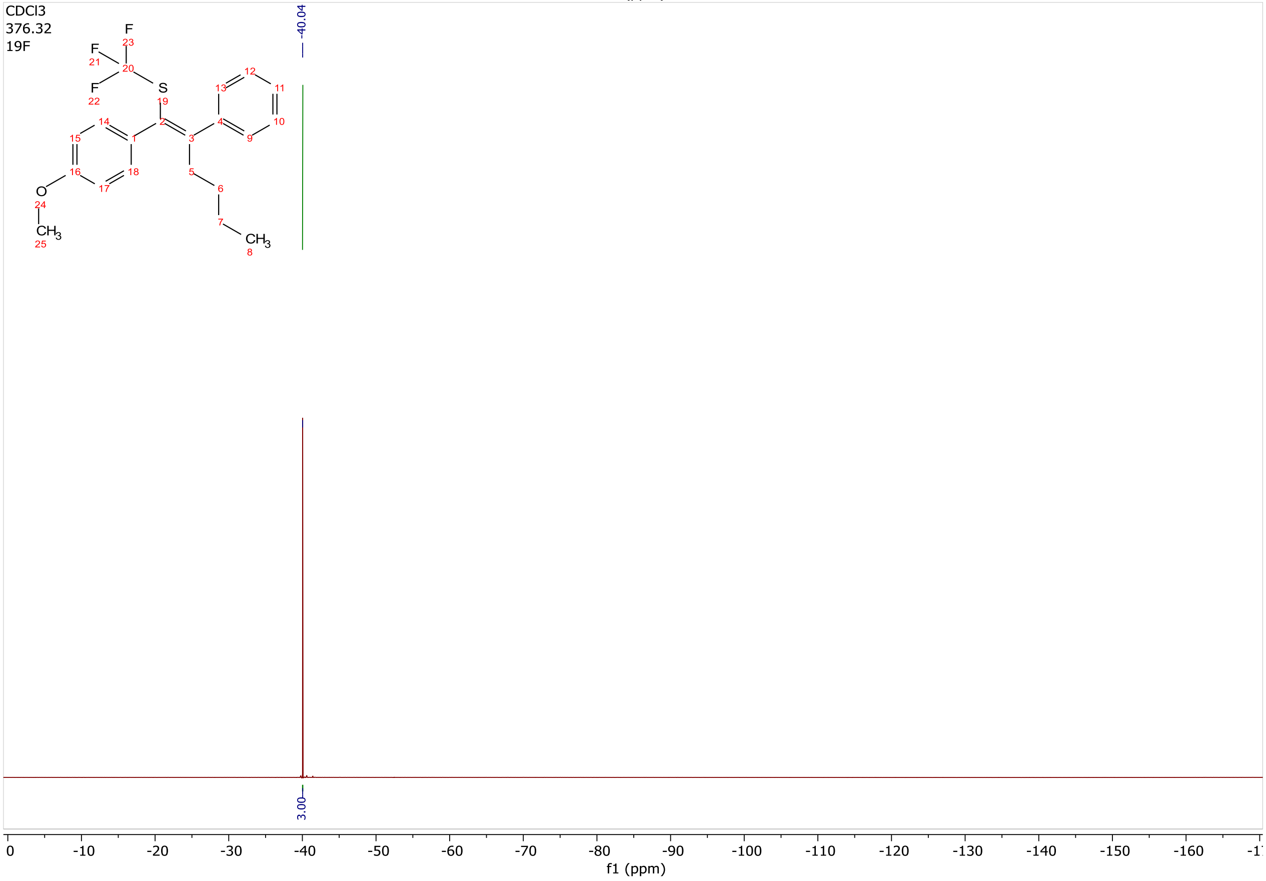

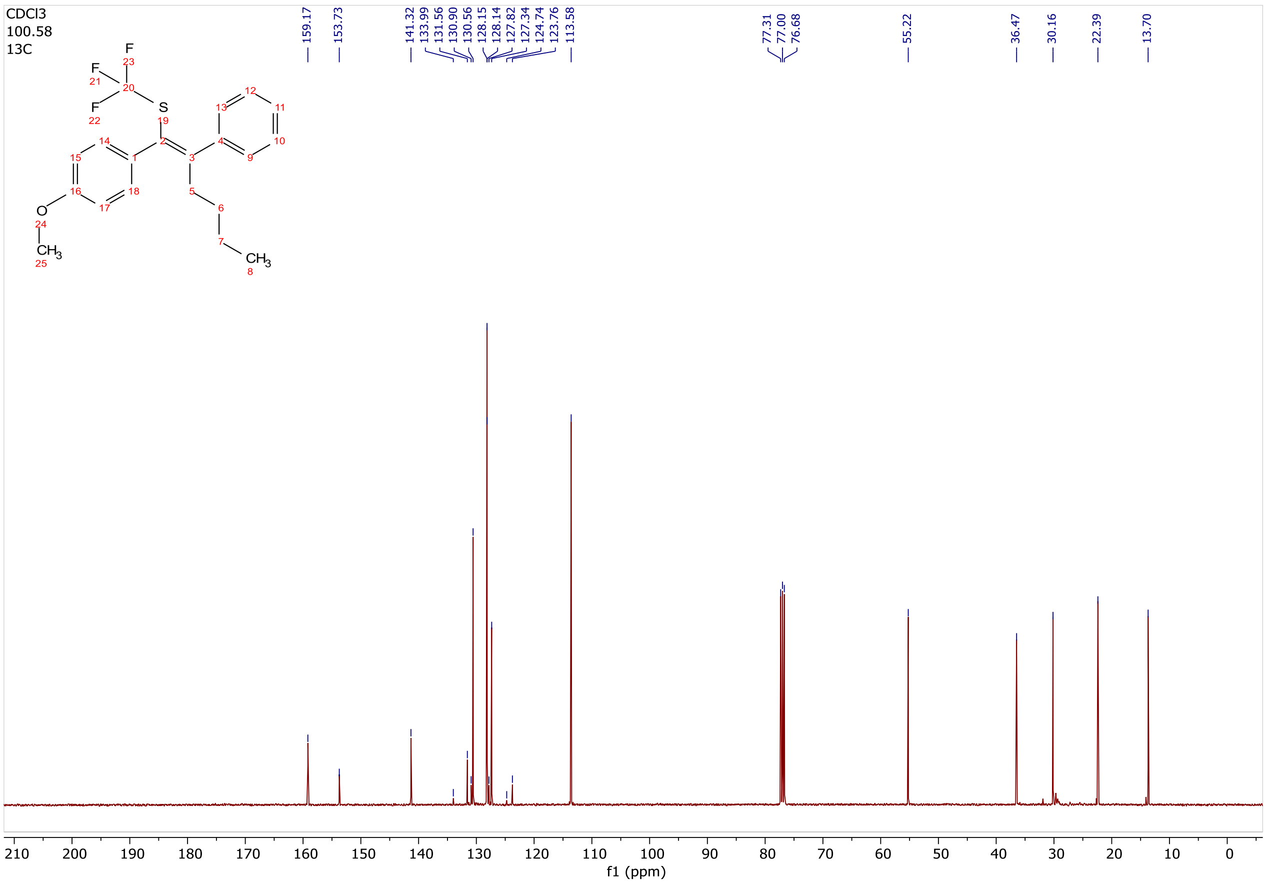

CDCl3
100.58
13C
159.17
153.73
141.32
133.99
131.56
130.90
130.56
128.15
128.14
127.82
127.34
124.74
123.76
113.58
77.31
77.00
76.68
55.22
36.47
30.16
22.39
13.70
f1 (ppm)

(*Z*)-(1-(naphthalen-1-yl)-2-phenylhex-1-en-1-yl))trifluoromethyl)sulfane (3h) (400 MHz [^{1}H];101 MHz [^{13}C], 376 MHz [^{19}F] $CDCl_3$)

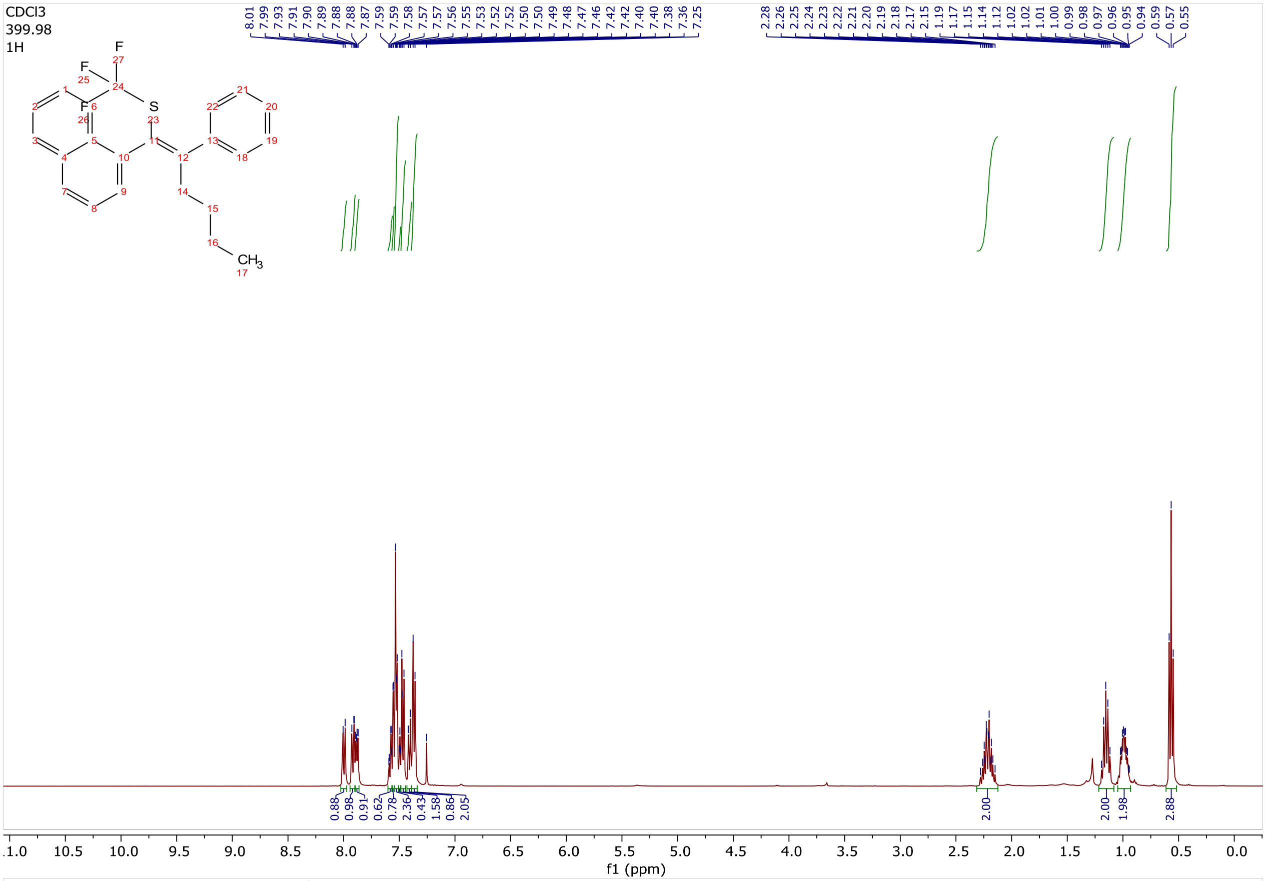

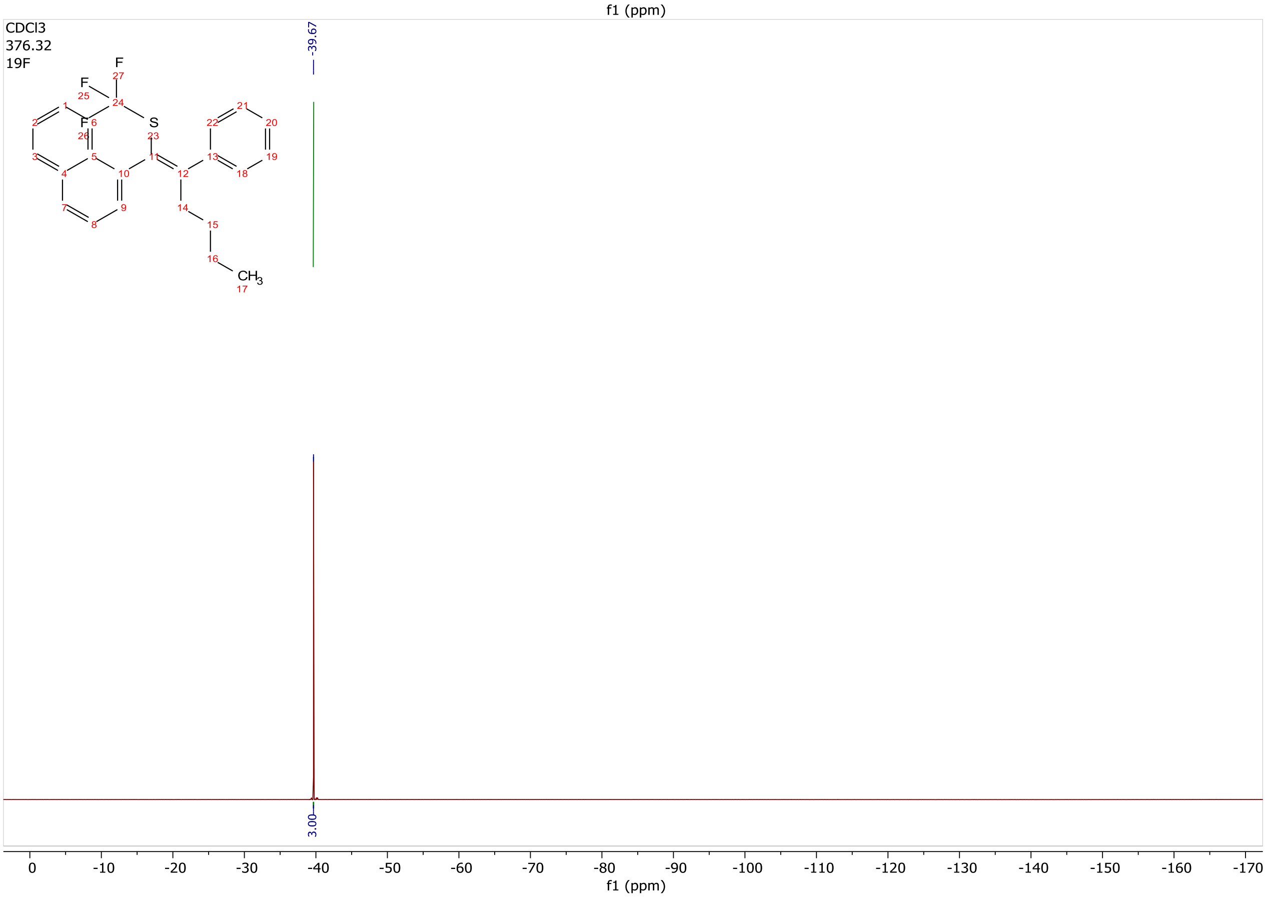

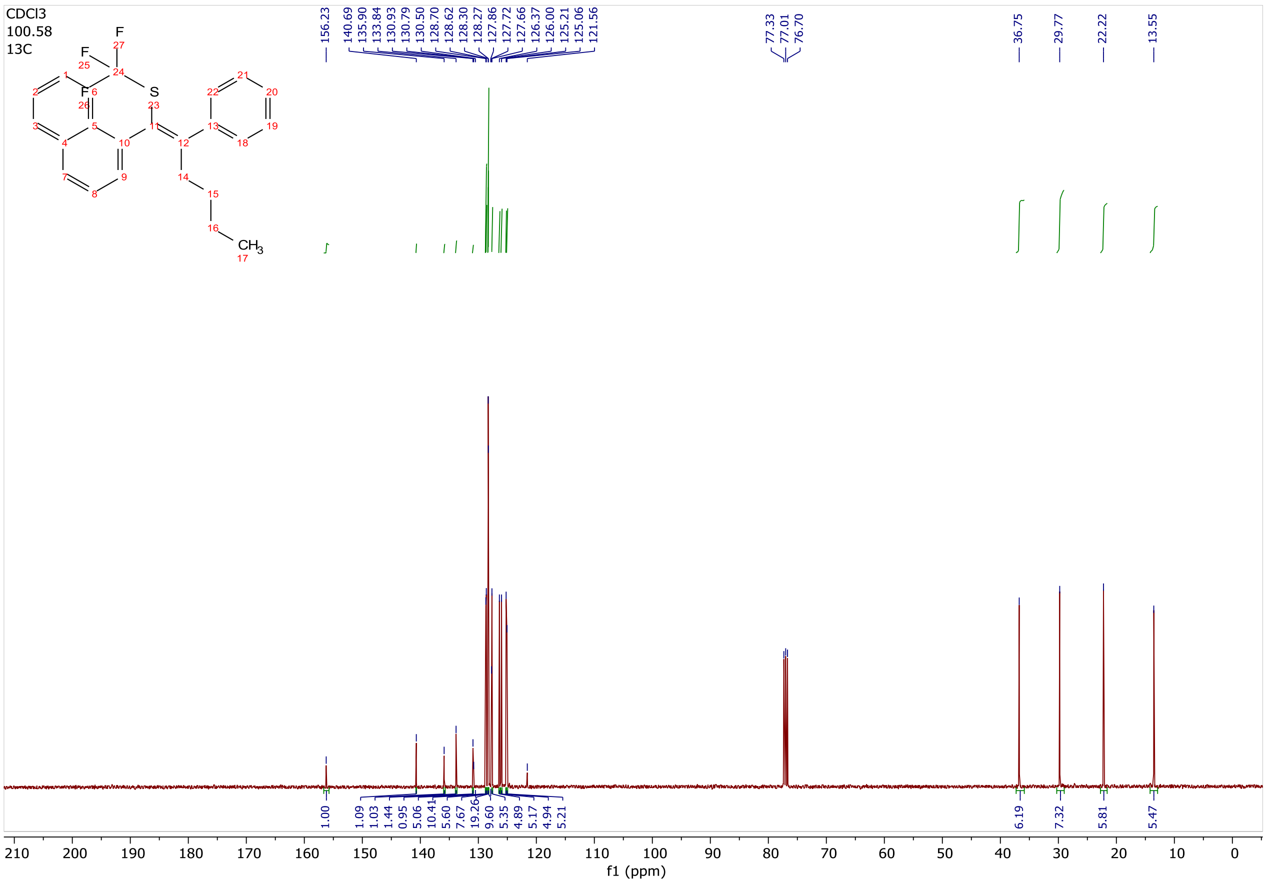
CDCl3
100.58
13C
156.23
140.69
135.90
133.84
130.93
130.79
130.50
128.70
128.62
128.30
128.27
127.86
127.72
127.66
126.37
126.00
125.21
125.06
121.56
77.33
77.01
76.70
36.75
29.77
22.22
13.55
1.00
1.09
1.03
1.44
0.95
5.06
10.41
5.60
7.67
19.26
9.60
5.35
4.89
5.17
4.94
5.21
6.19
7.32
5.81
5.47
f1 (ppm)

(*Z*)-(1-(4-fluorophenyl)-2-phenylhex-1-en-1-yl))trifluoromethyl)sulfane (3i) (400 MHz [^{1}H];101 MHz [^{13}C], 376 MHz [^{19}F] $CDCl_3$)

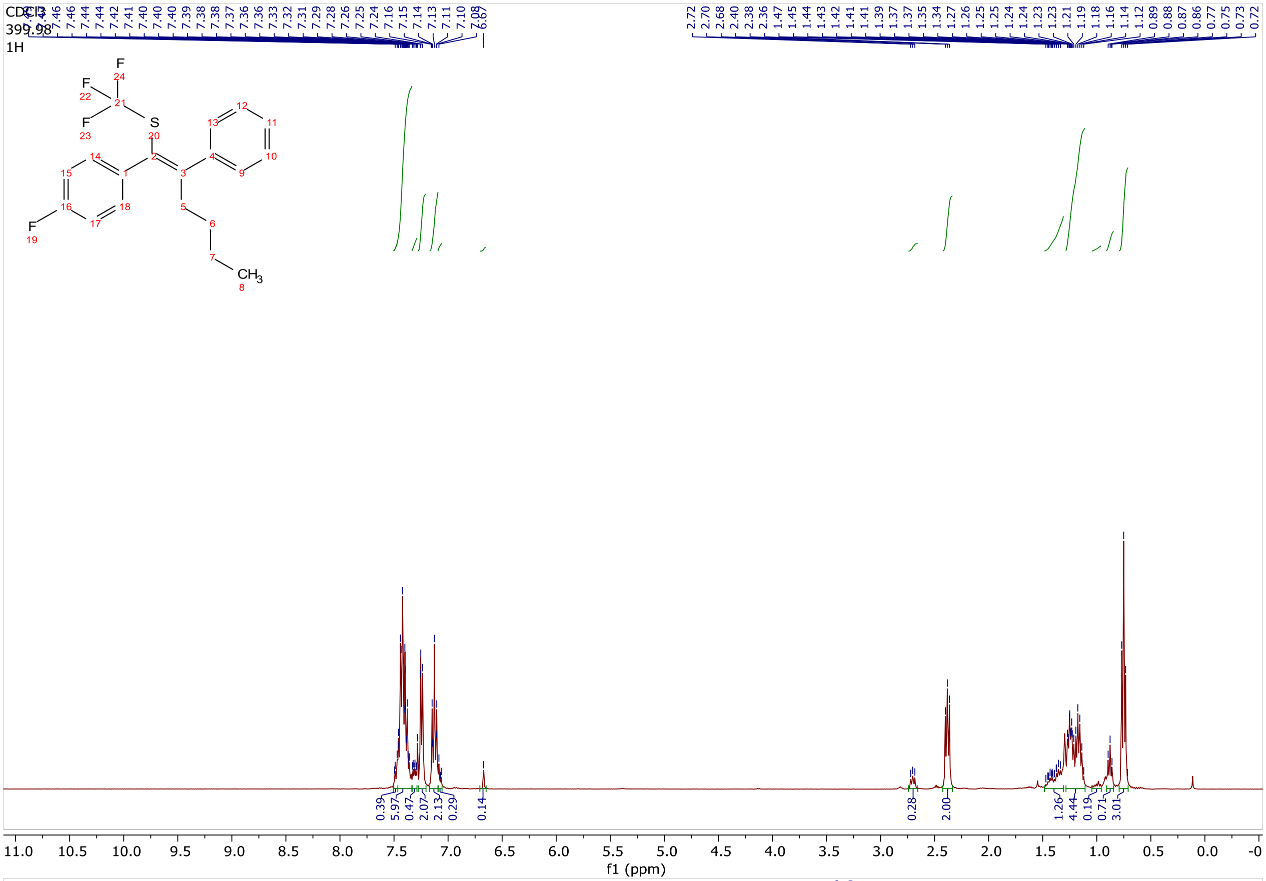

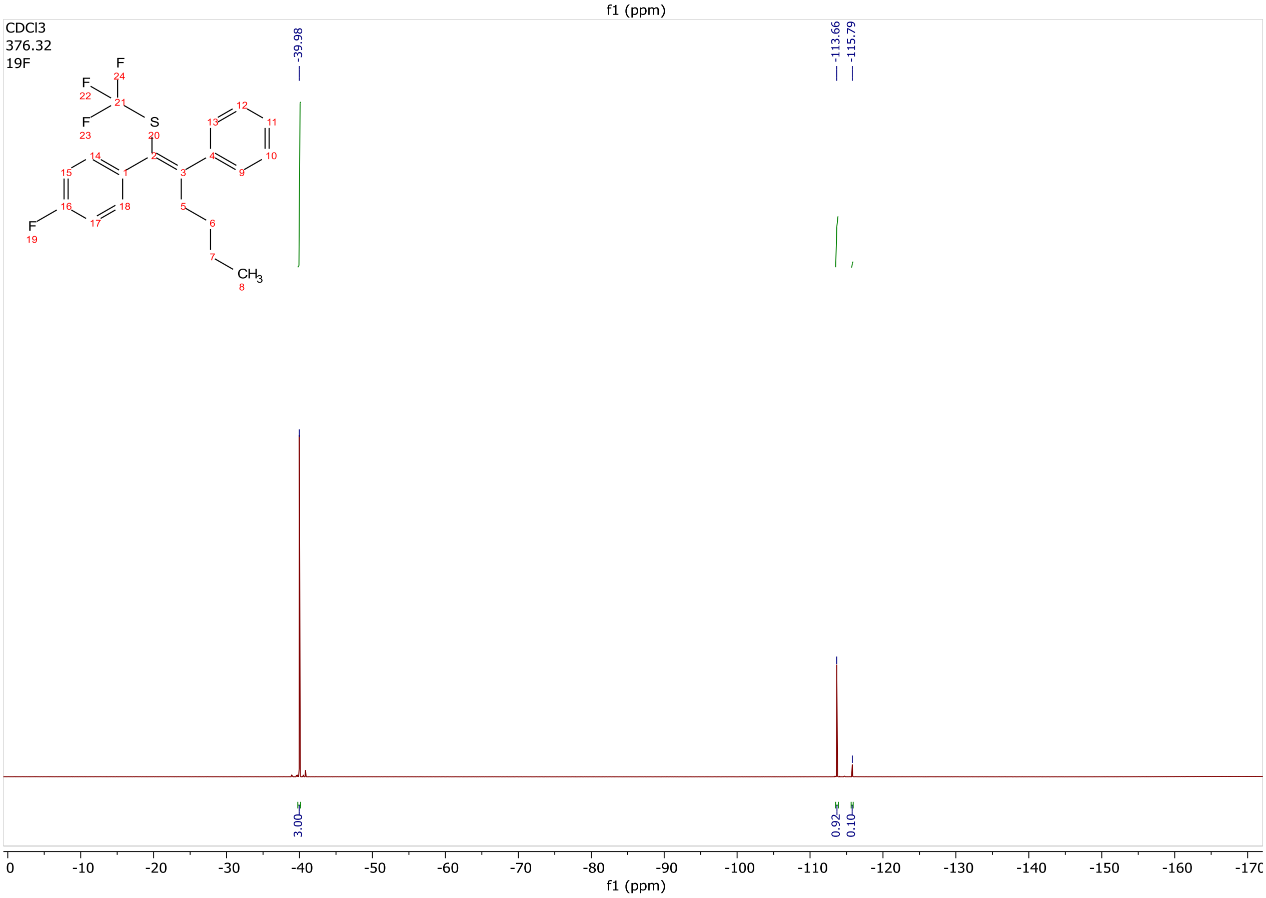

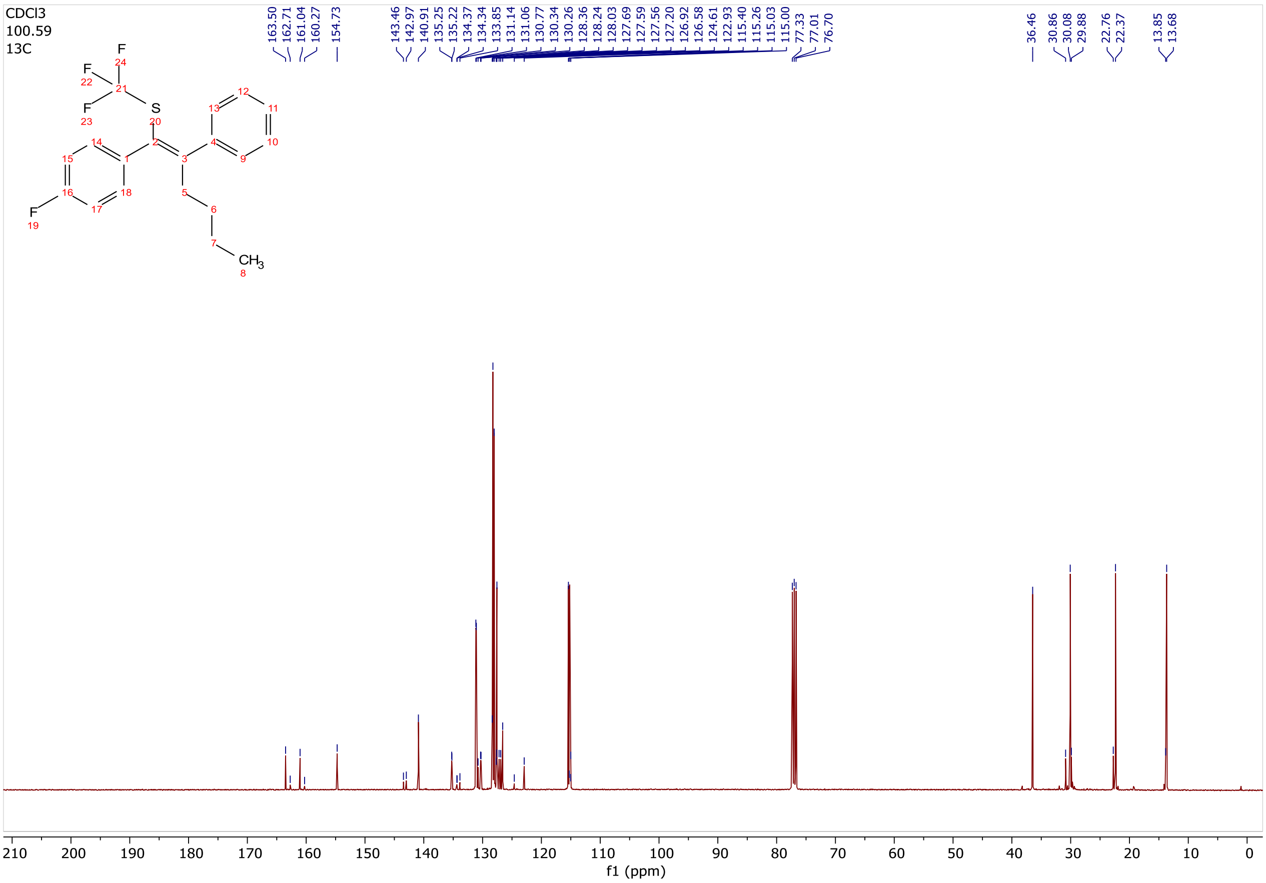
CDCl3
100.59
13C
163.50
162.71
161.04
160.27
154.73
143.46
142.97
140.91
135.25
135.22
134.37
134.34
133.85
131.14
131.06
130.77
130.34
130.26
128.36
128.24
128.03
127.69
127.59
127.56
127.20
126.92
126.58
124.61
122.93
115.40
115.26
115.03
115.00
77.33
77.01
76.70
36.46
30.86
30.08
29.88
22.76
22.37
13.85
13.68
f1 (ppm)

(Z)-4-(2-phenyl-1-((trifluoromethyl)thio)hex-1-en-1-yl)phenyl 4-methylbenzenesulfonate (3j) (400 MHz [^{1}H];101 MHz [^{13}C], 376 MHz [^{19}F] $CDCl_3$)

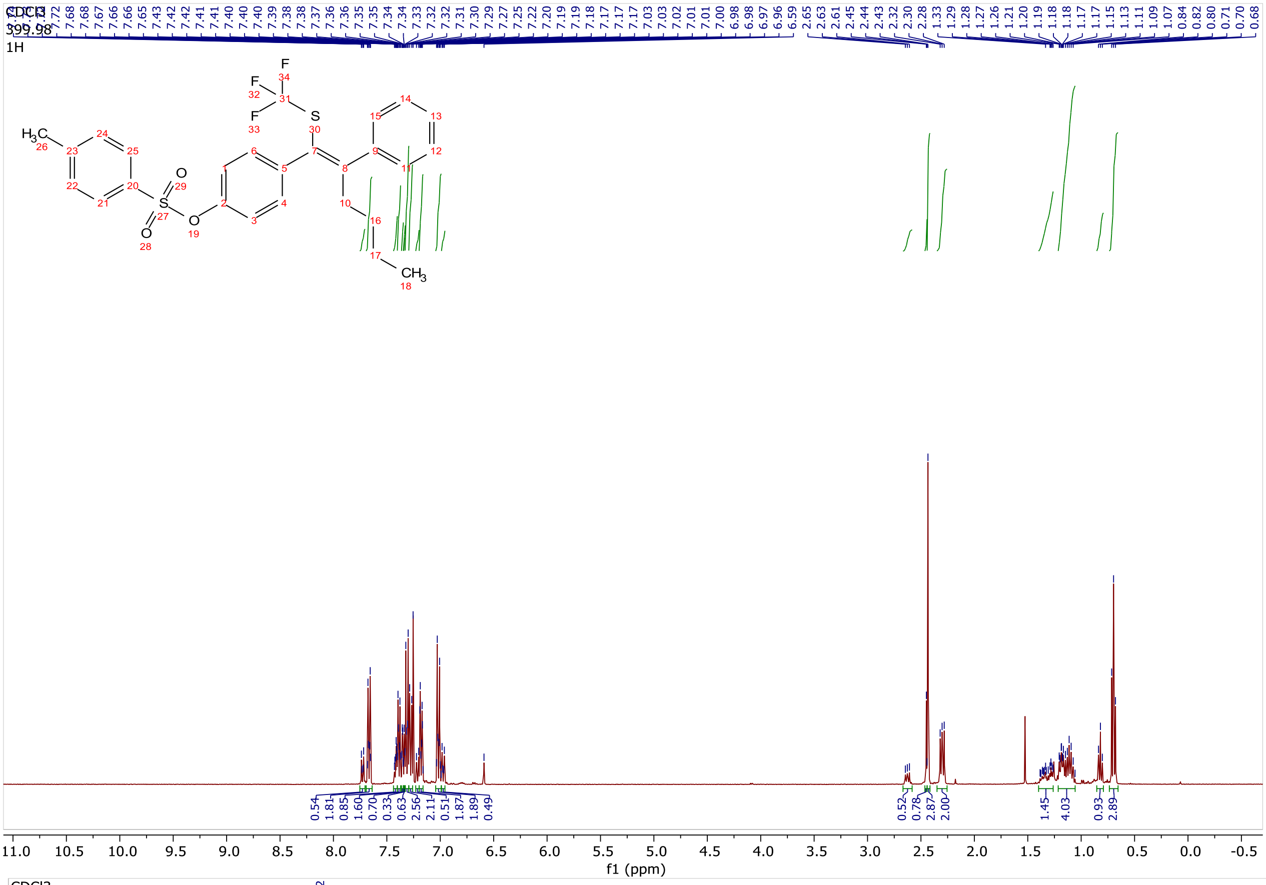

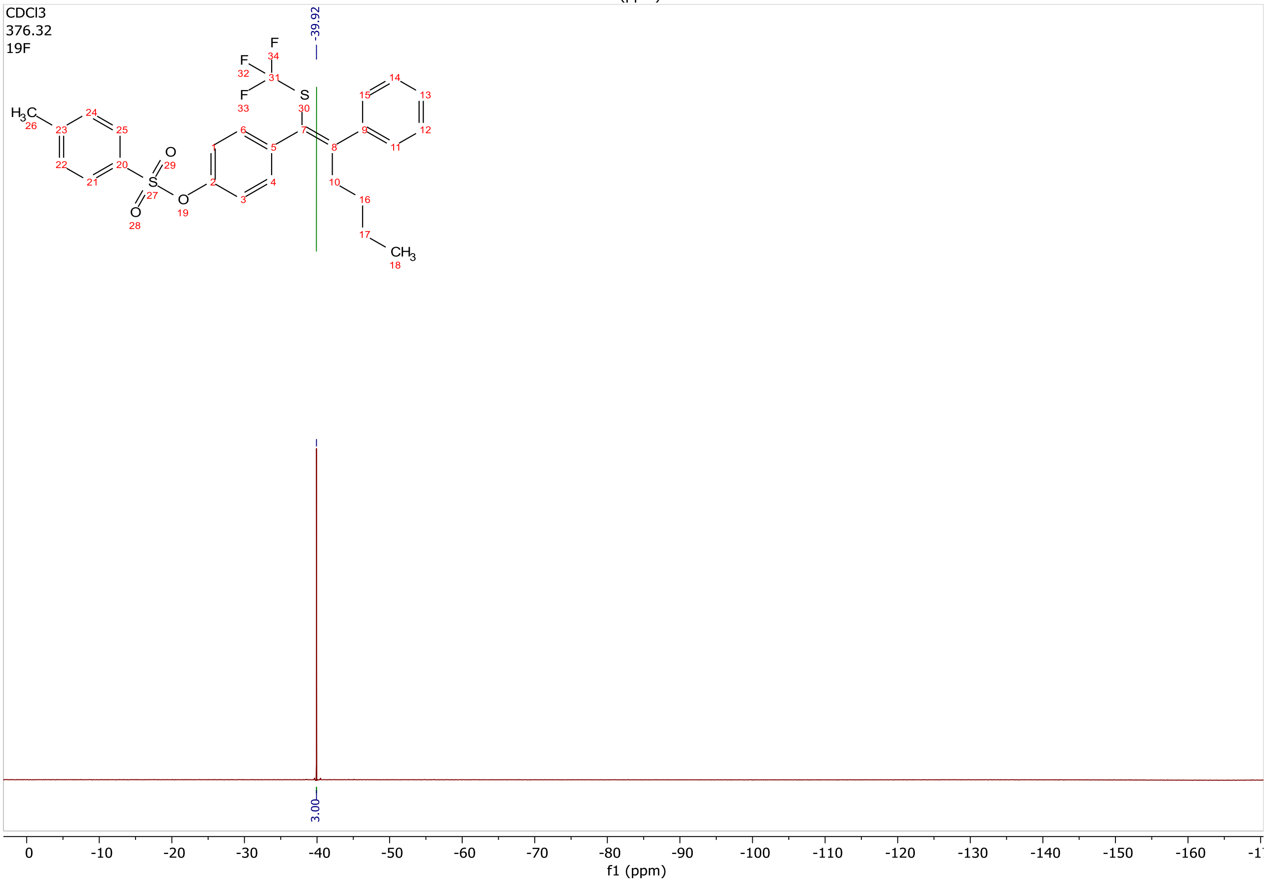

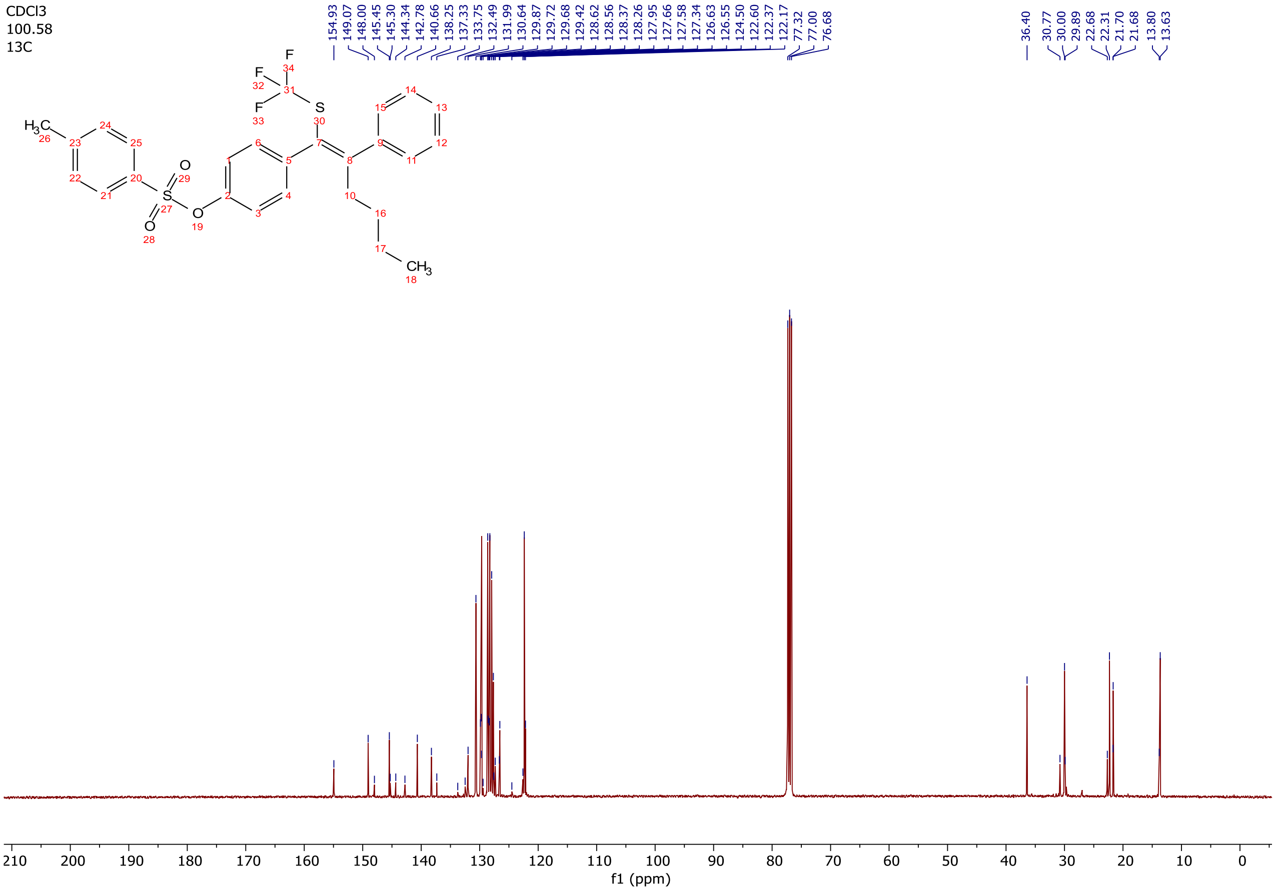

CDCl3
100.58
13C
154.93
149.07
148.00
145.45
145.30
144.34
142.78
140.66
138.25
137.33
133.75
132.49
131.99
130.64
129.87
129.72
129.68
129.42
128.62
128.56
128.37
128.26
127.95
127.66
127.58
127.34
126.63
126.55
124.50
122.60
122.37
122.17
77.32
77.00
76.68
36.40
30.77
30.00
29.89
22.68
22.31
21.70
21.68
13.80
13.63
f1 (ppm)

(Z)-4-(2-phenyl-1-((trifluoromethyl)thio)hex-1-en-1-yl)phenyl trifluoromethanesulfonate (3k) (400 MHz [^{1}H];101 MHz [^{13}C], 376 MHz [^{19}F] $CDCl_3$)

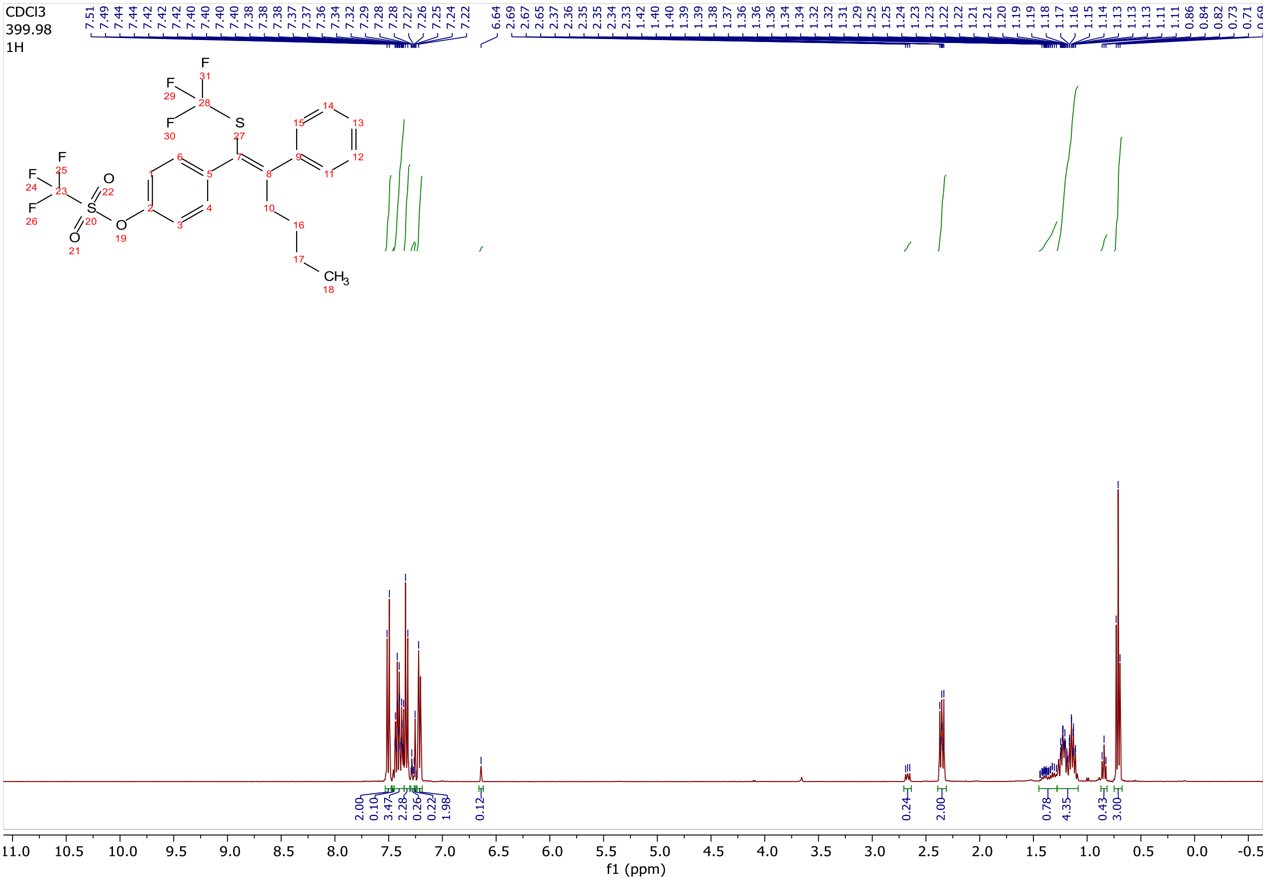

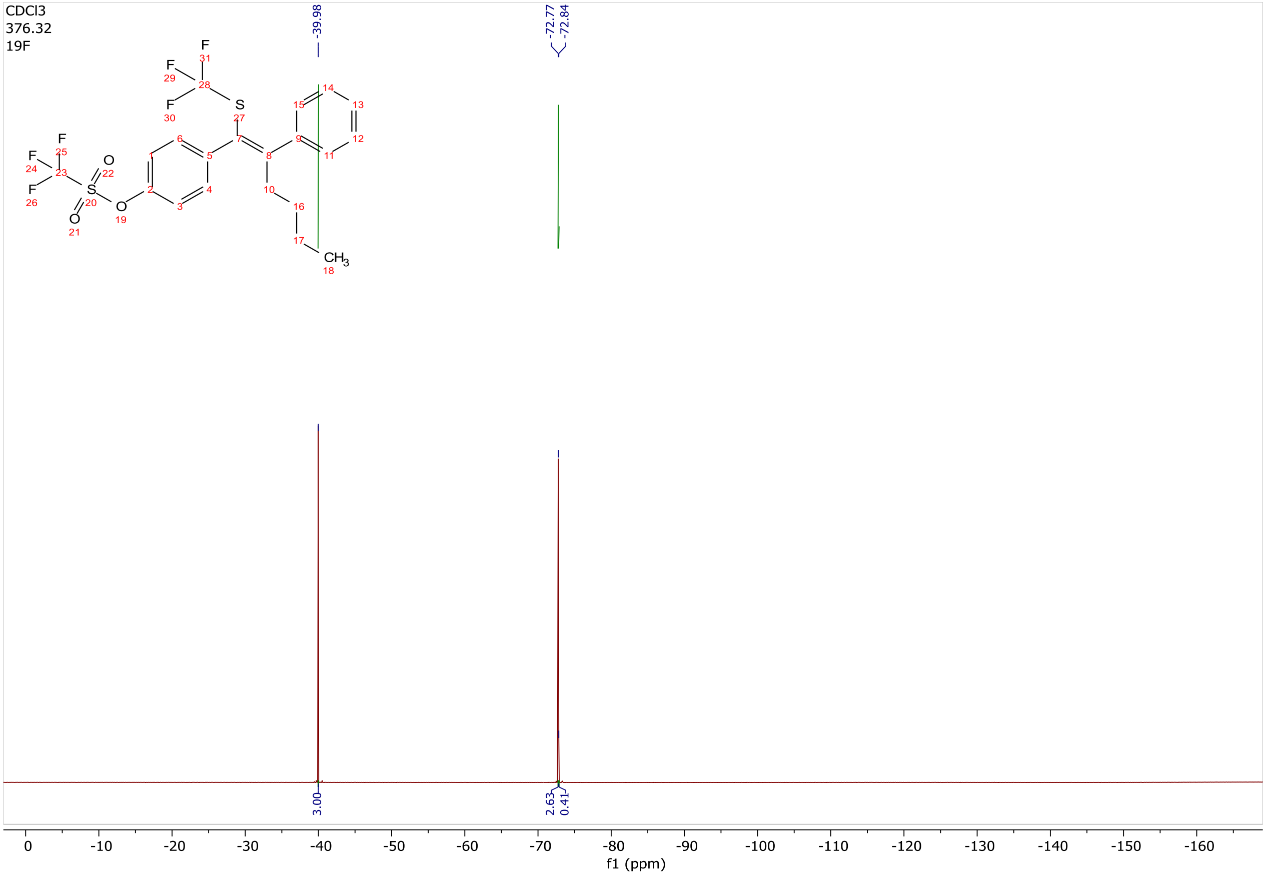

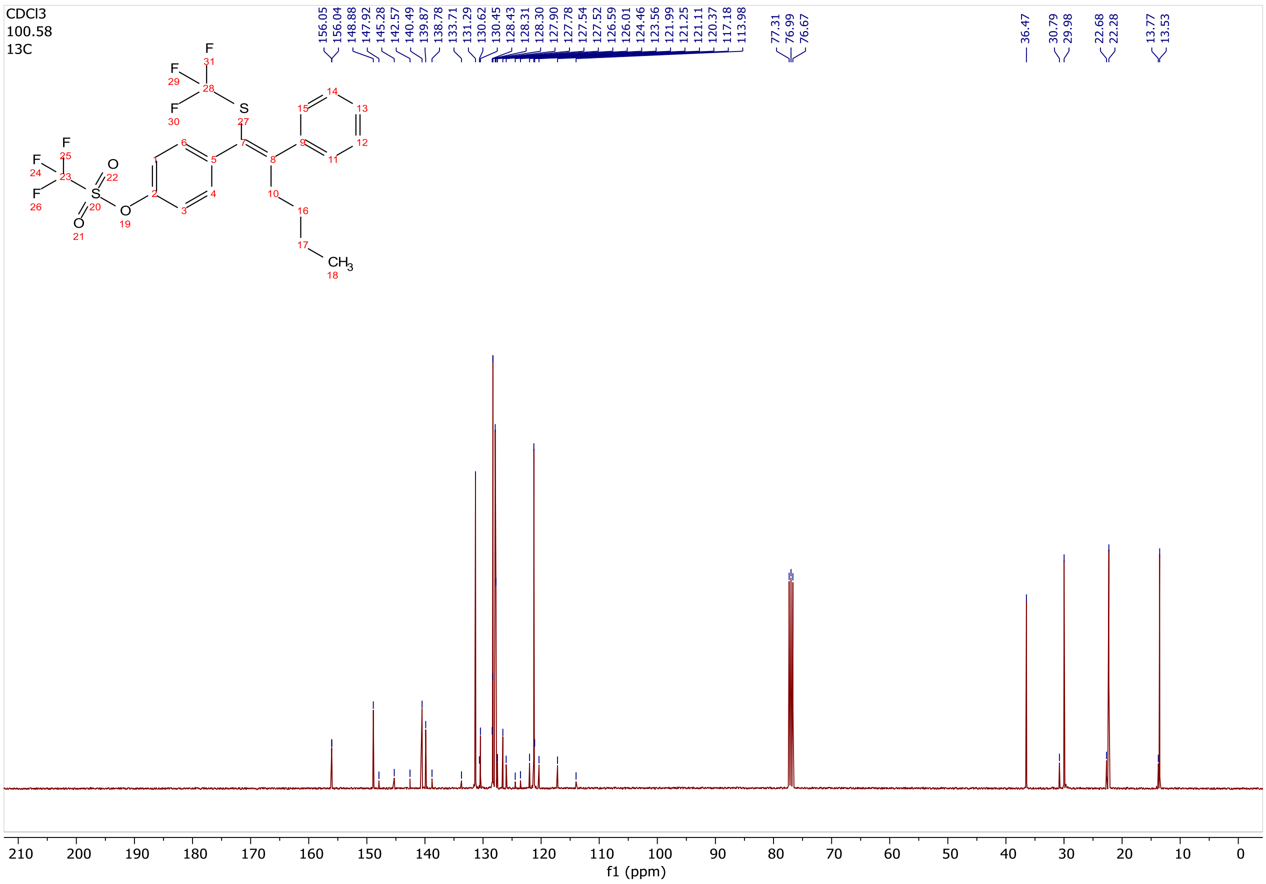
CDCl3
100.58
13C
156.05
156.04
148.88
147.92
145.28
142.57
140.49
139.87
138.78
133.71
131.29
130.62
130.45
128.43
128.31
128.30
127.90
127.78
127.54
127.52
126.59
126.01
124.46
123.56
121.99
121.25
121.11
120.37
117.18
113.98
77.31
76.99
76.67
36.47
30.79
29.98
22.68
22.28
13.77
13.53
f1 (ppm)

(Z)-(1-(4-(dimethoxymethyl)phenyl)-2-phenylhex-1-en-1-yl)(trifluoromethyl)sulfane (3l) (400 MHz [^{1}H];101 MHz [^{13}C], 376 MHz [^{19}F] $CDCl_3$)

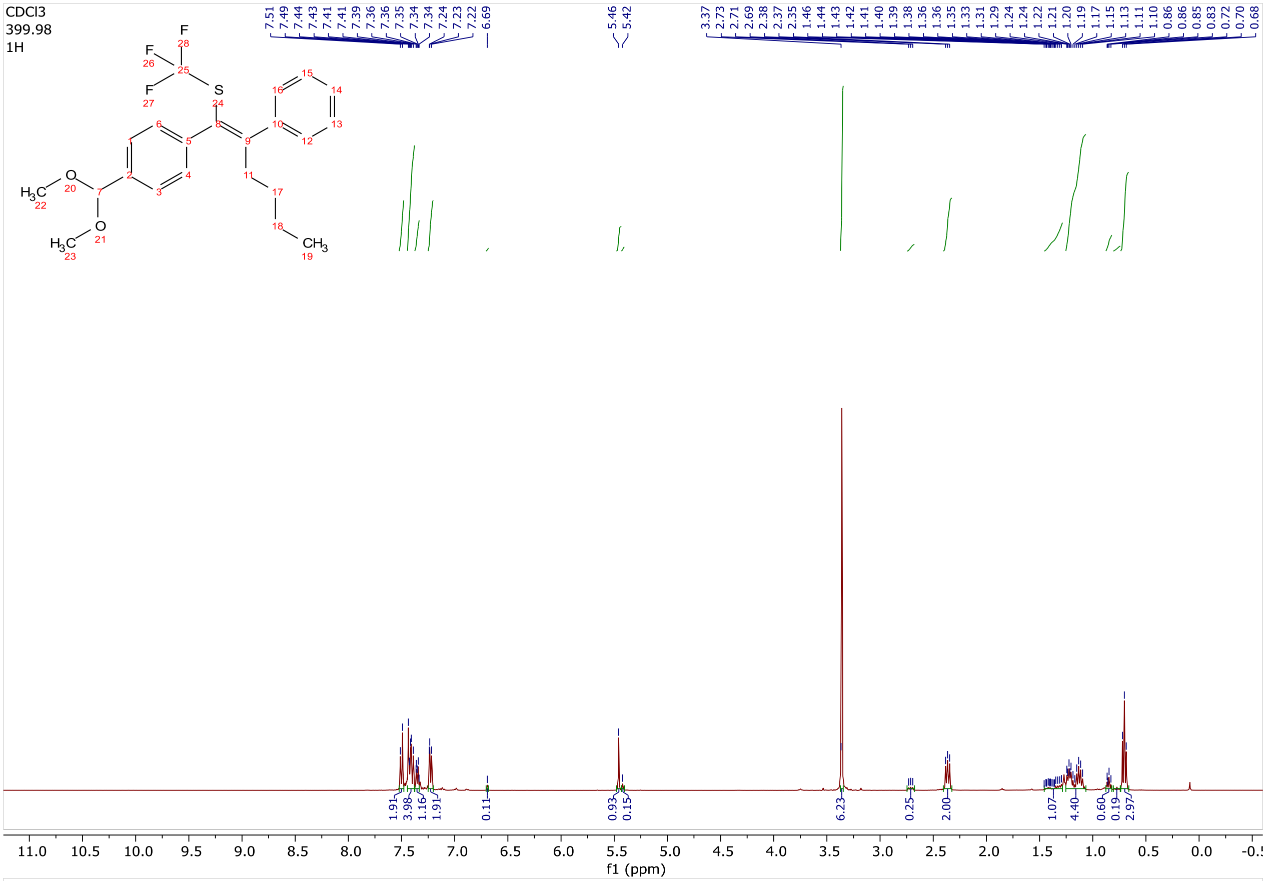

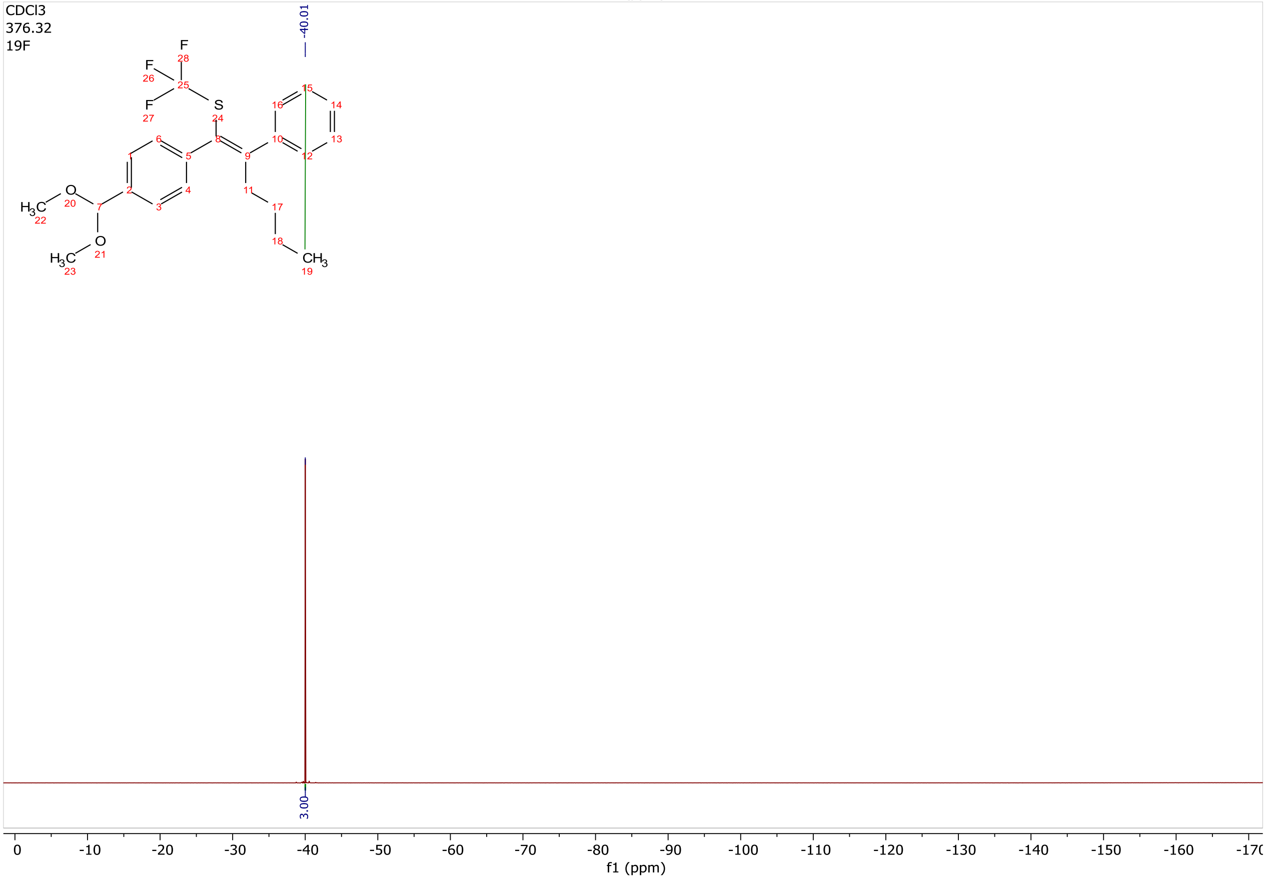

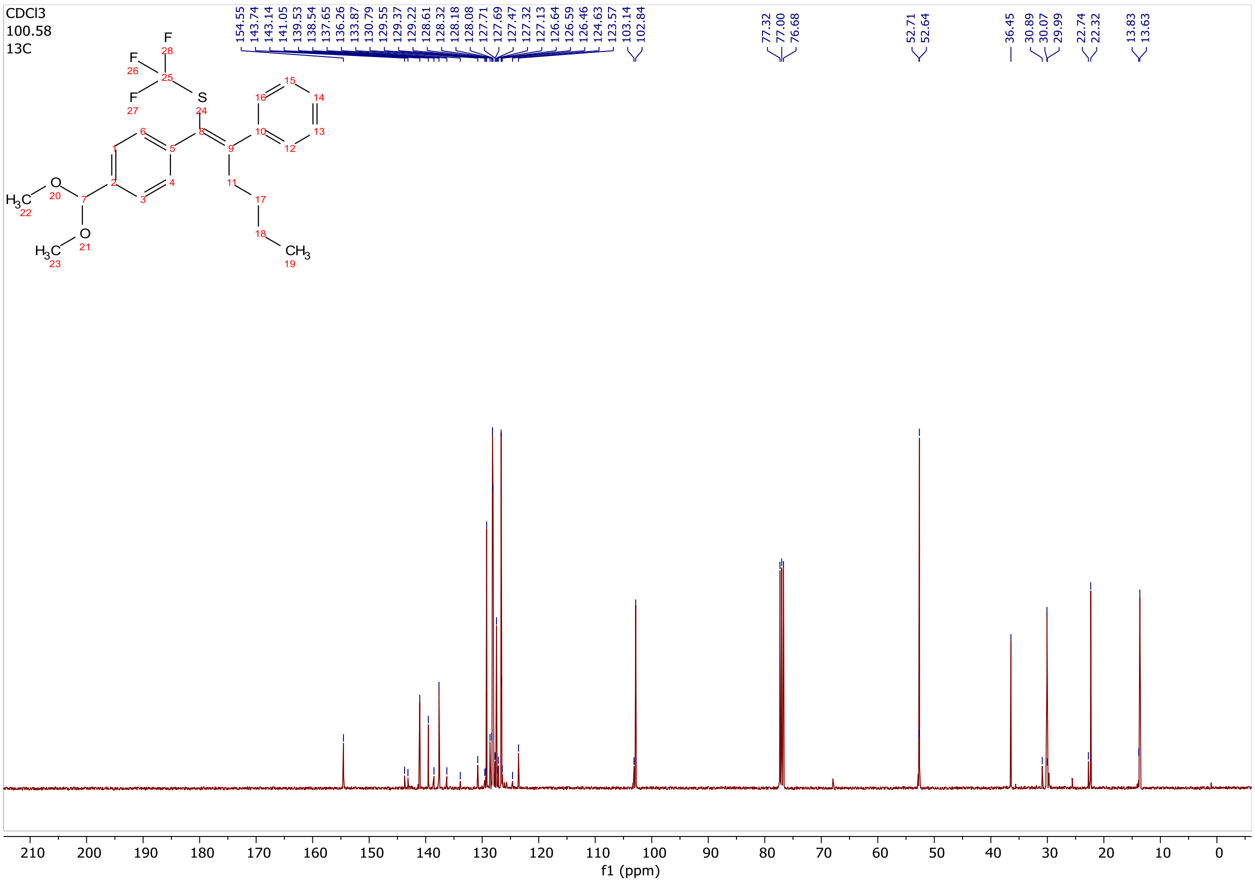
CDCl3
100.58
13C
154.55
143.74
143.14
141.05
139.53
138.54
137.65
136.26
133.87
130.79
129.55
129.37
129.22
128.61
128.32
128.18
128.08
127.71
127.69
127.47
127.32
127.13
126.64
126.59
126.46
124.63
123.57
103.14
102.84
77.32
77.00
76.68
52.71
52.64
36.45
30.89
30.07
29.99
22.74
22.32
13.83
13.63
F
F
F
S
O
O
H_3C
H_3C
CH_3
210
200
190
180
170
160
150
140
130
120
110
100
90
80
70
60
50
40
30
20
10
0
f1 (ppm)

(*Z*)-5-(2-phenyl-1-((trifluoromethyl)thio)hex-1-en-1-yl)benzo[*d*][1,3]dioxole (3m) (400 MHz [^{1}H];101 MHz [^{13}C], 376 MHz [^{19}F] $CDCl_3$)

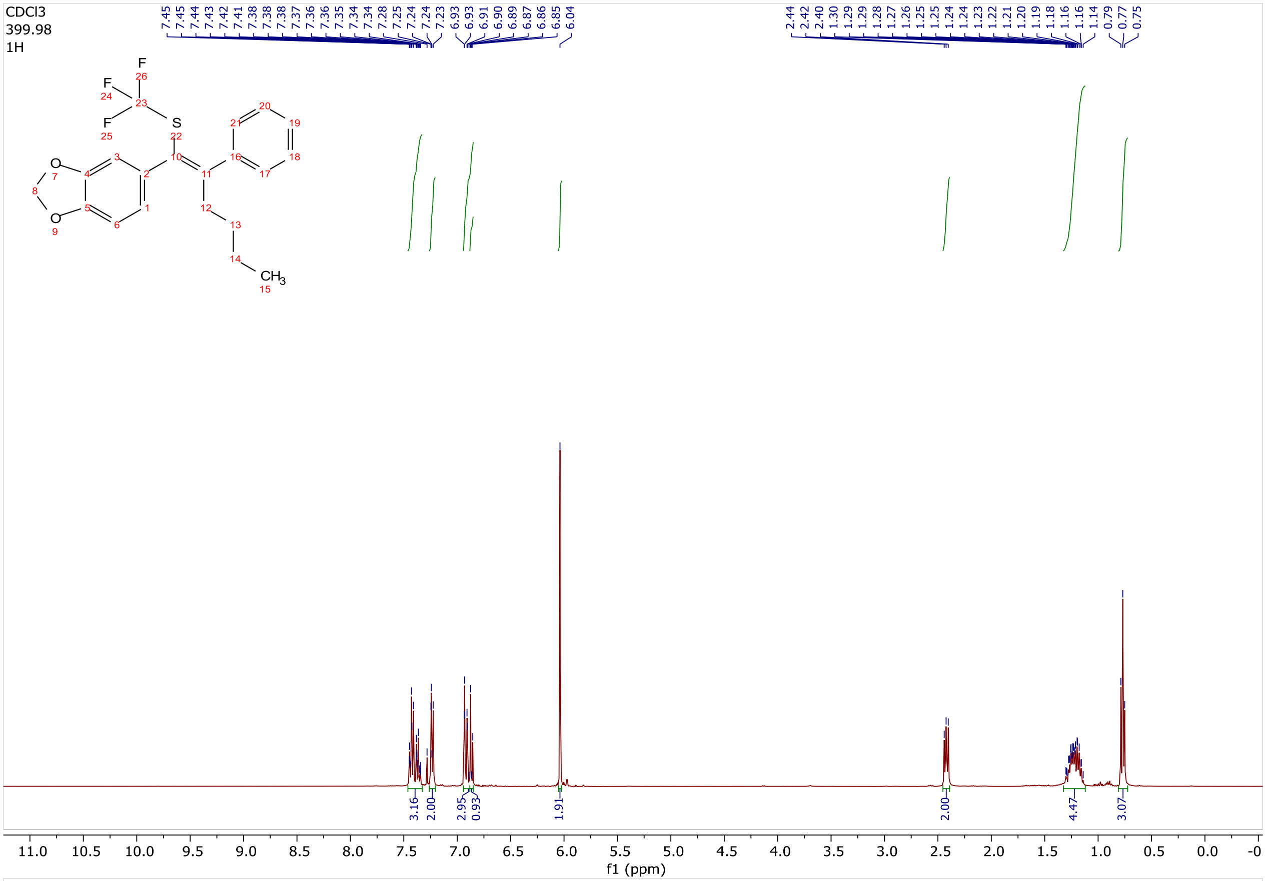

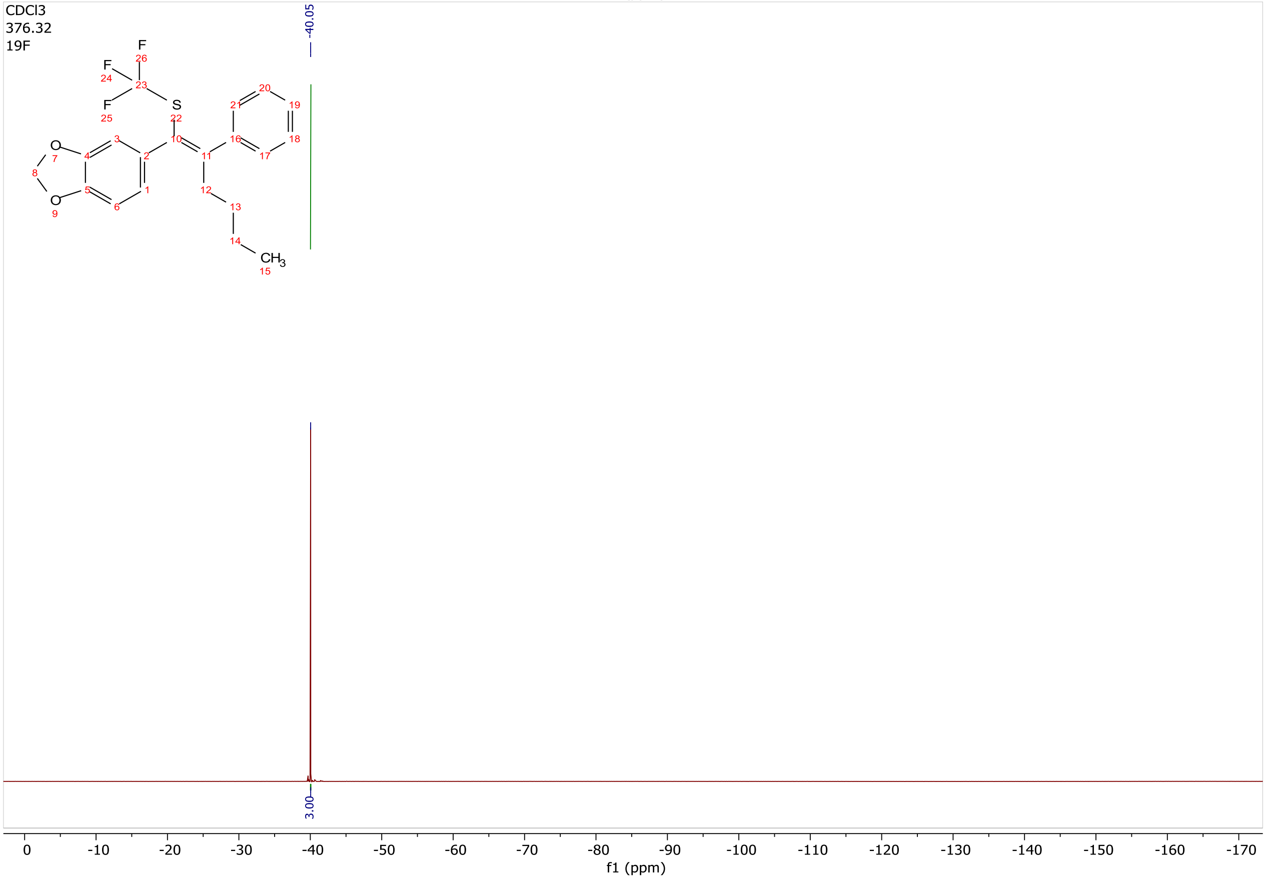

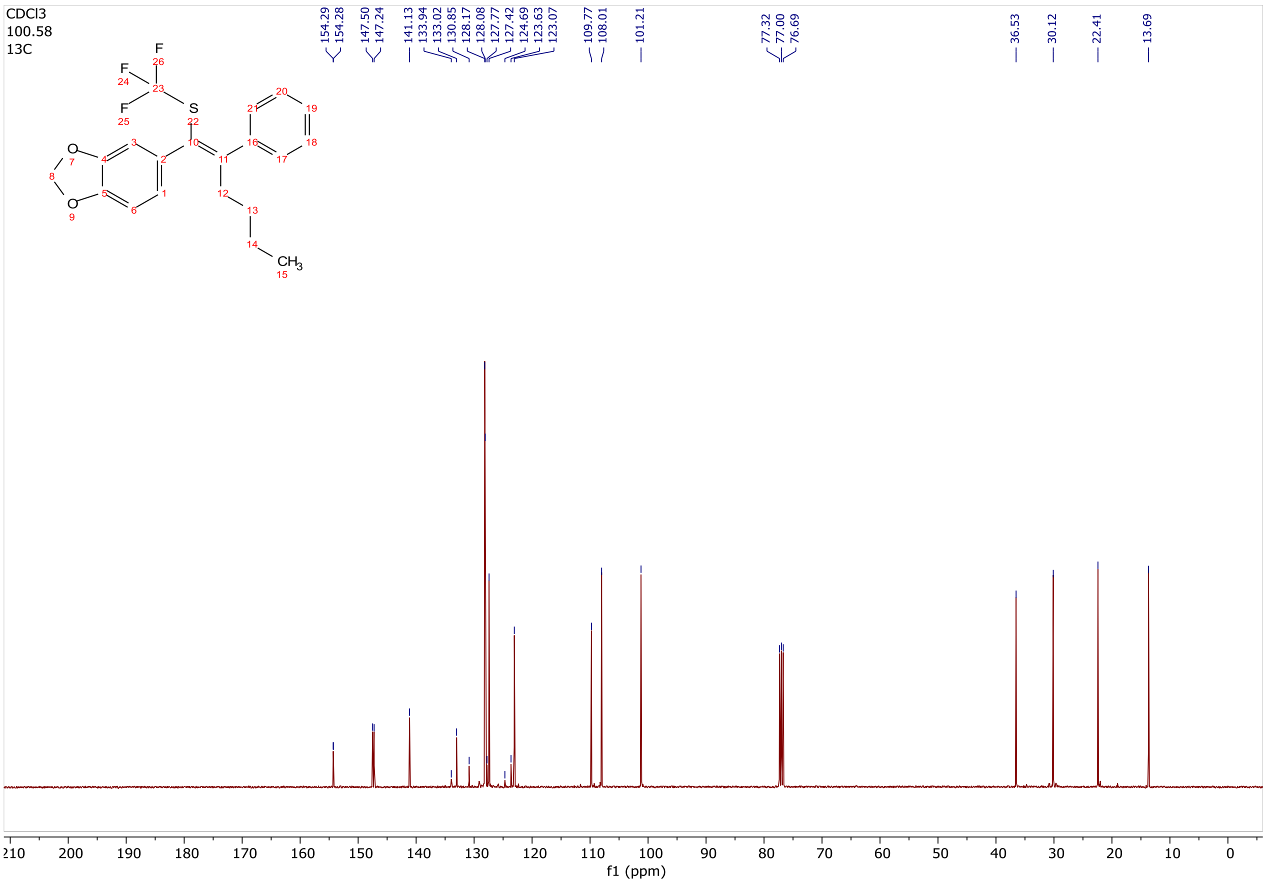

CDCl3
100.58
13C
154.29
154.28
147.50
147.24
141.13
133.94
133.02
130.85
128.17
128.08
127.77
127.42
124.69
123.63
123.07
109.77
108.01
101.21
77.32
77.00
76.69
36.53
30.12
22.41
13.69
f1 (ppm)

(*Z*)-1-methyl-5-(2-phenyl-1-((trifluoromethyl)thio)hex-1-en-1-yl)-1*H*-indole (3n) (400 MHz [^{1}H];101 MHz [^{13}C], 376 MHz [^{19}F] $CDCl_3$)

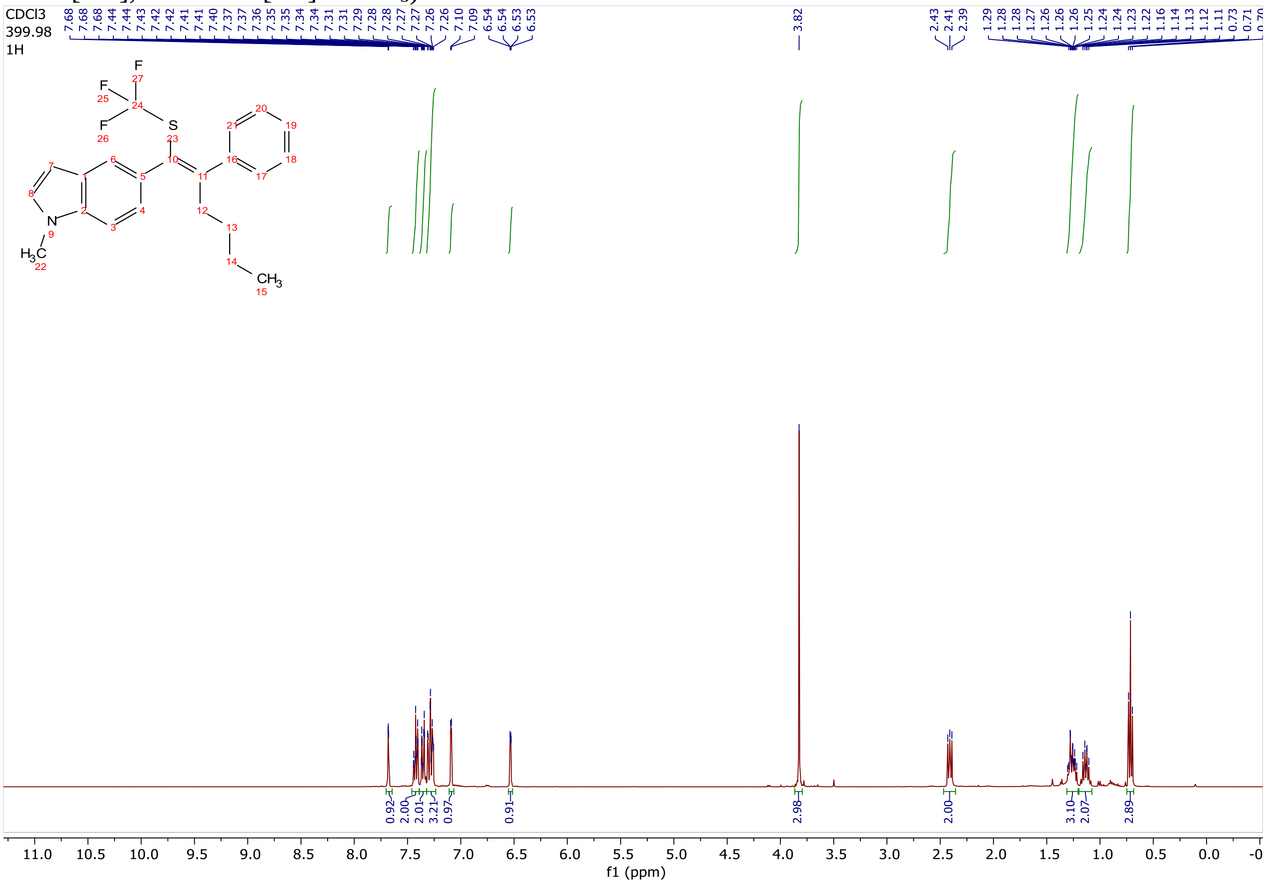

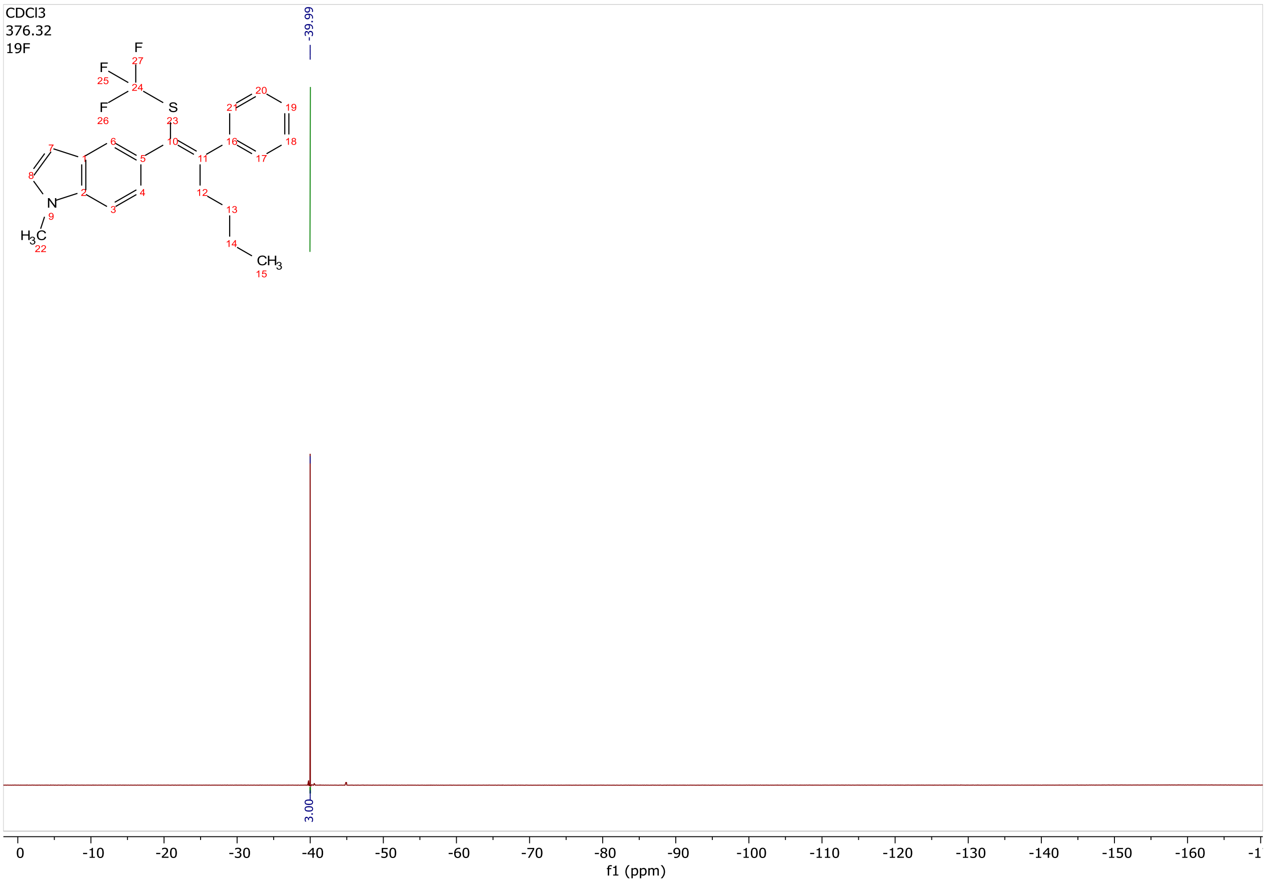

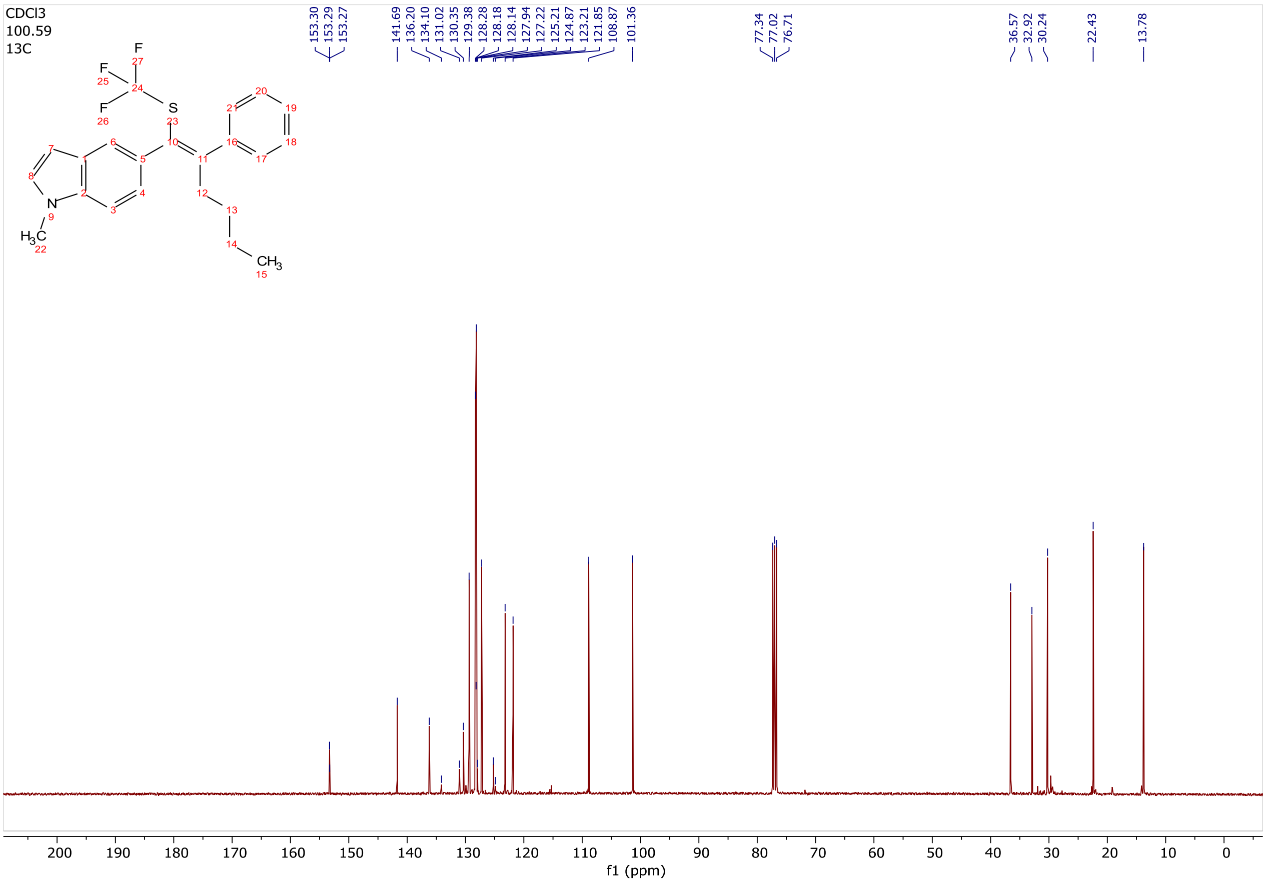
CDCl3
100.59
13C
153.30
153.29
153.27
141.69
136.20
134.10
131.02
130.35
129.38
128.28
128.18
128.14
127.94
127.22
125.21
124.87
123.21
121.85
108.87
101.36
77.34
77.02
76.71
36.57
32.92
30.24
22.43
13.78
f1 (ppm)

(*Z*)-2-(2-phenyl-1-((trifluoromethyl)thio)hex-1-en-1-yl)thiophene (3o) (400 MHz [^{1}H];101 MHz [^{13}C], 376 MHz [^{19}F] $CDCl_3$)

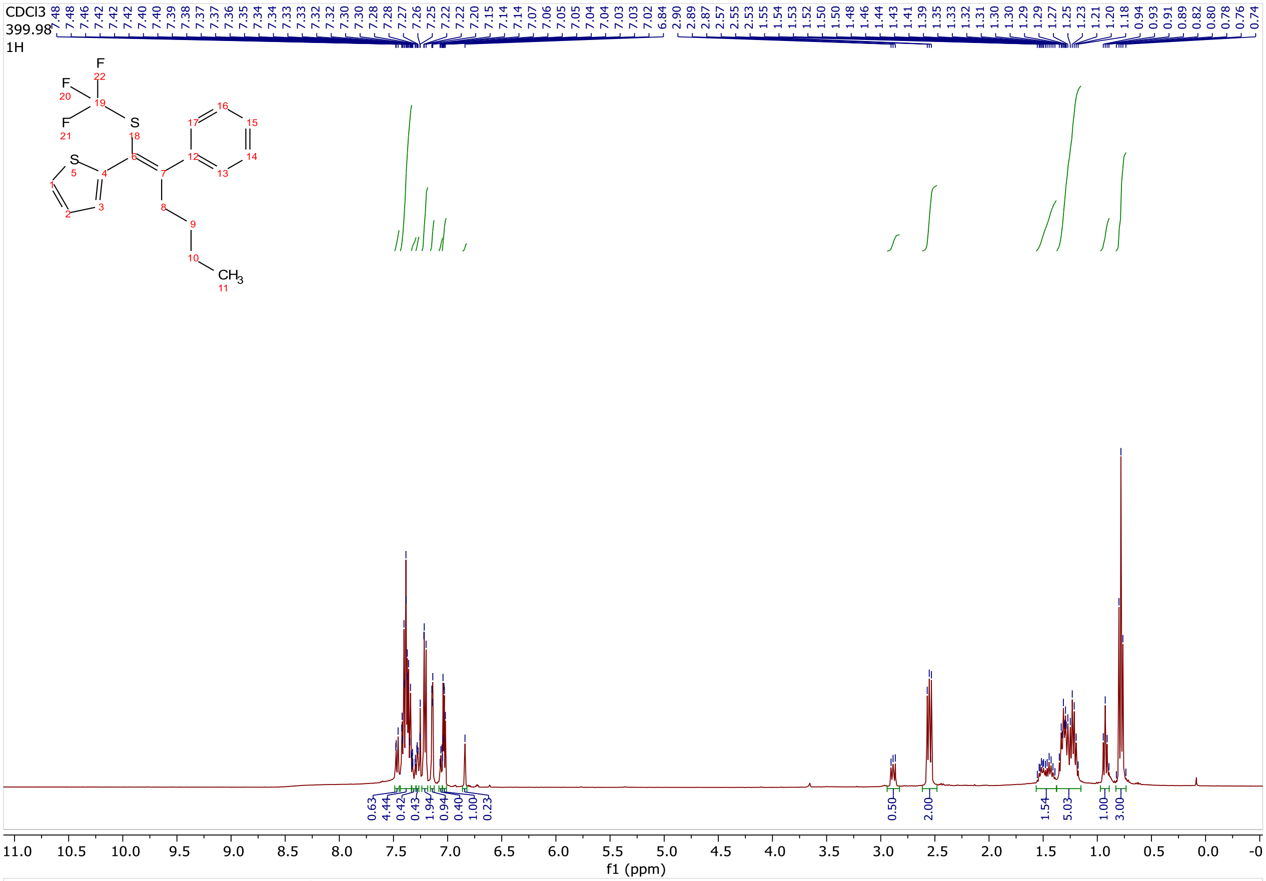

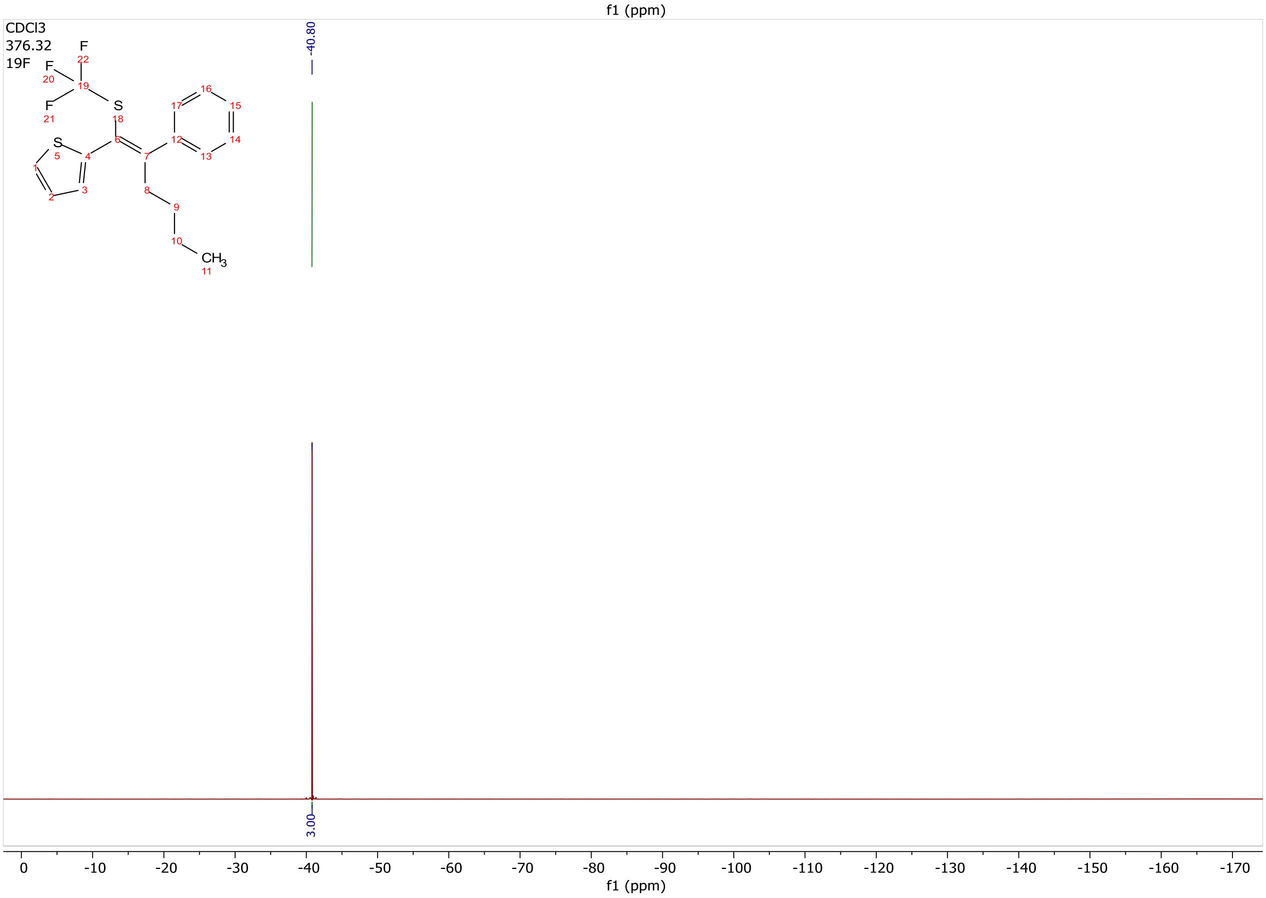

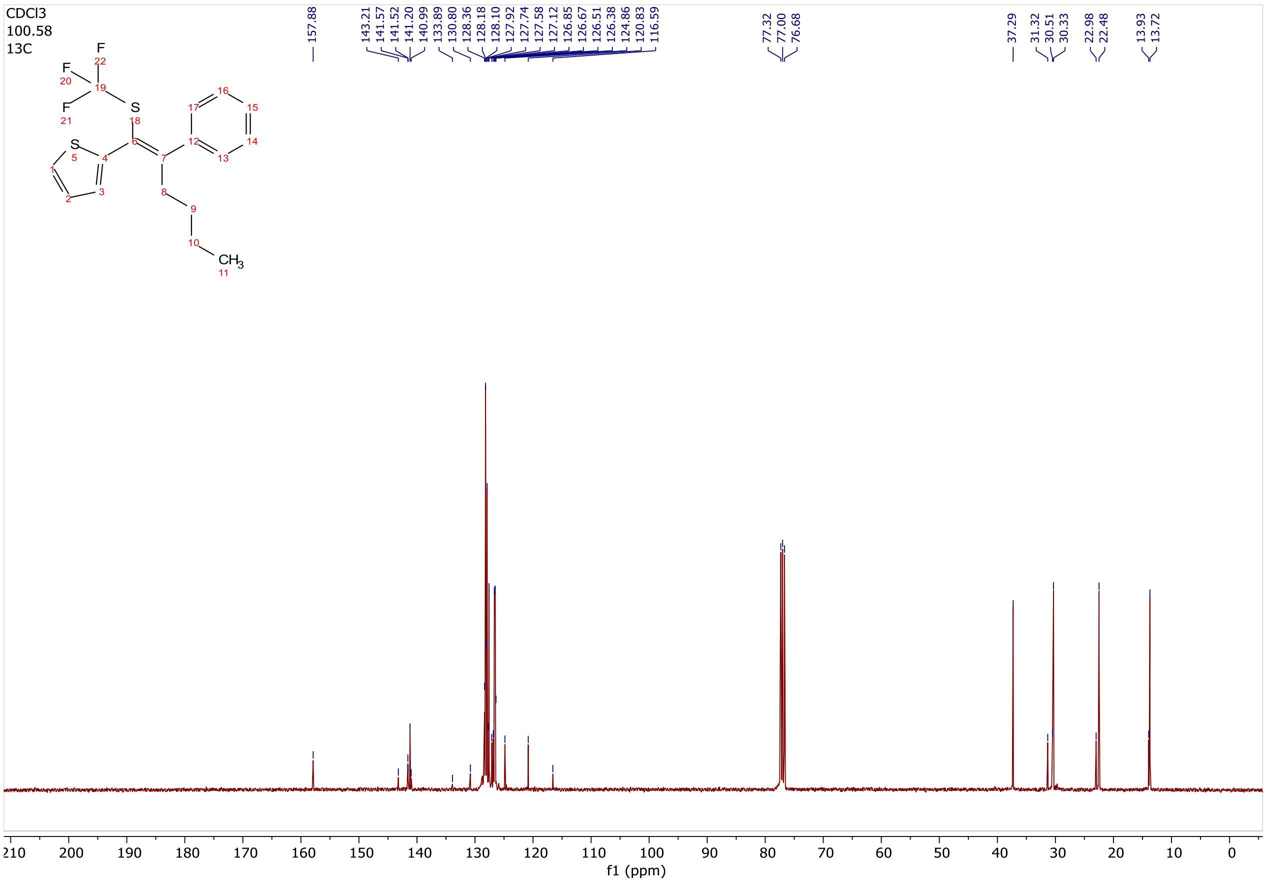
CDCl3
100.58
13C
157.88
143.21
141.57
141.52
141.20
140.99
133.89
130.80
128.36
128.18
128.10
127.92
127.74
127.58
127.12
126.85
126.67
126.51
126.38
124.86
120.83
116.59
77.32
77.00
76.68
37.29
31.32
30.51
30.33
22.98
22.48
13.93
13.72
f1 (ppm)

(Z)-(1-(cyclohex-1-en-1-yl)-2-phenylhex-1-en-1-yl)(trifluoromethyl)sulfane (3p) (400 MHz [^{1}H];101 MHz [^{13}C], 376 MHz [^{19}F] $CDCl_3$)

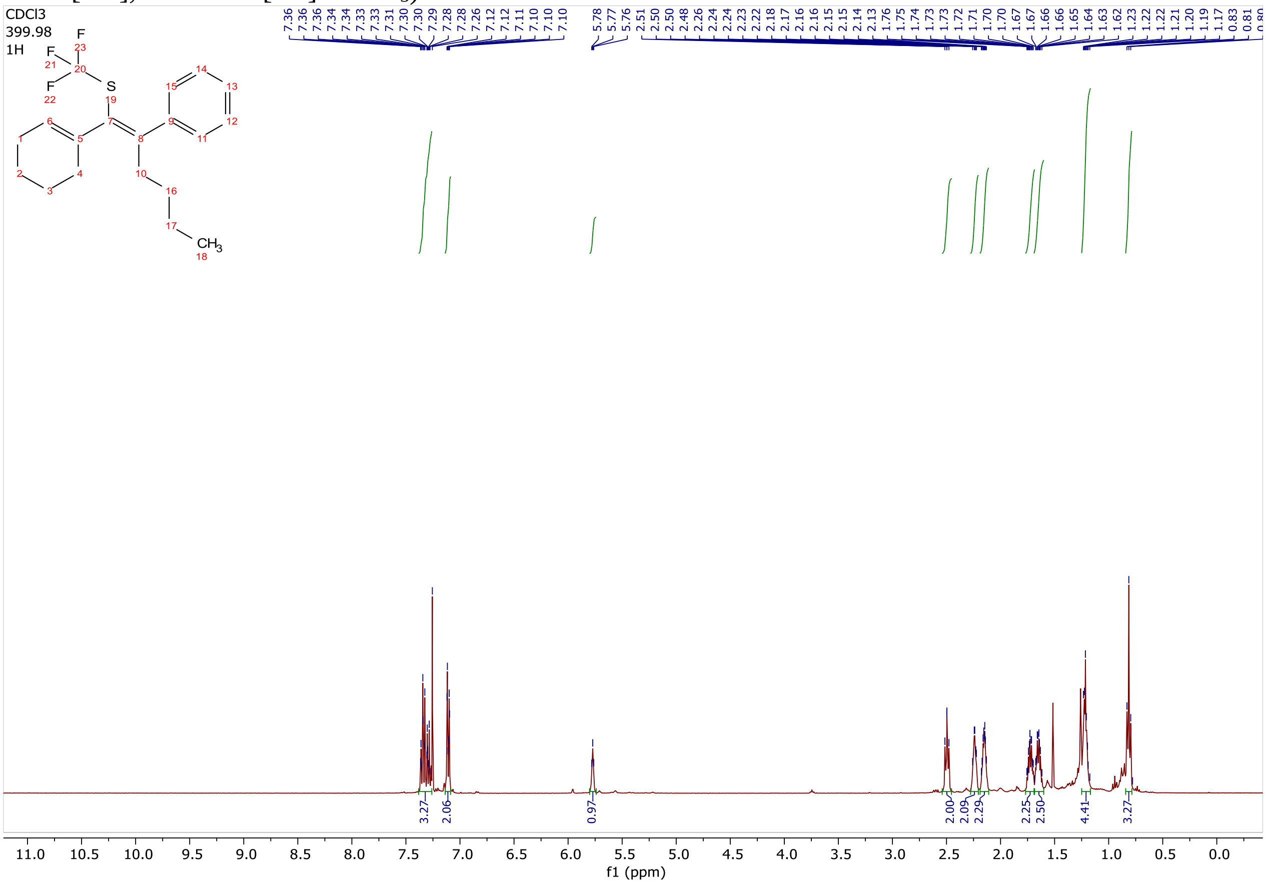

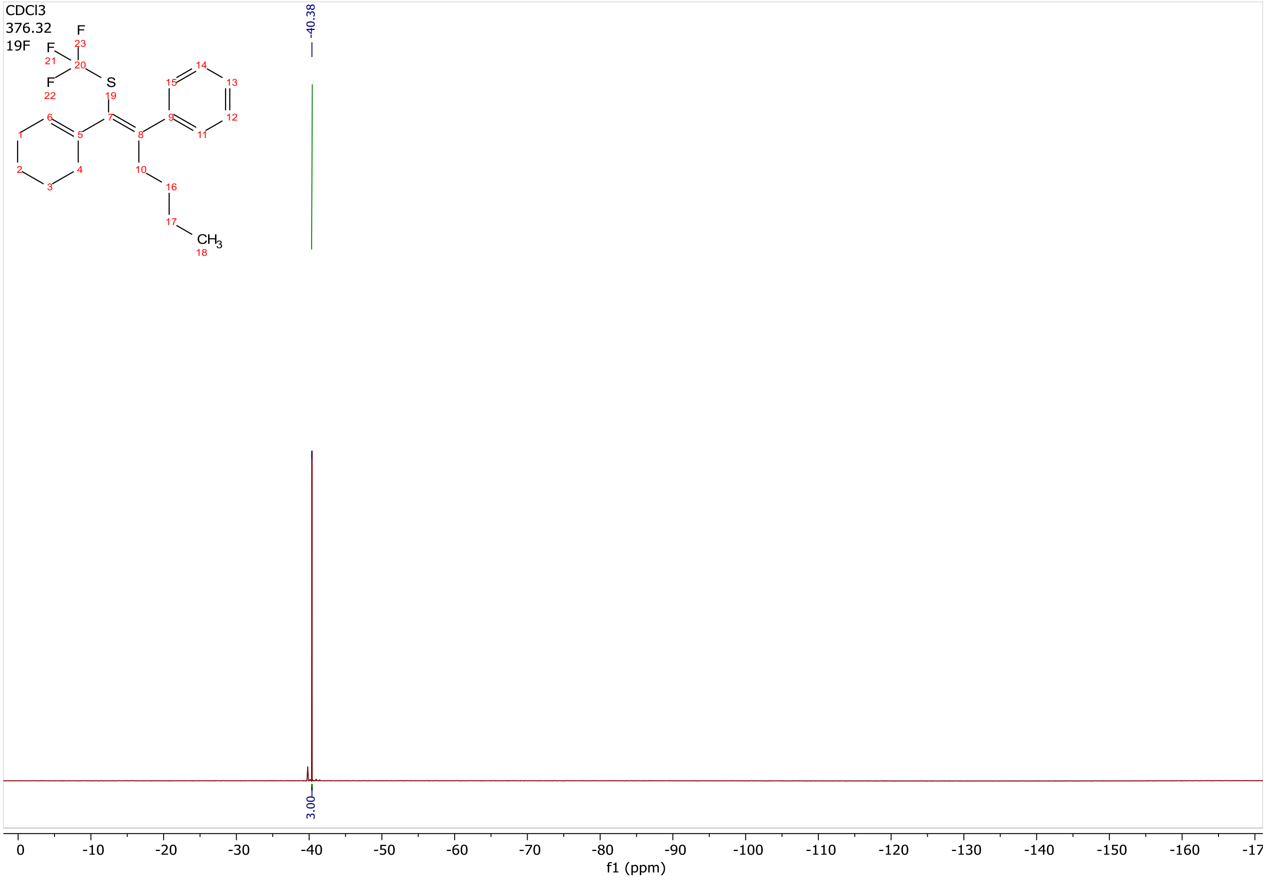

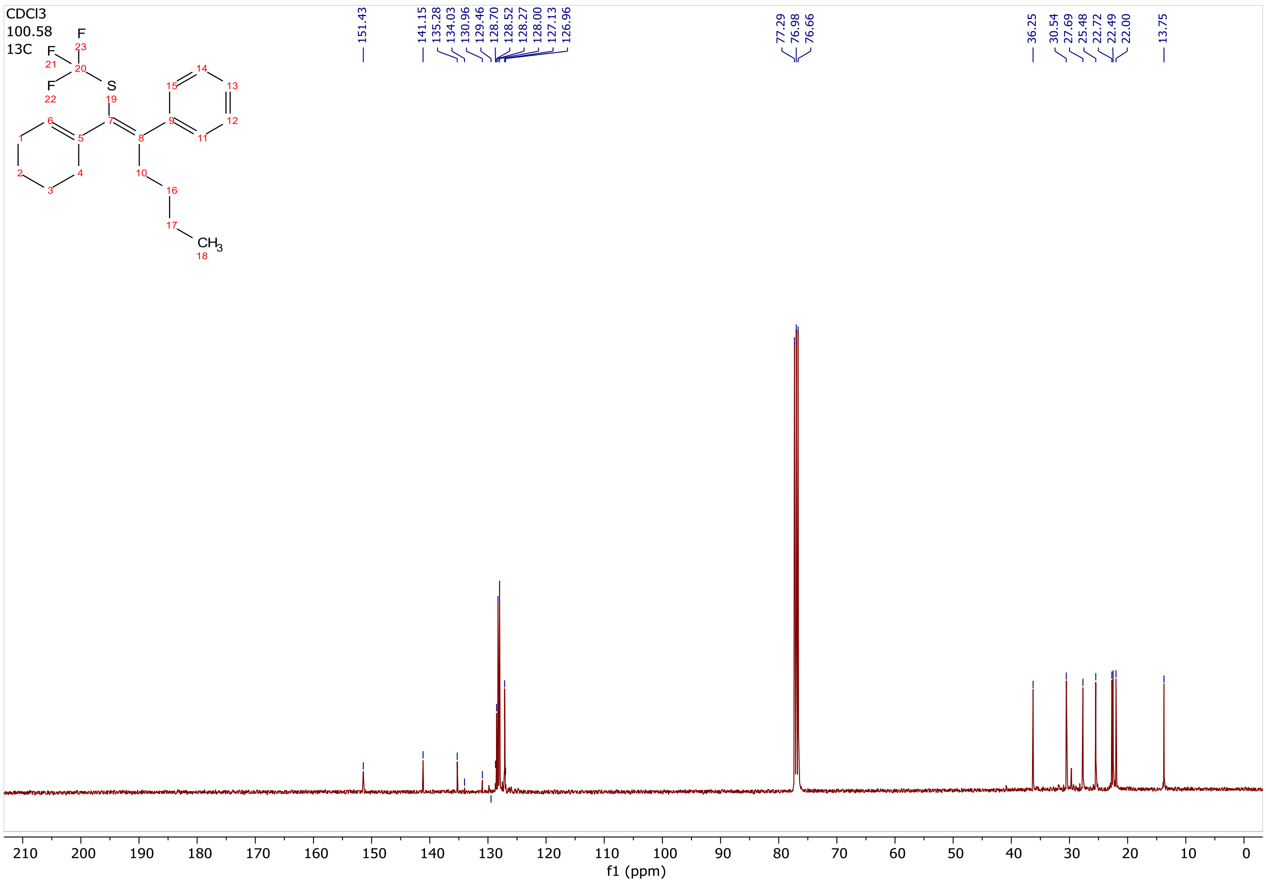
CDCl3
100.58
13C
151.43
141.15
135.28
134.03
130.96
129.46
128.70
128.52
128.27
128.00
127.13
126.96
77.29
76.98
76.66
36.25
30.54
27.69
25.48
22.72
22.49
22.00
13.75
f1 (ppm)

(Trifluoromethyl)(1,2,2-triphenylvinyl)sulfane (3q) (400 MHz [^{1}H];101 MHz [^{13}C], 376 MHz [^{19}F] $CDCl_3$)

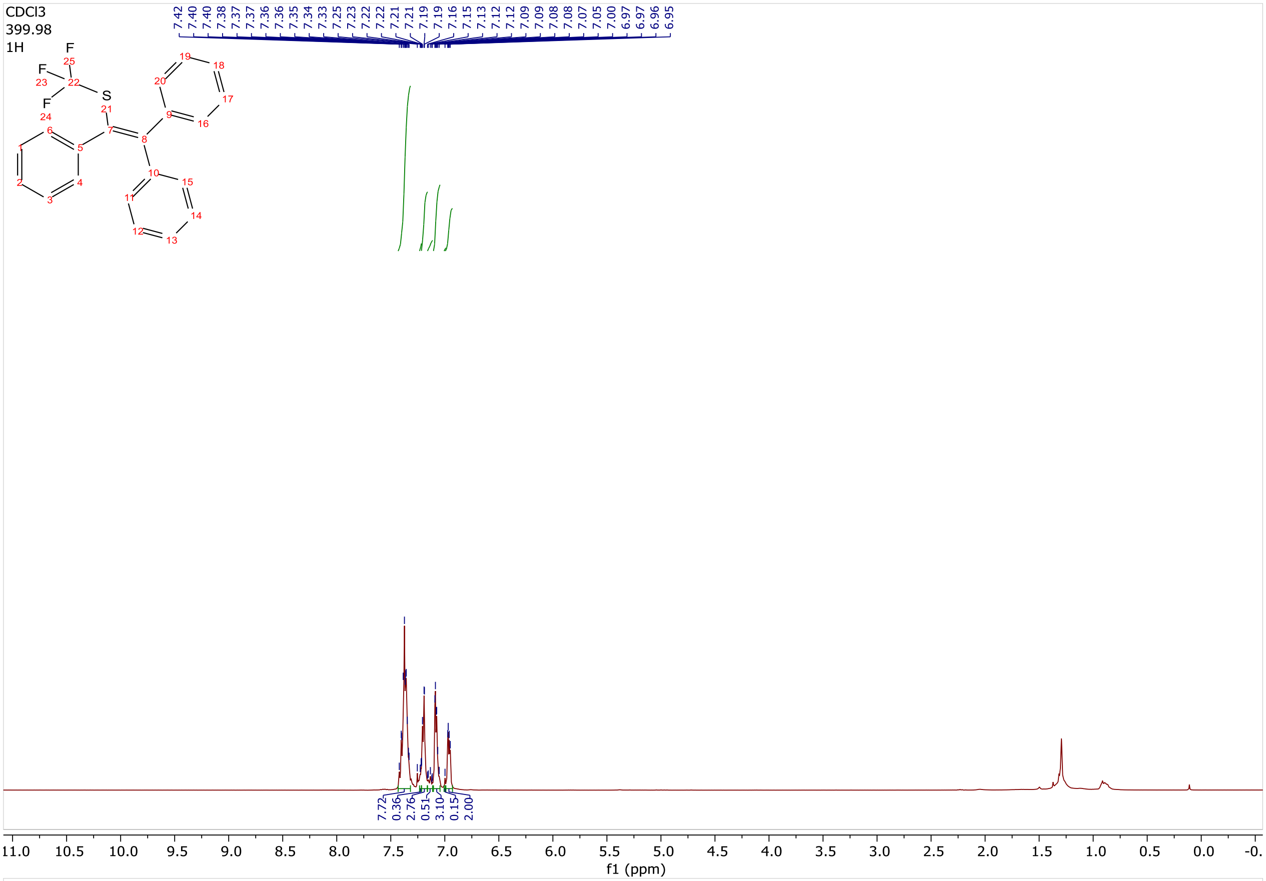

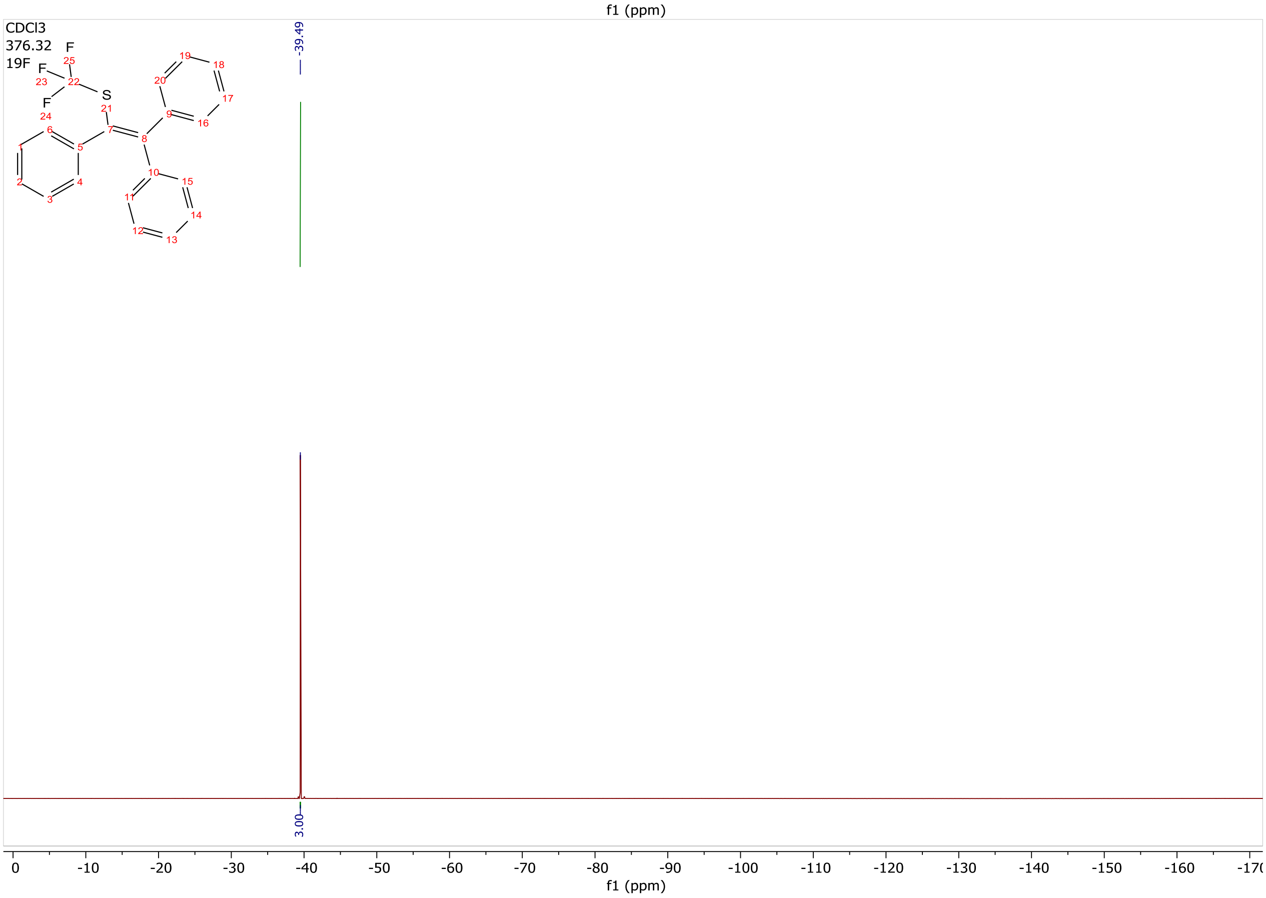

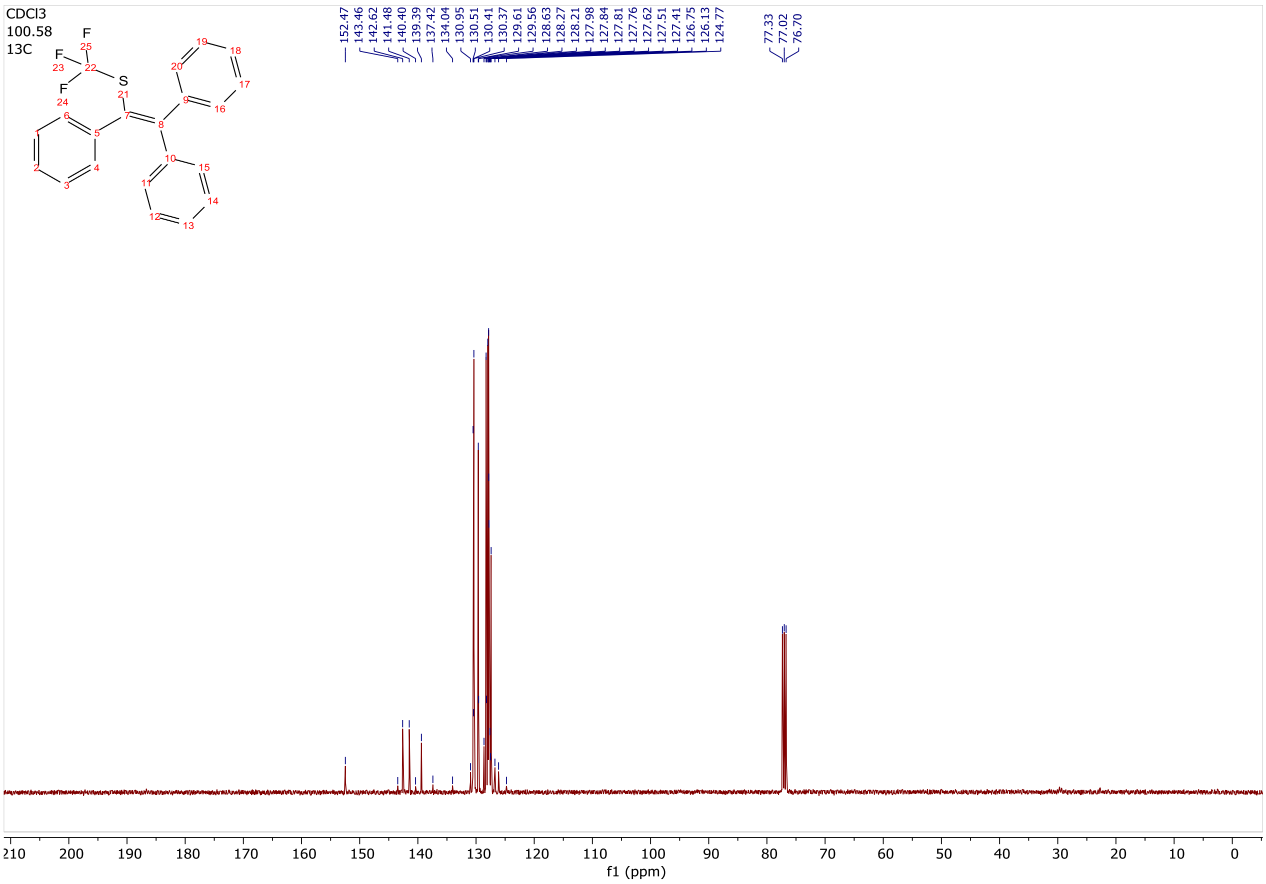
CDCl3
100.58
13C
152.47
143.46
142.62
141.48
140.40
139.39
137.42
134.04
130.95
130.51
130.41
130.37
129.61
129.56
128.63
128.27
128.21
127.98
127.84
127.81
127.76
127.62
127.51
127.41
126.75
126.13
124.77
77.33
77.02
76.70
f1 (ppm)

(*E*)-(2-(4-methoxyphenyl)-1,2-diphenylvinyl)(trifluoromethyl)sulfane (3r) (400 MHz [^{1}H];101 MHz [^{13}C], 376 MHz [^{19}F] $CDCl_3$)

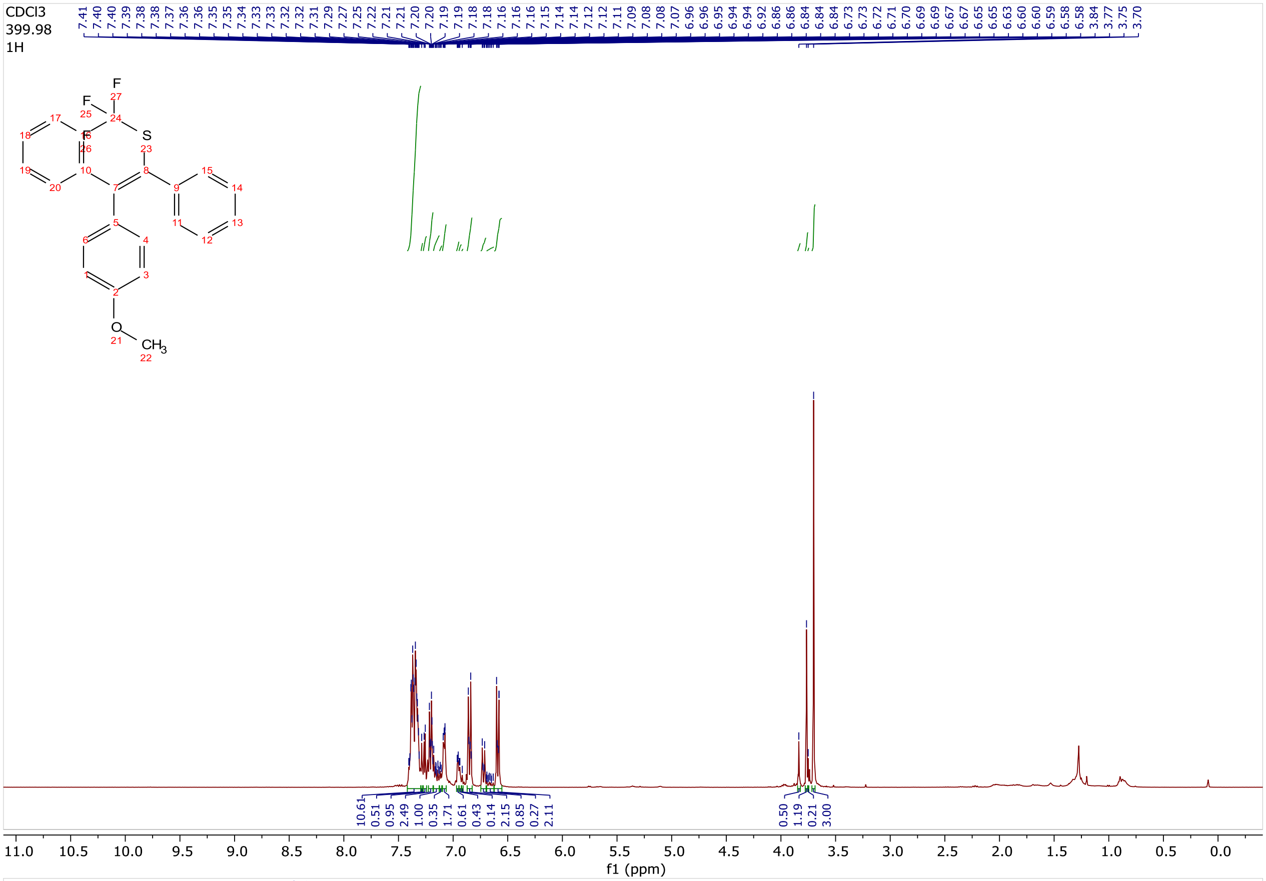

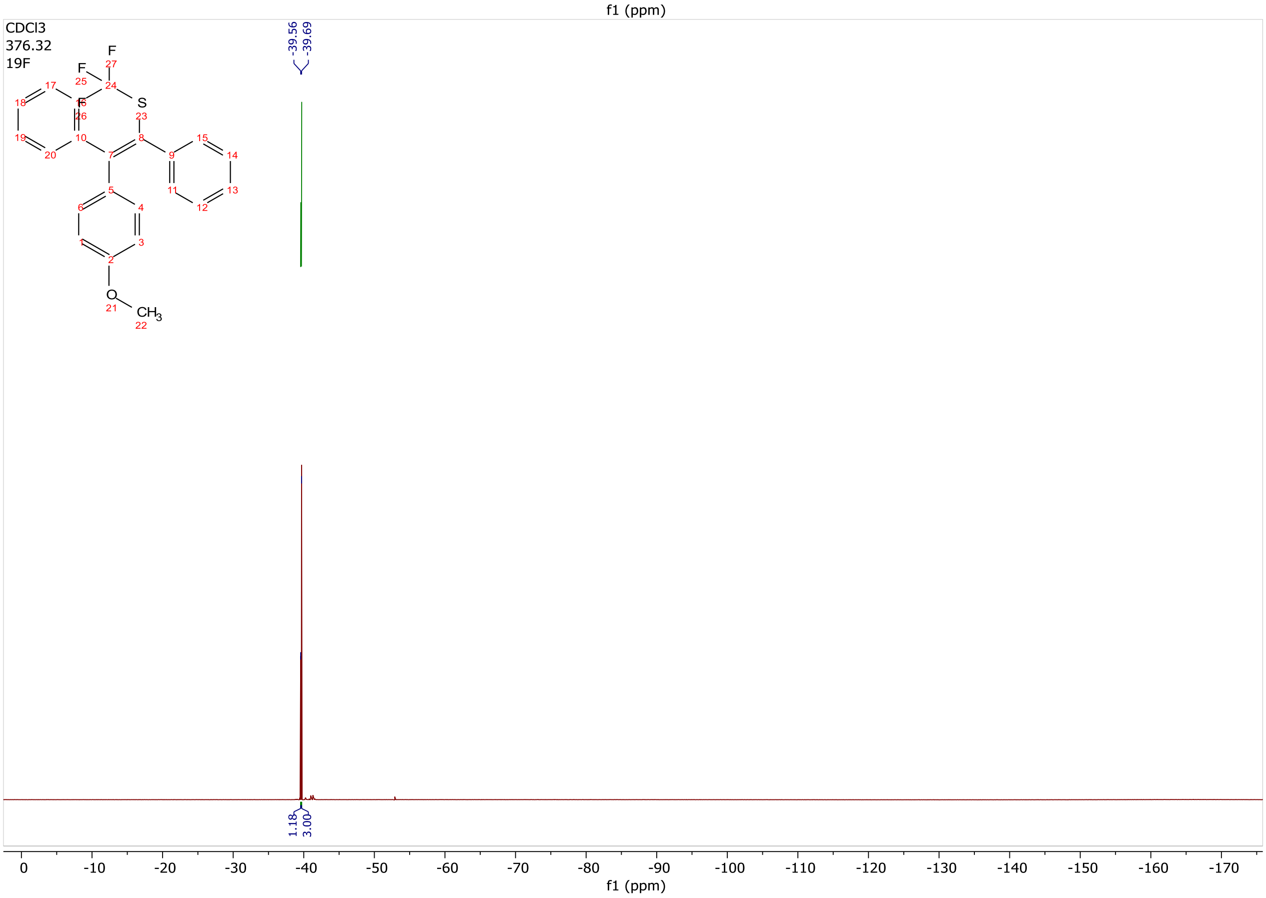

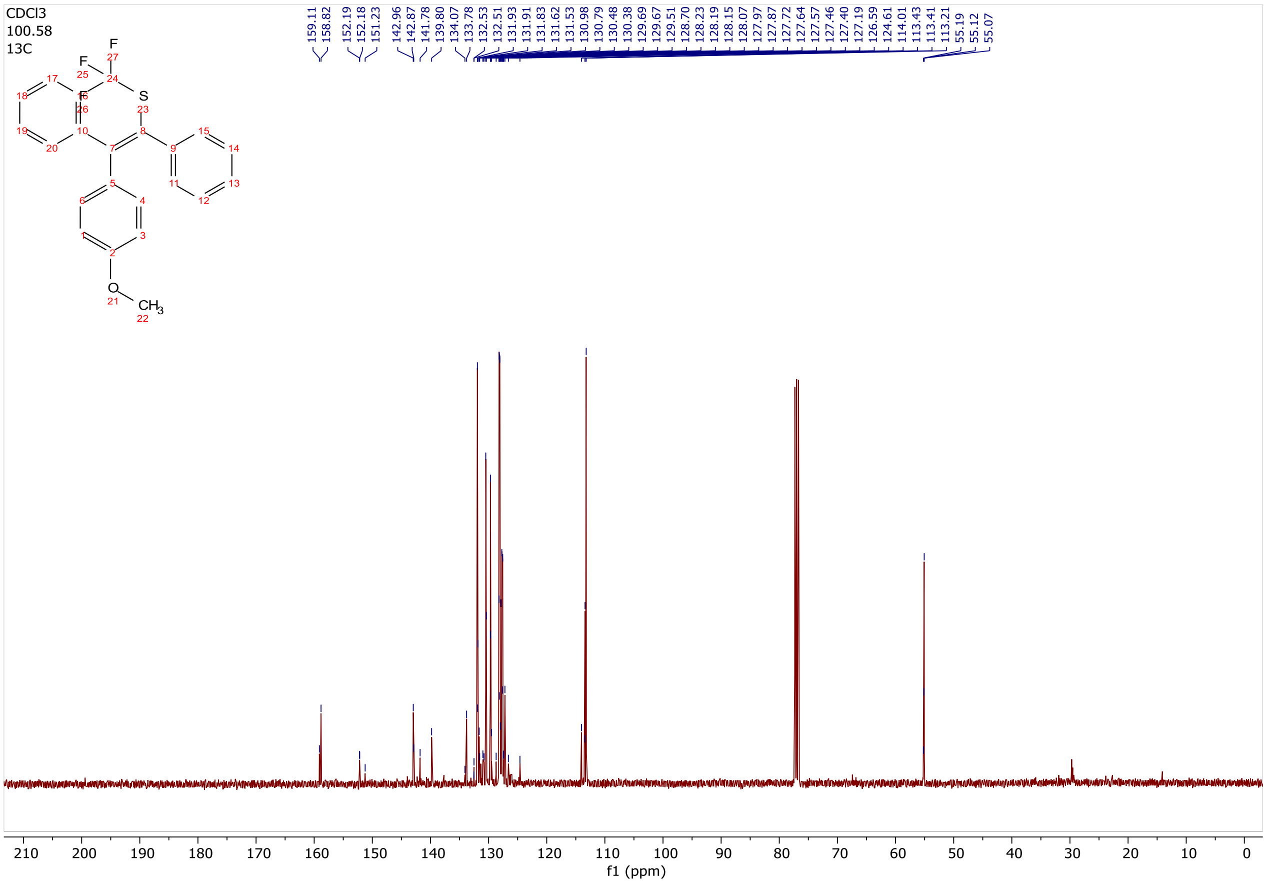
CDCl3
100.58
13C
159.11
158.82
152.19
152.18
151.23
142.96
142.87
141.78
139.80
134.07
133.78
132.53
132.51
131.93
131.91
131.83
131.62
131.53
130.98
130.79
130.48
130.38
129.69
129.67
129.51
128.70
128.23
128.19
128.15
128.07
127.97
127.87
127.72
127.64
127.57
127.46
127.40
127.19
126.59
124.61
114.01
113.43
113.41
113.21
55.19
55.12
55.07
f1 (ppm)

(1-(Naphthalen-2-yl)-2,2-diphenylvinyl)(trifluoromethyl)sulfane (3s) (400 MHz [^{1}H];101 MHz [^{13}C], 376 MHz [^{19}F] $CDCl_3$)

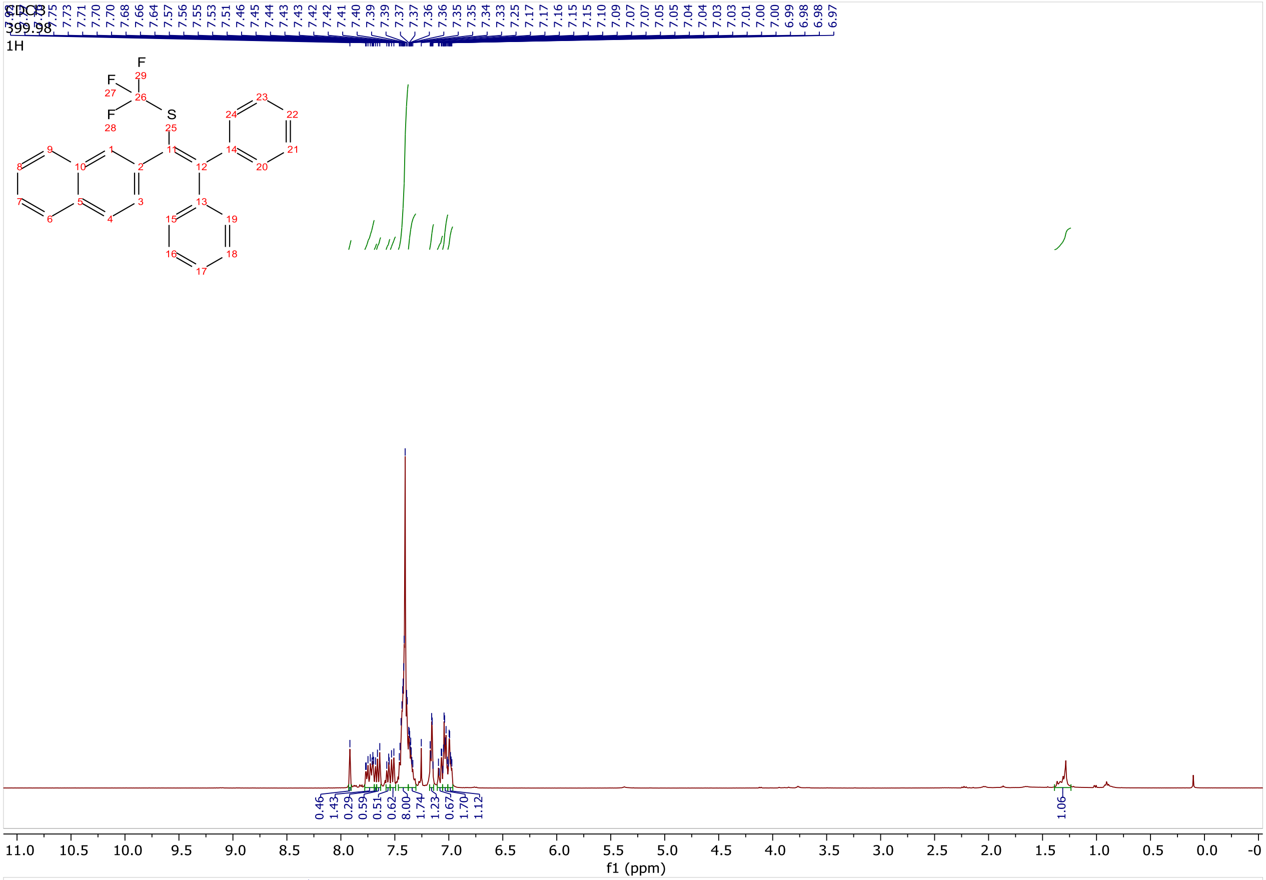

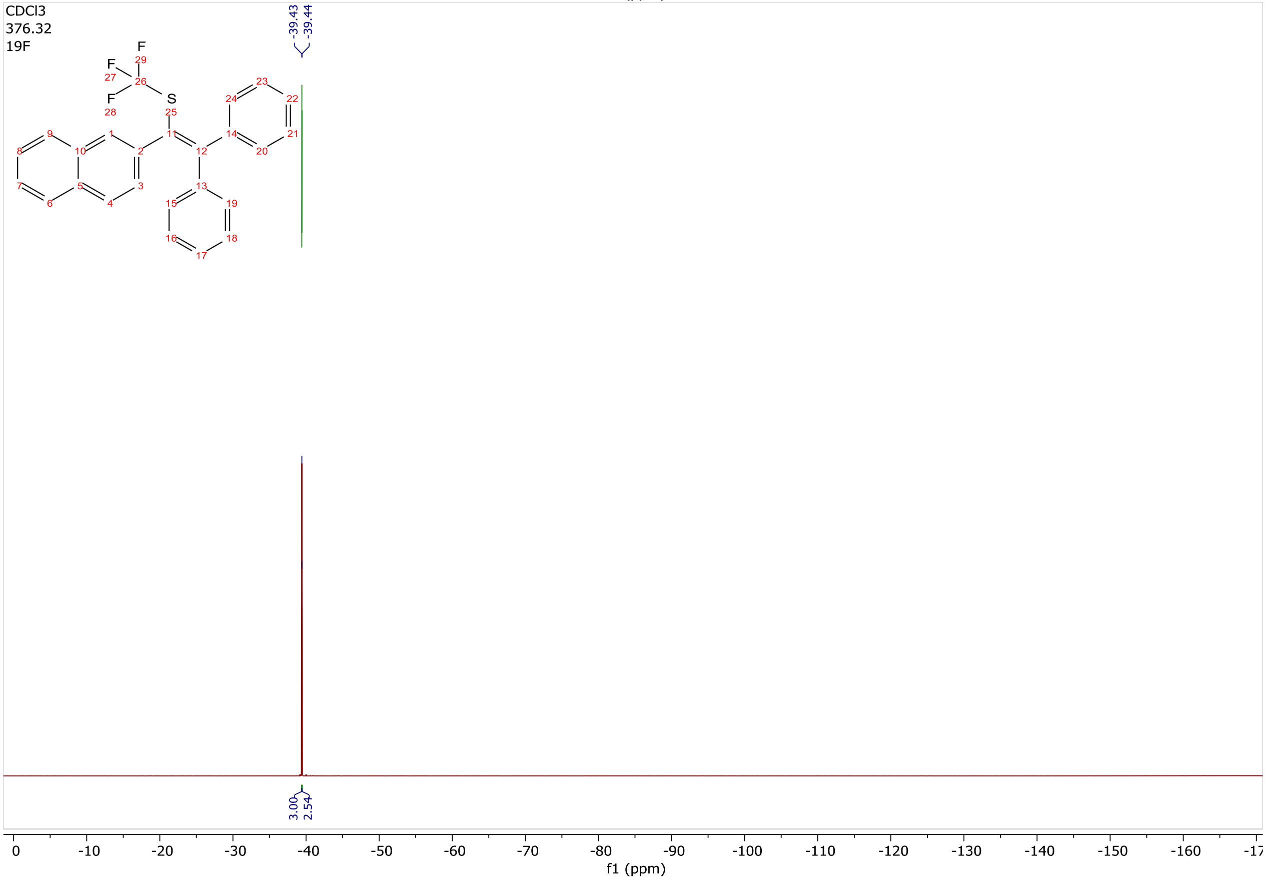

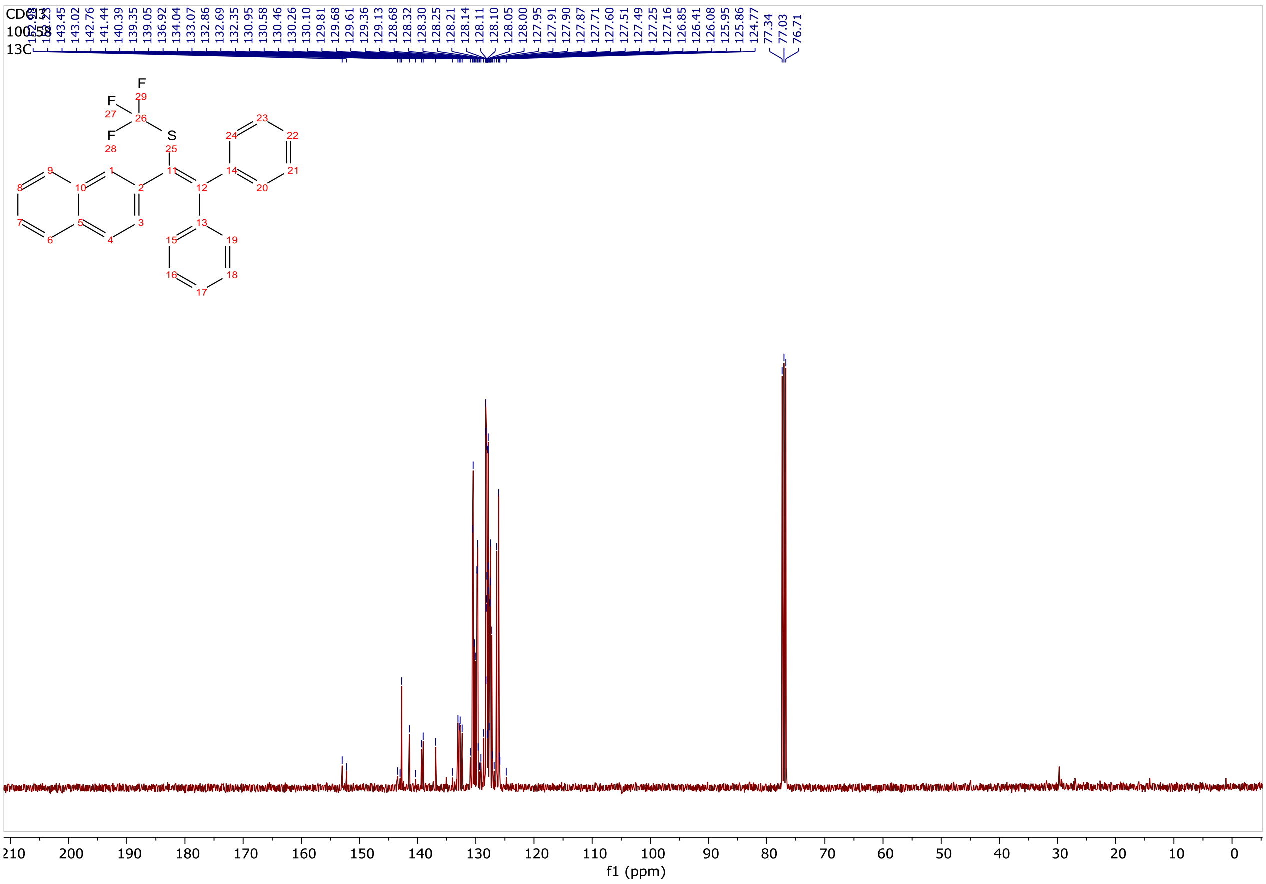

(1-(Naphthalen-1-yl)-2,2-diphenylvinyl)(trifluoromethyl)sulfane (3t) (400 MHz [^{1}H];101 MHz [^{13}C], 376 MHz [^{19}F] $CDCl_3$)

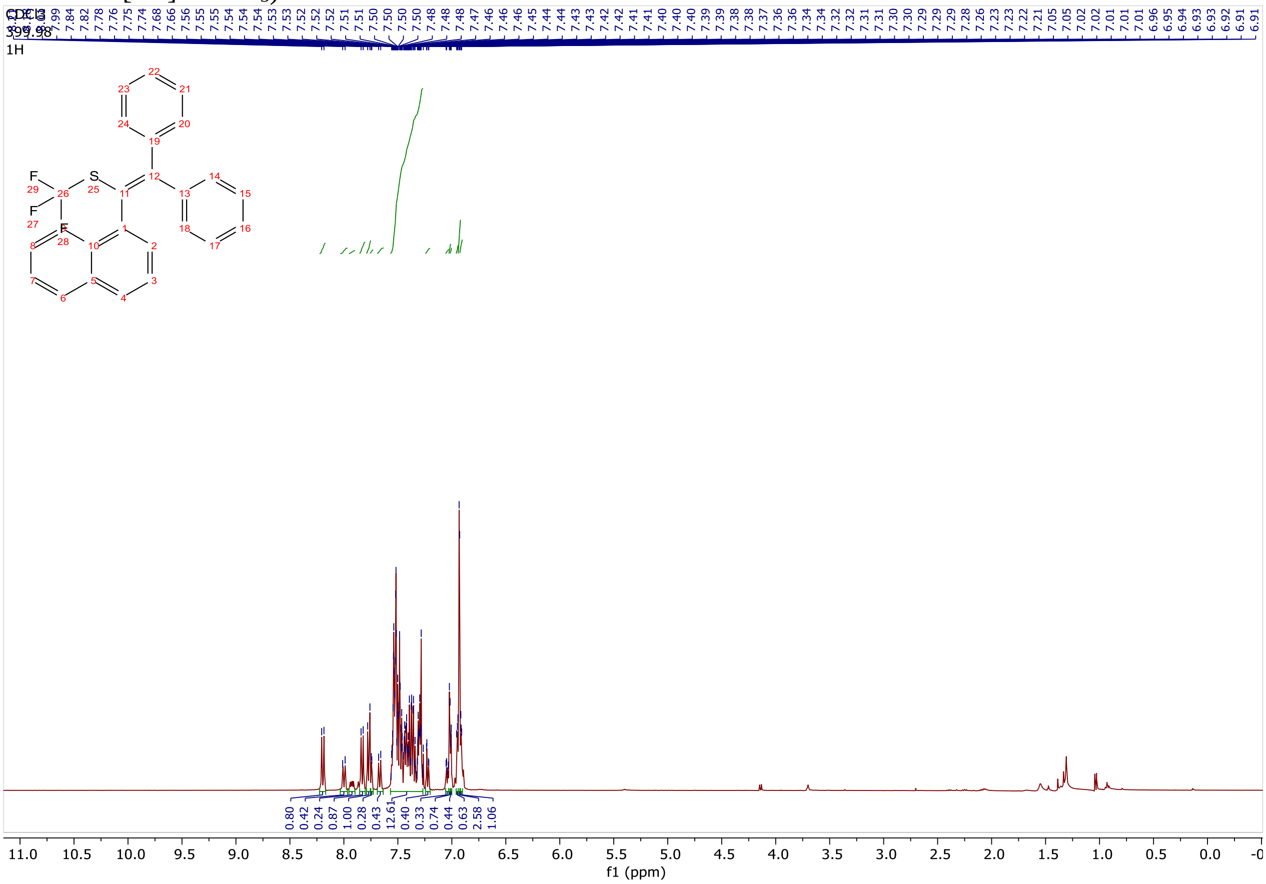

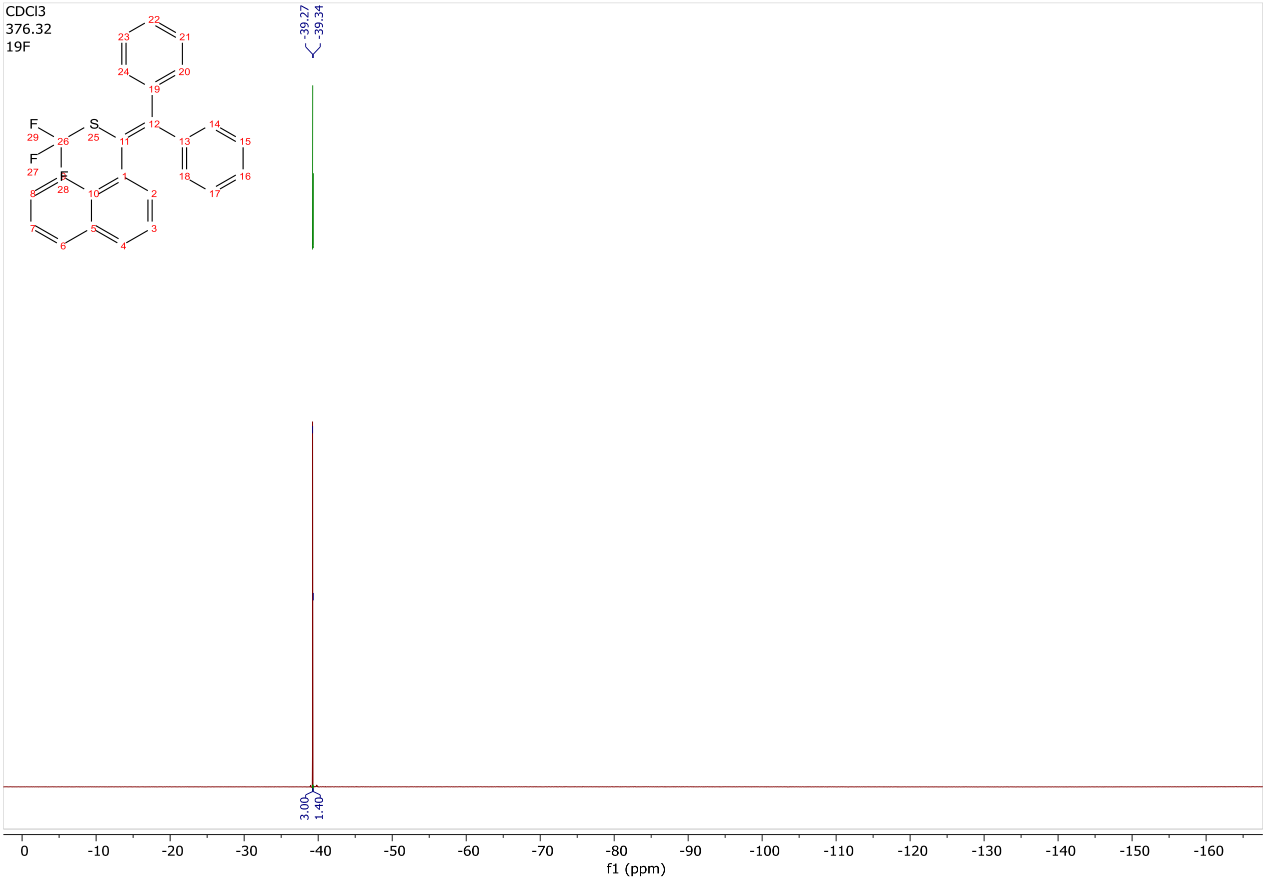

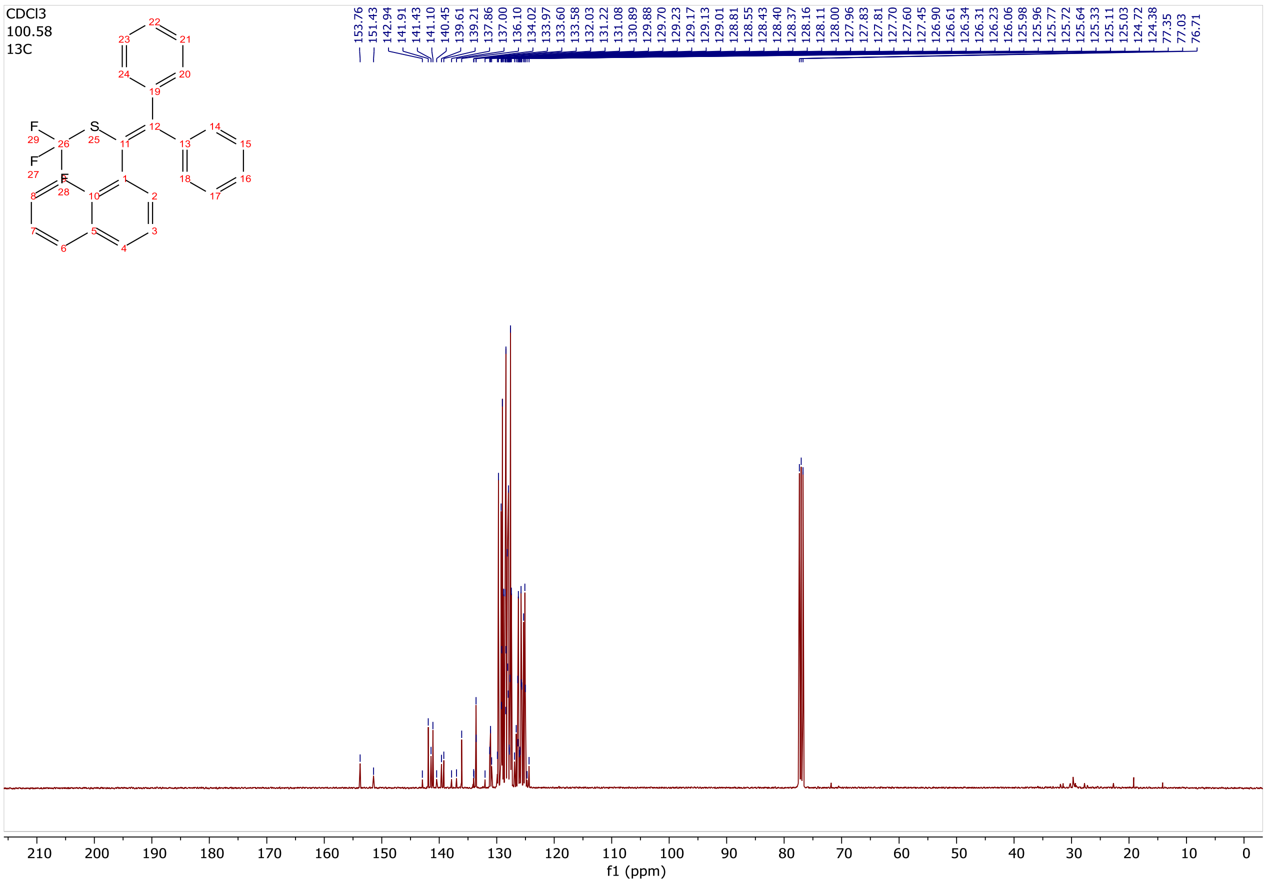
CDCl3
100.58
13C
153.76 151.43 142.94 141.91 141.43 141.10 140.45 139.61 139.21 137.86 137.00 136.10 134.02 133.97 133.60 133.58 132.03 131.22 131.08 130.89 129.88 129.70 129.23 129.17 129.13 129.01 128.81 128.55 128.43 128.40 128.37 128.16 128.11 128.00 127.96 127.83 127.81 127.70 127.60 127.45 126.90 126.61 126.34 126.31 126.23 126.06 125.98 125.96 125.77 125.72 125.64 125.33 125.11 125.03 124.72 124.38 77.35 77.03 76.71
f1 (ppm)

(Z)-(2-(4-methoxyphenyl)-1-phenylhex-1-en-1-yl)(trifluoromethyl)sulfane (3u) (400 MHz [^{1}H];101 MHz [^{13}C], 376 MHz [^{19}F] $CDCl_3$)

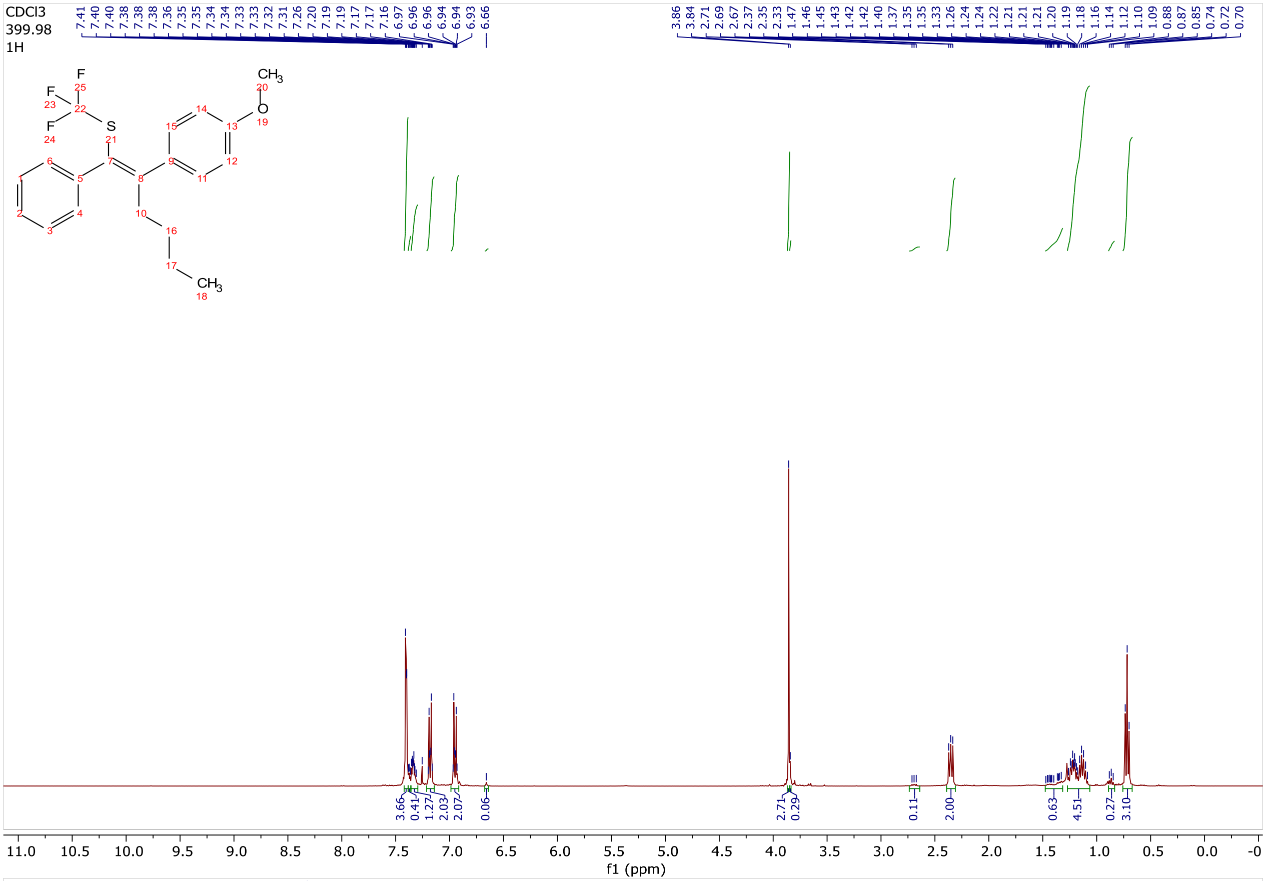

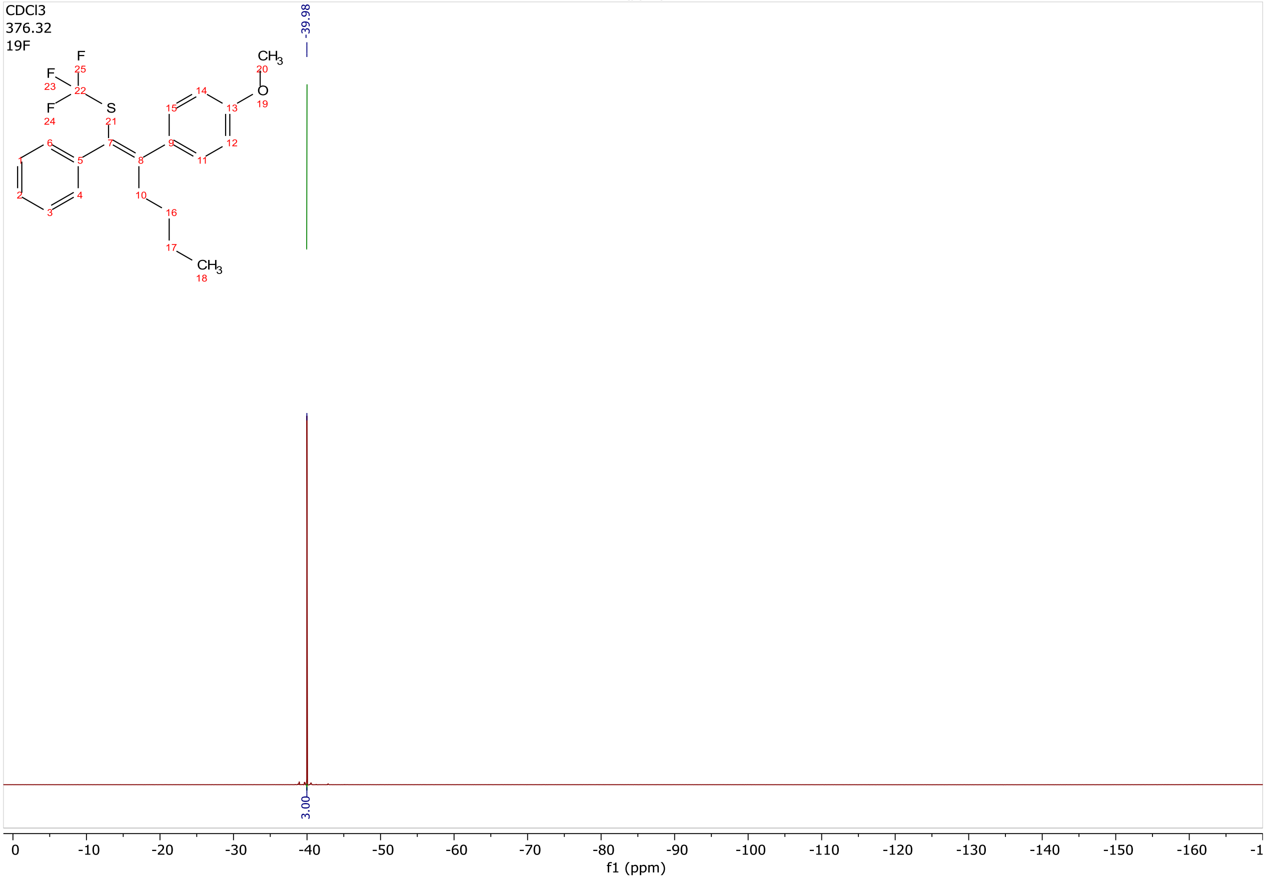

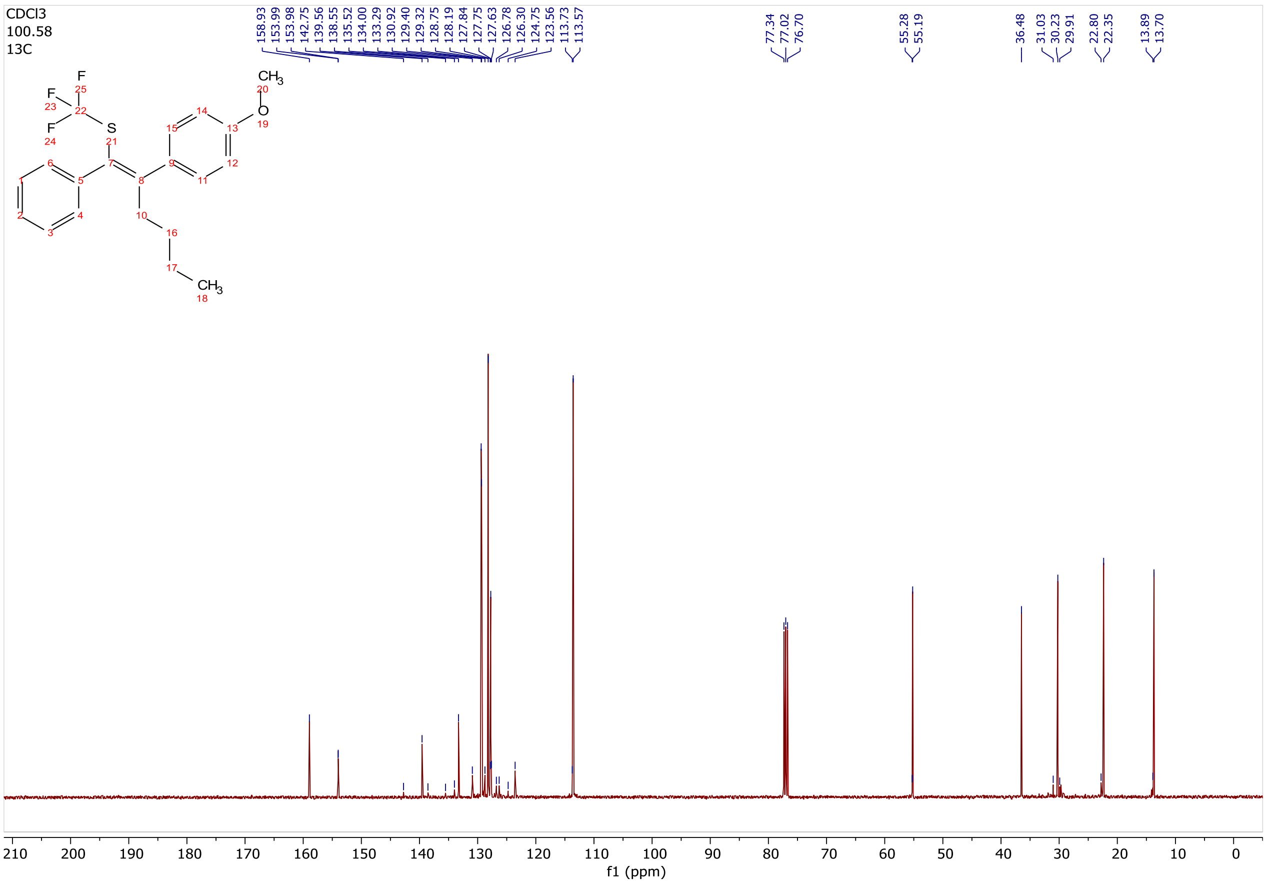
CDCl3
100.58
13C
158.93
153.99
153.98
142.75
139.56
138.55
135.52
134.00
133.29
130.92
129.40
129.32
128.75
128.19
127.84
127.75
127.63
126.78
126.30
124.75
123.56
113.73
113.57
77.34
77.02
76.70
55.28
55.19
36.48
31.03
30.23
29.91
22.80
22.35
13.89
13.70
f1 (ppm)

(Z)-(2-(4-Fluorophenyl)-1-phenylhex-1-en-1-yl)(trifluoromethyl)sulfane (3v) (400 MHz [^{1}H];101 MHz [^{13}C], 376 MHz [^{19}F] $CDCl_3$)

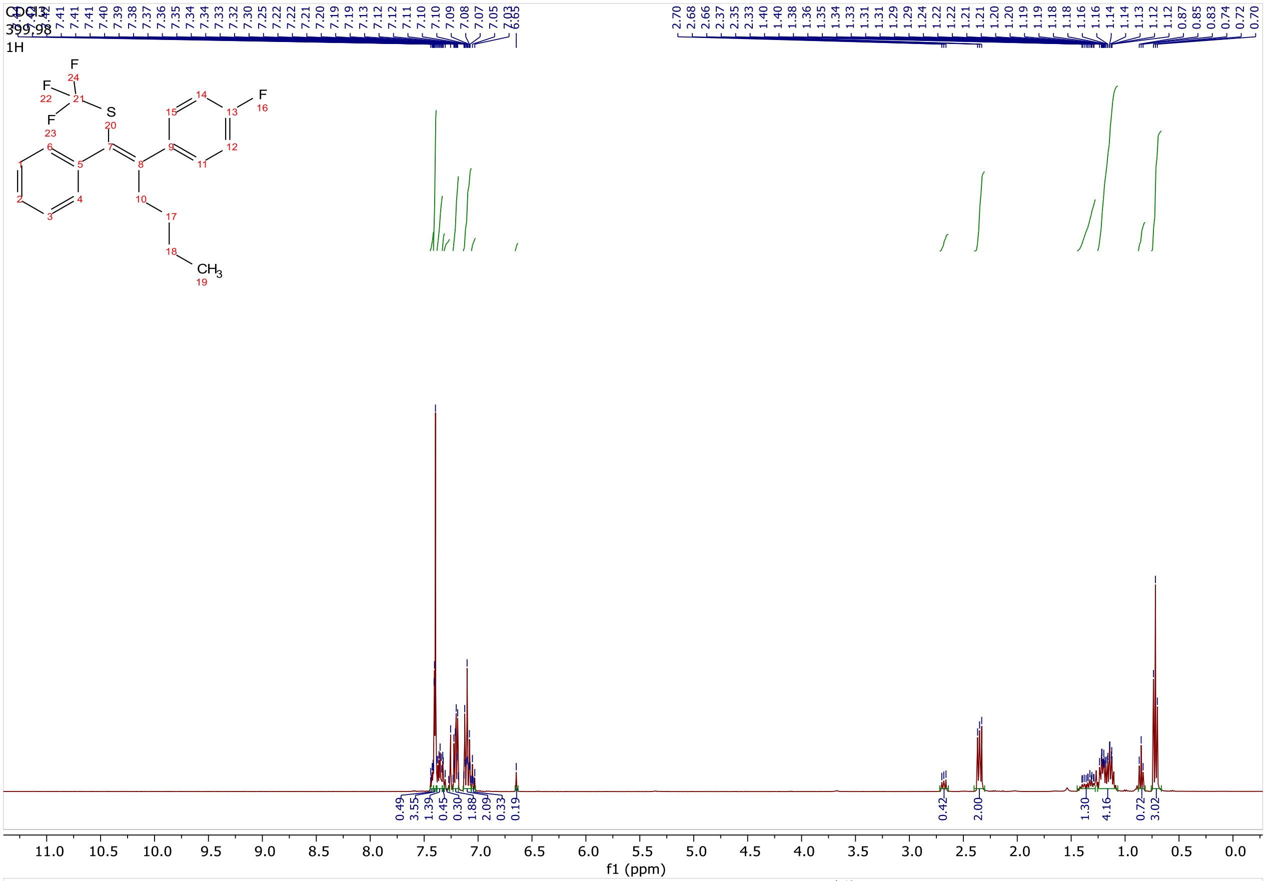

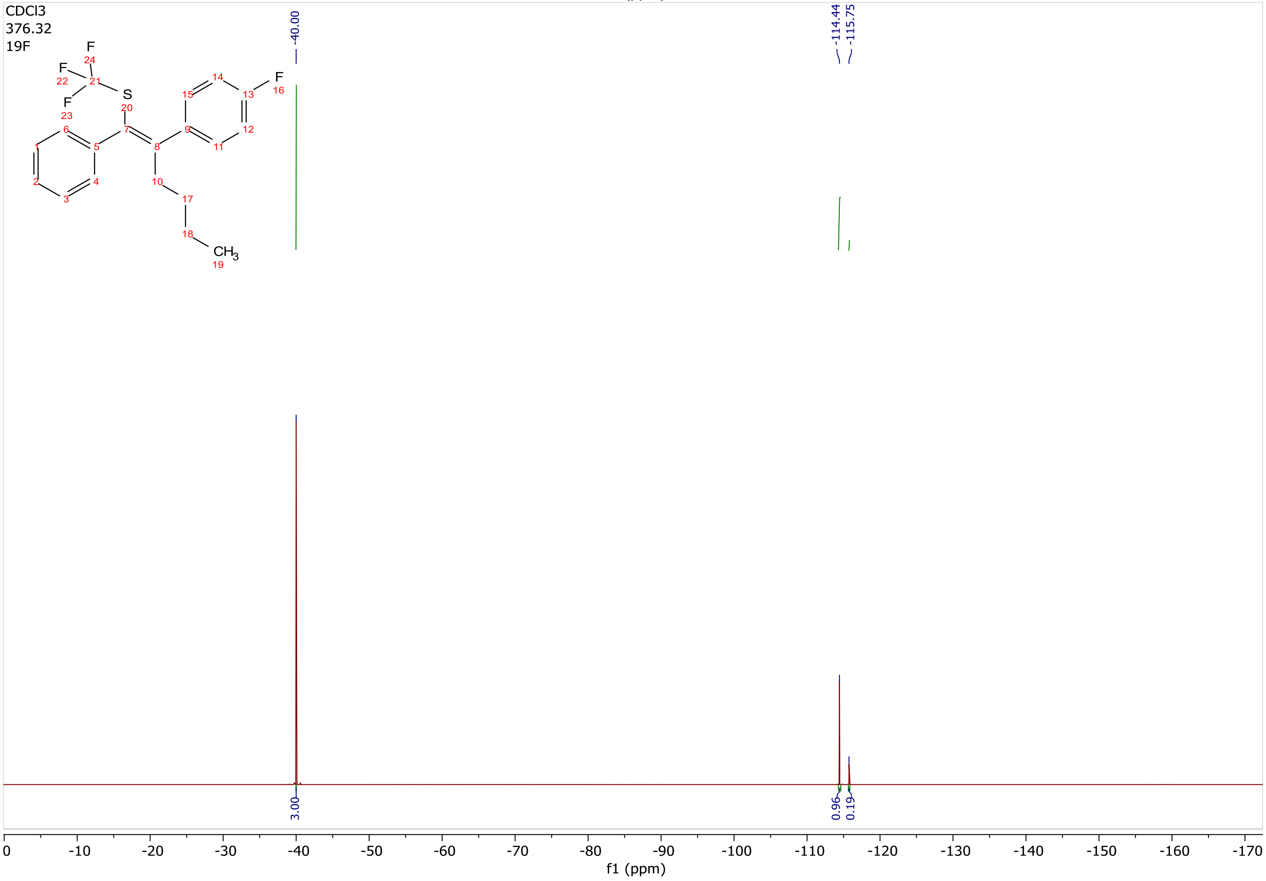

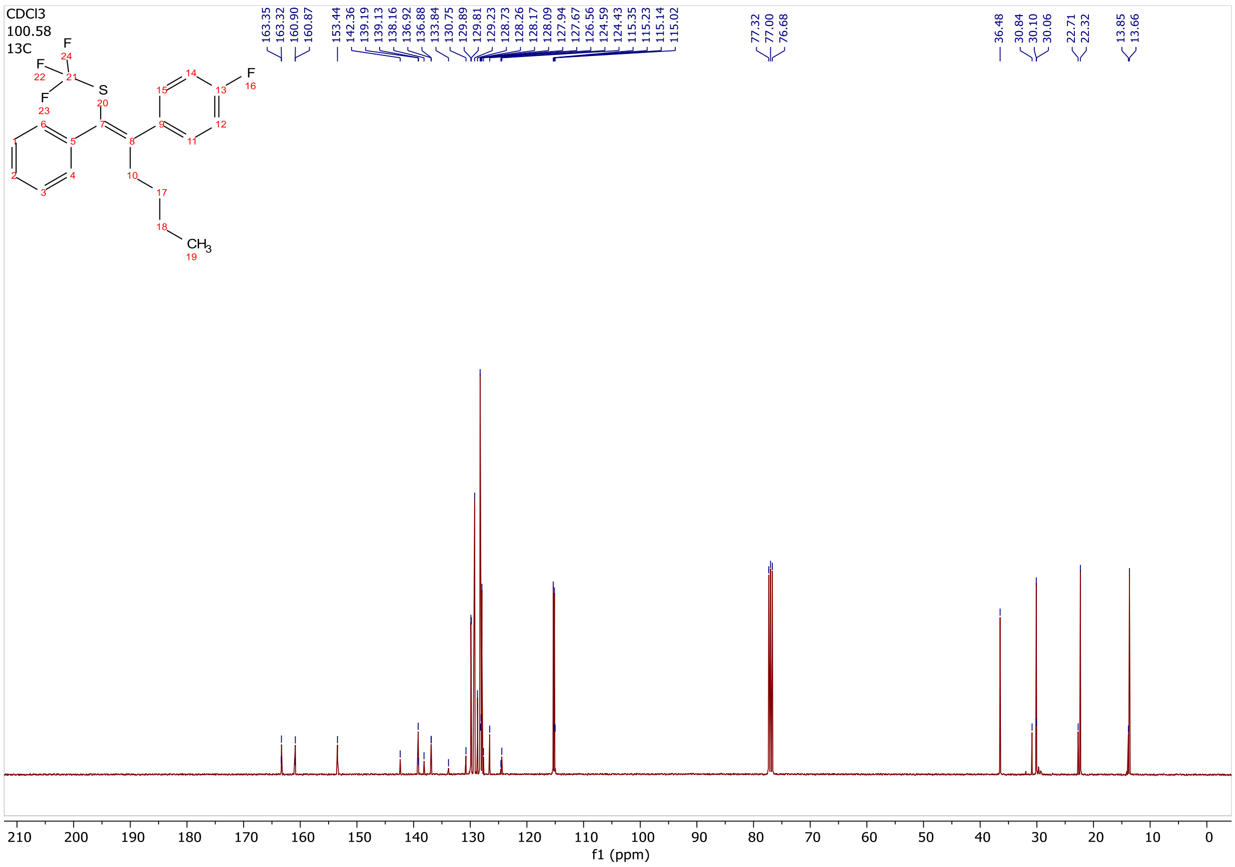
CDCl3
100.58
13C
163.35
163.32
160.90
160.87
153.44
142.36
139.19
139.13
138.16
136.92
136.88
133.84
130.75
129.89
129.81
129.23
128.73
128.26
128.17
128.09
127.94
127.67
126.56
124.59
124.43
115.35
115.23
115.14
115.02
77.32
77.00
76.68
36.48
30.84
30.10
30.06
22.71
22.32
13.85
13.66
f1 (ppm)

(Z)-(1-Phenyl-2-(p-tolyl)hex-1-en-1-yl)(trifluoromethyl)sulfane (3w) (400 MHz [^{1}H];101 MHz [^{13}C], 376 MHz [^{19}F] $CDCl_3$)

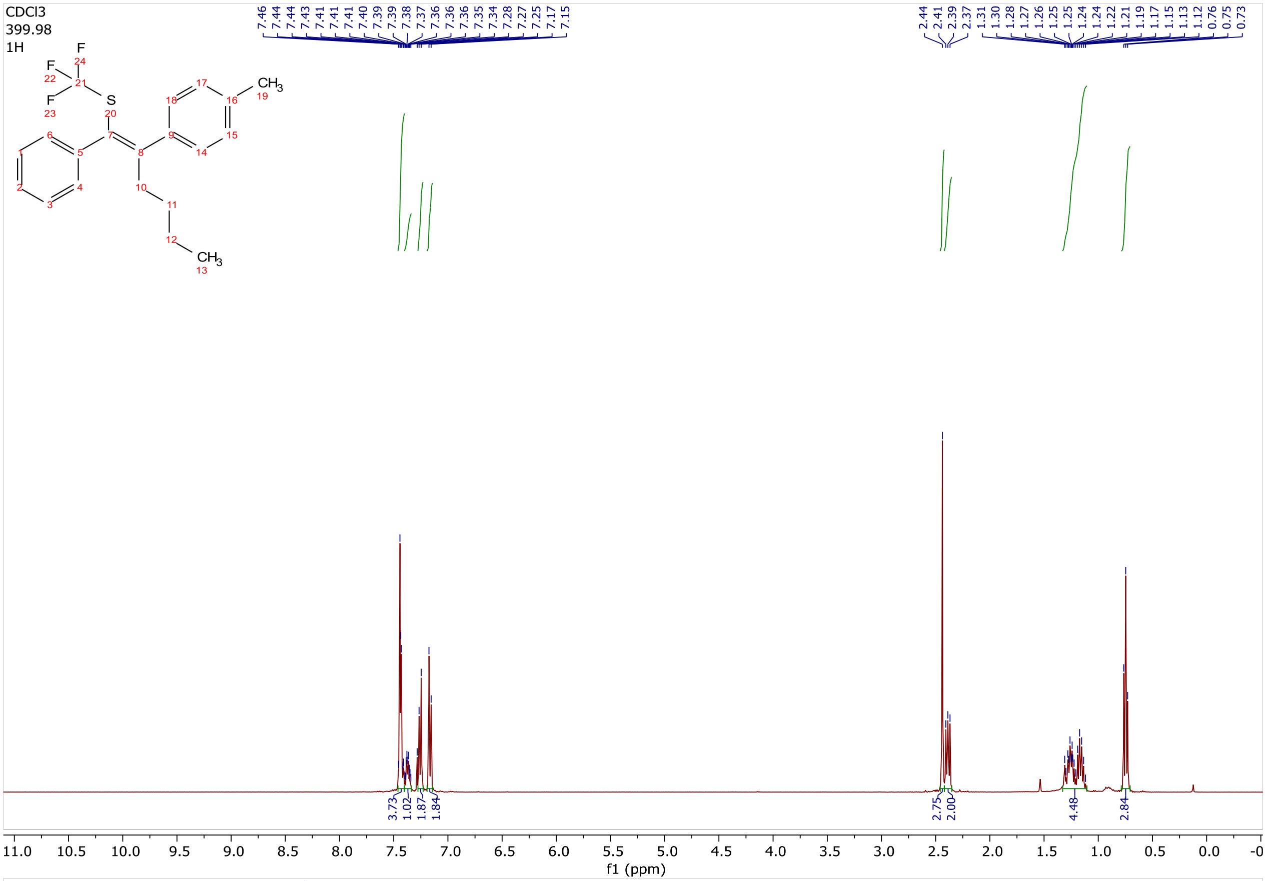

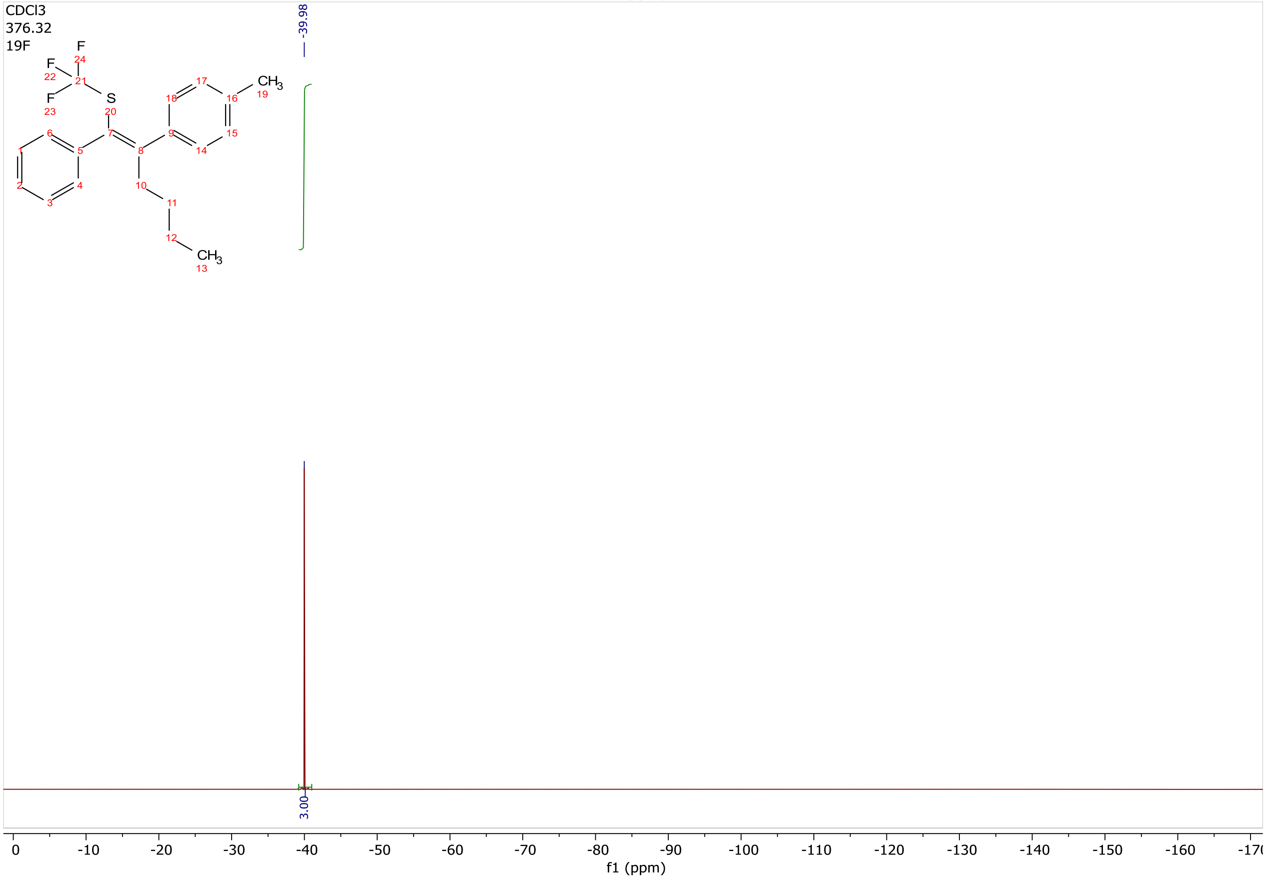

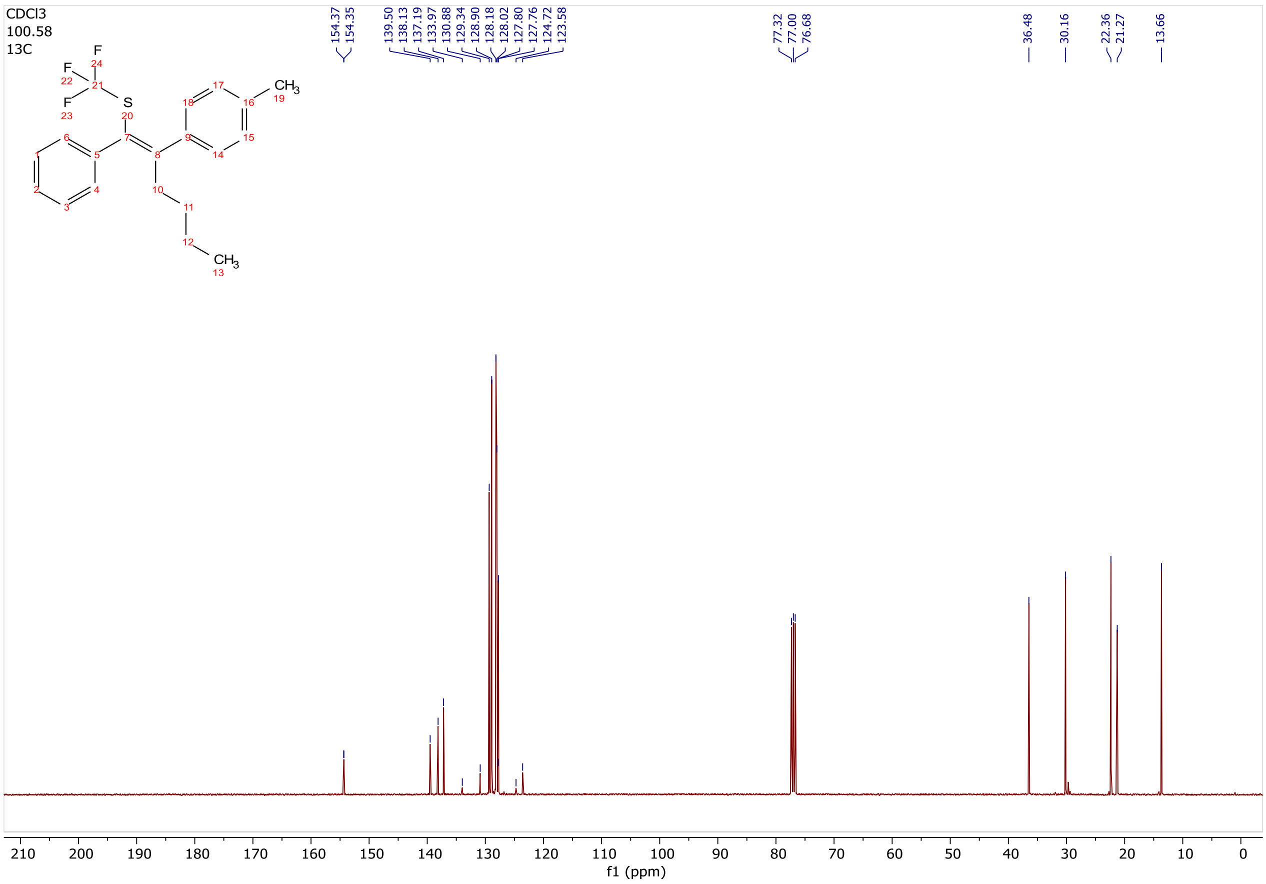
CDCl3
100.58
13C
154.37
154.35
139.50
138.13
137.19
133.97
130.88
129.34
128.90
128.18
128.02
127.80
127.76
124.72
123.58
77.32
77.00
76.68
36.48
30.16
22.36
21.27
13.66
f1 (ppm)

(Z)-(1-Phenyl-2-(o-tolyl)hex-1-en-1-yl)(trifluoromethyl)sulfane (3x) (400 MHz [^{1}H];101 MHz [^{13}C], 376 MHz [^{19}F] $CDCl_3$)

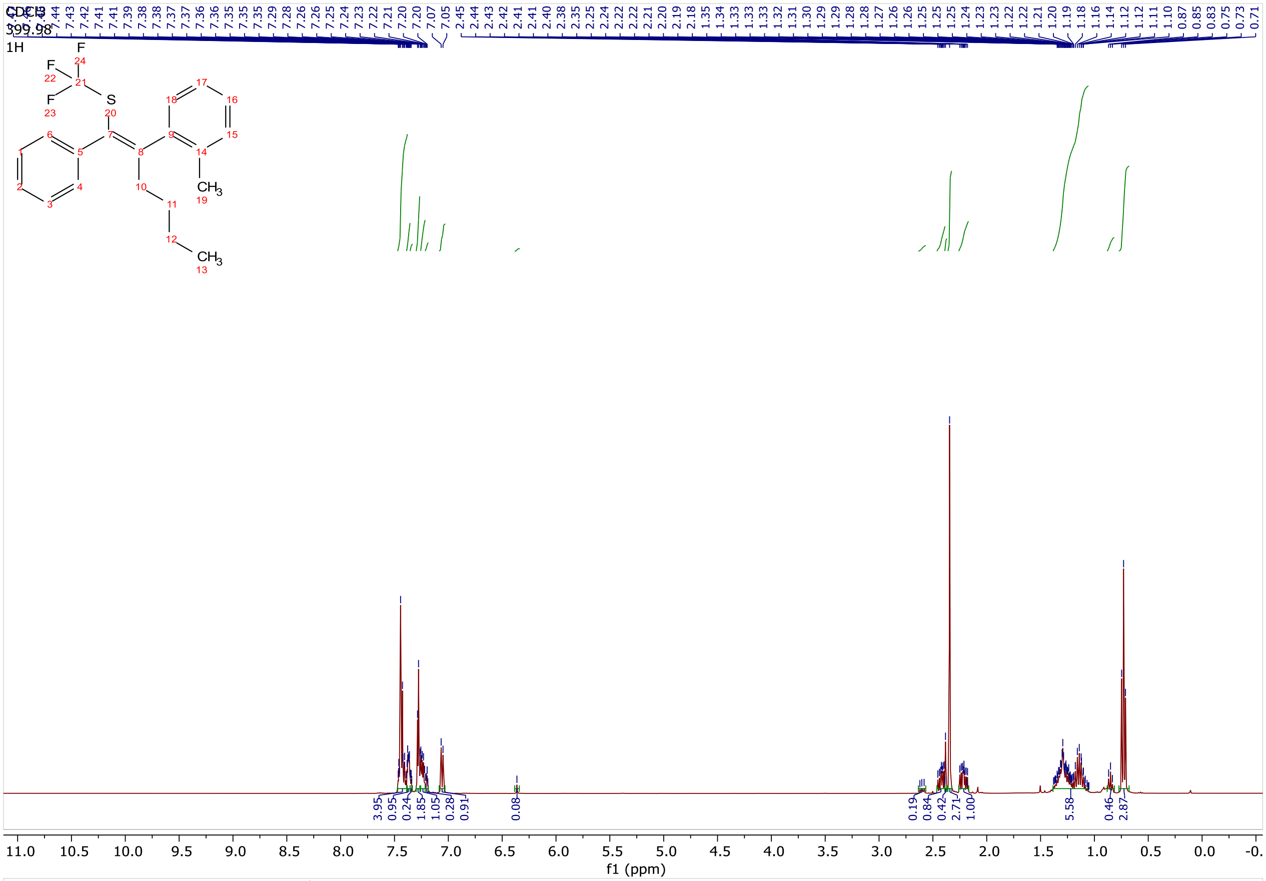

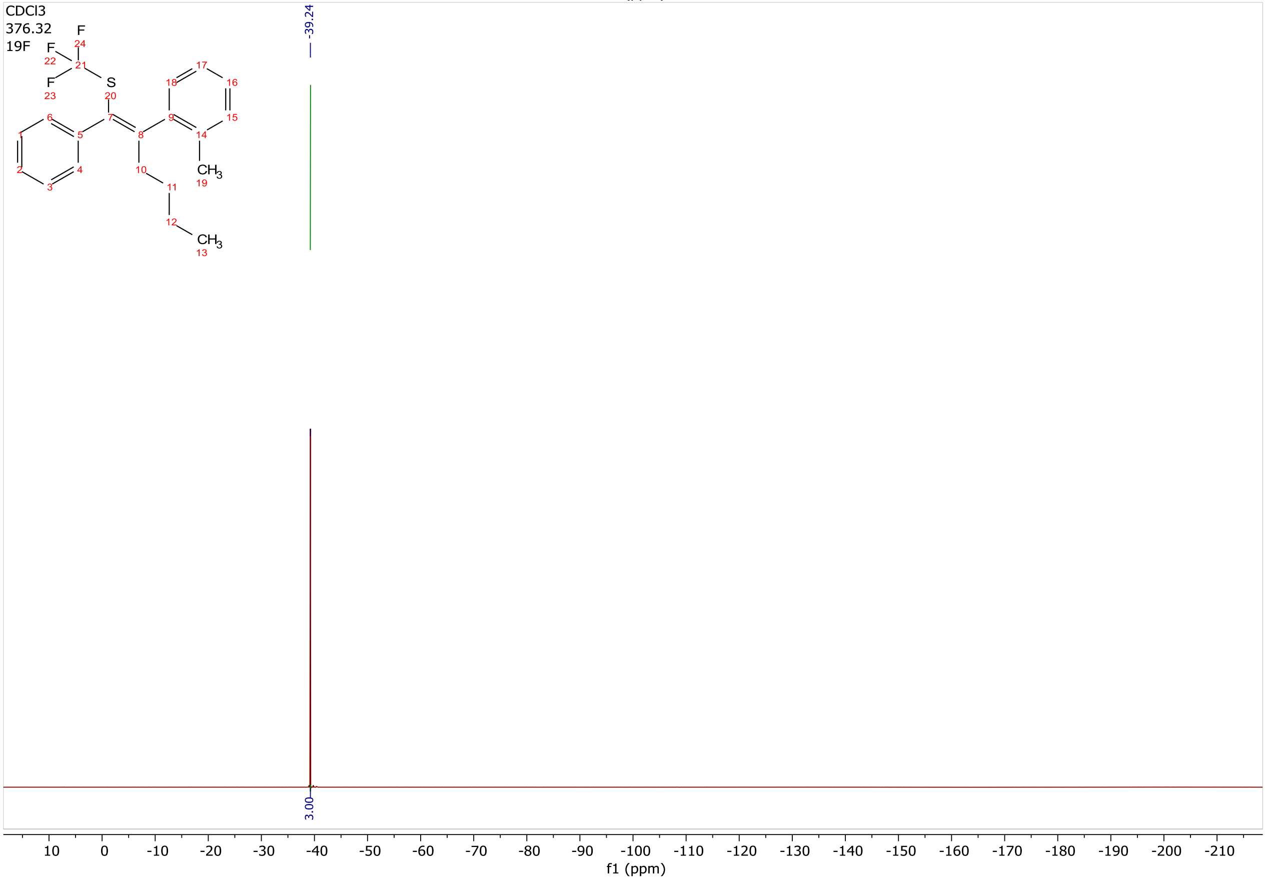

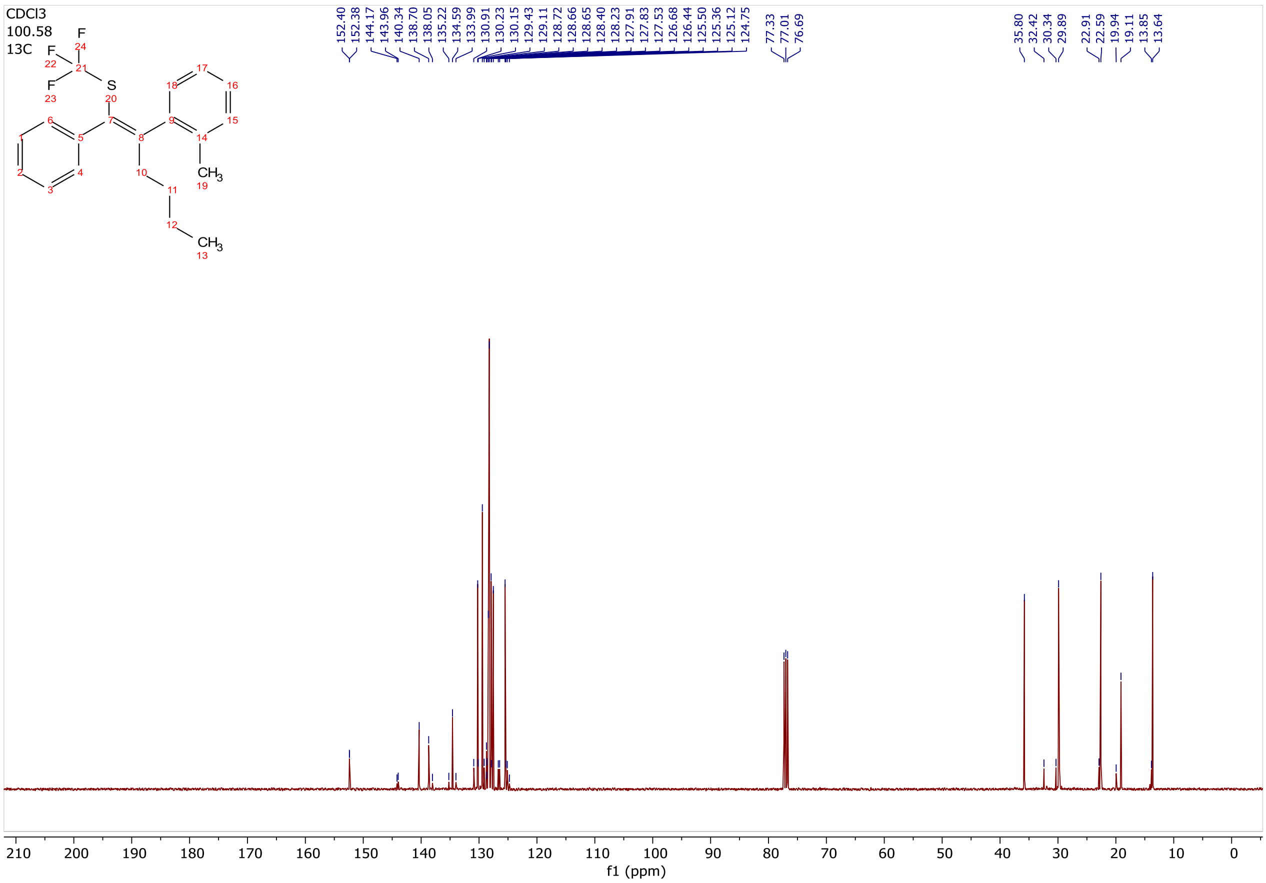
CDCl3
100.58
13C
152.40
152.38
144.17
143.96
140.34
138.70
138.05
135.22
134.59
133.99
130.91
130.23
130.15
129.43
129.11
128.72
128.66
128.65
128.40
128.23
127.91
127.83
127.53
126.68
126.44
125.50
125.36
125.12
124.75
77.33
77.01
76.69
35.80
32.42
30.34
29.89
22.91
22.59
19.94
19.11
13.85
13.64
f1 (ppm)

(Z)-(2-Mesityl-1-phenylhex-1-en-1-yl)(trifluoromethyl)sulfane (3y) (400 MHz [^{1}H];101 MHz [^{13}C], 376 MHz [^{19}F] $CDCl_3$)

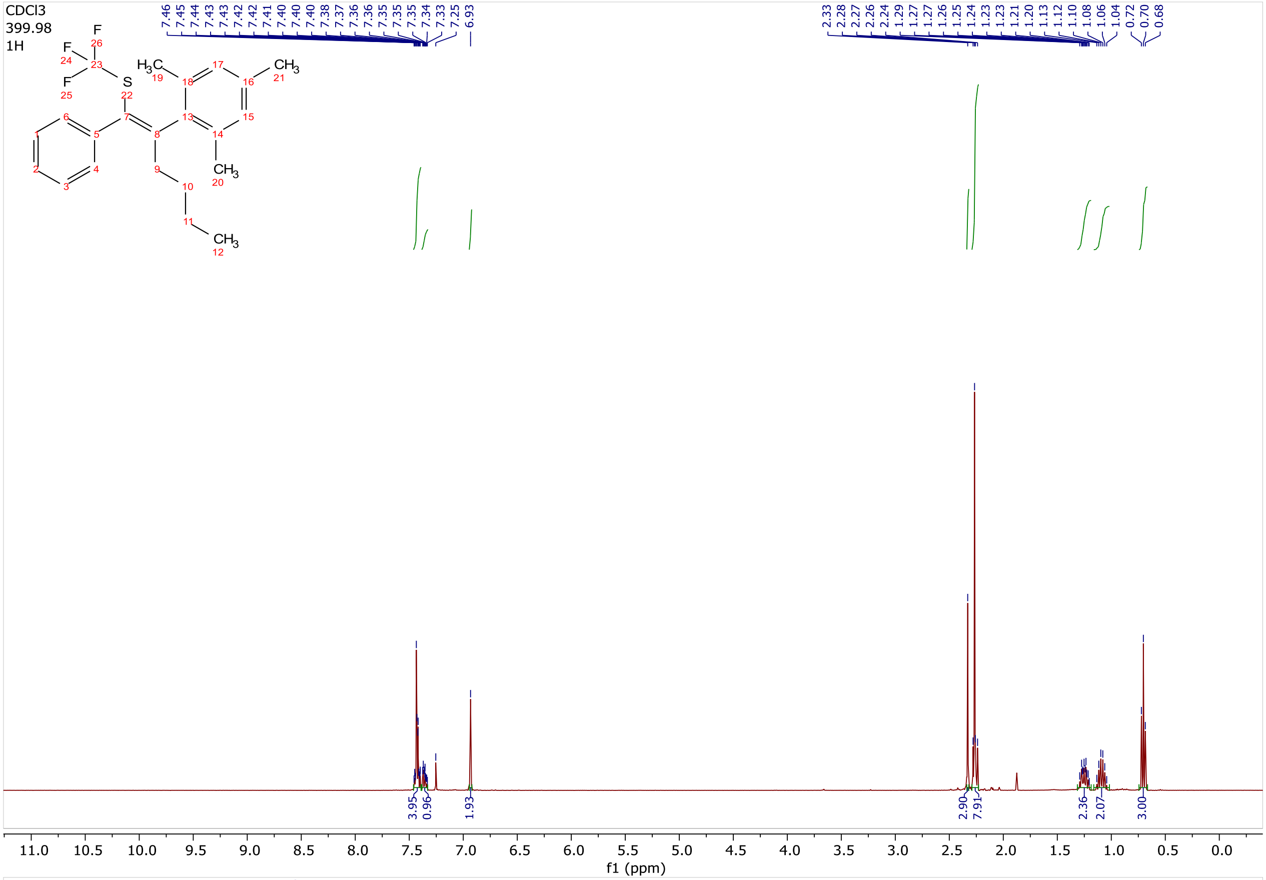

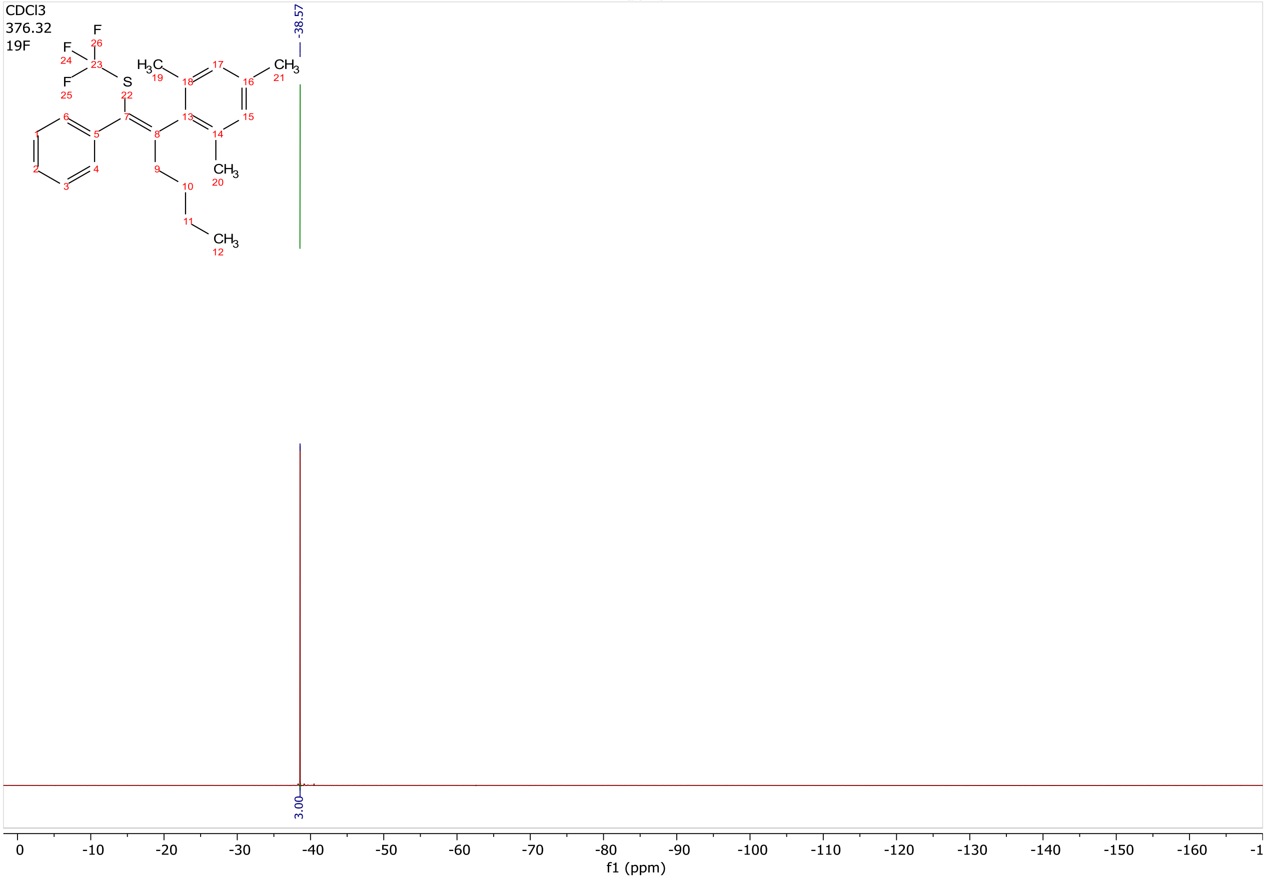

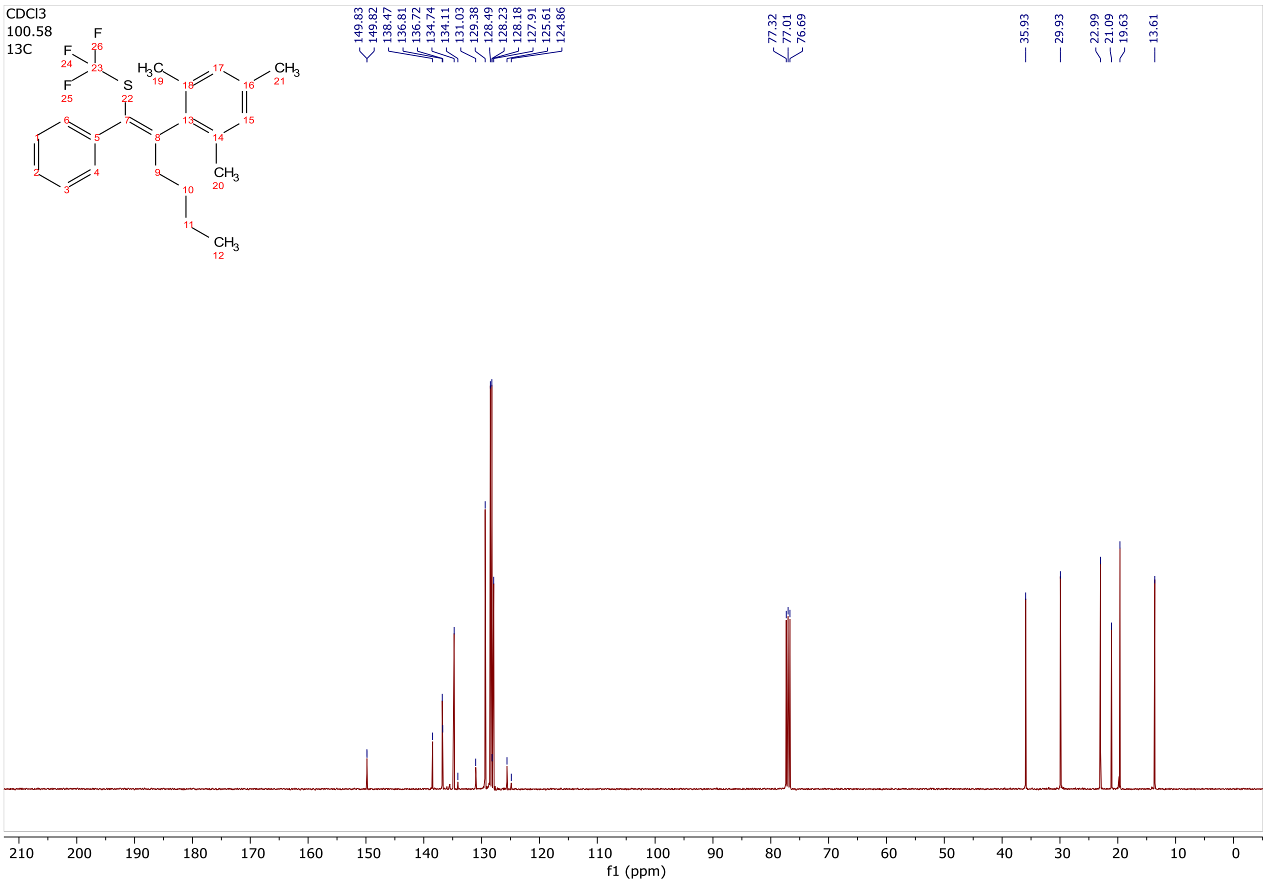

CDCl3
100.58
13C
149.83
149.82
138.47
136.81
136.72
134.74
134.11
131.03
129.38
128.49
128.23
128.18
127.91
125.61
124.86
77.32
77.01
76.69
35.93
29.93
22.99
21.09
19.63
13.61
f1 (ppm)

((Z)-1-Phenyl-2-((E)-prop-1-en-1-yl)hex-1-en-1-yl)(trifluoromethyl)sulfane (3z) (400 MHz [^{1}H];101 MHz [^{13}C], 376 MHz [^{19}F] $CDCl_3$)

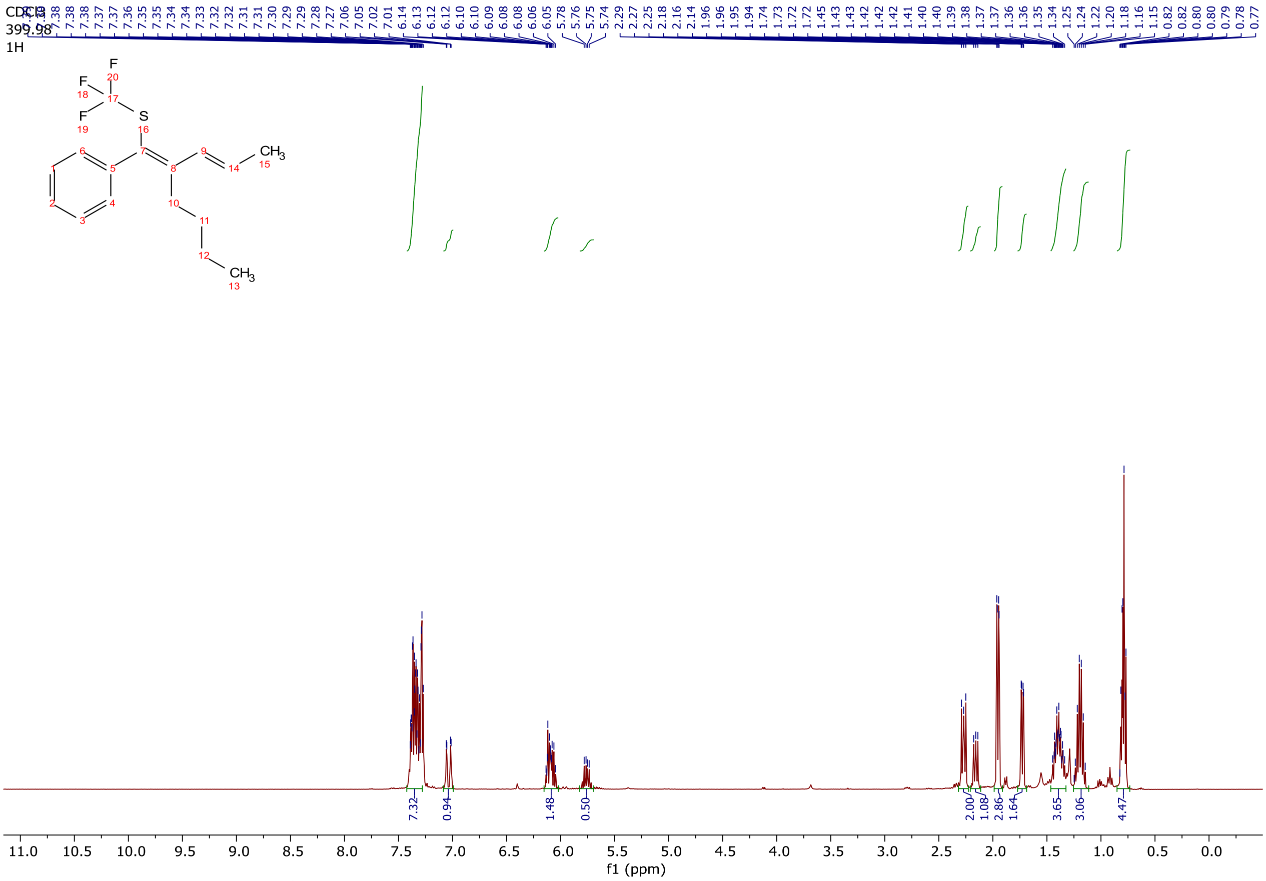

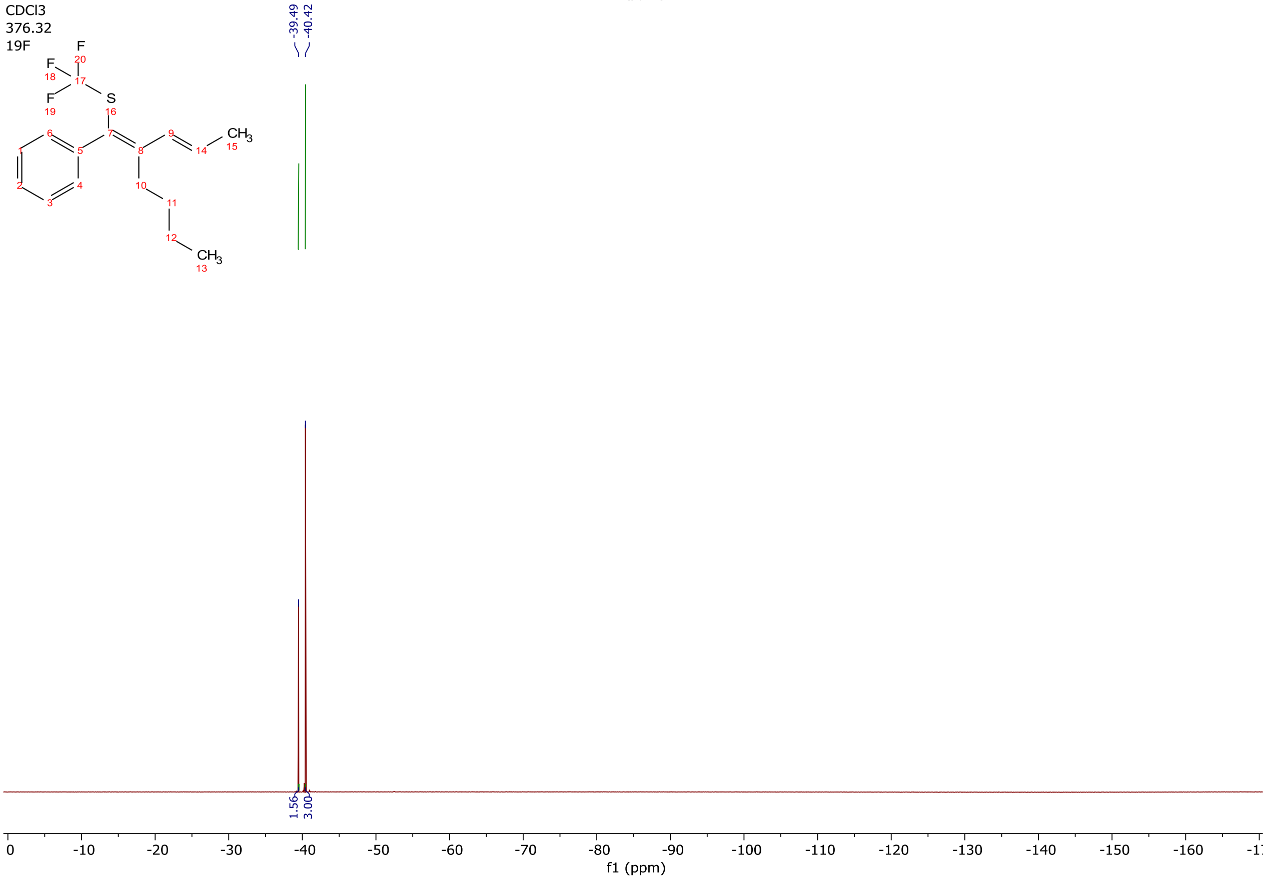

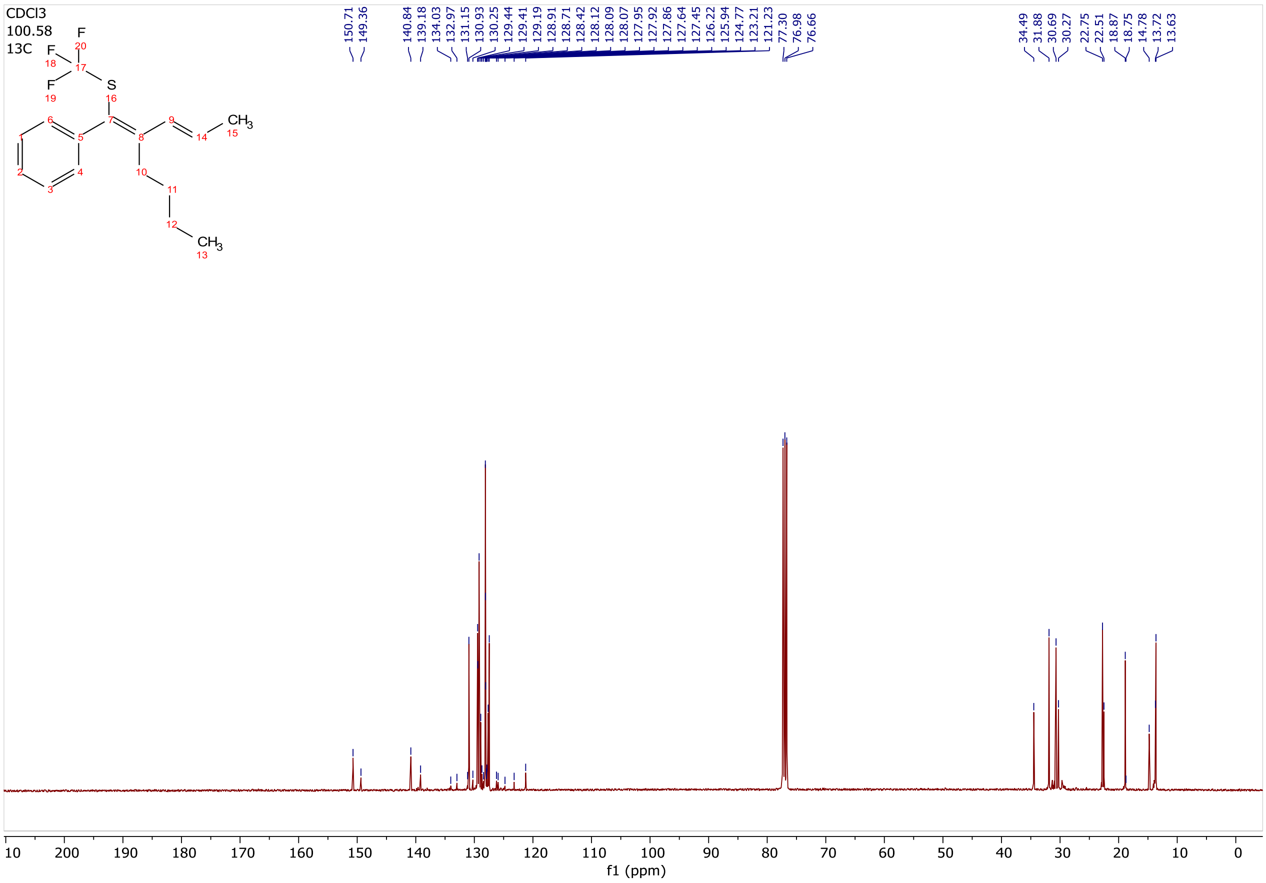
CDCl3
100.58
13C
F
F
F
S
CH3
CH3
150.71
149.36
140.84
139.18
134.03
132.97
131.15
130.93
130.25
129.44
129.41
129.19
128.91
128.71
128.42
128.12
128.09
128.07
127.95
127.92
127.86
127.64
127.45
126.22
125.94
124.77
123.21
121.23
77.30
76.98
76.66
34.49
31.88
30.69
30.27
22.75
22.51
18.87
18.75
14.78
13.72
13.63
f1 (ppm)

(Z)-(1-((Trifluoromethyl)sulfinyl)hex-1-ene-1,2-diyl)dibenzene (9) (400 MHz [^{1}H];101 MHz [^{13}C], 376 MHz [^{19}F] $CDCl_3$)

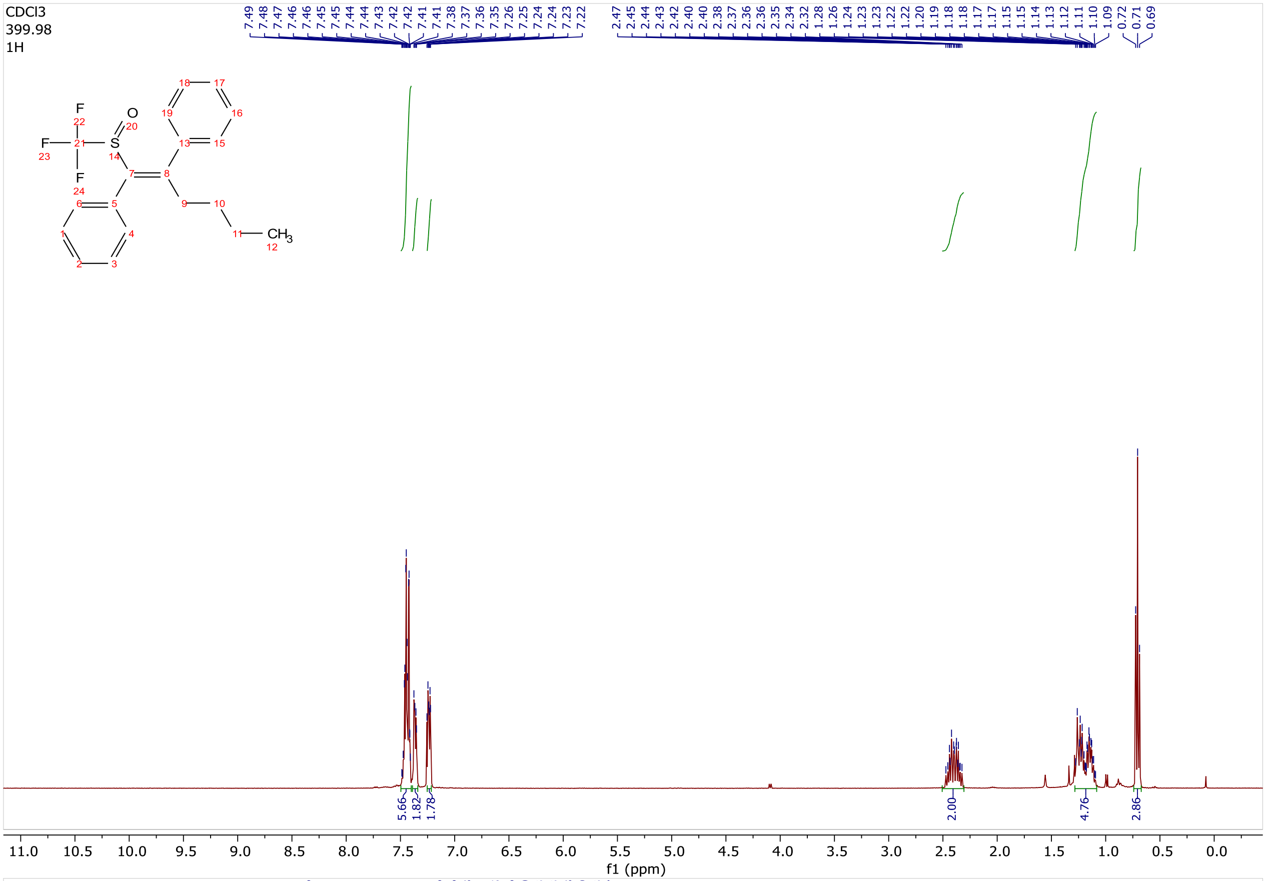

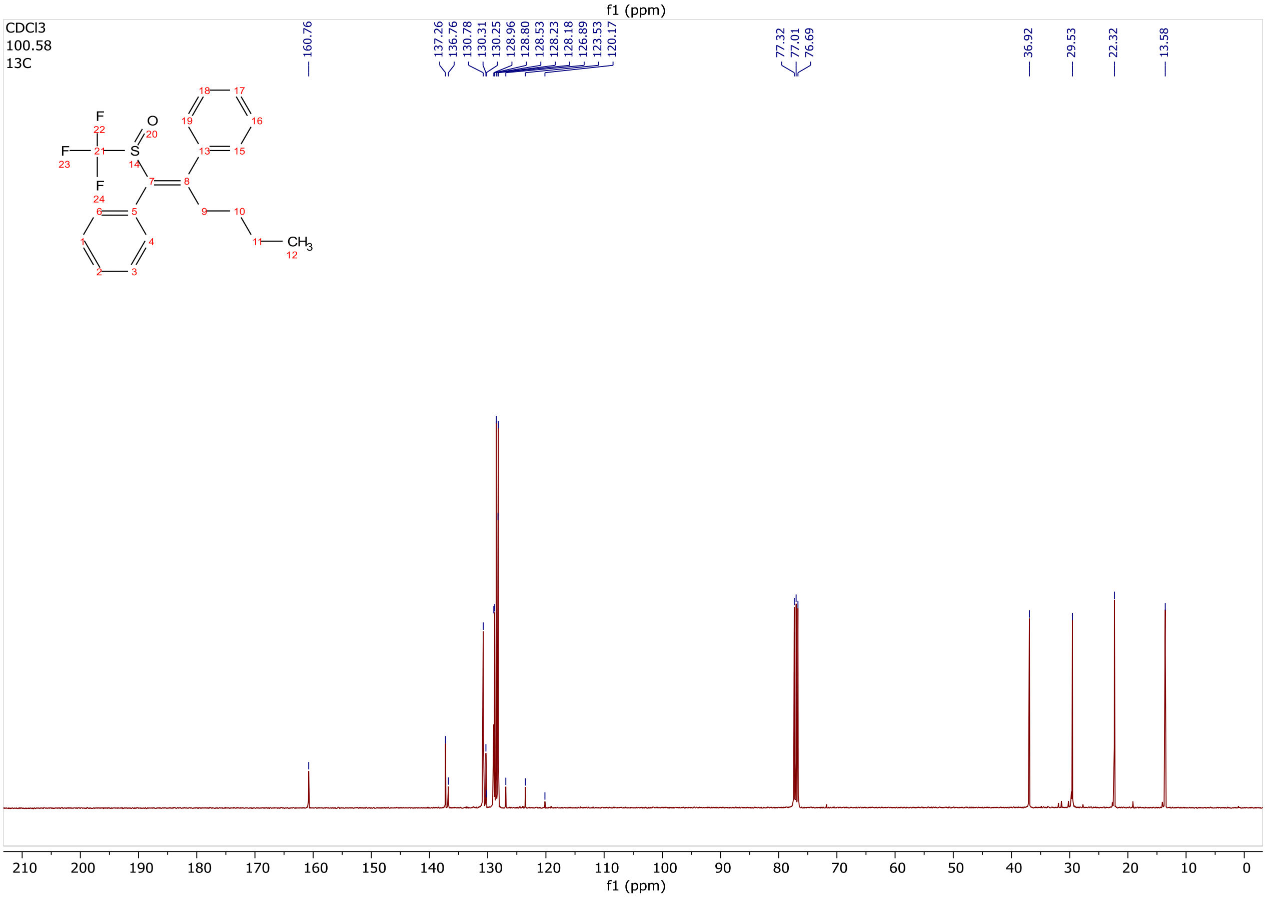

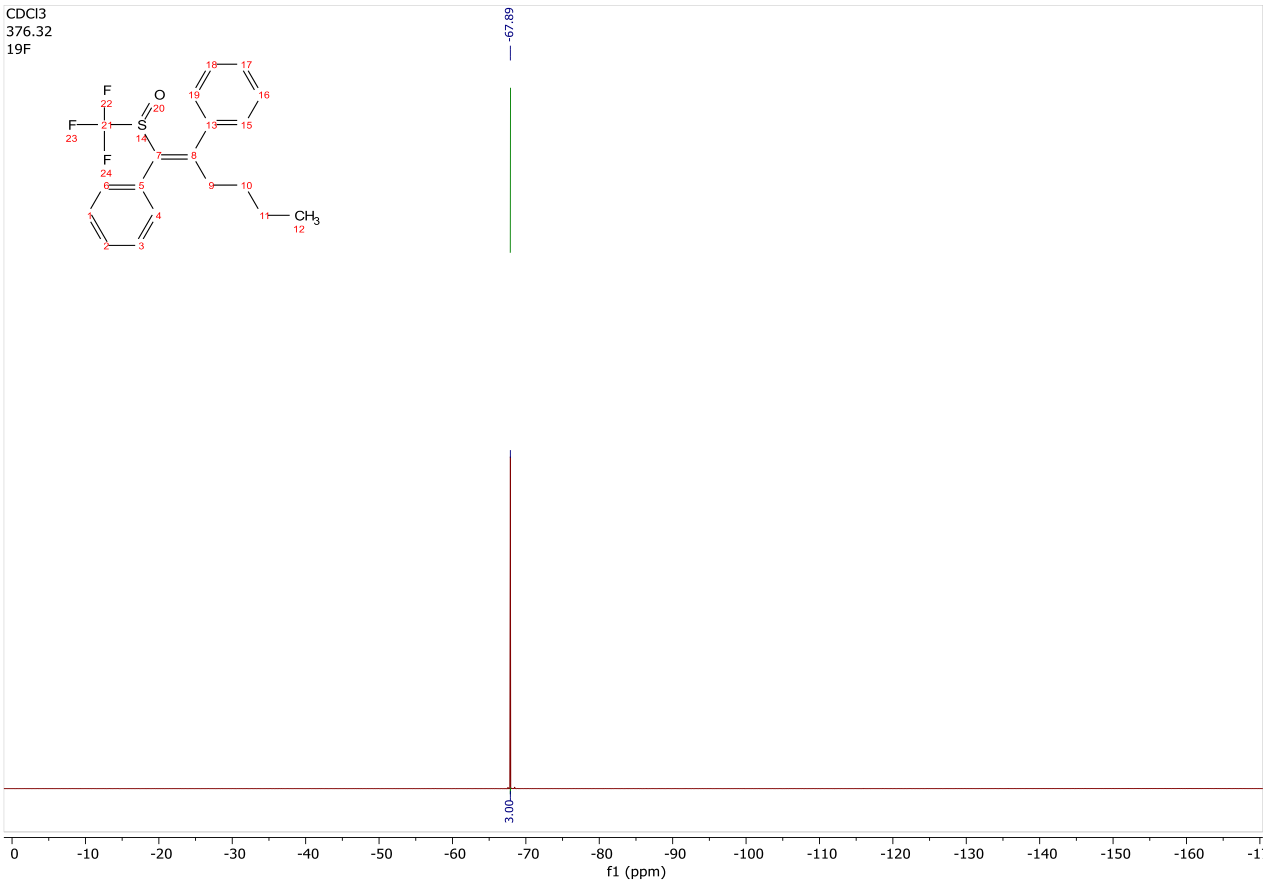
CDCl3
376.32
19F
-67.89
3.00
0
-10
-20
-30
-40
-50
-60
-70
-80
-90
-100
-110
-120
-130
-140
-150
-160
f1 (ppm)

(Z)-(1-((Trifluoromethyl)sulfonyl)hex-1-ene-1,2-diyl)dibenzene (10) (400 MHz [^{1}H];101 MHz [^{13}C], 376 MHz [^{19}F] $CDCl_3$)

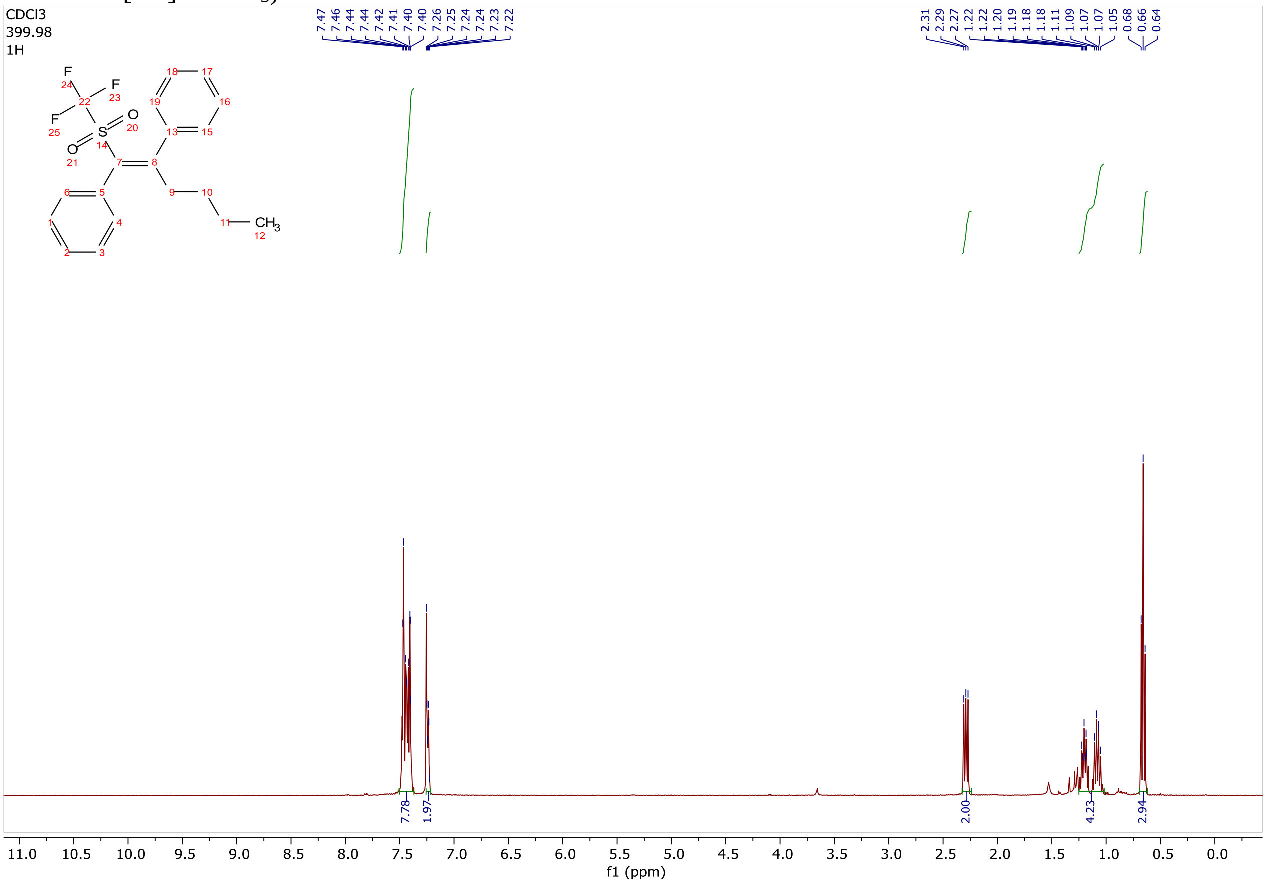

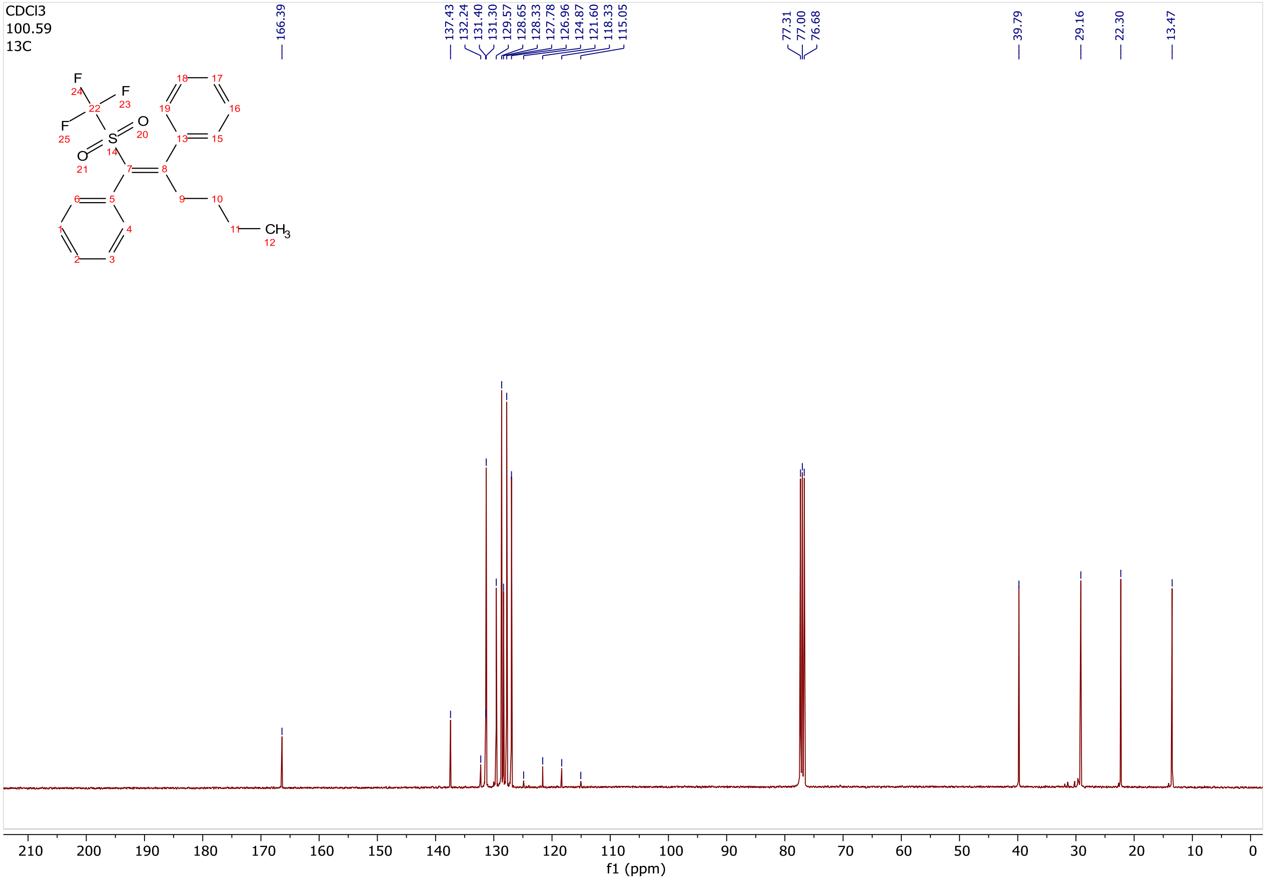

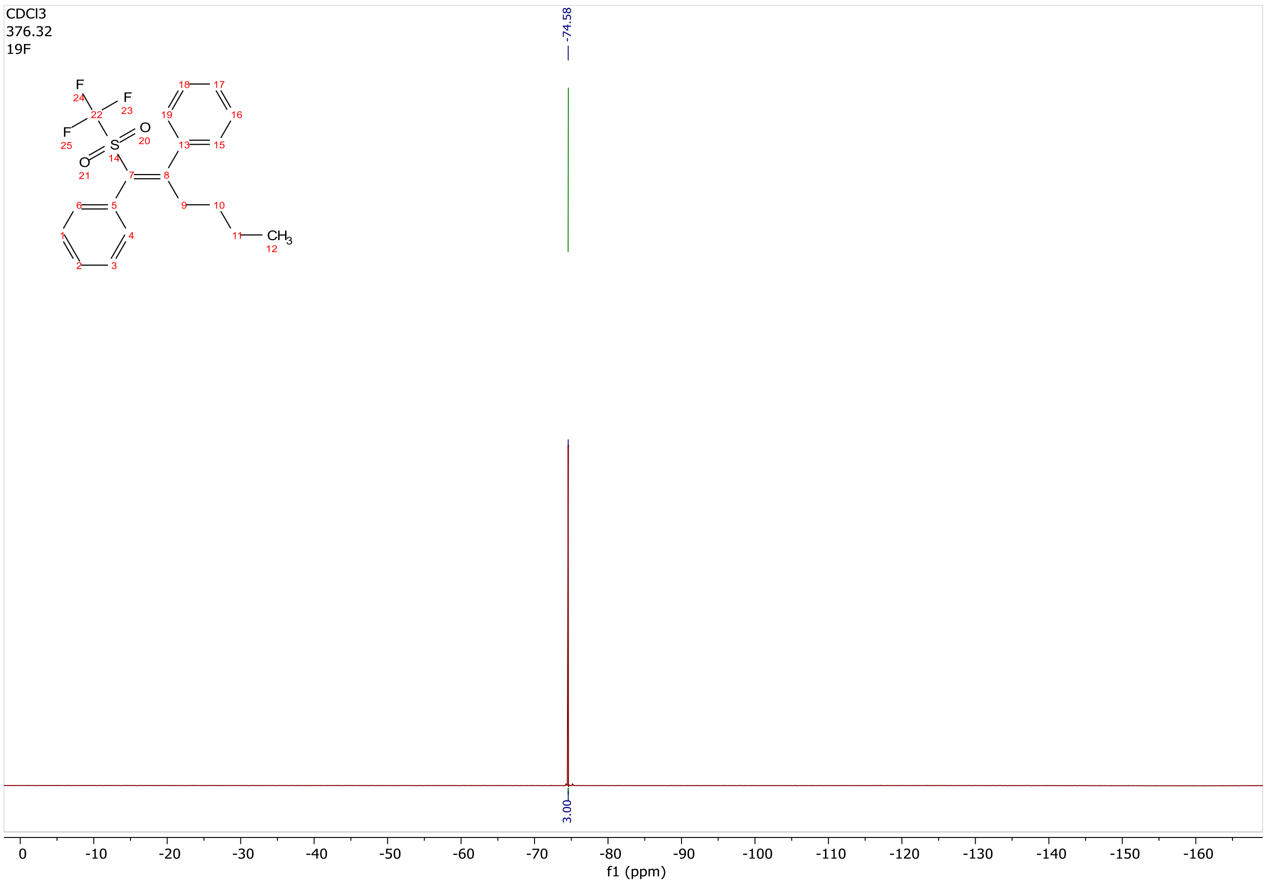
CDCl3
376.32
19F
-74.58
3.00
0
-10
-20
-30
-40
-50
-60
-70
-80
-90
-100
-110
-120
-130
-140
-150
-160
f1 (ppm)

(Z)-N-(4,5-Diphenyl-5-((trifluoromethyl)thio)pent-4-en-1-yl)-4-methyl-N-(oct-7-yn-1-yl)benzenesulfonamide (11) (400 MHz [^{1}H];101 MHz [^{13}C], 376 MHz [^{19}F] $CDCl_3$)

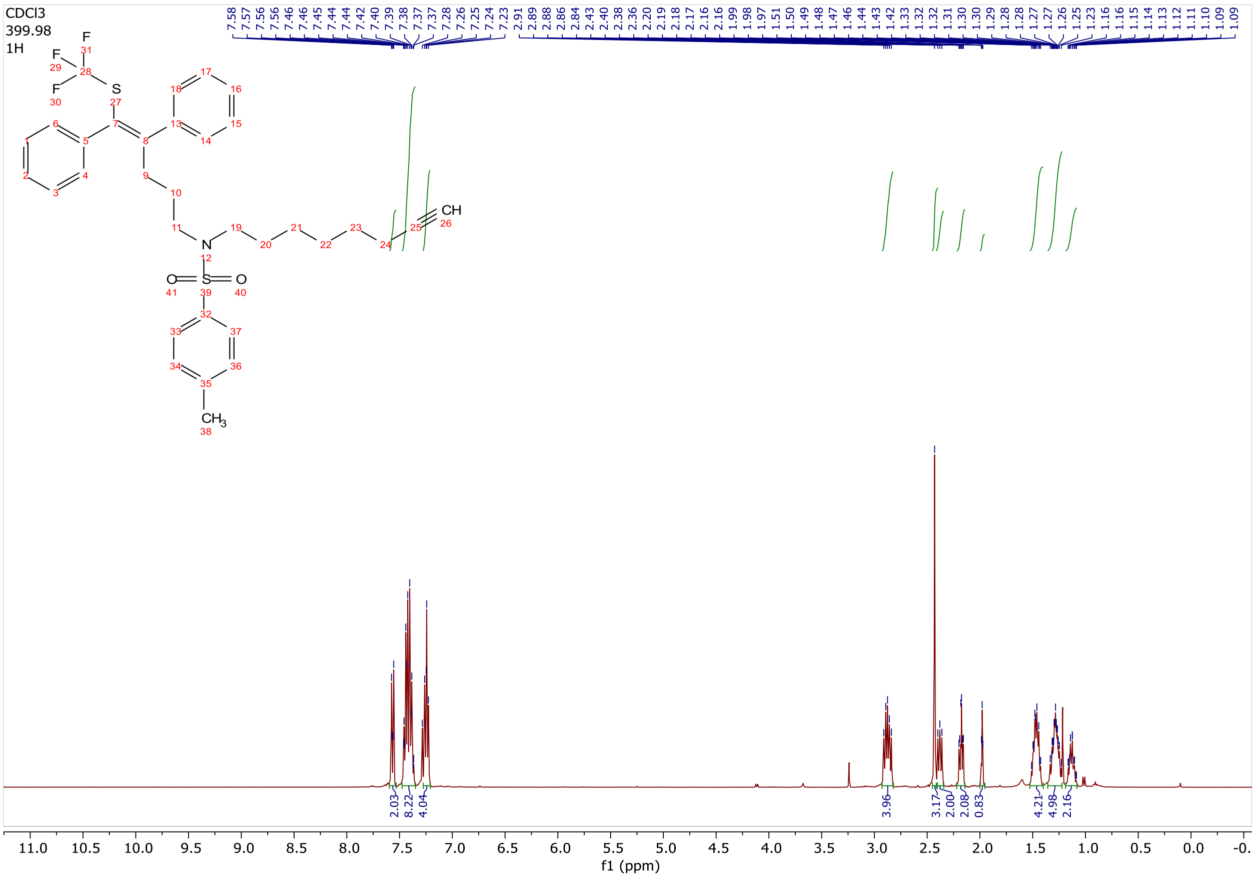

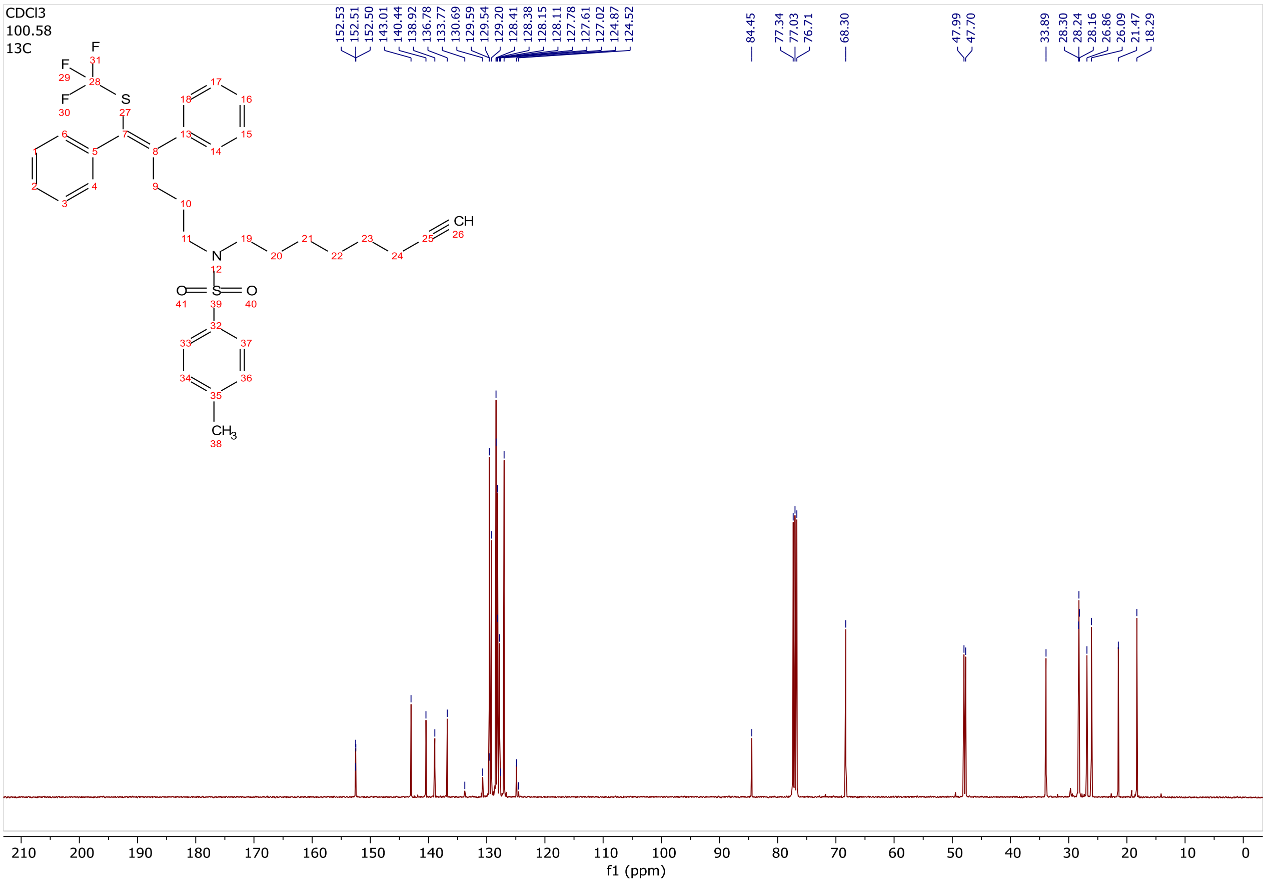

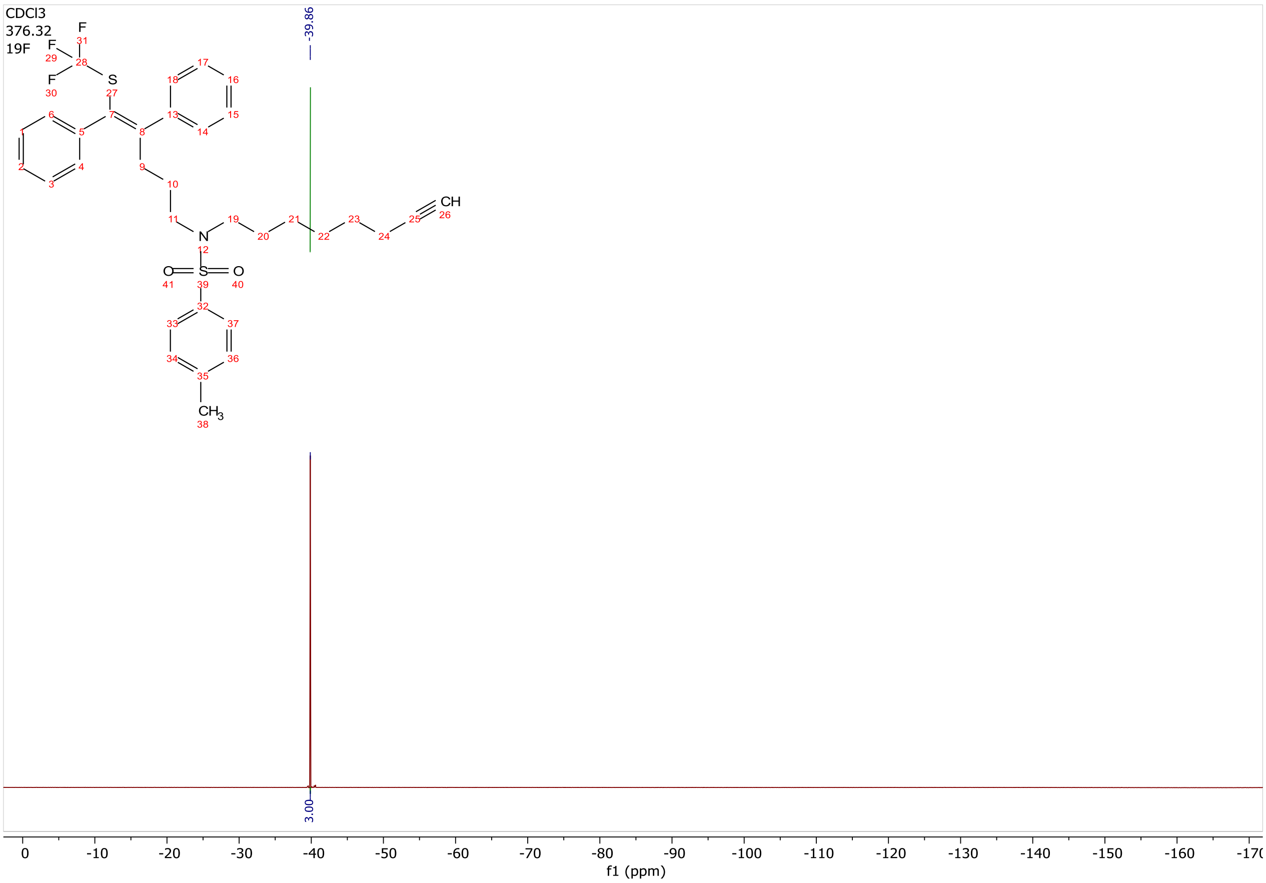
CDCl3
376.32
19F
-39.86
3.00
f1 (ppm)

(Z)-1-(4'-(2-Phenyl-1-((trifluoromethyl)thio)hex-1-en-1-yl)-[1,1'-biphenyl]-4-yl)ethan-1-one(12) (400 MHz [^{1}H];101 MHz [^{13}C], 376 MHz [^{19}F] $CDCl_3$)

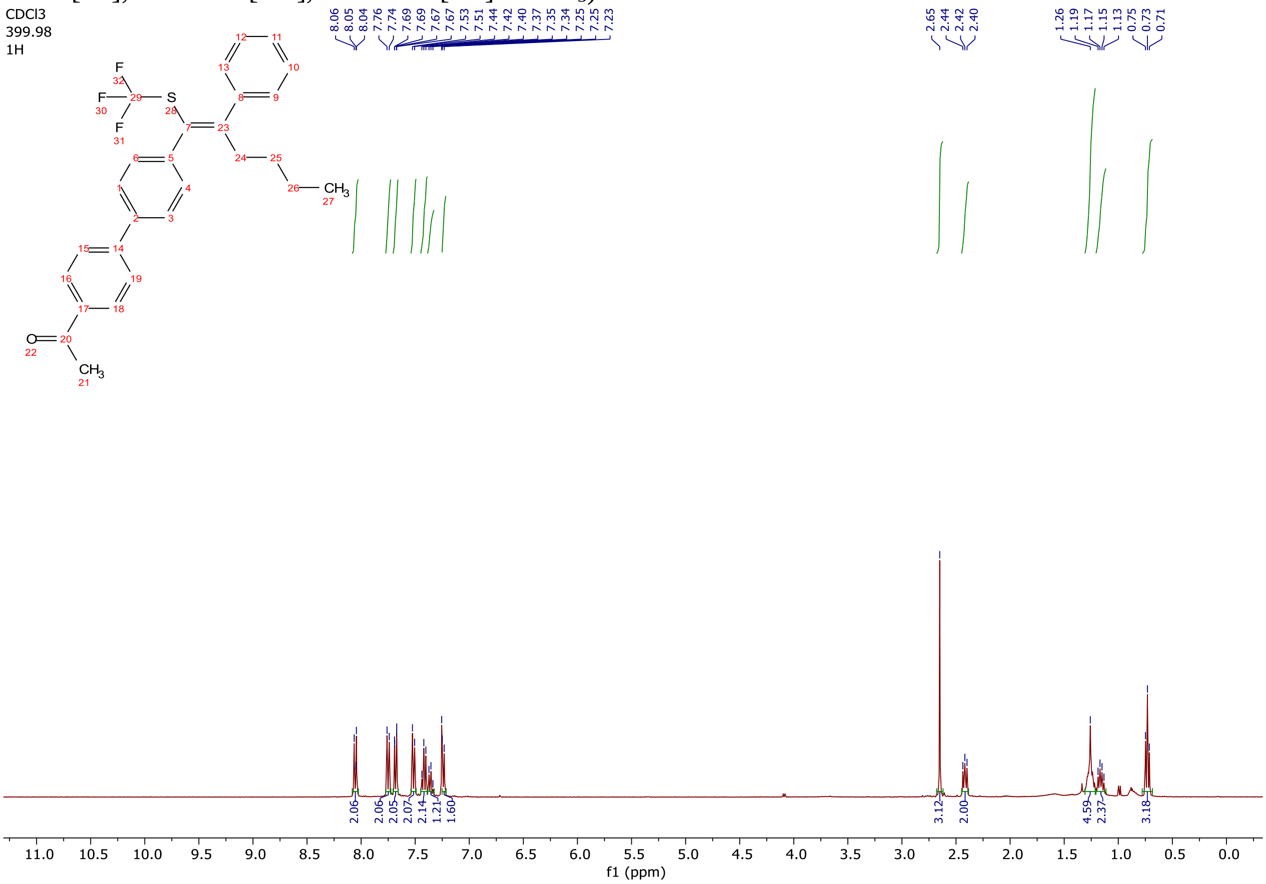

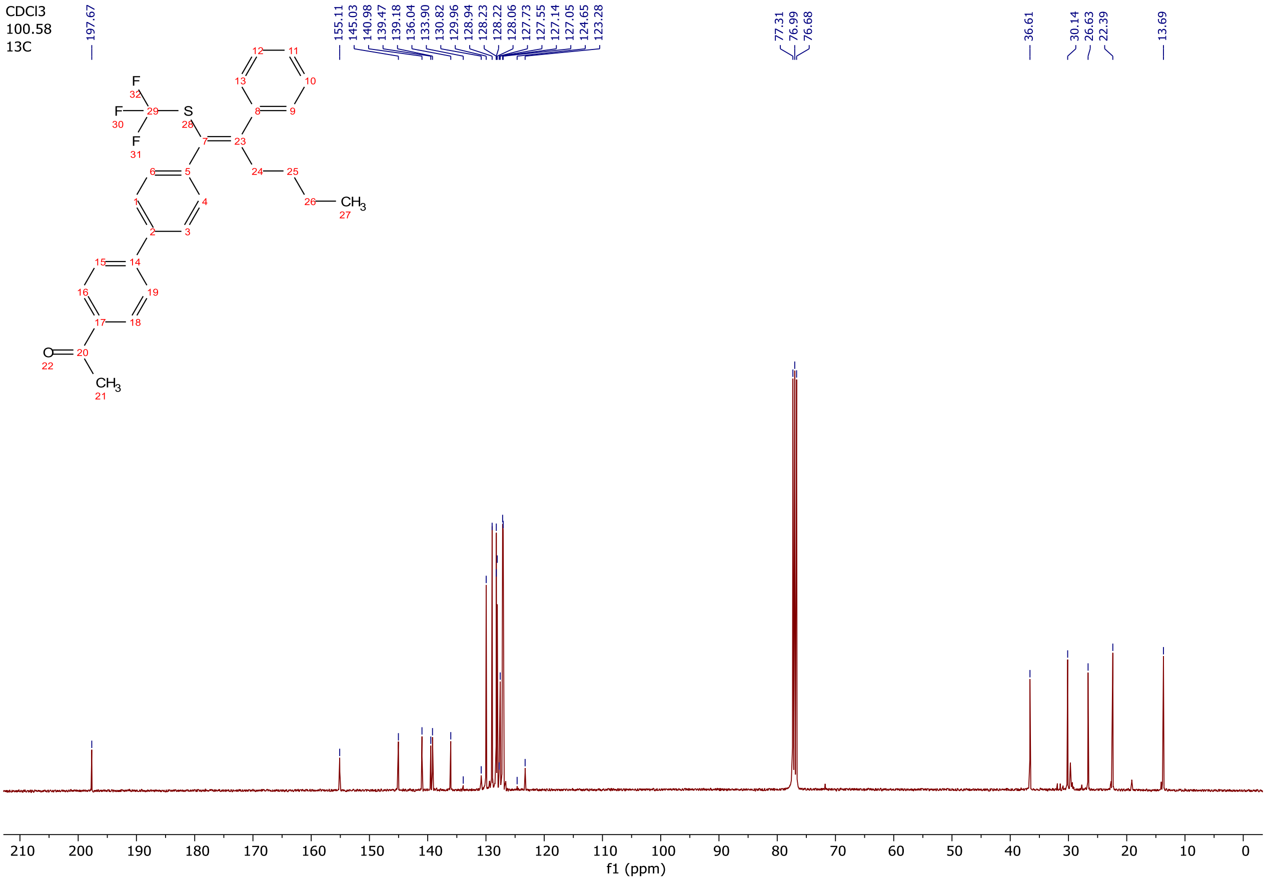

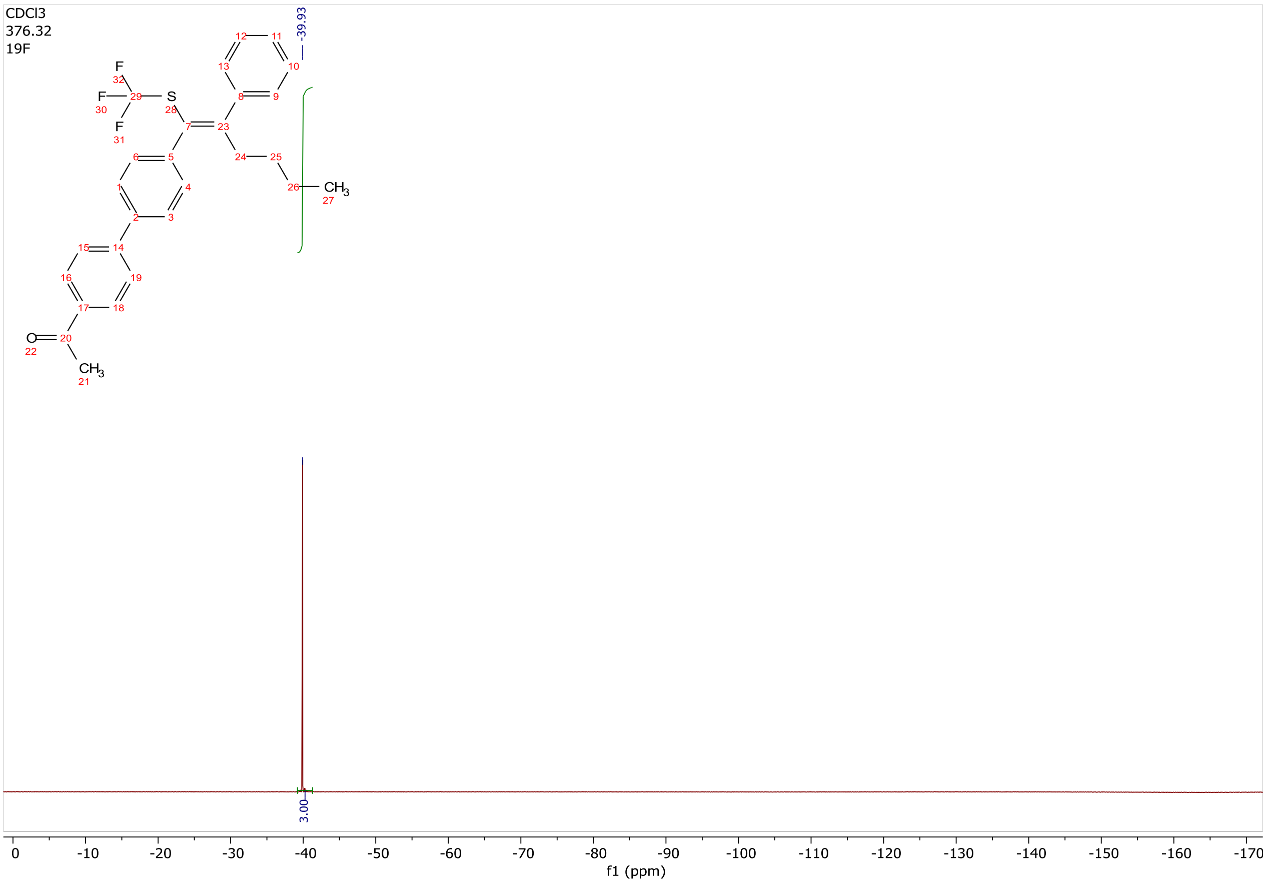
CDCl3
376.32
19F
-39.93
3.00
0
-10
-20
-30
-40
-50
-60
-70
-80
-90
-100
-110
-120
-130
-140
-150
-160
-170
f1 (ppm)

X-Ray Crystallographic Data for compound 10

Crystal Data and Experimental[22]

***R_1*=9.41%**

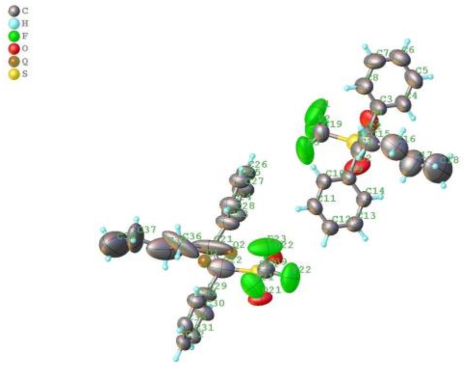

thermal ellipsoids shown at the 50% probability

Experimental. Single colourless needle-shaped crystals of **10** were obtained by recrystallisation from diethyl ether. A suitable crystal 0.41×0.16×0.04 mm^3 was selected and loop on an XtaLAB Synergy, Dualflex, HyPix-Arc 150 diffractometer. The crystal was kept at a steady T = 298 K during data collection. The structure was solved with the ShelXT (Sheldrick, 2015) structure solution program using the Intrinsic Phasing solution methods solution method and by using **Olex2** (Dolomanov et al., 2009) as the graphical interface. The model was refined with version 2018/3 of ShelXL 2018/3 (Sheldrick, 2015) using Least Squares minimisation.

Crystal Data. $C_{19}H_{19}F_3O_2S$, M_r = 368.40, tetragonal, $I4_1/a$ (No. 88), a = 50.5323(14) Å, b = 50.5323(14) Å, c = 5.9145(2) Å, $\alpha = \beta = \gamma = 90°$, V = 15102.8(10) Å^3, T = 298 K, Z = 32, Z' = 2, μ(Cu K$_\alpha$) = 1.863, 44861 reflections measured, 7369 unique (R_{int} = 0.0838) which were used in all calculations. The final wR_2 was 0.2999 (all data) and R_1 was 0.0941 (I > 2(I)).

**Compound**	**10**
Formula	$C_{19}H_{19}F_3O_2S$
$D_{calc.}$/ g cm^{-3}	1.296
μ/mm^{-1}	1.863
Formula Weight	368.40
Colour	colourless
Shape	needle
Size/mm^3	0.41×0.16×0.04
T/K	298
Crystal System	tetragonal
Space Group	$I4_1/a$
a/Å	50.5323(14)
b/Å	50.5323(14)
c/Å	5.9145(2)
α/°	90
β/°	90
γ/°	90
V/Å^3	15102.8(10)
Z	32
Z'	2
Wavelength/Å	1.54184
Radiation type	Cu K$_\alpha$
Θ_{min}/°	2.473
Θ_{max}/°	76.725
Measured Refl.	44861
Independent Refl.	7369
Reflections with I > 2(I)	3552
R_{int}	0.0838
Parameters	454
Restraints	9
Largest Peak	0.680
Deepest Hole	-0.613
GooF	1.059
wR_2 (all data)	0.2999
wR_2	0.2486
R_1 (all data)	0.1657
R_1	0.0941

Structure Quality Indicators

**Reflections:**	d min (CuKα) 2Θ=153.4° 0.79	I/σ(I) 12.3	Rint m=6.38 8.38%	Full 135.4° 92% to 153.4° 99.4
**Refinement:**	Shift 1.819	Max Peak 0.7	Min Peak -0.6	GooF 1.059

Experimental Extended. A colourless needle-shaped crystal with dimensions 0.41×0.16×0.04 mm^3 was loop. Data were collected using an XtaLAB Synergy, Dualflex, HyPix-Arc 150 diffractometer operating at T = 298 K.

Data were measured using ω scans of 0.5° per frame for 0.3 s using Cu K_α radiation. The diffraction pattern was indexed and the total number of runs and images was based on the strategy calculation from the program CrysAlisPro (Rigaku, V1.171.44.85a, 2024) The maximum resolution that was achieved was Θ = 76.725° (0.79 Å).

The diffraction pattern was indexed and the unit cell was refined on 3552 reflections, 92.3% of the observed reflections.

Data reduction, scaling and absorption corrections were performed using CrysAlisPro (Rigaku, V1.171.44.85a, 2024). The final completeness is 99.40 % out to 76.725° in Θ. A gaussian absorption correction was performed using CrysAlisPro 1.171.44.85a (Rigaku Oxford Diffraction, 2024)Numerical absorption correction based on gaussian integration overa multifaceted crystal modelEmpirical absorption correction using spherical harmonics,implemented in SCALE3 ABSPACK scaling algorithm. The absorption coefficient μ of this material is 1.863 mm^{-1} at this wavelength (λ = 1.542Å) and the minimum and maximum transmissions are 0 and 0.

The structure was solved and the space group $I4_1/a$ (# 88) determined by the the ShelXT (Sheldrick, 2015) structure solution program using Intrinsic Phasing methods and refined by Least Squares using version 2018/3 of ShelXL 2018/3 (Sheldrick, 2015). All non-hydrogen atoms were refined anisotropically. Hydrogen atom positions were calculated geometrically and refined using the riding model. Hydrogen atom positions were calculated geometrically and refined using the riding model.

Table 1: Fractional Atomic Coordinates (×10^4) and Equivalent Isotropic Displacement Parameters (Å^2×10^3) for **10**. U_{eq} is defined as 1/3 of the trace of the orthogonalised U_{ij}.

Atom	x	y	z	U_{eq}
S1	3403.0(3)	2291.0(3)	7920(2)	79.8(5)
S21	2791.7(2)	3827.1(2)	3211(2)	63.2(4)
F1	3173.5(11)	2088.2(13)	4433(9)	180(2)
F2	2938.0(9)	2098.5(11)	7313(9)	156.4(19)
F3	3006.4(11)	2439.9(11)	5412(13)	199(3)
F21	3030.3(8)	4126.6(9)	239(8)	140.9(17)
F22	3272.5(8)	3968.4(12)	2688(9)	166(2)
F23	3141.2(11)	3730.4(10)	96(10)	186(3)
O1	3490.9(11)	2039.4(9)	8576(9)	136.2(19)
O2	3319.0(12)	2471.5(11)	9572(9)	144(2)
O21	2747.9(9)	4050.3(8)	4554(7)	108.1(15)
O22	2867.1(9)	3587.9(8)	4209(9)	120.9(17)
C1	3625.2(16)	2481.5(15)	6070(12)	109(2)
C2	3809.4(16)	2380.8(16)	5089(13)	116(3)
C3	3892.1(14)	2086.2(13)	5227(11)	105(2)
C4	4046.8(14)	1991.7(13)	6940(12)	108(2)
C5	4153.3(15)	1740.5(14)	6825(13)	116(2)
C6	4110.6(17)	1585.6(15)	5001(14)	118(2)

Atom	x	y	z	U_{eq}
C7	3950.8(17)	1673.5(14)	3306(13)	121(3)
C8	3842.3(16)	1926.3(14)	3408(11)	118(3)
C9	3556.1(13)	2778.9(11)	5948(10)	87.5(17)
C10	3410.3(12)	2877.8(11)	4170(9)	83.9(16)
C11	3334.2(11)	3139.7(11)	4179(10)	83.7(16)
C12	3404.0(12)	3301.0(11)	5922(10)	84.1(16)
C13	3552.0(12)	3206.3(12)	7665(11)	87.1(17)
C14	3628.3(12)	2945.6(12)	7683(10)	90.8(18)
C15	3992.0(17)	2551.4(14)	3465(12)	109(2)
C16	4262(3)	2606(2)	4160(20)	181(4)
C17	4314(2)	2698(3)	6240(20)	187(5)
C18	4608(2)	2765(2)	6770(30)	251(7)
C19	3114.6(15)	2226.6(16)	6170(14)	101(2)
C21	2410(3)	3627.0(14)	621(18)	262(9)
C22	2498.3(17)	3831.9(15)	1298(15)	137(3)
C23	2550.3(17)	3362.8(12)	844(12)	113(2)
C24	2712.9(15)	3265.6(12)	-806(11)	104(2)
C25	2787.0(14)	3005.9(13)	-774(12)	100(2)
C26	2703.0(15)	2840.5(12)	895(14)	103(2)
C27	2541.5(16)	2932.4(12)	2528(13)	110(2)
C28	2464.4(16)	3191.7(14)	2498(12)	118(3)
C29	2373.2(13)	4112.7(10)	995(12)	92.5(18)
C30	2219.5(12)	4210.0(12)	2668(12)	97(2)
C31	2123.9(11)	4463.7(11)	2594(10)	79.9(15)
C32	2180.6(10)	4621.5(11)	822(10)	76.3(15)
C33	2335.2(11)	4528.6(11)	-891(10)	81.4(16)
C34	2429.0(12)	4271.7(11)	-858(10)	82.9(16)
C35	1986(3)	3665(3)	-1150(30)	274(11)
C36	2224(2)	3672.9(19)	-1747(19)	237(8)
C37	1784(2)	3620(3)	-3370(30)	214(7)
C38	1556(4)	3672(3)	-3620(50)	393(17)
C39	3071.4(12)	3915.4(13)	1379(12)	86.3(16)

Table 2: Anisotropic Displacement Parameters (×10[4]) **10**. The anisotropic displacement factor exponent takes the form: $-2\pi^2[h^2a^{*2} \times U_{11} + ... + 2hka^* \times b^* \times U_{12}]$

Atom	U_{11}	U_{22}	U_{33}	U_{23}	U_{13}	U_{12}
S1	95.1(11)	78.8(10)	65.3(8)	2.5(7)	-3.8(7)	-3.4(8)
S21	66.7(8)	58.4(7)	64.3(7)	0.4(6)	-4.8(6)	5.5(6)
F1	148(4)	264(7)	127(4)	-71(4)	-23(3)	-42(4)
F2	116(3)	187(5)	166(4)	26(4)	2(3)	-57(3)
F3	144(4)	149(4)	302(8)	78(5)	-104(5)	-17(3)
F21	119(3)	145(4)	158(4)	68(3)	46(3)	7(3)
F22	80(3)	242(6)	175(4)	24(4)	-11(3)	-40(3)
F23	177(5)	136(4)	245(6)	-79(4)	136(4)	-32(3)
O1	156(5)	94(3)	158(4)	41(3)	-50(4)	9(3)
O2	178(5)	134(4)	120(4)	-58(3)	31(4)	-11(4)
O21	136(4)	78(3)	110(3)	-36(2)	41(3)	-10(2)
O22	110(3)	91(3)	162(4)	60(3)	2(3)	31(2)
C1	118(6)	117(6)	93(4)	-24(4)	-45(4)	33(5)
C2	117(6)	128(6)	103(5)	-34(5)	-40(5)	44(5)
C3	134(6)	91(4)	90(4)	-17(4)	-31(4)	46(4)
C4	128(6)	96(5)	98(4)	-14(4)	-39(4)	43(4)
C5	136(6)	106(5)	106(5)	14(5)	-19(5)	48(5)
C6	154(7)	88(5)	113(5)	-2(4)	-1(5)	42(5)
C7	173(8)	89(5)	101(5)	-26(4)	-9(5)	24(5)
C8	172(7)	94(5)	87(4)	-14(4)	-40(5)	37(5)
C9	112(5)	65(3)	86(4)	-5(3)	-29(3)	20(3)
C10	104(4)	74(4)	73(3)	-2(3)	-25(3)	14(3)
C11	90(4)	77(4)	84(4)	11(3)	-12(3)	22(3)
C12	87(4)	71(4)	94(4)	-5(3)	-8(3)	9(3)

Atom	U_{11}	U_{22}	U_{33}	U_{23}	U_{13}	U_{12}
C13	92(4)	76(4)	94(4)	-15(3)	-11(3)	3(3)
C14	109(5)	80(4)	84(4)	-8(3)	-34(3)	13(3)
C15	129(6)	91(5)	109(5)	20(4)	29(5)	21(4)
C16	159(11)	181(11)	203(13)	31(10)	47(10)	-1(8)
C17	170(11)	229(13)	162(10)	-26(10)	-5(9)	-13(9)
C18	136(9)	213(13)	400(20)	8(14)	12(12)	-54(9)
C19	99(5)	99(5)	107(5)	13(4)	-20(4)	-1(4)
C21	550(30)	54(5)	179(11)	22(6)	129(14)	53(9)
C22	161(8)	101(6)	148(7)	8(5)	70(6)	-7(5)
C23	183(8)	61(4)	96(5)	10(4)	18(5)	25(4)
C24	150(6)	71(4)	91(4)	12(3)	8(4)	7(4)
C25	122(6)	70(4)	108(5)	-9(4)	7(4)	13(4)
C26	120(6)	55(3)	134(6)	-7(4)	-18(5)	7(3)
C27	157(7)	59(4)	115(5)	7(4)	1(5)	-3(4)
C28	169(7)	82(5)	104(5)	-4(4)	35(5)	24(5)
C29	105(5)	51(3)	121(5)	2(3)	28(4)	9(3)
C30	94(4)	78(4)	117(5)	31(4)	32(4)	24(3)
C31	73(4)	80(4)	87(4)	-1(3)	5(3)	23(3)
C32	63(3)	66(3)	100(4)	0(3)	-11(3)	16(3)
C33	84(4)	75(4)	85(4)	18(3)	-10(3)	5(3)
C34	94(4)	70(4)	85(4)	-5(3)	6(3)	14(3)
C35	163(11)	174(11)	480(30)	-102(15)	144(17)	-52(9)
C36	293(16)	153(9)	264(14)	131(10)	162(13)	135(10)
C37	130(9)	190(11)	321(17)	38(11)	-121(11)	-46(8)
C38	243(19)	247(19)	690(50)	-170(20)	-130(30)	28(15)
C39	75(4)	81(4)	103(4)	-3(4)	7(4)	-3(3)

Table 3: Bond Lengths in Å for **10**.

Atom	Atom	Length/Å
S1	O1	1.401(4)
S1	O2	1.403(5)
S1	C1	1.839(9)
S1	C19	1.817(7)
S21	O21	1.397(4)
S21	O22	1.398(4)
S21	C22	1.865(10)
S21	C39	1.836(6)
F1	C19	1.278(8)
F2	C19	1.293(8)
F3	C19	1.289(8)
F21	C39	1.279(7)
F22	C39	1.305(7)
F23	C39	1.255(7)
C1	C2	1.209(9)
C1	C9	1.544(9)
C2	C3	1.548(9)
C2	C15	1.586(11)
C3	C4	1.366(8)
C3	C8	1.369(8)
C4	C5	1.380(8)
C5	C6	1.350(10)
C6	C7	1.361(10)
C7	C8	1.392(9)
C9	C10	1.378(7)
C9	C14	1.376(8)

Atom	Atom	Length/Å
C10	C11	1.378(7)
C11	C12	1.360(7)
C12	C13	1.360(8)
C13	C14	1.373(8)
C15	C16	1.452(13)
C16	C17	1.338(13)
C17	C18	1.553(14)
C21	C22	1.196(9)
C21	C23	1.517(9)
C21	C36	1.702(13)
C22	C29	1.563(9)
C23	C24	1.367(9)
C23	C28	1.376(9)
C24	C25	1.365(8)
C25	C26	1.362(9)
C26	C27	1.347(9)
C27	C28	1.367(9)
C29	C30	1.351(8)
C29	C34	1.388(8)
C30	C31	1.371(7)
C31	C32	1.348(7)
C32	C33	1.363(7)
C33	C34	1.383(7)
C35	C36	1.257(13)
C35	C37	1.68(2)
C37	C38	1.194(16)

Table 4: Bond Angles in ° for **10.**

Atom	Atom	Atom	Angle/°
O1	S1	O2	119.5(4)
O1	S1	C1	116.5(3)
O1	S1	C19	104.4(3)
O2	S1	C1	105.0(3)
O2	S1	C19	105.7(4)
C19	S1	C1	104.1(3)
O21	S21	O22	120.1(3)
O21	S21	C22	102.0(3)
O21	S21	C39	105.1(3)
O22	S21	C22	118.9(3)
O22	S21	C39	104.5(3)
C39	S21	C22	104.5(3)
C2	C1	S1	122.3(7)
C2	C1	C9	124.1(8)
C9	C1	S1	113.5(5)
C1	C2	C3	125.9(9)
C1	C2	C15	120.6(8)
C3	C2	C15	113.4(6)
C4	C3	C2	122.0(6)
C4	C3	C8	118.8(6)
C8	C3	C2	118.4(6)
C3	C4	C5	120.6(6)
C6	C5	C4	120.7(6)
C5	C6	C7	119.6(6)
C6	C7	C8	120.1(7)
C3	C8	C7	120.2(6)
C10	C9	C1	120.6(5)
C14	C9	C1	120.1(5)
C14	C9	C10	119.2(5)
C9	C10	C11	119.6(5)
C12	C11	C10	120.4(5)
C11	C12	C13	120.4(5)
C12	C13	C14	119.8(6)
C13	C14	C9	120.5(5)
C16	C15	C2	118.5(7)
C17	C16	C15	120.8(11)
C16	C17	C18	116.7(13)

Atom	Atom	Atom	Angle/°
F1	C19	S1	111.7(6)
F1	C19	F2	107.9(7)
F1	C19	F3	106.1(7)
F2	C19	S1	110.2(5)
F3	C19	S1	112.8(6)
F3	C19	F2	107.9(7)
C22	C21	C23	124.0(11)
C22	C21	C36	111.2(8)
C23	C21	C36	116.7(7)
C21	C22	S21	119.2(8)
C21	C22	C29	126.7(9)
C29	C22	S21	113.8(5)
C24	C23	C21	122.3(7)
C24	C23	C28	118.1(6)
C28	C23	C21	117.9(8)
C25	C24	C23	120.1(6)
C26	C25	C24	121.0(7)
C27	C26	C25	119.8(6)
C26	C27	C28	119.6(7)
C27	C28	C23	121.4(7)
C30	C29	C22	118.6(6)
C30	C29	C34	119.0(5)
C34	C29	C22	122.3(6)
C29	C30	C31	121.3(6)
C32	C31	C30	120.2(5)
C31	C32	C33	119.8(5)
C32	C33	C34	120.6(5)
C33	C34	C29	119.0(5)
C36	C35	C37	111.6(15)
C35	C36	C21	107.1(12)
C38	C37	C35	131(2)
F21	C39	S21	112.9(4)
F21	C39	F22	105.6(6)
F22	C39	S21	107.4(5)
F23	C39	S21	113.1(5)
F23	C39	F21	110.4(6)
F23	C39	F22	107.0(6)

Table 5: Torsion Angles in ° for **10**.

Atom	Atom	Atom	Atom	Angle/°
S1	C1	C2	C3	-1.0(10)
S1	C1	C2	C15	178.6(5)
S1	C1	C9	C10	-98.3(6)
S1	C1	C9	C14	78.3(7)
S21	C22	C29	C30	-77.4(7)
S21	C22	C29	C34	98.2(7)
O1	S1	C1	C2	14.7(7)
O1	S1	C1	C9	-164.1(4)
O1	S1	C19	F1	-60.8(7)
O1	S1	C19	F2	59.1(7)
O1	S1	C19	F3	179.8(7)
O2	S1	C1	C2	149.4(6)
O2	S1	C1	C9	-29.3(5)
O2	S1	C19	F1	172.3(6)
O2	S1	C19	F2	-67.8(6)
O2	S1	C19	F3	52.9(8)
O21	S21	C22	C21	-148.6(9)
O21	S21	C22	C29	24.4(5)

Atom	Atom	Atom	Atom	Angle/˚
O21	S21	C39	F21	-55.7(6)
O21	S21	C39	F22	60.2(5)
O21	S21	C39	F23	178.1(6)
O22	S21	C22	C21	-13.9(10)
O22	S21	C22	C29	159.1(5)
O22	S21	C39	F21	177.0(5)
O22	S21	C39	F22	-67.1(6)
O22	S21	C39	F23	50.8(7)
C1	S1	C19	F1	61.9(7)
C1	S1	C19	F2	-178.2(6)
C1	S1	C19	F3	-57.5(7)
C1	C2	C3	C4	-83.4(10)
C1	C2	C3	C8	106.7(9)
C1	C2	C15	C16	110.4(10)
C1	C9	C10	C11	175.2(6)
C1	C9	C14	C13	-175.5(6)
C2	C1	C9	C10	82.9(9)
C2	C1	C9	C14	-100.5(9)
C2	C3	C4	C5	-169.2(7)
C2	C3	C8	C7	169.5(8)
C2	C15	C16	C17	-49.1(15)
C3	C2	C15	C16	-70.0(9)
C3	C4	C5	C6	1.2(12)
C4	C3	C8	C7	-0.7(13)
C4	C5	C6	C7	-3.1(13)
C5	C6	C7	C8	3.0(13)
C6	C7	C8	C3	-1.1(13)
C8	C3	C4	C5	0.7(12)
C9	C1	C2	C3	177.7(5)
C9	C1	C2	C15	-2.8(11)
C9	C10	C11	C12	0.5(9)
C10	C9	C14	C13	1.2(10)
C10	C11	C12	C13	0.7(9)
C11	C12	C13	C14	-0.9(9)
C12	C13	C14	C9	0.0(10)
C14	C9	C10	C11	-1.4(10)
C15	C2	C3	C4	97.0(9)
C15	C2	C3	C8	-72.9(9)
C15	C16	C17	C18	-176.7(9)
C19	S1	C1	C2	-99.7(7)
C19	S1	C1	C9	81.5(5)
C21	C22	C29	C30	94.9(12)
C21	C22	C29	C34	-89.5(11)
C21	C23	C24	C25	-165.4(8)
C21	C23	C28	C27	166.7(8)
C22	S21	C39	F21	51.3(6)
C22	S21	C39	F22	167.3(5)
C22	S21	C39	F23	-74.9(6)
C22	C21	C23	C24	-92.2(13)
C22	C21	C23	C28	102.8(13)
C22	C21	C36	C35	-101.8(13)
C22	C29	C30	C31	174.1(6)
C22	C29	C34	C33	-172.8(6)
C23	C21	C22	S21	-12.1(15)
C23	C21	C22	C29	176.0(7)
C23	C21	C36	C35	108.1(12)
C23	C24	C25	C26	-0.5(11)
C24	C23	C28	C27	1.0(13)
C24	C25	C26	C27	0.9(11)
C25	C26	C27	C28	-0.3(11)
C26	C27	C28	C23	-0.7(12)
C28	C23	C24	C25	-0.4(12)

Atom	Atom	Atom	Atom	Angle/˚
C29	C30	C31	C32	0.5(10)
C30	C29	C34	C33	2.8(10)
C30	C31	C32	C33	-0.5(9)
C31	C32	C33	C34	1.7(9)
C32	C33	C34	C29	-2.8(9)
C34	C29	C30	C31	-1.7(10)
C36	C21	C22	S21	-159.6(6)
C36	C21	C22	C29	28.5(14)
C36	C21	C23	C24	53.7(13)
C36	C21	C23	C28	-111.3(10)
C36	C35	C37	C38	-157(2)
C37	C35	C36	C21	-163.5(9)
C39	S21	C22	C21	102.1(9)
C39	S21	C22	C29	-84.9(5)

Table 6: Hydrogen Fractional Atomic Coordinates (×10⁴) and Equivalent Isotropic Displacement Parameters (Å²×10³) for **10**. U_{eq} is defined as 1/3 of the trace of the orthogonalised U_{ij}.

Atom	x	y	z	U_{eq}
H4	4080.71	2097.44	8193.52	129
H5	4255.47	1677.34	8016.05	139
H6	4189.89	1419.9	4902.25	142
H7	3914.07	1564.38	2079.42	145
H8	3735.7	1986.9	2235.42	141
H10	3363.43	2768.44	2970.67	101
H11	3234.52	3206.63	2986.99	100
H12	3350.23	3476.96	5921.57	101
H13	3601.32	3318	8841.28	105
H14	3729.53	2881.15	8874.89	109
H15A	3999.34	2461.93	2015.66	131
H15B	3904.67	2719.93	3218.35	131
H16A	4361.3	2443.01	3983.35	217
H16B	4334.6	2731.83	3092.68	217
H17A	4254.18	2568.22	7327.23	225
H17B	4210.25	2857.29	6467.57	225
H18A	4615.6	2912.58	7784.5	376
H18B	4698.62	2808.14	5391.55	376
H18C	4691.11	2614.1	7458.64	376
H24	2773.15	3376.49	-1950.07	124
H25	2896.55	2941.01	-1910.03	120
H26	2756.88	2664.49	907.73	123
H27	2482.8	2820.22	3669.5	132
H28	2351.34	3253.84	3619.89	142
H30	2177.65	4102.68	3894.48	116
H31	2019.56	4527.18	3770.96	96
H32	2114.41	4793.09	768.71	92
H33	2377.93	4639.2	-2093.86	98
H34	2528.07	4206.3	-2059.84	99
H35A	1940.04	3829.91	-402.72	329
H35B	1960.73	3522.41	-79.98	329
H36A	2265.17	3842.62	-2431.12	284
H36B	2260.83	3534.73	-2842.25	284
H37A	1872.73	3707.81	-4615.91	257
H37B	1795.73	3432.16	-3692.28	257
H38A	1478.99	3553.48	-4701.71	589
H38B	1539.04	3851	-4153.54	589
H38C	1464.93	3654.9	-2199.7	589

References

(1) Abas, H.; Linsdall, S. M.; Mamboury, M.; Rzepa, H. S.; Spivey, A. C. Total Synthesis of (+)-Lophirone H and Its Pentamethyl Ether Utilizing an Oxonium–Prins Cyclization. *Org. Lett.* **2017**, *19*, 2486–2489.

(2) Mori, A.; Shimada, T.; Kondo, T.; Sekiguchi, A. A Highly Effective Pd/Cu-Catalyzed Coupling Reaction of Terminal Alkynes with Organic Halides Promoted by Tetrabutylammonium Fluoride or Hydroxide. *Synlett* **2001**, *2001*, 649–651.

(3) Choi, G.; Kim, H. E.; Hwang, S.; Jang, H.; Chung, W. Phosphorus(III)-Mediated, Tandem Deoxygenative Geminal Chlorofluorination of 1,2-Diketones. *Org. Lett.* **2020**, *22*), 4190–4195.

(4) Zeng, Q.; Cai, P.; Li, Z.; Qin, J.; Tang, B. Z. An Imidazole-Functionalized Polyacetylene: Convenient Synthesis and Selective Chemosensor for Metal Ions and Cyanide. *Chem. Commun.* **2008**, No. 9, 1094–1096. https://doi.org/10.1039/B717764J.

(5) Teske, J.; Plietker, B. Fe-Catalyzed Cycloisomerization of Aryl Allenyl Ketones: Access to 3-Arylidene-Indan-1-Ones. *Org. Lett.* **2018**, *20*, 2257–2260.

(6) Wang, B.; Ong, D. Y.; Li, Y.; Pang, J. H.; Watanabe, K.; Takita, R.; Chiba, S. Stereo-Controlled Anti-Hydromagnesiation of Aryl Alkynes by Magnesium Hydrides. *Chem. Sci.* **2020**, *11* (20), 5267–5272.

(7) Dixon, L. I.; Carroll, M. A.; Gregson, T. J.; Ellames, G. J.; Harrington, R. W.; Clegg, W. Synthesis and Reactivity of Aryl(Alkynyl)Iodonium Salts. *Eur. J. Org. Chem.* **2013**, *2013*, 2334–2345.

(8) Zhang, W.-W.; Zhang, X.-G.; Li, J.-H. Palladium-Catalyzed Decarboxylative Coupling of Alkynyl Carboxylic Acids with Benzyl Halides or Aryl Halides. *J. Org. Chem.* **2010**, *75* (15), 5259–5264. https://doi.org/10.1021/jo1010284.

(9) Ngassa, F. N.; Lindsey, E. A.; Haines, B. E. The First Cu- and Amine-Free Sonogashira-Type Cross-Coupling in the C-6-Alkynylation of Protected 2′-Deoxyadenosine. *Tetrahedron* **2009**, *65*, 4085–4091.

(10) Saga, Y.; Motoki, R.; Makino, S.; Shimizu, Y.; Kanai, M.; Shibasaki, M. Catalytic Asymmetric Synthesis of R207910. *J. Am. Chem. Soc.* **2010**, *132*, 7905–7907.

(11) Lu, B.; Li, C.; Zhang, L. Gold-Catalyzed Highly Regioselective Oxidation of C−C Triple Bonds without Acid Additives: Propargyl Moieties as Masked α,β-Unsaturated Carbonyls. *J. Am. Chem. Soc.* **2010**, *132*, 14070–14072.

(12) Roesch, K. R.; Larock, R. C. Synthesis of Isoindolo[2,1-a]Indoles by the Palladium-Catalyzed Annulation of Internal Acetylenes. *J. Org. Chem.* **2001**, *66*, 412–420.

(13) Zhao, J.; Yu, Y.; Ma, S. Ligand Effects on the Pd-Catalyzed Cross-Coupling Reaction of 3-Iodoalk-2-Enoates with Propargyl/1,2-Allenylic Metallic Species: An Efficient Regiodivergent Synthesis of 2,4,5-Trienoates. *Chem. Eur. J.* **2010**, *16*, 74–80.

(14) Gelman, D.; Buchwald, S. L. Efficient Palladium-Catalyzed Coupling of Aryl Chlorides and Tosylates with Terminal Alkynes: Use of a Copper Cocatalyst Inhibits the Reaction. *Angew. Chem. Int. Ed.* **2003**, *42*, 5993–5996.

(15) Yoshida, M.; Hayashi, M.; Shishido, K. Palladium-Catalyzed Diastereoselective Coupling of Propargylic Oxiranes with Terminal Alkynes. *Org. Lett.* **2007**, *9*, 1643–1646.

(16) Yu, F.; Li, P.; Wang, B.; Han, K. Reversible Near-Infrared Fluorescent Probe Introducing Tellurium to Mimetic Glutathione Peroxidase for Monitoring the Redox Cycles between Peroxynitrite and Glutathione in Vivo. *J. Am. Chem. Soc.* **2013**, *135*, 7674–7680.

(17) Bruno, N. C.; Tudge, M. T.; Buchwald, S. L. Design and Preparation of New Palladium Precatalysts for C–C and C–N Cross-Coupling Reactions. *Chem. Sci.* **2013**, *4*, 916–920.

(18) Glenadel, Q.; Alazet, S.; Baert, F.; Billard, T. Multigram Scale Syntheses of First and Second Generation of Trifluoromethanesulfenamide Reagents. *Org. Process Res. Dev.* **2016**, *20* (5), 960–964. https://doi.org/10.1021/acs.oprd.6b00062.
(19) Dalgleish, S.; Robertson, N. A Stable near IR Switchable Electrochromic Polymer Based on an Indole-Substituted Nickel Dithiolene. *Chem. Commun.* **2009**, No. 39, 5826–5828. https://doi.org/10.1039/B913174D.
(20) Zheng, G.; Zhao, J.; Li, Z.; Zhang, Q.; Sun, J.; Sun, H.; Zhang, Q. Highly Regio- and Stereoselective Intermolecular Seleno- and Thioamination of Alkynes. *Chem. – Eur. J.* **2016**, *22* (10), 3513–3518. https://doi.org/10.1002/chem.201504534.
(21) Ackermann, L.; Lygin, A. V. Cationic Ruthenium(II) Catalysts for Oxidative C–H/N–H Bond Functionalizations of Anilines with Removable Directing Group: Synthesis of Indoles in Water. *Org. Lett.* **2012**, *14* (3), 764–767. https://doi.org/10.1021/ol203309y.
(22) Dolomanov, O. V.; Bourhis, L. J.; Gildea, R. J.; Howard, J. a. K.; Puschmann, H. OLEX2: A Complete Structure Solution, Refinement and Analysis Program. *J Appl Cryst* **2009**, *42* (2), 339–341. https://doi.org/10.1107/S0021889808042726.
